# Electron Density of Adipose Tissues Determined by Phase-Contrast Computed Tomography Provides a Measure for Mitochondrial Density and Fat Content

**DOI:** 10.3389/fphys.2018.00707

**Published:** 2018-06-15

**Authors:** Lorenz Birnbacher, Stefanie Maurer, Katharina Scheidt, Julia Herzen, Franz Pfeiffer, Tobias Fromme

**Affiliations:** ^1^Biomedical Physics, Department of Physics and Munich School of BioEngineering, Technical University of Munich, Munich, Germany; ^2^Chair of Molecular Nutritional Medicine, TUM School of Life Sciences, Technical University of Munich, Munich, Germany; ^3^Else Kröner-Fresenius Center for Nutritional Medicine, Technical University of Munich, Freising, Germany; ^4^Department of Diagnostic and Interventional Radiology, Klinikum rechts der Isar, Technical University of Munich, Munich, Germany; ^5^Institute for Advanced Study, Technical University of Munich, Munich, Germany

**Keywords:** phase-contrast computed tomography, brown adipose tissue, white adipose tissue, brite adipocyte, beige adipocyte, electron density, browning, postnatal

## Abstract

Phase-contrast computed tomography (PCCT) is an X-ray-based imaging method measuring differences in the refractive index during tissue passage. While conventional X-ray techniques rely on the absorption of radiation due to differing tissue-specific attenuation coefficients, PCCT enables the determination of the electron density (ED). By the analysis of respective phantoms and *ex vivo* specimens, we identified the components responsible for different electron densities in murine adipose tissue depots to be cellular fat and mitochondrial content, two parameters typically different between white adipose tissue (WAT) and brown adipose tissue (BAT). Brown adipocytes provide mammals with a means of non-shivering thermogenesis to defend normothermia in a cold environment. Brown adipocytes are found in dedicated BAT depots and interspersed within white fat depots, a cell type referred to as brite (brown in white) adipocyte. Localization and quantification of brown and brite adipocytes *in situ* allows an estimate of depot thermogenic capacity and potential contribution to maximal metabolic rate in the cold. We utilized PCCT to infer the composition of white, brite, and brown adipose tissue from ED of individual depots. As proof of principle, we imaged mice 10, 20, and 30 days of age. During this period, several WAT depots are known to undergo transient browning. Based on ED, classical WAT and BAT could be clearly distinguished. Retroperitoneal and inguinal WAT depots increased transiently in ED during the known remodeling from white to brite/brown and back to white. We systematically analyzed 18 anatomically defined adipose tissue locations and identified changes in fat content and mitochondrial density that imply an orchestrated pattern of simultaneous browning and whitening on the organismic level. Taken together, PCCT provides a three-dimensional imaging technique to visualize ED of tissues *in situ*. Within the adipose organ, ED provides a measure of mitochondrial density and fat content. Depending on experimental setting, these constitute surrogate markers of cellular distribution of white, brite, and brown adipocytes and thereby an estimate of thermogenic capacity.

## Introduction

Brown adipose tissue (BAT) is a mammalian heater organ providing adaptive, non-shivering thermogenesis to defend normothermia in a cold environment (for a review, see Klingenspor et al., 2017). Thermogenic brown adipocytes reside both in characteristic, homogeneous BAT depots and interspersed within white adipose tissue (WAT) depots. In rodents, the number of these brite (brown in white) fat cells varies considerably depending on depot, ambient temperature acclimation state, age, diet, and genetic factors (Young et al., 1984; Guerra et al., 1998; Fromme and Klingenspor, 2011; Vitali et al., 2012; Lasar et al., 2013; Li et al., 2014a,b). “Browning” and “whitening” of WAT depots in model organisms are intensely studied phenomena due to their potential to serve as pharmacological targets allowing to increase the number of brown adipocytes in human patients of metabolic disease (Tseng et al., 2010; Bartelt and Heeren, 2014). Until now, however, analysis of brown adipocyte number in a rodent depot requires dissection and subsequent gene expression analysis and/or histology, which effectively prevents repeated measures in the same animal. While genetic mouse models with brown adipocyte specific luciferase expression can be repeatedly subjected to *in vivo* imaging, the methodology suffers from interfering parameters that change over time during experiments, i.e., light absorption by fur and overlaying WAT tissue of differing thickness, skin pigmentation or altered luminogenic substrate distribution owing to changes in body mass and composition (Galmozzi et al., 2014; Mao et al., 2017).

A viable alternative seems X-ray computed tomography (CT) that has been demonstrated to allow a good discrimination of anatomically defined WAT and BAT depots (Lubura et al., 2012). While conventional X-ray techniques rely on the absorption of X-rays due to differing tissue specific attenuation coefficients, phase-contrast computed tomography (PCCT) measures differences in refractive index during tissue passage, enabling determination of the electron density (ED; electrons per cubic nanometer; Weitkamp et al., 2005; Pfeiffer et al., 2006, 2007; Herzen et al., 2009). In this study, we established ED as a surrogate measure for brown adipocyte content in murine fat depots and investigated the underlying properties governing tissue ED.

## Materials and Methods

### Animal Samples

We obtained male mice 10, 20, and 30 days of age of the 129S6/SvEvTac strain (three per age, total *n* = 9). All animals were bred in our specific pathogen-free facility in in accordance with the German Animal Welfare Law. Mice were killed, submerged in an excess volume of 4% paraformaldehyde in phosphate buffered saline, and fixed for 3–4 days at 5°C. The entire animal was subjected to PCCT.

Four more male mice 30 days of age of the 129S6/SvEvTac strain were killed to prepare liver, epididymal, inguinal, and interscapular adipose tissue. Adipose tissue depots were fixed in an excess volume of 4% paraformaldehyde in phosphate buffered saline, weighed, and subjected to PCCT.

### Determination of Cellular Fat and Mitochondrial Content

After measurement of ED by PCCT, we isolated DNA from paraformaldehyde fixated tissues by an established protocol (Campos and Gilbert, 2012). During the procedure, a distinctly separate layer of fat formed, was punctured by a syringe needle to aspirate the subjacent solution, dried, and weighed. Concentration of the final DNA isolate was determined spectrophotometrically (NanoQuant Plate, Tecan). We assumed a genome mass of 3 pg per cell. Mitochondrial and nuclear DNA was quantified by quantitative PCR directed against unique target sequences with the following primers: nuclear forward tttacaggatctccaagattcaga, nuclear reverse gatcaccccatgtgaacaaa; mitochondrial forward caaatttacccgctactcaactc, mitochondrial reverse gctataatttttcgtatttgtgtttgg.

Phantoms were generated from liver mitochondria isolated as described previously (Jastroch et al., 2007). Mitochondrial protein content was determined by the Biuret method and different amounts of mitochondrial suspension mixed with solidifying agarose in water. ED of phantoms was determined by PCCT.

### Phase-Contrast Computed Tomography

The laboratory grating-based PCCT setup consisted of an X-ray source, three X-ray gratings, and an X-ray detector. The X-ray source was an Enraf Nonius FR591 rotating anode with a molybdenum target operating at 40 kV and 70 mA. The detector was a single-photon counting Pilatus II 100K detector (Dectris Ltd., Baden, Switzerland) with a pixel size of 172 μm × 172 μm. The first grating was an absorption grating enabling the use of X-ray sources with limited coherence, as often occurring in a laboratory environment. The second grating was a phase grating creating a periodic, re-occurring interference pattern (Talbot effect) designed for a beam energy of 27 keV and a phase-shift of π. The third grating was an absorption grating allowing to resolve the interference pattern. The periods of all three gratings were 5.4 μm and the gratings were fabricated by the Institute of Microstructure, Karlsruhe Institute of Technology (KIT, Karlsruhe, Germany). Both inter-grating distances were 85.7 cm. The PCCT setup allows the simultaneous retrieval of the differential phase-contrast and conventional absorption contrast data using the phase-stepping technique. The number of phase-steps was 11 and the exposure time was 5 s. The tomographic scans were performed with 1200 projections and the data were reconstructed using statistical iterative reconstruction (Hahn et al., 2015). In order to avoid phase-wrapping, the sample was immersed in a water container. The effective energy of the phase-contrast interferometer was 27 keV. Due to cone beam magnification, the effective pixel size was 85 μm. More details can be found in previous publications (Weitkamp et al., 2005; Pfeiffer et al., 2006; Birnbacher et al., 2016).

### Data Analysis

The ED related to the refractive index and the differential phase-contrast signal was calculated using effective energy calibration as described earlier (Herzen et al., 2009). As the extent of the samples was larger than the detector height, several separate tomographic data sets were stitched together for one sample. To improve data quality, we used bilateral filtering (Allner et al., 2016). We analyzed raw data with a clinical 3D software suite (Osirix; Rosset et al., 2004). To attribute electron densities to adipose tissue depots, we defined a maximal area section in one characteristic plane per anatomically defined depot and determined mean ED and area. We refrained from statistical evaluation of group comparisons in this low sample number prospective study. Correlations were analyzed by a linear regressions model (GraphPad Prism 6).

Scan data of all nine animals can be downloaded in the form of image stacks (**Supplementary Material**). Furthermore, BAT and WAT of one 30-days-old mouse were reconstructed as a navigable 3D model for educational purposes (segmented with VGStudio Max 2.1; **Supplementary Material**).

## Results

### White and Brown Adipose Tissue Can Be Differentiated by Electron Density

**FIGURE 1:**
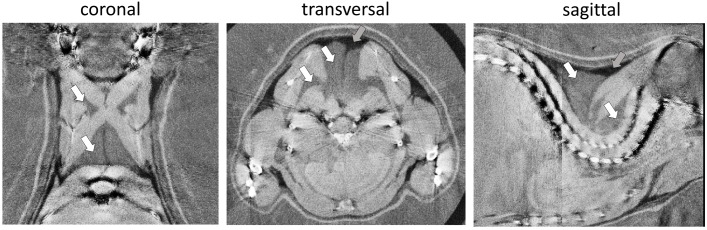
Representative phase-contrast computed tomography images of the murine upper thoracic region in the coronal, transversal, and sagittal plane. Brown adipose tissue (white arrows) can clearly be distinguished from overlaying white adipose tissue (darker, less electron dense; gray arrows) and bounding muscular structures (lighter, more electron dense). The electron density is displayed in a linear range from 310 to 350 e/nm^3^.

Phase-contrast computed tomography determines ED in electrons per nanometer cubed (e/nm^3^) compared to multifactorial radiodensity/attenuation coefficient measured by classical CT. We explored the potential benefit of PCCT to distinguish murine WAT and BAT depots. In the three-dimensionally reconstructed tomography of 30-days-old mice, the characteristic interscapular/subscapular BAT depot was readily discernible (**Figure 1**). The ED of BAT was in the range of 339.3–340.3 e/nm^3^ and lower than most lean tissues (e.g., ventricle 354–357 e/nm^3^, leg muscle 347–352 e/nm^3^). Epididymal white fat, on the other hand, displayed the lowest ED of any tissue type in the murine body with 306.4–312.2 e/nm^3^ and was thus clearly distinguishable from BAT. ED proved to be suitable to clearly delimit WAT and BAT depots and was well in line with published values (Woodard and White, 1986).

### Fat Content and Mitochondria Determine Adipose Tissue Electron Density

**FIGURE 2:**
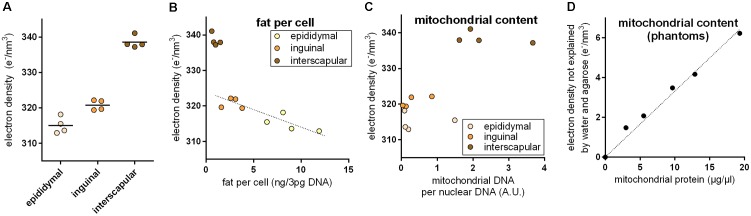
**(A)** Electron density of explants from the epididymal white, inguinal brite, and interscapular brown adipose tissue depot. Electron density increased with assumed brown adipocyte content. **(B)** Electron density of white adipose tissue explants correlated with fat content per cell, and while brown adipose tissue was electron denser than expected from fat content. The dotted line represents a linear regression across inguinal and epididymal explants (*R*^2^ 0.78, *p* < 0.05). **(C)** High electron density in brown adipose tissue coincided with high mitochondrial content. **(D)** In agarose phantoms with varying additions of embedded mitochondria, mitochondrial protein content correlated with residual electron density. The dotted lined represents a linear regression across all phantoms (*R*^2^ 0.98, *p* < 0.05).

Brown adipocytes are not only found in classical BAT depots, but also interspersed within WAT depots. The number of these brite (brown in white) adipocytes is very different among WAT depots and physiological states. In 30-days-old mice, we applied PCCT to determine ED of adipose tissue explants with a typically different content of brown adipocytes. ED was lowest in epididymal (white), higher in inguinal (brite), and highest in interscapular (brown) adipose tissue, proportional to the assumed content of brown adipocytes in these depots (**Figure 2A**). In depots of intermediate ED, we often observed sub-structures of higher ED irregularly pervading the depot in line with the notion of a heterogeneous localization of brite adipocyte nests reported earlier (Barreau et al., 2016; **Supplementary Figure S1**).

Multilocular brown adipocytes contain less triglyceride per cell than unilocular white adipocytes. Since fat is less electron dense than water, we tested the hypothesis that a different fat content caused ED differences between fat depots. Indeed, triglyceride per cell correlated well with ED in epididymal white and inguinal brite fat (**Figure 2B**); 1 ng fat per white adipocyte decreased ED by 0.866 e/nm^3^. Interscapular brown fat, however, markedly deviated from the white/brite fat regression and displayed a much higher ED than predicted by fat content. We therefore considered the extremely high mitochondrial protein content of brown adipocytes as a second parameter influencing ED. Mitochondrial DNA per cellular DNA ratios coincided with high ED in this tissue (**Figure 2C**). We therefore generated phantoms of different mitochondrial density by adding isolated murine mitochondria to solidifying agarose in water. The fractional ED not explained by agarose and water correlated very well with mitochondrial protein content and allowed us to quantify the ED contribution per μg/μl mitochondrial protein to 0.333 e/nm^3^ (**Figure 2D**). Assuming fat and mitochondrial protein content to be the major determinants of ED in adipose tissue, the approximatively 20–25 e/nm^3^ difference between white and brown fat can be plausibly explained by a difference of around 5 ng mitochondrial protein, 25 ng fat, or any combination of these per adipocyte of 50 μm diameter (for comparison: a 50 μm diameter sphere of water weighs 65.5 ng).

### Electron Density May Serve as a Surrogate Measure for Brown Adipocyte Content

White adipose tissue and BAT depots had a different ED (**Figure 2**). We probed the possibility to determine composition of mixed brite depots by PCCT. The number of brown/brite adipocytes within murine WAT depots is highly flexible and depends on many factors including depot, ambient temperature, mouse strain, and developmental stage. We chose a model of well-characterized composition changes occurring after birth in the retroperitoneal and inguinal adipose tissue depot. On postnatal day 10, these depots appear mostly white, markedly brown/brite on day 20 (“browning”), and return to a white phenotype on day 30 (“whitening”; Xue et al., 2007; Lasar et al., 2013). We applied this knowledge on timed appearance and disappearance of brite cells to test whether ED as measured by PCCT is a suitable surrogate measure for brite adipocyte content of a depot.

**FIGURE 3:**
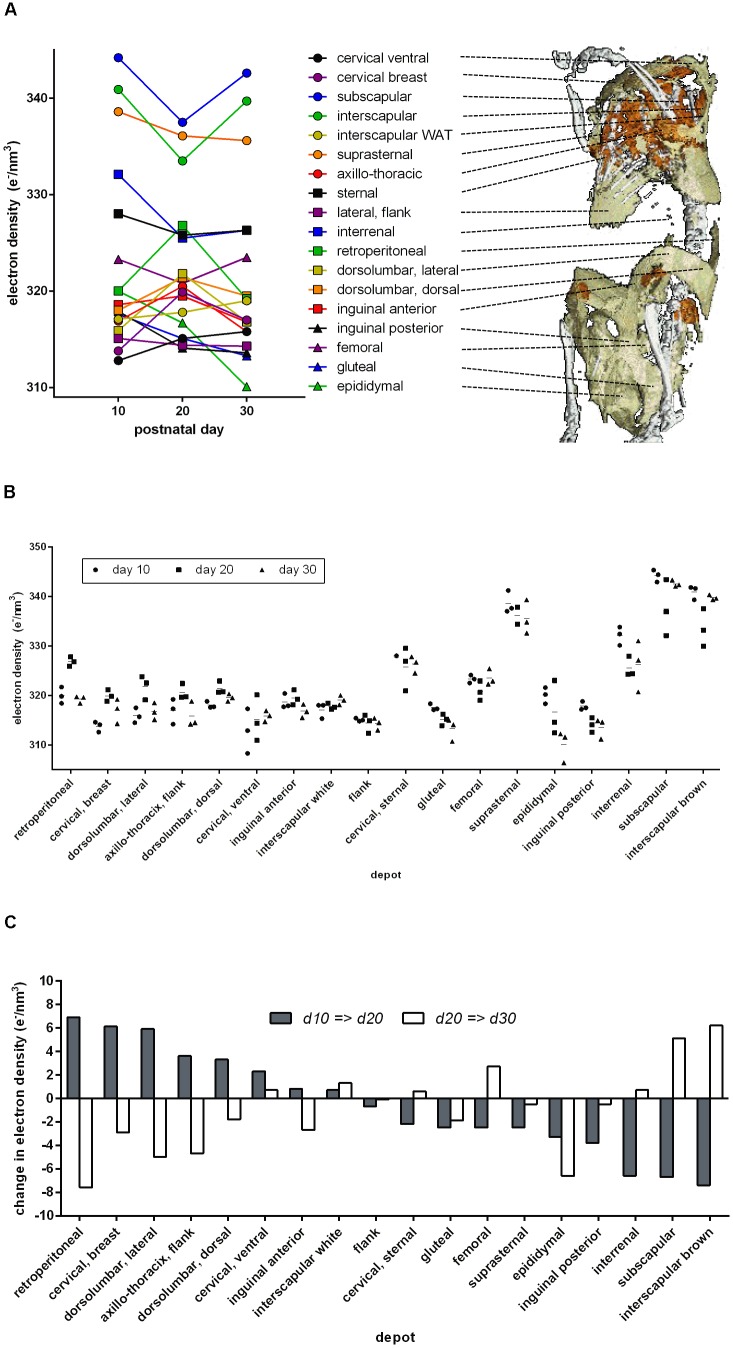
Electron density and anatomical location of 18 defined adipose tissues at three time points after birth. **(A)** Dots indicate mean values of three individuals, and individual values are given in **B**. Lines connect the respective depot along the time axis. The 3D anatomical model underlying the snapshot shown here is available as a download in the supplement. **(B)** Individual electron densities at three time points after birth, *n* = 3. Anatomical location as outlined in **A**. **(C)** Change in mean electron density from day 10 to 20 (gray bars) and from day 20 to 30 (white bars). Some adipose tissues transiently “brown” at day 20 (gray bar positive, white bar negative, e.g., retroperitoneal), while others “whiten” (gray bar negative, white bar positive, e.g., subscapular).

In mice 10, 20, or 30 days of age, we analyzed 18 anatomical adipose tissue locations in a maximal area plane characteristically defined for each depot. Indeed, ED of retroperitoneal and inguinal WAT increased during browning from day 10 to day 20 and decreased to the level of white fat on day 30 (**Figure 3**). In these depots, ED followed the known pattern of altered cellular composition and thus proved a surrogate marker of brite adipocyte content. The approximate 5 e/nm^3^ ED change during browning corresponds to a difference of around 1 ng mitochondrial protein, 6 ng fat, or any combination of these per adipocyte of 50 μm diameter (for comparison: a 50 μm diameter sphere of water weighs 65.5 ng).

Beyond retroperitoneal and inguinal fat, many depots displayed a transient alteration in ED during postnatal day 20. Surprisingly, the transient browning event in WAT was accompanied by changes in ED implying transient whitening of classical BAT depots, e.g., interscapular and subscapular BAT. Both visualized as absolute ED (**Figure 3B**) and as change in ED (**Figure 3C**), it is obvious that the phenomenon of postnatal adipose tissue remodeling is in fact bidirectional and dependent on the specific depot in question.

**FIGURE 4:**
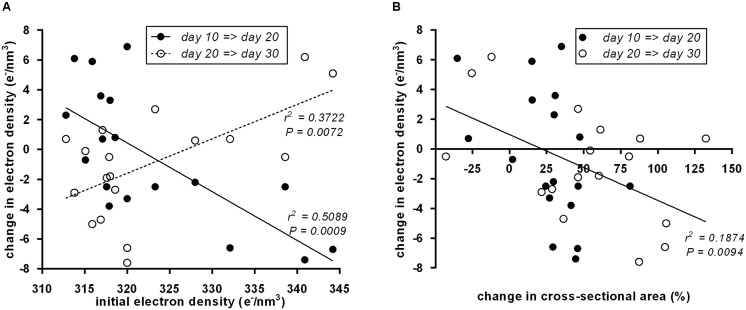
**(A)** Initial electron density at postnatal day 10 of 18 murine adipose tissue depots was a predictor of subsequent electron density changes. From day 10 to day 20 (black dots), the correlation was negative, i.e., white fat becomes brown and brown fat becomes white. From day 20 to day 30 (white dots), the reversal of transient “browning” and “whitening” manifested in a positive correlation. Dots represent mean values (*n* = 3). **(B)** Within both periods, day 10 to day 20 (black dots) and day 20 to day 30 (white dots), a change in depots size negatively correlated to changes in electron density, i.e., depots shrank/grew less during “browning” and grew more during “whitening.” Dots represent mean values (*n* = 3).

To systematically address whether the initial “brownness”/“whiteness” of a depot predicts the direction of its postnatal remodeling, we plotted initial ED at day 10 vs. the change in ED (**Figure 4A**). Indeed, we detected a significant negative correlation: the less brown adipocytes a depot contains, the more it browns from day 10 to day 20, and vice versa. All of these changes are transient as evident from the converse, positive correlation of initial ED to changes occurring from day 20 to 30.

Previously, we reported growth arrest of adipose tissue depots undergoing postnatal browning (Lasar et al., 2013). Here, we used the cross-sectional area of the depot-intersecting plane analyzed as a surrogate for depot size. Strikingly, the established connection between attenuated growth and browning proved reversible: the growth in tissue size accelerated during whitening as interpreted from decreasing ED. This rule universally applied to all size and ED changes determined: both from postnatal day 10 to 20 and from day 20 to 30 (**Figure 4B**).

In summary, we established PCCT-determined ED as a surrogate measure for mitochondrial density and fat content. As such, it provides information on brown adipocyte content in adipose tissues. Based on this assumption, murine adipose tissues undergo global remodeling during the first 4 weeks after birth. White depots transiently decreased growth during browning and accelerated growth during re-whitening. Brown depots mirrored this phenomenon simultaneously in reverse.

## Discussion

Activation and recruitment of BAT is considered a viable target for the pharmacological treatment of human metabolic disease (Tseng et al., 2010; Gerngross et al., 2017). The phenomena of murine browning/whitening, i.e., the change in brite adipocyte number in white fat, constitute a valuable model to study the mechanisms regulating such alterations in the number of thermogenic brown fat cells (Bartelt and Heeren, 2014; Li et al., 2014b). A major drawback impeding *in vivo* research is the lack of reliable methods to follow browning over the time course of an experimental intervention within the same individual.

We here for the first time demonstrate that ED as determined by PCCT can be employed to determine adipocyte fat and mitochondrial content. Depending on scenario, these may serve as surrogate measures of brown and brite adipocyte content in adipose tissue depots. In this conceptual study, we employed dead and fixated animals to allow long exposure times (21 h/mouse) for maximal data quality. Furthermore, in our experimental non-gantry setup, the sample rotated instead of the entire machine as realized in clinical CT systems. Finally, the samples were immersed in water to avoid streak artifacts. For all these reasons, the current setup is incompatible with anesthetized mice. There is, however, no fundamental obstacle to perform such measurements in anesthetized mice in an optimized setup. Commercially available gantry phase-contrast micro CT systems (e.g., Bruker Skyscan 1294) are already suitable to acquire such data in less than 1 h, albeit at a far lower spatial and ED resolution than reported here and prone to movement/breathing artifacts (Bech et al., 2013; Velroyen et al., 2015). We expect next generation machines to overcome these limitations with improved interferometer quality and respiratory/cardiac gating routines. From this proof-of-concept study, it is clear that remaining obstacles are of technical and not of fundamental nature.

The ED of fresh tissue is practically unchanged by paraformaldehyde fixation (Willner et al., 2015). WAT, brite, and BAT feature different ED due to a difference in cellular fat as well as mitochondrial content, a finding well in line with the established morphological differences (multi- vs. unilocularity; high vs. low mitochondrial density) between brown/brite and white adipocytes (Li et al., 2014b; Klingenspor et al., 2017).

Importantly, we showed ED to be a measure of adipocyte mitochondrial and triglyceride content, not of thermogenic uncoupling protein 1 or any other specific structure of brown or brite adipocytes. While changes in brite adipocyte content will clearly lead to concomitant changes in fat and mitochondrial content, the reasoning is less unambiguous vice versa. In fact, these parameters are inherently different in adipose tissue depots and subject to alterations in response to physiological (e.g., dietary) stimuli (Palou et al., 2009; Schottl et al., 2015a,b). To which extent such changes are consequence of altered brite cell density is subject to interpretation and will be dependent on the chosen experimental scenario.

As proof-of-principle, we analyzed a well-characterized model of altered brite cell abundance, i.e., the postnatal transition of retroperitoneal and inguinal WAT from white (day 10) to brite (day 20) and back to white (day 30). This process is of varying amplitude in different strains of mice and especially prominent in the strain chosen here, 129S6/SvEvTac (Xue et al., 2007; Lasar et al., 2013). Indeed, we were able to observe changes in ED concomitant with browning and whitening in these tissues as observed in our earlier study (Lasar et al., 2013). Assuming this correlation to be causal, the browning of retroperitoneal and inguinal WAT is accompanied by a massive, organism-wide remodeling of adipose tissue depots with white depots undergoing transient browning and, vice versa, brown adipose tissues becoming transiently whiter. During all changes and in depots of any composition, browning was associated with attenuated tissue growth and whitening with accelerated expansion.

The physiological reason for postnatal adipose tissue remodeling remains unknown. We previously speculated that the initial cold exposure of hairless mice after birth requires the transient recruitment of thermogenic brite cells that become obsolete with the advent of dense fur and an increasing body size (Lasar et al., 2013). In the light of our new data indicating a parallel BAT whitening, this hypothesis seems highly unlikely. Possibly, the radical changes in macronutrient utilization from carbohydrate (*in utero*) to milk fat (early postnatal, day 10) to complex solid food (weaning period, around day 20) require adipose tissue remodeling. The physiological adaptation to these diet changes is dominantly regulated by fibroblast growth factor 21 (FGF21; Hondares et al., 2010; Gavalda-Navarro et al., 2015). FGF21 is a pleiotropic peptide hormone indeed known to be implicated in many adipose tissue adaptations including autocrine secretion, adipose tissue browning, and substrate utilization (Hondares et al., 2011; Fisher et al., 2012; Keipert et al., 2015; Jeanson et al., 2016; Villarroya et al., 2017). It will certainly be informative to look at postnatal adipose tissues of mice devoid of FGF21 to explore this concept.

## Conclusion

Taken together, we here demonstrate the applicability of PCCT measured ED to follow adipocyte fat and mitochondrial content. Depending on scenario, these parameters may serve as surrogate markers for adipose tissue browning and whitening in mice. Thus, PCCT has the potential to evolve into the urgently needed methodology to determine brite adipocyte content repeatedly in WAT depots during long experimental interventions.

## Ethics Statement

This study was carried out in accordance with the German Animal Welfare Act. It is exempt from prior committee approval by §7(2) German Animal Welfare Act, because all experimental procedures were carried out after death of the animals.

## Author Contributions

LB contrived and constructed the PCCT imaging system and performed the PCCT measurements including processing and reconstruction. SM assisted in animal breeding and preparation. KS performed data stitching and 3D modeling. JH and FP contrived the PCCT imaging system and provided technical counsel on PCCT. TF devised the study, determined fat and mitochondrial content, analyzed and interpreted all data, and drafted the manuscript. All authors read and approved the manuscript.

## Conflict of Interest Statement

The authors declare that the research was conducted in the absence of any commercial or financial relationships that could be construed as a potential conflict of interest.

## Supplementary Material

FIGURE S1 Phase-contrast computed tomography images of the inguinal region of one example mouse 30 days of age in the coronal, transversal, and sagittal plane. Dotted lines indicate plane positions. Dashed lines envelope the anterior inguinal/lateral dorsolumbar adipose tissue depot. Irregular, light (high ED) structures of putative brite adipocyte nests traverse the dark (low ED) extent of white adipose tissue.
