# Supplementary figures and images for: Electron Density of Adipose Tissues Determined by Phase-Contrast Computed Tomography Provides a Measure for Mitochondrial Density and Fat Content
Source: Front Physiol. 2018 Jun 15;9:707. doi: 10.3389/fphys.2018.00707 (PMC6013718; doi:10.3389/fphys.2018.00707)

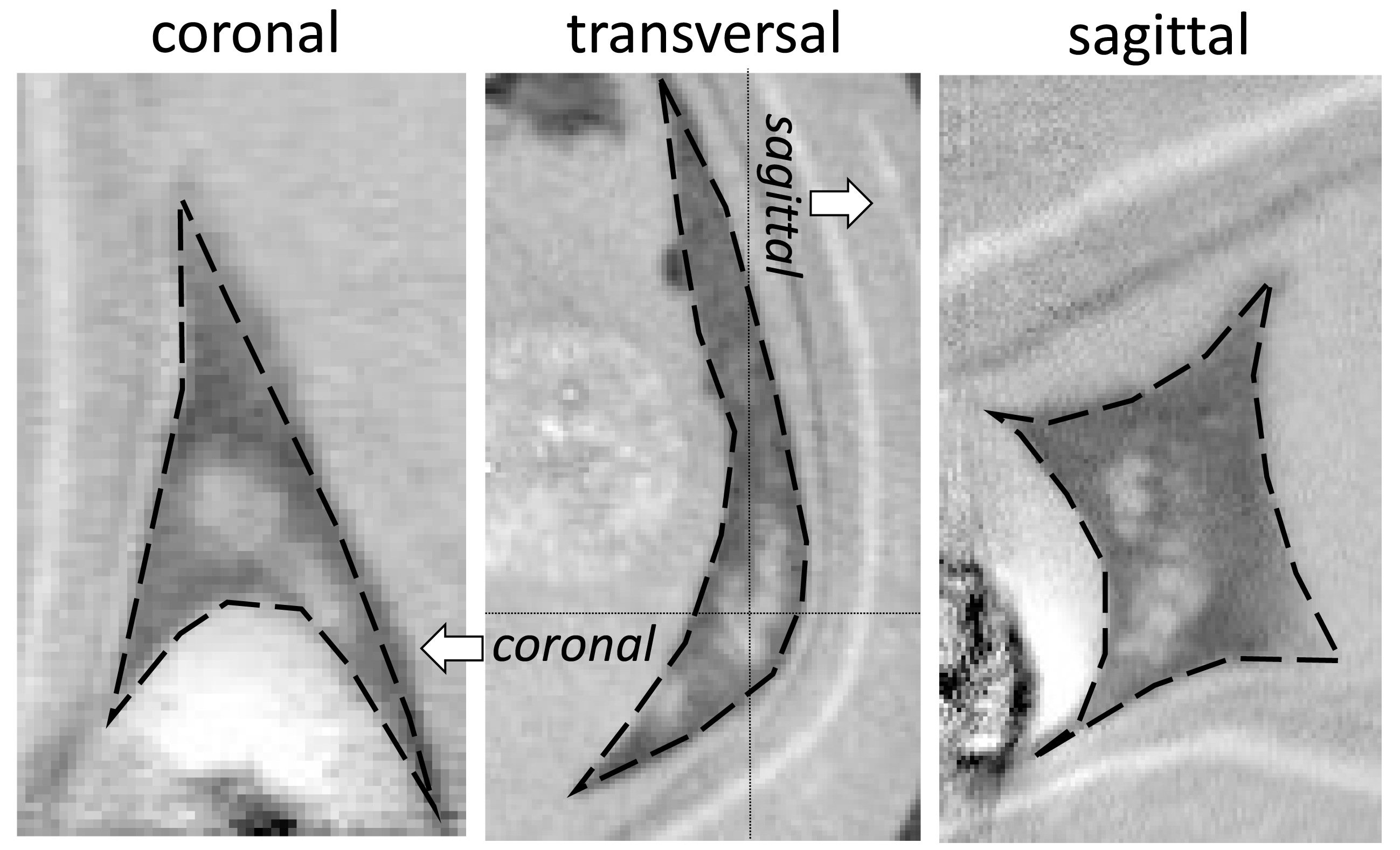

Supplement: FIGURE S1 — Phase-contrast computed tomography images of the inguinal region of one example mouse 30 days of age in the coronal, transversal, and sagittal plane. Dotted lines indicate plane positions. Dashed lines envelope the anterior inguinal/lateral dorsolumbar adipose tissue depot. Irregular, light (high ED) structures of putative brite adipocyte nests traverse the dark (low ED) extent of white adipose tissue. [file Image_1.TIF]

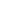

Supplement: Supplementary file 2 [file Data_Sheet_1.zip › [vg-project] BAT_project/656D707479.vgp]

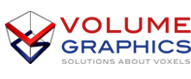

Supplement: Supplementary file 2 [file Data_Sheet_1.zip › [vg-project] BAT_project/686561646C696E65.vgp]

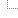

Supplement: Supplementary file 2 [file Data_Sheet_1.zip › [vg-project] BAT_project/6A626F74746F6D.vgp]

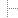

Supplement: Supplementary file 2 [file Data_Sheet_1.zip › [vg-project] BAT_project/6A6F696E.vgp]

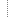

Supplement: Supplementary file 2 [file Data_Sheet_1.zip › [vg-project] BAT_project/6C696E65.vgp]

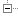

Supplement: Supplementary file 2 [file Data_Sheet_1.zip › [vg-project] BAT_project/6D626F746F6D.vgp]

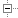

Supplement: Supplementary file 2 [file Data_Sheet_1.zip › [vg-project] BAT_project/6D696E7573.vgp]

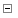

Supplement: Supplementary file 2 [file Data_Sheet_1.zip › [vg-project] BAT_project/6E6C6D696E7573.vgp]

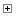

Supplement: Supplementary file 2 [file Data_Sheet_1.zip › [vg-project] BAT_project/6E6C706C7573.vgp]

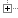

Supplement: Supplementary file 2 [file Data_Sheet_1.zip › [vg-project] BAT_project/70626F74746F6D.vgp]

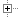

Supplement: Supplementary file 2 [file Data_Sheet_1.zip › [vg-project] BAT_project/706C7573.vgp]

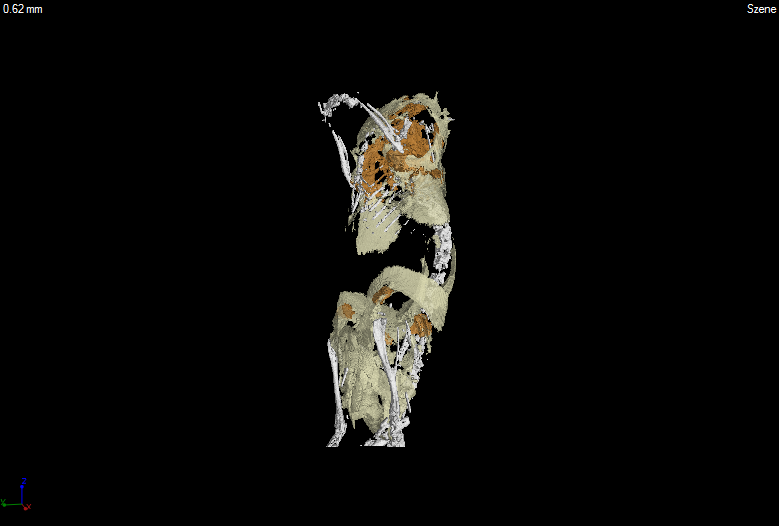

Supplement: Supplementary file 2 [file Data_Sheet_1.zip › [vg-project] BAT_project/70726576696477.vgp]

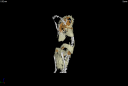

Supplement: Supplementary file 2 [file Data_Sheet_1.zip › [vg-project] BAT_project/7468756D626E61696C.vgp]
