# Supplementary material for: Electron Density of Adipose Tissues Determined by Phase-Contrast Computed Tomography Provides a Measure for Mitochondrial Density and Fat Content
Source: Front Physiol. 2018 Jun 15;9:707. doi: 10.3389/fphys.2018.00707 (PMC6013718; doi:10.3389/fphys.2018.00707)
Supplement: Supplementary file 2 [file Data_Sheet_1.zip › [vg-project] BAT_project/696E666F70616765.vgp]

| open all | close all | Scanner Manufacturer Information |  |  | | --- | --- | | Name: |  | | Adresse: |  | | Internetseite: |  | | Scanner: |  | | Scansoftware: |  |  Component Information |  |  | | --- | --- | | Beschreibung: |  | | Losnummer: |  | | Seriennummer: |  |  Scan Information |  |  | | --- | --- | | Röhrenspannung: |  | | Röhrenstromstärke: |  | | Scandauer: |  | | Rekonstruktionsdauer: |  | | Gesamtverarbeitungszeit: |  | | Rekonstruktionsalgorithmus: |  | | Scanmethode: |  | | Geometrie: |  | | Integrationszeit: |  | | Filtern: |  | | Projektionsanzahl: |  | | Datum, Zeit: |  | | Benutzer: |  |  Reconstruction Parameters NO RECONSTRUCTION PARAMETERS AVAILABLE  Import Settings |  |  |  |  | | --- | --- | --- | --- | | Source/Type: | Reconstructed Volume | | | | Name: | Volumen 1 | | | | Axes swap mode: | XYZT | | | | Mirror axes: | None | | | | Data mapping: | Ramp | | | | Data range source mapping: | -1...1 | | | | Data range destination mapping: | -1...1 | | | | Data type mapping: | Unknown | | | | Voxel skip: | 0 | 0 | 0 | | Auto region of interest: | Off | | Region of interest (min): | 0 | 0 | 0 | | Region of interest (max): | -1 | -1 | -1 | | Slice interpolation mode: | Off | | | | Slice interpolation threshold: | 0 | | | | Resolution [mm]: | 1 | 1 | 1 | | Resampling mode: | Off | | | | Auto histogram mode: | Off | | Lower auto histogram boundary at (%): | 0 | | Upper auto histogram boundary at (%): | 0 |  Analysis Information |  |  | | --- | --- | | Wall thickness: | 0 | | Defect detection: | 0 | | Nominal/actual comparison: | 0 | | Number of reference objects: | 0 |  File List (678) | files (678) | dimensions | format | type | endian | header | | --- | --- | --- | --- | --- | --- | | C:/.../mou...D000.raw | not available | raw | Unknown | Unknown | 0 | | C:/.../mou...D001.raw | not available | raw | Unknown | Unknown | 0 | | C:/.../mou...D002.raw | not available | raw | Unknown | Unknown | 0 | | C:/.../mou...D003.raw | not available | raw | Unknown | Unknown | 0 | | C:/.../mou...D004.raw | not available | raw | Unknown | Unknown | 0 | | C:/.../mou...D005.raw | not available | raw | Unknown | Unknown | 0 | | C:/.../mou...D006.raw | not available | raw | Unknown | Unknown | 0 | | C:/.../mou...D007.raw | not available | raw | Unknown | Unknown | 0 | | C:/.../mou...D008.raw | not available | raw | Unknown | Unknown | 0 | | C:/.../mou...D009.raw | not available | raw | Unknown | Unknown | 0 | | C:/.../mou...D010.raw | not available | raw | Unknown | Unknown | 0 | | C:/.../mou...D011.raw | not available | raw | Unknown | Unknown | 0 | | C:/.../mou...D012.raw | not available | raw | Unknown | Unknown | 0 | | C:/.../mou...D013.raw | not available | raw | Unknown | Unknown | 0 | | C:/.../mou...D014.raw | not available | raw | Unknown | Unknown | 0 | | C:/.../mou...D015.raw | not available | raw | Unknown | Unknown | 0 | | C:/.../mou...D016.raw | not available | raw | Unknown | Unknown | 0 | | C:/.../mou...D017.raw | not available | raw | Unknown | Unknown | 0 | | C:/.../mou...D018.raw | not available | raw | Unknown | Unknown | 0 | | C:/.../mou...D019.raw | not available | raw | Unknown | Unknown | 0 | | C:/.../mou...D020.raw | not available | raw | Unknown | Unknown | 0 | | C:/.../mou...D021.raw | not available | raw | Unknown | Unknown | 0 | | C:/.../mou...D022.raw | not available | raw | Unknown | Unknown | 0 | | C:/.../mou...D023.raw | not available | raw | Unknown | Unknown | 0 | | C:/.../mou...D024.raw | not available | raw | Unknown | Unknown | 0 | | C:/.../mou...D025.raw | not available | raw | Unknown | Unknown | 0 | | C:/.../mou...D026.raw | not available | raw | Unknown | Unknown | 0 | | C:/.../mou...D027.raw | not available | raw | Unknown | Unknown | 0 | | C:/.../mou...D028.raw | not available | raw | Unknown | Unknown | 0 | | C:/.../mou...D029.raw | not available | raw | Unknown | Unknown | 0 | | C:/.../mou...D030.raw | not available | raw | Unknown | Unknown | 0 | | C:/.../mou...D031.raw | not available | raw | Unknown | Unknown | 0 | | C:/.../mou...D032.raw | not available | raw | Unknown | Unknown | 0 | | C:/.../mou...D033.raw | not available | raw | Unknown | Unknown | 0 | | C:/.../mou...D034.raw | not available | raw | Unknown | Unknown | 0 | | C:/.../mou...D035.raw | not available | raw | Unknown | Unknown | 0 | | C:/.../mou...D036.raw | not available | raw | Unknown | Unknown | 0 | | C:/.../mou...D037.raw | not available | raw | Unknown | Unknown | 0 | | C:/.../mou...D038.raw | not available | raw | Unknown | Unknown | 0 | | C:/.../mou...D039.raw | not available | raw | Unknown | Unknown | 0 | | C:/.../mou...D040.raw | not available | raw | Unknown | Unknown | 0 | | C:/.../mou...D041.raw | not available | raw | Unknown | Unknown | 0 | | C:/.../mou...D042.raw | not available | raw | Unknown | Unknown | 0 | | C:/.../mou...D043.raw | not available | raw | Unknown | Unknown | 0 | | C:/.../mou...D044.raw | not available | raw | Unknown | Unknown | 0 | | C:/.../mou...D045.raw | not available | raw | Unknown | Unknown | 0 | | C:/.../mou...D046.raw | not available | raw | Unknown | Unknown | 0 | | C:/.../mou...D047.raw | not available | raw | Unknown | Unknown | 0 | | C:/.../mou...D048.raw | not available | raw | Unknown | Unknown | 0 | | C:/.../mou...D049.raw | not available | raw | Unknown | Unknown | 0 | | C:/.../mou...D050.raw | not available | raw | Unknown | Unknown | 0 | | C:/.../mou...D051.raw | not available | raw | Unknown | Unknown | 0 | | C:/.../mou...D052.raw | not available | raw | Unknown | Unknown | 0 | | C:/.../mou...D053.raw | not available | raw | Unknown | Unknown | 0 | | C:/.../mou...D054.raw | not available | raw | Unknown | Unknown | 0 | | C:/.../mou...D055.raw | not available | raw | Unknown | Unknown | 0 | | C:/.../mou...D056.raw | not available | raw | Unknown | Unknown | 0 | | C:/.../mou...D057.raw | not available | raw | Unknown | Unknown | 0 | | C:/.../mou...D058.raw | not available | raw | Unknown | Unknown | 0 | | C:/.../mou...D059.raw | not available | raw | Unknown | Unknown | 0 | | C:/.../mou...D060.raw | not available | raw | Unknown | Unknown | 0 | | C:/.../mou...D061.raw | not available | raw | Unknown | Unknown | 0 | | C:/.../mou...D062.raw | not available | raw | Unknown | Unknown | 0 | | C:/.../mou...D063.raw | not available | raw | Unknown | Unknown | 0 | | C:/.../mou...D064.raw | not available | raw | Unknown | Unknown | 0 | | C:/.../mou...D065.raw | not available | raw | Unknown | Unknown | 0 | | C:/.../mou...D066.raw | not available | raw | Unknown | Unknown | 0 | | C:/.../mou...D067.raw | not available | raw | Unknown | Unknown | 0 | | C:/.../mou...D068.raw | not available | raw | Unknown | Unknown | 0 | | C:/.../mou...D069.raw | not available | raw | Unknown | Unknown | 0 | | C:/.../mou...D070.raw | not available | raw | Unknown | Unknown | 0 | | C:/.../mou...D071.raw | not available | raw | Unknown | Unknown | 0 | | C:/.../mou...D072.raw | not available | raw | Unknown | Unknown | 0 | | C:/.../mou...D073.raw | not available | raw | Unknown | Unknown | 0 | | C:/.../mou...D074.raw | not available | raw | Unknown | Unknown | 0 | | C:/.../mou...D075.raw | not available | raw | Unknown | Unknown | 0 | | C:/.../mou...D076.raw | not available | raw | Unknown | Unknown | 0 | | C:/.../mou...D077.raw | not available | raw | Unknown | Unknown | 0 | | C:/.../mou...D078.raw | not available | raw | Unknown | Unknown | 0 | | C:/.../mou...D079.raw | not available | raw | Unknown | Unknown | 0 | | C:/.../mou...D080.raw | not available | raw | Unknown | Unknown | 0 | | C:/.../mou...D081.raw | not available | raw | Unknown | Unknown | 0 | | C:/.../mou...D082.raw | not available | raw | Unknown | Unknown | 0 | | C:/.../mou...D083.raw | not available | raw | Unknown | Unknown | 0 | | C:/.../mou...D084.raw | not available | raw | Unknown | Unknown | 0 | | C:/.../mou...D085.raw | not available | raw | Unknown | Unknown | 0 | | C:/.../mou...D086.raw | not available | raw | Unknown | Unknown | 0 | | C:/.../mou...D087.raw | not available | raw | Unknown | Unknown | 0 | | C:/.../mou...D088.raw | not available | raw | Unknown | Unknown | 0 | | C:/.../mou...D089.raw | not available | raw | Unknown | Unknown | 0 | | C:/.../mou...D090.raw | not available | raw | Unknown | Unknown | 0 | | C:/.../mou...D091.raw | not available | raw | Unknown | Unknown | 0 | | C:/.../mou...D092.raw | not available | raw | Unknown | Unknown | 0 | | C:/.../mou...D093.raw | not available | raw | Unknown | Unknown | 0 | | C:/.../mou...D094.raw | not available | raw | Unknown | Unknown | 0 | | C:/.../mou...D095.raw | not available | raw | Unknown | Unknown | 0 | | C:/.../mou...D096.raw | not available | raw | Unknown | Unknown | 0 | | C:/.../mou...D097.raw | not available | raw | Unknown | Unknown | 0 | | C:/.../mou...D098.raw | not available | raw | Unknown | Unknown | 0 | | C:/.../mou...D099.raw | not available | raw | Unknown | Unknown | 0 | | C:/.../mou...D100.raw | not available | raw | Unknown | Unknown | 0 | | C:/.../mou...D101.raw | not available | raw | Unknown | Unknown | 0 | | C:/.../mou...D102.raw | not available | raw | Unknown | Unknown | 0 | | C:/.../mou...D103.raw | not available | raw | Unknown | Unknown | 0 | | C:/.../mou...D104.raw | not available | raw | Unknown | Unknown | 0 | | C:/.../mou...D105.raw | not available | raw | Unknown | Unknown | 0 | | C:/.../mou...D106.raw | not available | raw | Unknown | Unknown | 0 | | C:/.../mou...D107.raw | not available | raw | Unknown | Unknown | 0 | | C:/.../mou...D108.raw | not available | raw | Unknown | Unknown | 0 | | C:/.../mou...D109.raw | not available | raw | Unknown | Unknown | 0 | | C:/.../mou...D110.raw | not available | raw | Unknown | Unknown | 0 | | C:/.../mou...D111.raw | not available | raw | Unknown | Unknown | 0 | | C:/.../mou...D112.raw | not available | raw | Unknown | Unknown | 0 | | C:/.../mou...D113.raw | not available | raw | Unknown | Unknown | 0 | | C:/.../mou...D114.raw | not available | raw | Unknown | Unknown | 0 | | C:/.../mou...D115.raw | not available | raw | Unknown | Unknown | 0 | | C:/.../mou...D116.raw | not available | raw | Unknown | Unknown | 0 | | C:/.../mou...D117.raw | not available | raw | Unknown | Unknown | 0 | | C:/.../mou...D118.raw | not available | raw | Unknown | Unknown | 0 | | C:/.../mou...D119.raw | not available | raw | Unknown | Unknown | 0 | | C:/.../mou...D120.raw | not available | raw | Unknown | Unknown | 0 | | C:/.../mou...D121.raw | not available | raw | Unknown | Unknown | 0 | | C:/.../mou...D122.raw | not available | raw | Unknown | Unknown | 0 | | C:/.../mou...D123.raw | not available | raw | Unknown | Unknown | 0 | | C:/.../mou...D124.raw | not available | raw | Unknown | Unknown | 0 | | C:/.../mou...D125.raw | not available | raw | Unknown | Unknown | 0 | | C:/.../mou...D126.raw | not available | raw | Unknown | Unknown | 0 | | C:/.../mou...D127.raw | not available | raw | Unknown | Unknown | 0 | | C:/.../mou...D128.raw | not available | raw | Unknown | Unknown | 0 | | C:/.../mou...D129.raw | not available | raw | Unknown | Unknown | 0 | | C:/.../mou...D130.raw | not available | raw | Unknown | Unknown | 0 | | C:/.../mou...D131.raw | not available | raw | Unknown | Unknown | 0 | | C:/.../mou...D132.raw | not available | raw | Unknown | Unknown | 0 | | C:/.../mou...D133.raw | not available | raw | Unknown | Unknown | 0 | | C:/.../mou...D134.raw | not available | raw | Unknown | Unknown | 0 | | C:/.../mou...D135.raw | not available | raw | Unknown | Unknown | 0 | | C:/.../mou...D136.raw | not available | raw | Unknown | Unknown | 0 | | C:/.../mou...D137.raw | not available | raw | Unknown | Unknown | 0 | | C:/.../mou...D138.raw | not available | raw | Unknown | Unknown | 0 | | C:/.../mou...D139.raw | not available | raw | Unknown | Unknown | 0 | | C:/.../mou...D140.raw | not available | raw | Unknown | Unknown | 0 | | C:/.../mou...D141.raw | not available | raw | Unknown | Unknown | 0 | | C:/.../mou...D142.raw | not available | raw | Unknown | Unknown | 0 | | C:/.../mou...D143.raw | not available | raw | Unknown | Unknown | 0 | | C:/.../mou...D144.raw | not available | raw | Unknown | Unknown | 0 | | C:/.../mou...D145.raw | not available | raw | Unknown | Unknown | 0 | | C:/.../mou...D146.raw | not available | raw | Unknown | Unknown | 0 | | C:/.../mou...D147.raw | not available | raw | Unknown | Unknown | 0 | | C:/.../mou...D148.raw | not available | raw | Unknown | Unknown | 0 | | C:/.../mou...D149.raw | not available | raw | Unknown | Unknown | 0 | | C:/.../mou...D150.raw | not available | raw | Unknown | Unknown | 0 | | C:/.../mou...D151.raw | not available | raw | Unknown | Unknown | 0 | | C:/.../mou...D152.raw | not available | raw | Unknown | Unknown | 0 | | C:/.../mou...D153.raw | not available | raw | Unknown | Unknown | 0 | | C:/.../mou...D154.raw | not available | raw | Unknown | Unknown | 0 | | C:/.../mou...D155.raw | not available | raw | Unknown | Unknown | 0 | | C:/.../mou...D156.raw | not available | raw | Unknown | Unknown | 0 | | C:/.../mou...D157.raw | not available | raw | Unknown | Unknown | 0 | | C:/.../mou...D158.raw | not available | raw | Unknown | Unknown | 0 | | C:/.../mou...D159.raw | not available | raw | Unknown | Unknown | 0 | | C:/.../mou...D160.raw | not available | raw | Unknown | Unknown | 0 | | C:/.../mou...D161.raw | not available | raw | Unknown | Unknown | 0 | | C:/.../mou...D162.raw | not available | raw | Unknown | Unknown | 0 | | C:/.../mou...D163.raw | not available | raw | Unknown | Unknown | 0 | | C:/.../mou...D164.raw | not available | raw | Unknown | Unknown | 0 | | C:/.../mou...D165.raw | not available | raw | Unknown | Unknown | 0 | | C:/.../mou...D166.raw | not available | raw | Unknown | Unknown | 0 | | C:/.../mou...D167.raw | not available | raw | Unknown | Unknown | 0 | | C:/.../mou...D168.raw | not available | raw | Unknown | Unknown | 0 | | C:/.../mou...D169.raw | not available | raw | Unknown | Unknown | 0 | | C:/.../mou...D170.raw | not available | raw | Unknown | Unknown | 0 | | C:/.../mou...D171.raw | not available | raw | Unknown | Unknown | 0 | | C:/.../mou...D172.raw | not available | raw | Unknown | Unknown | 0 | | C:/.../mou...D173.raw | not available | raw | Unknown | Unknown | 0 | | C:/.../mou...D174.raw | not available | raw | Unknown | Unknown | 0 | | C:/.../mou...D175.raw | not available | raw | Unknown | Unknown | 0 | | C:/.../mou...D176.raw | not available | raw | Unknown | Unknown | 0 | | C:/.../mou...D177.raw | not available | raw | Unknown | Unknown | 0 | | C:/.../mou...D178.raw | not available | raw | Unknown | Unknown | 0 | | C:/.../mou...D179.raw | not available | raw | Unknown | Unknown | 0 | | C:/.../mou...D180.raw | not available | raw | Unknown | Unknown | 0 | | C:/.../mou...D181.raw | not available | raw | Unknown | Unknown | 0 | | C:/.../mou...D182.raw | not available | raw | Unknown | Unknown | 0 | | C:/.../mou...D183.raw | not available | raw | Unknown | Unknown | 0 | | C:/.../mou...D184.raw | not available | raw | Unknown | Unknown | 0 | | C:/.../mou...D185.raw | not available | raw | Unknown | Unknown | 0 | | C:/.../mou...D186.raw | not available | raw | Unknown | Unknown | 0 | | C:/.../mou...D187.raw | not available | raw | Unknown | Unknown | 0 | | C:/.../mou...D188.raw | not available | raw | Unknown | Unknown | 0 | | C:/.../mou...D189.raw | not available | raw | Unknown | Unknown | 0 | | C:/.../mou...D190.raw | not available | raw | Unknown | Unknown | 0 | | C:/.../mou...D191.raw | not available | raw | Unknown | Unknown | 0 | | C:/.../mou...D192.raw | not available | raw | Unknown | Unknown | 0 | | C:/.../mou...D193.raw | not available | raw | Unknown | Unknown | 0 | | C:/.../mou...D194.raw | not available | raw | Unknown | Unknown | 0 | | C:/.../mou...D195.raw | not available | raw | Unknown | Unknown | 0 | | C:/.../mou...D196.raw | not available | raw | Unknown | Unknown | 0 | | C:/.../mou...D197.raw | not available | raw | Unknown | Unknown | 0 | | C:/.../mou...D198.raw | not available | raw | Unknown | Unknown | 0 | | C:/.../mou...D199.raw | not available | raw | Unknown | Unknown | 0 | | C:/.../mou...D200.raw | not available | raw | Unknown | Unknown | 0 | | C:/.../mou...D201.raw | not available | raw | Unknown | Unknown | 0 | | C:/.../mou...D202.raw | not available | raw | Unknown | Unknown | 0 | | C:/.../mou...D203.raw | not available | raw | Unknown | Unknown | 0 | | C:/.../mou...D204.raw | not available | raw | Unknown | Unknown | 0 | | C:/.../mou...D205.raw | not available | raw | Unknown | Unknown | 0 | | C:/.../mou...D206.raw | not available | raw | Unknown | Unknown | 0 | | C:/.../mou...D207.raw | not available | raw | Unknown | Unknown | 0 | | C:/.../mou...D208.raw | not available | raw | Unknown | Unknown | 0 | | C:/.../mou...D209.raw | not available | raw | Unknown | Unknown | 0 | | C:/.../mou...D210.raw | not available | raw | Unknown | Unknown | 0 | | C:/.../mou...D211.raw | not available | raw | Unknown | Unknown | 0 | | C:/.../mou...D212.raw | not available | raw | Unknown | Unknown | 0 | | C:/.../mou...D213.raw | not available | raw | Unknown | Unknown | 0 | | C:/.../mou...D214.raw | not available | raw | Unknown | Unknown | 0 | | C:/.../mou...D215.raw | not available | raw | Unknown | Unknown | 0 | | C:/.../mou...D216.raw | not available | raw | Unknown | Unknown | 0 | | C:/.../mou...D217.raw | not available | raw | Unknown | Unknown | 0 | | C:/.../mou...D218.raw | not available | raw | Unknown | Unknown | 0 | | C:/.../mou...D219.raw | not available | raw | Unknown | Unknown | 0 | | C:/.../mou...D220.raw | not available | raw | Unknown | Unknown | 0 | | C:/.../mou...D221.raw | not available | raw | Unknown | Unknown | 0 | | C:/.../mou...D222.raw | not available | raw | Unknown | Unknown | 0 | | C:/.../mou...D223.raw | not available | raw | Unknown | Unknown | 0 | | C:/.../mou...D224.raw | not available | raw | Unknown | Unknown | 0 | | C:/.../mou...D225.raw | not available | raw | Unknown | Unknown | 0 | | C:/.../mou...D226.raw | not available | raw | Unknown | Unknown | 0 | | C:/.../mou...D227.raw | not available | raw | Unknown | Unknown | 0 | | C:/.../mou...D228.raw | not available | raw | Unknown | Unknown | 0 | | C:/.../mou...D229.raw | not available | raw | Unknown | Unknown | 0 | | C:/.../mou...D230.raw | not available | raw | Unknown | Unknown | 0 | | C:/.../mou...D231.raw | not available | raw | Unknown | Unknown | 0 | | C:/.../mou...D232.raw | not available | raw | Unknown | Unknown | 0 | | C:/.../mou...D233.raw | not available | raw | Unknown | Unknown | 0 | | C:/.../mou...D234.raw | not available | raw | Unknown | Unknown | 0 | | C:/.../mou...D235.raw | not available | raw | Unknown | Unknown | 0 | | C:/.../mou...D236.raw | not available | raw | Unknown | Unknown | 0 | | C:/.../mou...D237.raw | not available | raw | Unknown | Unknown | 0 | | C:/.../mou...D238.raw | not available | raw | Unknown | Unknown | 0 | | C:/.../mou...D239.raw | not available | raw | Unknown | Unknown | 0 | | C:/.../mou...D240.raw | not available | raw | Unknown | Unknown | 0 | | C:/.../mou...D241.raw | not available | raw | Unknown | Unknown | 0 | | C:/.../mou...D242.raw | not available | raw | Unknown | Unknown | 0 | | C:/.../mou...D243.raw | not available | raw | Unknown | Unknown | 0 | | C:/.../mou...D244.raw | not available | raw | Unknown | Unknown | 0 | | C:/.../mou...D245.raw | not available | raw | Unknown | Unknown | 0 | | C:/.../mou...D246.raw | not available | raw | Unknown | Unknown | 0 | | C:/.../mou...D247.raw | not available | raw | Unknown | Unknown | 0 | | C:/.../mou...D248.raw | not available | raw | Unknown | Unknown | 0 | | C:/.../mou...D249.raw | not available | raw | Unknown | Unknown | 0 | | C:/.../mou...D250.raw | not available | raw | Unknown | Unknown | 0 | | C:/.../mou...D251.raw | not available | raw | Unknown | Unknown | 0 | | C:/.../mou...D252.raw | not available | raw | Unknown | Unknown | 0 | | C:/.../mou...D253.raw | not available | raw | Unknown | Unknown | 0 | | C:/.../mou...D254.raw | not available | raw | Unknown | Unknown | 0 | | C:/.../mou...D255.raw | not available | raw | Unknown | Unknown | 0 | | C:/.../mou...D256.raw | not available | raw | Unknown | Unknown | 0 | | C:/.../mou...D257.raw | not available | raw | Unknown | Unknown | 0 | | C:/.../mou...D258.raw | not available | raw | Unknown | Unknown | 0 | | C:/.../mou...D259.raw | not available | raw | Unknown | Unknown | 0 | | C:/.../mou...D260.raw | not available | raw | Unknown | Unknown | 0 | | C:/.../mou...D261.raw | not available | raw | Unknown | Unknown | 0 | | C:/.../mou...D262.raw | not available | raw | Unknown | Unknown | 0 | | C:/.../mou...D263.raw | not available | raw | Unknown | Unknown | 0 | | C:/.../mou...D264.raw | not available | raw | Unknown | Unknown | 0 | | C:/.../mou...D265.raw | not available | raw | Unknown | Unknown | 0 | | C:/.../mou...D266.raw | not available | raw | Unknown | Unknown | 0 | | C:/.../mou...D267.raw | not available | raw | Unknown | Unknown | 0 | | C:/.../mou...D268.raw | not available | raw | Unknown | Unknown | 0 | | C:/.../mou...D269.raw | not available | raw | Unknown | Unknown | 0 | | C:/.../mou...D270.raw | not available | raw | Unknown | Unknown | 0 | | C:/.../mou...D271.raw | not available | raw | Unknown | Unknown | 0 | | C:/.../mou...D272.raw | not available | raw | Unknown | Unknown | 0 | | C:/.../mou...D273.raw | not available | raw | Unknown | Unknown | 0 | | C:/.../mou...D274.raw | not available | raw | Unknown | Unknown | 0 | | C:/.../mou...D275.raw | not available | raw | Unknown | Unknown | 0 | | C:/.../mou...D276.raw | not available | raw | Unknown | Unknown | 0 | | C:/.../mou...D277.raw | not available | raw | Unknown | Unknown | 0 | | C:/.../mou...D278.raw | not available | raw | Unknown | Unknown | 0 | | C:/.../mou...D279.raw | not available | raw | Unknown | Unknown | 0 | | C:/.../mou...D280.raw | not available | raw | Unknown | Unknown | 0 | | C:/.../mou...D281.raw | not available | raw | Unknown | Unknown | 0 | | C:/.../mou...D282.raw | not available | raw | Unknown | Unknown | 0 | | C:/.../mou...D283.raw | not available | raw | Unknown | Unknown | 0 | | C:/.../mou...D284.raw | not available | raw | Unknown | Unknown | 0 | | C:/.../mou...D285.raw | not available | raw | Unknown | Unknown | 0 | | C:/.../mou...D286.raw | not available | raw | Unknown | Unknown | 0 | | C:/.../mou...D287.raw | not available | raw | Unknown | Unknown | 0 | | C:/.../mou...D288.raw | not available | raw | Unknown | Unknown | 0 | | C:/.../mou...D289.raw | not available | raw | Unknown | Unknown | 0 | | C:/.../mou...D290.raw | not available | raw | Unknown | Unknown | 0 | | C:/.../mou...D291.raw | not available | raw | Unknown | Unknown | 0 | | C:/.../mou...D292.raw | not available | raw | Unknown | Unknown | 0 | | C:/.../mou...D293.raw | not available | raw | Unknown | Unknown | 0 | | C:/.../mou...D294.raw | not available | raw | Unknown | Unknown | 0 | | C:/.../mou...D295.raw | not available | raw | Unknown | Unknown | 0 | | C:/.../mou...D296.raw | not available | raw | Unknown | Unknown | 0 | | C:/.../mou...D297.raw | not available | raw | Unknown | Unknown | 0 | | C:/.../mou...D298.raw | not available | raw | Unknown | Unknown | 0 | | C:/.../mou...D299.raw | not available | raw | Unknown | Unknown | 0 | | C:/.../mou...D300.raw | not available | raw | Unknown | Unknown | 0 | | C:/.../mou...D301.raw | not available | raw | Unknown | Unknown | 0 | | C:/.../mou...D302.raw | not available | raw | Unknown | Unknown | 0 | | C:/.../mou...D303.raw | not available | raw | Unknown | Unknown | 0 | | C:/.../mou...D304.raw | not available | raw | Unknown | Unknown | 0 | | C:/.../mou...D305.raw | not available | raw | Unknown | Unknown | 0 | | C:/.../mou...D306.raw | not available | raw | Unknown | Unknown | 0 | | C:/.../mou...D307.raw | not available | raw | Unknown | Unknown | 0 | | C:/.../mou...D308.raw | not available | raw | Unknown | Unknown | 0 | | C:/.../mou...D309.raw | not available | raw | Unknown | Unknown | 0 | | C:/.../mou...D310.raw | not available | raw | Unknown | Unknown | 0 | | C:/.../mou...D311.raw | not available | raw | Unknown | Unknown | 0 | | C:/.../mou...D312.raw | not available | raw | Unknown | Unknown | 0 | | C:/.../mou...D313.raw | not available | raw | Unknown | Unknown | 0 | | C:/.../mou...D314.raw | not available | raw | Unknown | Unknown | 0 | | C:/.../mou...D315.raw | not available | raw | Unknown | Unknown | 0 | | C:/.../mou...D316.raw | not available | raw | Unknown | Unknown | 0 | | C:/.../mou...D317.raw | not available | raw | Unknown | Unknown | 0 | | C:/.../mou...D318.raw | not available | raw | Unknown | Unknown | 0 | | C:/.../mou...D319.raw | not available | raw | Unknown | Unknown | 0 | | C:/.../mou...D320.raw | not available | raw | Unknown | Unknown | 0 | | C:/.../mou...D321.raw | not available | raw | Unknown | Unknown | 0 | | C:/.../mou...D322.raw | not available | raw | Unknown | Unknown | 0 | | C:/.../mou...D323.raw | not available | raw | Unknown | Unknown | 0 | | C:/.../mou...D324.raw | not available | raw | Unknown | Unknown | 0 | | C:/.../mou...D325.raw | not available | raw | Unknown | Unknown | 0 | | C:/.../mou...D326.raw | not available | raw | Unknown | Unknown | 0 | | C:/.../mou...D327.raw | not available | raw | Unknown | Unknown | 0 | | C:/.../mou...D328.raw | not available | raw | Unknown | Unknown | 0 | | C:/.../mou...D329.raw | not available | raw | Unknown | Unknown | 0 | | C:/.../mou...D330.raw | not available | raw | Unknown | Unknown | 0 | | C:/.../mou...D331.raw | not available | raw | Unknown | Unknown | 0 | | C:/.../mou...D332.raw | not available | raw | Unknown | Unknown | 0 | | C:/.../mou...D333.raw | not available | raw | Unknown | Unknown | 0 | | C:/.../mou...D334.raw | not available | raw | Unknown | Unknown | 0 | | C:/.../mou...D335.raw | not available | raw | Unknown | Unknown | 0 | | C:/.../mou...D336.raw | not available | raw | Unknown | Unknown | 0 | | C:/.../mou...D337.raw | not available | raw | Unknown | Unknown | 0 | | C:/.../mou...D338.raw | not available | raw | Unknown | Unknown | 0 | | C:/.../mou...D339.raw | not available | raw | Unknown | Unknown | 0 | | C:/.../mou...D340.raw | not available | raw | Unknown | Unknown | 0 | | C:/.../mou...D341.raw | not available | raw | Unknown | Unknown | 0 | | C:/.../mou...D342.raw | not available | raw | Unknown | Unknown | 0 | | C:/.../mou...D343.raw | not available | raw | Unknown | Unknown | 0 | | C:/.../mou...D344.raw | not available | raw | Unknown | Unknown | 0 | | C:/.../mou...D345.raw | not available | raw | Unknown | Unknown | 0 | | C:/.../mou...D346.raw | not available | raw | Unknown | Unknown | 0 | | C:/.../mou...D347.raw | not available | raw | Unknown | Unknown | 0 | | C:/.../mou...D348.raw | not available | raw | Unknown | Unknown | 0 | | C:/.../mou...D349.raw | not available | raw | Unknown | Unknown | 0 | | C:/.../mou...D350.raw | not available | raw | Unknown | Unknown | 0 | | C:/.../mou...D351.raw | not available | raw | Unknown | Unknown | 0 | | C:/.../mou...D352.raw | not available | raw | Unknown | Unknown | 0 | | C:/.../mou...D353.raw | not available | raw | Unknown | Unknown | 0 | | C:/.../mou...D354.raw | not available | raw | Unknown | Unknown | 0 | | C:/.../mou...D355.raw | not available | raw | Unknown | Unknown | 0 | | C:/.../mou...D356.raw | not available | raw | Unknown | Unknown | 0 | | C:/.../mou...D357.raw | not available | raw | Unknown | Unknown | 0 | | C:/.../mou...D358.raw | not available | raw | Unknown | Unknown | 0 | | C:/.../mou...D359.raw | not available | raw | Unknown | Unknown | 0 | | C:/.../mou...D360.raw | not available | raw | Unknown | Unknown | 0 | | C:/.../mou...D361.raw | not available | raw | Unknown | Unknown | 0 | | C:/.../mou...D362.raw | not available | raw | Unknown | Unknown | 0 | | C:/.../mou...D363.raw | not available | raw | Unknown | Unknown | 0 | | C:/.../mou...D364.raw | not available | raw | Unknown | Unknown | 0 | | C:/.../mou...D365.raw | not available | raw | Unknown | Unknown | 0 | | C:/.../mou...D366.raw | not available | raw | Unknown | Unknown | 0 | | C:/.../mou...D367.raw | not available | raw | Unknown | Unknown | 0 | | C:/.../mou...D368.raw | not available | raw | Unknown | Unknown | 0 | | C:/.../mou...D369.raw | not available | raw | Unknown | Unknown | 0 | | C:/.../mou...D370.raw | not available | raw | Unknown | Unknown | 0 | | C:/.../mou...D371.raw | not available | raw | Unknown | Unknown | 0 | | C:/.../mou...D372.raw | not available | raw | Unknown | Unknown | 0 | | C:/.../mou...D373.raw | not available | raw | Unknown | Unknown | 0 | | C:/.../mou...D374.raw | not available | raw | Unknown | Unknown | 0 | | C:/.../mou...D375.raw | not available | raw | Unknown | Unknown | 0 | | C:/.../mou...D376.raw | not available | raw | Unknown | Unknown | 0 | | C:/.../mou...D377.raw | not available | raw | Unknown | Unknown | 0 | | C:/.../mou...D378.raw | not available | raw | Unknown | Unknown | 0 | | C:/.../mou...D379.raw | not available | raw | Unknown | Unknown | 0 | | C:/.../mou...D380.raw | not available | raw | Unknown | Unknown | 0 | | C:/.../mou...D381.raw | not available | raw | Unknown | Unknown | 0 | | C:/.../mou...D382.raw | not available | raw | Unknown | Unknown | 0 | | C:/.../mou...D383.raw | not available | raw | Unknown | Unknown | 0 | | C:/.../mou...D384.raw | not available | raw | Unknown | Unknown | 0 | | C:/.../mou...D385.raw | not available | raw | Unknown | Unknown | 0 | | C:/.../mou...D386.raw | not available | raw | Unknown | Unknown | 0 | | C:/.../mou...D387.raw | not available | raw | Unknown | Unknown | 0 | | C:/.../mou...D388.raw | not available | raw | Unknown | Unknown | 0 | | C:/.../mou...D389.raw | not available | raw | Unknown | Unknown | 0 | | C:/.../mou...D390.raw | not available | raw | Unknown | Unknown | 0 | | C:/.../mou...D391.raw | not available | raw | Unknown | Unknown | 0 | | C:/.../mou...D392.raw | not available | raw | Unknown | Unknown | 0 | | C:/.../mou...D393.raw | not available | raw | Unknown | Unknown | 0 | | C:/.../mou...D394.raw | not available | raw | Unknown | Unknown | 0 | | C:/.../mou...D395.raw | not available | raw | Unknown | Unknown | 0 | | C:/.../mou...D396.raw | not available | raw | Unknown | Unknown | 0 | | C:/.../mou...D397.raw | not available | raw | Unknown | Unknown | 0 | | C:/.../mou...D398.raw | not available | raw | Unknown | Unknown | 0 | | C:/.../mou...D399.raw | not available | raw | Unknown | Unknown | 0 | | C:/.../mou...D400.raw | not available | raw | Unknown | Unknown | 0 | | C:/.../mou...D401.raw | not available | raw | Unknown | Unknown | 0 | | C:/.../mou...D402.raw | not available | raw | Unknown | Unknown | 0 | | C:/.../mou...D403.raw | not available | raw | Unknown | Unknown | 0 | | C:/.../mou...D404.raw | not available | raw | Unknown | Unknown | 0 | | C:/.../mou...D405.raw | not available | raw | Unknown | Unknown | 0 | | C:/.../mou...D406.raw | not available | raw | Unknown | Unknown | 0 | | C:/.../mou...D407.raw | not available | raw | Unknown | Unknown | 0 | | C:/.../mou...D408.raw | not available | raw | Unknown | Unknown | 0 | | C:/.../mou...D409.raw | not available | raw | Unknown | Unknown | 0 | | C:/.../mou...D410.raw | not available | raw | Unknown | Unknown | 0 | | C:/.../mou...D411.raw | not available | raw | Unknown | Unknown | 0 | | C:/.../mou...D412.raw | not available | raw | Unknown | Unknown | 0 | | C:/.../mou...D413.raw | not available | raw | Unknown | Unknown | 0 | | C:/.../mou...D414.raw | not available | raw | Unknown | Unknown | 0 | | C:/.../mou...D415.raw | not available | raw | Unknown | Unknown | 0 | | C:/.../mou...D416.raw | not available | raw | Unknown | Unknown | 0 | | C:/.../mou...D417.raw | not available | raw | Unknown | Unknown | 0 | | C:/.../mou...D418.raw | not available | raw | Unknown | Unknown | 0 | | C:/.../mou...D419.raw | not available | raw | Unknown | Unknown | 0 | | C:/.../mou...D420.raw | not available | raw | Unknown | Unknown | 0 | | C:/.../mou...D421.raw | not available | raw | Unknown | Unknown | 0 | | C:/.../mou...D422.raw | not available | raw | Unknown | Unknown | 0 | | C:/.../mou...D423.raw | not available | raw | Unknown | Unknown | 0 | | C:/.../mou...D424.raw | not available | raw | Unknown | Unknown | 0 | | C:/.../mou...D425.raw | not available | raw | Unknown | Unknown | 0 | | C:/.../mou...D426.raw | not available | raw | Unknown | Unknown | 0 | | C:/.../mou...D427.raw | not available | raw | Unknown | Unknown | 0 | | C:/.../mou...D428.raw | not available | raw | Unknown | Unknown | 0 | | C:/.../mou...D429.raw | not available | raw | Unknown | Unknown | 0 | | C:/.../mou...D430.raw | not available | raw | Unknown | Unknown | 0 | | C:/.../mou...D431.raw | not available | raw | Unknown | Unknown | 0 | | C:/.../mou...D432.raw | not available | raw | Unknown | Unknown | 0 | | C:/.../mou...D433.raw | not available | raw | Unknown | Unknown | 0 | | C:/.../mou...D434.raw | not available | raw | Unknown | Unknown | 0 | | C:/.../mou...D435.raw | not available | raw | Unknown | Unknown | 0 | | C:/.../mou...D436.raw | not available | raw | Unknown | Unknown | 0 | | C:/.../mou...D437.raw | not available | raw | Unknown | Unknown | 0 | | C:/.../mou...D438.raw | not available | raw | Unknown | Unknown | 0 | | C:/.../mou...D439.raw | not available | raw | Unknown | Unknown | 0 | | C:/.../mou...D440.raw | not available | raw | Unknown | Unknown | 0 | | C:/.../mou...D441.raw | not available | raw | Unknown | Unknown | 0 | | C:/.../mou...D442.raw | not available | raw | Unknown | Unknown | 0 | | C:/.../mou...D443.raw | not available | raw | Unknown | Unknown | 0 | | C:/.../mou...D444.raw | not available | raw | Unknown | Unknown | 0 | | C:/.../mou...D445.raw | not available | raw | Unknown | Unknown | 0 | | C:/.../mou...D446.raw | not available | raw | Unknown | Unknown | 0 | | C:/.../mou...D447.raw | not available | raw | Unknown | Unknown | 0 | | C:/.../mou...D448.raw | not available | raw | Unknown | Unknown | 0 | | C:/.../mou...D449.raw | not available | raw | Unknown | Unknown | 0 | | C:/.../mou...D450.raw | not available | raw | Unknown | Unknown | 0 | | C:/.../mou...D451.raw | not available | raw | Unknown | Unknown | 0 | | C:/.../mou...D452.raw | not available | raw | Unknown | Unknown | 0 | | C:/.../mou...D453.raw | not available | raw | Unknown | Unknown | 0 | | C:/.../mou...D454.raw | not available | raw | Unknown | Unknown | 0 | | C:/.../mou...D455.raw | not available | raw | Unknown | Unknown | 0 | | C:/.../mou...D456.raw | not available | raw | Unknown | Unknown | 0 | | C:/.../mou...D457.raw | not available | raw | Unknown | Unknown | 0 | | C:/.../mou...D458.raw | not available | raw | Unknown | Unknown | 0 | | C:/.../mou...D459.raw | not available | raw | Unknown | Unknown | 0 | | C:/.../mou...D460.raw | not available | raw | Unknown | Unknown | 0 | | C:/.../mou...D461.raw | not available | raw | Unknown | Unknown | 0 | | C:/.../mou...D462.raw | not available | raw | Unknown | Unknown | 0 | | C:/.../mou...D463.raw | not available | raw | Unknown | Unknown | 0 | | C:/.../mou...D464.raw | not available | raw | Unknown | Unknown | 0 | | C:/.../mou...D465.raw | not available | raw | Unknown | Unknown | 0 | | C:/.../mou...D466.raw | not available | raw | Unknown | Unknown | 0 | | C:/.../mou...D467.raw | not available | raw | Unknown | Unknown | 0 | | C:/.../mou...D468.raw | not available | raw | Unknown | Unknown | 0 | | C:/.../mou...D469.raw | not available | raw | Unknown | Unknown | 0 | | C:/.../mou...D470.raw | not available | raw | Unknown | Unknown | 0 | | C:/.../mou...D471.raw | not available | raw | Unknown | Unknown | 0 | | C:/.../mou...D472.raw | not available | raw | Unknown | Unknown | 0 | | C:/.../mou...D473.raw | not available | raw | Unknown | Unknown | 0 | | C:/.../mou...D474.raw | not available | raw | Unknown | Unknown | 0 | | C:/.../mou...D475.raw | not available | raw | Unknown | Unknown | 0 | | C:/.../mou...D476.raw | not available | raw | Unknown | Unknown | 0 | | C:/.../mou...D477.raw | not available | raw | Unknown | Unknown | 0 | | C:/.../mou...D478.raw | not available | raw | Unknown | Unknown | 0 | | C:/.../mou...D479.raw | not available | raw | Unknown | Unknown | 0 | | C:/.../mou...D480.raw | not available | raw | Unknown | Unknown | 0 | | C:/.../mou...D481.raw | not available | raw | Unknown | Unknown | 0 | | C:/.../mou...D482.raw | not available | raw | Unknown | Unknown | 0 | | C:/.../mou...D483.raw | not available | raw | Unknown | Unknown | 0 | | C:/.../mou...D484.raw | not available | raw | Unknown | Unknown | 0 | | C:/.../mou...D485.raw | not available | raw | Unknown | Unknown | 0 | | C:/.../mou...D486.raw | not available | raw | Unknown | Unknown | 0 | | C:/.../mou...D487.raw | not available | raw | Unknown | Unknown | 0 | | C:/.../mou...D488.raw | not available | raw | Unknown | Unknown | 0 | | C:/.../mou...D489.raw | not available | raw | Unknown | Unknown | 0 | | C:/.../mou...D490.raw | not available | raw | Unknown | Unknown | 0 | | C:/.../mou...D491.raw | not available | raw | Unknown | Unknown | 0 | | C:/.../mou...D492.raw | not available | raw | Unknown | Unknown | 0 | | C:/.../mou...D493.raw | not available | raw | Unknown | Unknown | 0 | | C:/.../mou...D494.raw | not available | raw | Unknown | Unknown | 0 | | C:/.../mou...D495.raw | not available | raw | Unknown | Unknown | 0 | | C:/.../mou...D496.raw | not available | raw | Unknown | Unknown | 0 | | C:/.../mou...D497.raw | not available | raw | Unknown | Unknown | 0 | | C:/.../mou...D498.raw | not available | raw | Unknown | Unknown | 0 | | C:/.../mou...D499.raw | not available | raw | Unknown | Unknown | 0 | | C:/.../mou...D500.raw | not available | raw | Unknown | Unknown | 0 | | C:/.../mou...D501.raw | not available | raw | Unknown | Unknown | 0 | | C:/.../mou...D502.raw | not available | raw | Unknown | Unknown | 0 | | C:/.../mou...D503.raw | not available | raw | Unknown | Unknown | 0 | | C:/.../mou...D504.raw | not available | raw | Unknown | Unknown | 0 | | C:/.../mou...D505.raw | not available | raw | Unknown | Unknown | 0 | | C:/.../mou...D506.raw | not available | raw | Unknown | Unknown | 0 | | C:/.../mou...D507.raw | not available | raw | Unknown | Unknown | 0 | | C:/.../mou...D508.raw | not available | raw | Unknown | Unknown | 0 | | C:/.../mou...D509.raw | not available | raw | Unknown | Unknown | 0 | | C:/.../mou...D510.raw | not available | raw | Unknown | Unknown | 0 | | C:/.../mou...D511.raw | not available | raw | Unknown | Unknown | 0 | | C:/.../mou...D512.raw | not available | raw | Unknown | Unknown | 0 | | C:/.../mou...D513.raw | not available | raw | Unknown | Unknown | 0 | | C:/.../mou...D514.raw | not available | raw | Unknown | Unknown | 0 | | C:/.../mou...D515.raw | not available | raw | Unknown | Unknown | 0 | | C:/.../mou...D516.raw | not available | raw | Unknown | Unknown | 0 | | C:/.../mou...D517.raw | not available | raw | Unknown | Unknown | 0 | | C:/.../mou...D518.raw | not available | raw | Unknown | Unknown | 0 | | C:/.../mou...D519.raw | not available | raw | Unknown | Unknown | 0 | | C:/.../mou...D520.raw | not available | raw | Unknown | Unknown | 0 | | C:/.../mou...D521.raw | not available | raw | Unknown | Unknown | 0 | | C:/.../mou...D522.raw | not available | raw | Unknown | Unknown | 0 | | C:/.../mou...D523.raw | not available | raw | Unknown | Unknown | 0 | | C:/.../mou...D524.raw | not available | raw | Unknown | Unknown | 0 | | C:/.../mou...D525.raw | not available | raw | Unknown | Unknown | 0 | | C:/.../mou...D526.raw | not available | raw | Unknown | Unknown | 0 | | C:/.../mou...D527.raw | not available | raw | Unknown | Unknown | 0 | | C:/.../mou...D528.raw | not available | raw | Unknown | Unknown | 0 | | C:/.../mou...D529.raw | not available | raw | Unknown | Unknown | 0 | | C:/.../mou...D530.raw | not available | raw | Unknown | Unknown | 0 | | C:/.../mou...D531.raw | not available | raw | Unknown | Unknown | 0 | | C:/.../mou...D532.raw | not available | raw | Unknown | Unknown | 0 | | C:/.../mou...D533.raw | not available | raw | Unknown | Unknown | 0 | | C:/.../mou...D534.raw | not available | raw | Unknown | Unknown | 0 | | C:/.../mou...D535.raw | not available | raw | Unknown | Unknown | 0 | | C:/.../mou...D536.raw | not available | raw | Unknown | Unknown | 0 | | C:/.../mou...D537.raw | not available | raw | Unknown | Unknown | 0 | | C:/.../mou...D538.raw | not available | raw | Unknown | Unknown | 0 | | C:/.../mou...D539.raw | not available | raw | Unknown | Unknown | 0 | | C:/.../mou...D540.raw | not available | raw | Unknown | Unknown | 0 | | C:/.../mou...D541.raw | not available | raw | Unknown | Unknown | 0 | | C:/.../mou...D542.raw | not available | raw | Unknown | Unknown | 0 | | C:/.../mou...D543.raw | not available | raw | Unknown | Unknown | 0 | | C:/.../mou...D544.raw | not available | raw | Unknown | Unknown | 0 | | C:/.../mou...D545.raw | not available | raw | Unknown | Unknown | 0 | | C:/.../mou...D546.raw | not available | raw | Unknown | Unknown | 0 | | C:/.../mou...D547.raw | not available | raw | Unknown | Unknown | 0 | | C:/.../mou...D548.raw | not available | raw | Unknown | Unknown | 0 | | C:/.../mou...D549.raw | not available | raw | Unknown | Unknown | 0 | | C:/.../mou...D550.raw | not available | raw | Unknown | Unknown | 0 | | C:/.../mou...D551.raw | not available | raw | Unknown | Unknown | 0 | | C:/.../mou...D552.raw | not available | raw | Unknown | Unknown | 0 | | C:/.../mou...D553.raw | not available | raw | Unknown | Unknown | 0 | | C:/.../mou...D554.raw | not available | raw | Unknown | Unknown | 0 | | C:/.../mou...D555.raw | not available | raw | Unknown | Unknown | 0 | | C:/.../mou...D556.raw | not available | raw | Unknown | Unknown | 0 | | C:/.../mou...D557.raw | not available | raw | Unknown | Unknown | 0 | | C:/.../mou...D558.raw | not available | raw | Unknown | Unknown | 0 | | C:/.../mou...D559.raw | not available | raw | Unknown | Unknown | 0 | | C:/.../mou...D560.raw | not available | raw | Unknown | Unknown | 0 | | C:/.../mou...D561.raw | not available | raw | Unknown | Unknown | 0 | | C:/.../mou...D562.raw | not available | raw | Unknown | Unknown | 0 | | C:/.../mou...D563.raw | not available | raw | Unknown | Unknown | 0 | | C:/.../mou...D564.raw | not available | raw | Unknown | Unknown | 0 | | C:/.../mou...D565.raw | not available | raw | Unknown | Unknown | 0 | | C:/.../mou...D566.raw | not available | raw | Unknown | Unknown | 0 | | C:/.../mou...D567.raw | not available | raw | Unknown | Unknown | 0 | | C:/.../mou...D568.raw | not available | raw | Unknown | Unknown | 0 | | C:/.../mou...D569.raw | not available | raw | Unknown | Unknown | 0 | | C:/.../mou...D570.raw | not available | raw | Unknown | Unknown | 0 | | C:/.../mou...D571.raw | not available | raw | Unknown | Unknown | 0 | | C:/.../mou...D572.raw | not available | raw | Unknown | Unknown | 0 | | C:/.../mou...D573.raw | not available | raw | Unknown | Unknown | 0 | | C:/.../mou...D574.raw | not available | raw | Unknown | Unknown | 0 | | C:/.../mou...D575.raw | not available | raw | Unknown | Unknown | 0 | | C:/.../mou...D576.raw | not available | raw | Unknown | Unknown | 0 | | C:/.../mou...D577.raw | not available | raw | Unknown | Unknown | 0 | | C:/.../mou...D578.raw | not available | raw | Unknown | Unknown | 0 | | C:/.../mou...D579.raw | not available | raw | Unknown | Unknown | 0 | | C:/.../mou...D580.raw | not available | raw | Unknown | Unknown | 0 | | C:/.../mou...D581.raw | not available | raw | Unknown | Unknown | 0 | | C:/.../mou...D582.raw | not available | raw | Unknown | Unknown | 0 | | C:/.../mou...D583.raw | not available | raw | Unknown | Unknown | 0 | | C:/.../mou...D584.raw | not available | raw | Unknown | Unknown | 0 | | C:/.../mou...D585.raw | not available | raw | Unknown | Unknown | 0 | | C:/.../mou...D586.raw | not available | raw | Unknown | Unknown | 0 | | C:/.../mou...D587.raw | not available | raw | Unknown | Unknown | 0 | | C:/.../mou...D588.raw | not available | raw | Unknown | Unknown | 0 | | C:/.../mou...D589.raw | not available | raw | Unknown | Unknown | 0 | | C:/.../mou...D590.raw | not available | raw | Unknown | Unknown | 0 | | C:/.../mou...D591.raw | not available | raw | Unknown | Unknown | 0 | | C:/.../mou...D592.raw | not available | raw | Unknown | Unknown | 0 | | C:/.../mou...D593.raw | not available | raw | Unknown | Unknown | 0 | | C:/.../mou...D594.raw | not available | raw | Unknown | Unknown | 0 | | C:/.../mou...D595.raw | not available | raw | Unknown | Unknown | 0 | | C:/.../mou...D596.raw | not available | raw | Unknown | Unknown | 0 | | C:/.../mou...D597.raw | not available | raw | Unknown | Unknown | 0 | | C:/.../mou...D598.raw | not available | raw | Unknown | Unknown | 0 | | C:/.../mou...D599.raw | not available | raw | Unknown | Unknown | 0 | | C:/.../mou...D600.raw | not available | raw | Unknown | Unknown | 0 | | C:/.../mou...D601.raw | not available | raw | Unknown | Unknown | 0 | | C:/.../mou...D602.raw | not available | raw | Unknown | Unknown | 0 | | C:/.../mou...D603.raw | not available | raw | Unknown | Unknown | 0 | | C:/.../mou...D604.raw | not available | raw | Unknown | Unknown | 0 | | C:/.../mou...D605.raw | not available | raw | Unknown | Unknown | 0 | | C:/.../mou...D606.raw | not available | raw | Unknown | Unknown | 0 | | C:/.../mou...D607.raw | not available | raw | Unknown | Unknown | 0 | | C:/.../mou...D608.raw | not available | raw | Unknown | Unknown | 0 | | C:/.../mou...D609.raw | not available | raw | Unknown | Unknown | 0 | | C:/.../mou...D610.raw | not available | raw | Unknown | Unknown | 0 | | C:/.../mou...D611.raw | not available | raw | Unknown | Unknown | 0 | | C:/.../mou...D612.raw | not available | raw | Unknown | Unknown | 0 | | C:/.../mou...D613.raw | not available | raw | Unknown | Unknown | 0 | | C:/.../mou...D614.raw | not available | raw | Unknown | Unknown | 0 | | C:/.../mou...D615.raw | not available | raw | Unknown | Unknown | 0 | | C:/.../mou...D616.raw | not available | raw | Unknown | Unknown | 0 | | C:/.../mou...D617.raw | not available | raw | Unknown | Unknown | 0 | | C:/.../mou...D618.raw | not available | raw | Unknown | Unknown | 0 | | C:/.../mou...D619.raw | not available | raw | Unknown | Unknown | 0 | | C:/.../mou...D620.raw | not available | raw | Unknown | Unknown | 0 | | C:/.../mou...D621.raw | not available | raw | Unknown | Unknown | 0 | | C:/.../mou...D622.raw | not available | raw | Unknown | Unknown | 0 | | C:/.../mou...D623.raw | not available | raw | Unknown | Unknown | 0 | | C:/.../mou...D624.raw | not available | raw | Unknown | Unknown | 0 | | C:/.../mou...D625.raw | not available | raw | Unknown | Unknown | 0 | | C:/.../mou...D626.raw | not available | raw | Unknown | Unknown | 0 | | C:/.../mou...D627.raw | not available | raw | Unknown | Unknown | 0 | | C:/.../mou...D628.raw | not available | raw | Unknown | Unknown | 0 | | C:/.../mou...D629.raw | not available | raw | Unknown | Unknown | 0 | | C:/.../mou...D630.raw | not available | raw | Unknown | Unknown | 0 | | C:/.../mou...D631.raw | not available | raw | Unknown | Unknown | 0 | | C:/.../mou...D632.raw | not available | raw | Unknown | Unknown | 0 | | C:/.../mou...D633.raw | not available | raw | Unknown | Unknown | 0 | | C:/.../mou...D634.raw | not available | raw | Unknown | Unknown | 0 | | C:/.../mou...D635.raw | not available | raw | Unknown | Unknown | 0 | | C:/.../mou...D636.raw | not available | raw | Unknown | Unknown | 0 | | C:/.../mou...D637.raw | not available | raw | Unknown | Unknown | 0 | | C:/.../mou...D638.raw | not available | raw | Unknown | Unknown | 0 | | C:/.../mou...D639.raw | not available | raw | Unknown | Unknown | 0 | | C:/.../mou...D640.raw | not available | raw | Unknown | Unknown | 0 | | C:/.../mou...D641.raw | not available | raw | Unknown | Unknown | 0 | | C:/.../mou...D642.raw | not available | raw | Unknown | Unknown | 0 | | C:/.../mou...D643.raw | not available | raw | Unknown | Unknown | 0 | | C:/.../mou...D644.raw | not available | raw | Unknown | Unknown | 0 | | C:/.../mou...D645.raw | not available | raw | Unknown | Unknown | 0 | | C:/.../mou...D646.raw | not available | raw | Unknown | Unknown | 0 | | C:/.../mou...D647.raw | not available | raw | Unknown | Unknown | 0 | | C:/.../mou...D648.raw | not available | raw | Unknown | Unknown | 0 | | C:/.../mou...D649.raw | not available | raw | Unknown | Unknown | 0 | | C:/.../mou...D650.raw | not available | raw | Unknown | Unknown | 0 | | C:/.../mou...D651.raw | not available | raw | Unknown | Unknown | 0 | | C:/.../mou...D652.raw | not available | raw | Unknown | Unknown | 0 | | C:/.../mou...D653.raw | not available | raw | Unknown | Unknown | 0 | | C:/.../mou...D654.raw | not available | raw | Unknown | Unknown | 0 | | C:/.../mou...D655.raw | not available | raw | Unknown | Unknown | 0 | | C:/.../mou...D656.raw | not available | raw | Unknown | Unknown | 0 | | C:/.../mou...D657.raw | not available | raw | Unknown | Unknown | 0 | | C:/.../mou...D658.raw | not available | raw | Unknown | Unknown | 0 | | C:/.../mou...D659.raw | not available | raw | Unknown | Unknown | 0 | | C:/.../mou...D660.raw | not available | raw | Unknown | Unknown | 0 | | C:/.../mou...D661.raw | not available | raw | Unknown | Unknown | 0 | | C:/.../mou...D662.raw | not available | raw | Unknown | Unknown | 0 | | C:/.../mou...D663.raw | not available | raw | Unknown | Unknown | 0 | | C:/.../mou...D664.raw | not available | raw | Unknown | Unknown | 0 | | C:/.../mou...D665.raw | not available | raw | Unknown | Unknown | 0 | | C:/.../mou...D666.raw | not available | raw | Unknown | Unknown | 0 | | C:/.../mou...D667.raw | not available | raw | Unknown | Unknown | 0 | | C:/.../mou...D668.raw | not available | raw | Unknown | Unknown | 0 | | C:/.../mou...D669.raw | not available | raw | Unknown | Unknown | 0 | | C:/.../mou...D670.raw | not available | raw | Unknown | Unknown | 0 | | C:/.../mou...D671.raw | not available | raw | Unknown | Unknown | 0 | | C:/.../mou...D672.raw | not available | raw | Unknown | Unknown | 0 | | C:/.../mou...D673.raw | not available | raw | Unknown | Unknown | 0 | | C:/.../mou...D674.raw | not available | raw | Unknown | Unknown | 0 | | C:/.../mou...D675.raw | not available | raw | Unknown | Unknown | 0 | | C:/.../mou...D676.raw | not available | raw | Unknown | Unknown | 0 | | C:/.../mou...D677.raw | not available | raw | Unknown | Unknown | 0 |  Scanner Manufacturer Information |  |  | | --- | --- | | Name: |  | | Adresse: |  | | Internetseite: |  | | Scanner: |  | | Scansoftware: |  |  Component Information |  |  | | --- | --- | | Beschreibung: |  | | Losnummer: |  | | Seriennummer: |  |  Scan Information |  |  | | --- | --- | | Röhrenspannung: |  | | Röhrenstromstärke: |  | | Scandauer: |  | | Rekonstruktionsdauer: |  | | Gesamtverarbeitungszeit: |  | | Rekonstruktionsalgorithmus: |  | | Scanmethode: |  | | Geometrie: |  | | Integrationszeit: |  | | Filtern: |  | | Projektionsanzahl: |  | | Datum, Zeit: |  | | Benutzer: |  |  Reconstruction Parameters NO RECONSTRUCTION PARAMETERS AVAILABLE  Import Settings |  |  |  |  | | --- | --- | --- | --- | | Source/Type: | Reconstructed Volume | | | | Name: | Vereinigung braun skelett aus Volumen 1 | | | | Axes swap mode: | XYZT | | | | Mirror axes: | None | | | | Data mapping: | Ramp | | | | Data range source mapping: | -1...1 | | | | Data range destination mapping: | -1...1 | | | | Data type mapping: | Unknown | | | | Voxel skip: | 0 | 0 | 0 | | Auto region of interest: | Off | | Region of interest (min): | 0 | 0 | 0 | | Region of interest (max): | -1 | -1 | -1 | | Slice interpolation mode: | Off | | | | Slice interpolation threshold: | 0 | | | | Resolution [mm]: | 1 | 1 | 1 | | Resampling mode: | Off | | | | Auto histogram mode: | Off | | Lower auto histogram boundary at (%): | 0 | | Upper auto histogram boundary at (%): | 0 |  Analysis Information |  |  | | --- | --- | | Wall thickness: | 0 | | Defect detection: | 0 | | Nominal/actual comparison: | 0 | | Number of reference objects: | 0 |  File List (678) | files (678) | dimensions | format | type | endian | header | | --- | --- | --- | --- | --- | --- | | C:/.../bra...4000.raw | not available | raw | Unknown | Unknown | 0 | | C:/.../bra...4001.raw | not available | raw | Unknown | Unknown | 0 | | C:/.../bra...4002.raw | not available | raw | Unknown | Unknown | 0 | | C:/.../bra...4003.raw | not available | raw | Unknown | Unknown | 0 | | C:/.../bra...4004.raw | not available | raw | Unknown | Unknown | 0 | | C:/.../bra...4005.raw | not available | raw | Unknown | Unknown | 0 | | C:/.../bra...4006.raw | not available | raw | Unknown | Unknown | 0 | | C:/.../bra...4007.raw | not available | raw | Unknown | Unknown | 0 | | C:/.../bra...4008.raw | not available | raw | Unknown | Unknown | 0 | | C:/.../bra...4009.raw | not available | raw | Unknown | Unknown | 0 | | C:/.../bra...4010.raw | not available | raw | Unknown | Unknown | 0 | | C:/.../bra...4011.raw | not available | raw | Unknown | Unknown | 0 | | C:/.../bra...4012.raw | not available | raw | Unknown | Unknown | 0 | | C:/.../bra...4013.raw | not available | raw | Unknown | Unknown | 0 | | C:/.../bra...4014.raw | not available | raw | Unknown | Unknown | 0 | | C:/.../bra...4015.raw | not available | raw | Unknown | Unknown | 0 | | C:/.../bra...4016.raw | not available | raw | Unknown | Unknown | 0 | | C:/.../bra...4017.raw | not available | raw | Unknown | Unknown | 0 | | C:/.../bra...4018.raw | not available | raw | Unknown | Unknown | 0 | | C:/.../bra...4019.raw | not available | raw | Unknown | Unknown | 0 | | C:/.../bra...4020.raw | not available | raw | Unknown | Unknown | 0 | | C:/.../bra...4021.raw | not available | raw | Unknown | Unknown | 0 | | C:/.../bra...4022.raw | not available | raw | Unknown | Unknown | 0 | | C:/.../bra...4023.raw | not available | raw | Unknown | Unknown | 0 | | C:/.../bra...4024.raw | not available | raw | Unknown | Unknown | 0 | | C:/.../bra...4025.raw | not available | raw | Unknown | Unknown | 0 | | C:/.../bra...4026.raw | not available | raw | Unknown | Unknown | 0 | | C:/.../bra...4027.raw | not available | raw | Unknown | Unknown | 0 | | C:/.../bra...4028.raw | not available | raw | Unknown | Unknown | 0 | | C:/.../bra...4029.raw | not available | raw | Unknown | Unknown | 0 | | C:/.../bra...4030.raw | not available | raw | Unknown | Unknown | 0 | | C:/.../bra...4031.raw | not available | raw | Unknown | Unknown | 0 | | C:/.../bra...4032.raw | not available | raw | Unknown | Unknown | 0 | | C:/.../bra...4033.raw | not available | raw | Unknown | Unknown | 0 | | C:/.../bra...4034.raw | not available | raw | Unknown | Unknown | 0 | | C:/.../bra...4035.raw | not available | raw | Unknown | Unknown | 0 | | C:/.../bra...4036.raw | not available | raw | Unknown | Unknown | 0 | | C:/.../bra...4037.raw | not available | raw | Unknown | Unknown | 0 | | C:/.../bra...4038.raw | not available | raw | Unknown | Unknown | 0 | | C:/.../bra...4039.raw | not available | raw | Unknown | Unknown | 0 | | C:/.../bra...4040.raw | not available | raw | Unknown | Unknown | 0 | | C:/.../bra...4041.raw | not available | raw | Unknown | Unknown | 0 | | C:/.../bra...4042.raw | not available | raw | Unknown | Unknown | 0 | | C:/.../bra...4043.raw | not available | raw | Unknown | Unknown | 0 | | C:/.../bra...4044.raw | not available | raw | Unknown | Unknown | 0 | | C:/.../bra...4045.raw | not available | raw | Unknown | Unknown | 0 | | C:/.../bra...4046.raw | not available | raw | Unknown | Unknown | 0 | | C:/.../bra...4047.raw | not available | raw | Unknown | Unknown | 0 | | C:/.../bra...4048.raw | not available | raw | Unknown | Unknown | 0 | | C:/.../bra...4049.raw | not available | raw | Unknown | Unknown | 0 | | C:/.../bra...4050.raw | not available | raw | Unknown | Unknown | 0 | | C:/.../bra...4051.raw | not available | raw | Unknown | Unknown | 0 | | C:/.../bra...4052.raw | not available | raw | Unknown | Unknown | 0 | | C:/.../bra...4053.raw | not available | raw | Unknown | Unknown | 0 | | C:/.../bra...4054.raw | not available | raw | Unknown | Unknown | 0 | | C:/.../bra...4055.raw | not available | raw | Unknown | Unknown | 0 | | C:/.../bra...4056.raw | not available | raw | Unknown | Unknown | 0 | | C:/.../bra...4057.raw | not available | raw | Unknown | Unknown | 0 | | C:/.../bra...4058.raw | not available | raw | Unknown | Unknown | 0 | | C:/.../bra...4059.raw | not available | raw | Unknown | Unknown | 0 | | C:/.../bra...4060.raw | not available | raw | Unknown | Unknown | 0 | | C:/.../bra...4061.raw | not available | raw | Unknown | Unknown | 0 | | C:/.../bra...4062.raw | not available | raw | Unknown | Unknown | 0 | | C:/.../bra...4063.raw | not available | raw | Unknown | Unknown | 0 | | C:/.../bra...4064.raw | not available | raw | Unknown | Unknown | 0 | | C:/.../bra...4065.raw | not available | raw | Unknown | Unknown | 0 | | C:/.../bra...4066.raw | not available | raw | Unknown | Unknown | 0 | | C:/.../bra...4067.raw | not available | raw | Unknown | Unknown | 0 | | C:/.../bra...4068.raw | not available | raw | Unknown | Unknown | 0 | | C:/.../bra...4069.raw | not available | raw | Unknown | Unknown | 0 | | C:/.../bra...4070.raw | not available | raw | Unknown | Unknown | 0 | | C:/.../bra...4071.raw | not available | raw | Unknown | Unknown | 0 | | C:/.../bra...4072.raw | not available | raw | Unknown | Unknown | 0 | | C:/.../bra...4073.raw | not available | raw | Unknown | Unknown | 0 | | C:/.../bra...4074.raw | not available | raw | Unknown | Unknown | 0 | | C:/.../bra...4075.raw | not available | raw | Unknown | Unknown | 0 | | C:/.../bra...4076.raw | not available | raw | Unknown | Unknown | 0 | | C:/.../bra...4077.raw | not available | raw | Unknown | Unknown | 0 | | C:/.../bra...4078.raw | not available | raw | Unknown | Unknown | 0 | | C:/.../bra...4079.raw | not available | raw | Unknown | Unknown | 0 | | C:/.../bra...4080.raw | not available | raw | Unknown | Unknown | 0 | | C:/.../bra...4081.raw | not available | raw | Unknown | Unknown | 0 | | C:/.../bra...4082.raw | not available | raw | Unknown | Unknown | 0 | | C:/.../bra...4083.raw | not available | raw | Unknown | Unknown | 0 | | C:/.../bra...4084.raw | not available | raw | Unknown | Unknown | 0 | | C:/.../bra...4085.raw | not available | raw | Unknown | Unknown | 0 | | C:/.../bra...4086.raw | not available | raw | Unknown | Unknown | 0 | | C:/.../bra...4087.raw | not available | raw | Unknown | Unknown | 0 | | C:/.../bra...4088.raw | not available | raw | Unknown | Unknown | 0 | | C:/.../bra...4089.raw | not available | raw | Unknown | Unknown | 0 | | C:/.../bra...4090.raw | not available | raw | Unknown | Unknown | 0 | | C:/.../bra...4091.raw | not available | raw | Unknown | Unknown | 0 | | C:/.../bra...4092.raw | not available | raw | Unknown | Unknown | 0 | | C:/.../bra...4093.raw | not available | raw | Unknown | Unknown | 0 | | C:/.../bra...4094.raw | not available | raw | Unknown | Unknown | 0 | | C:/.../bra...4095.raw | not available | raw | Unknown | Unknown | 0 | | C:/.../bra...4096.raw | not available | raw | Unknown | Unknown | 0 | | C:/.../bra...4097.raw | not available | raw | Unknown | Unknown | 0 | | C:/.../bra...4098.raw | not available | raw | Unknown | Unknown | 0 | | C:/.../bra...4099.raw | not available | raw | Unknown | Unknown | 0 | | C:/.../bra...4100.raw | not available | raw | Unknown | Unknown | 0 | | C:/.../bra...4101.raw | not available | raw | Unknown | Unknown | 0 | | C:/.../bra...4102.raw | not available | raw | Unknown | Unknown | 0 | | C:/.../bra...4103.raw | not available | raw | Unknown | Unknown | 0 | | C:/.../bra...4104.raw | not available | raw | Unknown | Unknown | 0 | | C:/.../bra...4105.raw | not available | raw | Unknown | Unknown | 0 | | C:/.../bra...4106.raw | not available | raw | Unknown | Unknown | 0 | | C:/.../bra...4107.raw | not available | raw | Unknown | Unknown | 0 | | C:/.../bra...4108.raw | not available | raw | Unknown | Unknown | 0 | | C:/.../bra...4109.raw | not available | raw | Unknown | Unknown | 0 | | C:/.../bra...4110.raw | not available | raw | Unknown | Unknown | 0 | | C:/.../bra...4111.raw | not available | raw | Unknown | Unknown | 0 | | C:/.../bra...4112.raw | not available | raw | Unknown | Unknown | 0 | | C:/.../bra...4113.raw | not available | raw | Unknown | Unknown | 0 | | C:/.../bra...4114.raw | not available | raw | Unknown | Unknown | 0 | | C:/.../bra...4115.raw | not available | raw | Unknown | Unknown | 0 | | C:/.../bra...4116.raw | not available | raw | Unknown | Unknown | 0 | | C:/.../bra...4117.raw | not available | raw | Unknown | Unknown | 0 | | C:/.../bra...4118.raw | not available | raw | Unknown | Unknown | 0 | | C:/.../bra...4119.raw | not available | raw | Unknown | Unknown | 0 | | C:/.../bra...4120.raw | not available | raw | Unknown | Unknown | 0 | | C:/.../bra...4121.raw | not available | raw | Unknown | Unknown | 0 | | C:/.../bra...4122.raw | not available | raw | Unknown | Unknown | 0 | | C:/.../bra...4123.raw | not available | raw | Unknown | Unknown | 0 | | C:/.../bra...4124.raw | not available | raw | Unknown | Unknown | 0 | | C:/.../bra...4125.raw | not available | raw | Unknown | Unknown | 0 | | C:/.../bra...4126.raw | not available | raw | Unknown | Unknown | 0 | | C:/.../bra...4127.raw | not available | raw | Unknown | Unknown | 0 | | C:/.../bra...4128.raw | not available | raw | Unknown | Unknown | 0 | | C:/.../bra...4129.raw | not available | raw | Unknown | Unknown | 0 | | C:/.../bra...4130.raw | not available | raw | Unknown | Unknown | 0 | | C:/.../bra...4131.raw | not available | raw | Unknown | Unknown | 0 | | C:/.../bra...4132.raw | not available | raw | Unknown | Unknown | 0 | | C:/.../bra...4133.raw | not available | raw | Unknown | Unknown | 0 | | C:/.../bra...4134.raw | not available | raw | Unknown | Unknown | 0 | | C:/.../bra...4135.raw | not available | raw | Unknown | Unknown | 0 | | C:/.../bra...4136.raw | not available | raw | Unknown | Unknown | 0 | | C:/.../bra...4137.raw | not available | raw | Unknown | Unknown | 0 | | C:/.../bra...4138.raw | not available | raw | Unknown | Unknown | 0 | | C:/.../bra...4139.raw | not available | raw | Unknown | Unknown | 0 | | C:/.../bra...4140.raw | not available | raw | Unknown | Unknown | 0 | | C:/.../bra...4141.raw | not available | raw | Unknown | Unknown | 0 | | C:/.../bra...4142.raw | not available | raw | Unknown | Unknown | 0 | | C:/.../bra...4143.raw | not available | raw | Unknown | Unknown | 0 | | C:/.../bra...4144.raw | not available | raw | Unknown | Unknown | 0 | | C:/.../bra...4145.raw | not available | raw | Unknown | Unknown | 0 | | C:/.../bra...4146.raw | not available | raw | Unknown | Unknown | 0 | | C:/.../bra...4147.raw | not available | raw | Unknown | Unknown | 0 | | C:/.../bra...4148.raw | not available | raw | Unknown | Unknown | 0 | | C:/.../bra...4149.raw | not available | raw | Unknown | Unknown | 0 | | C:/.../bra...4150.raw | not available | raw | Unknown | Unknown | 0 | | C:/.../bra...4151.raw | not available | raw | Unknown | Unknown | 0 | | C:/.../bra...4152.raw | not available | raw | Unknown | Unknown | 0 | | C:/.../bra...4153.raw | not available | raw | Unknown | Unknown | 0 | | C:/.../bra...4154.raw | not available | raw | Unknown | Unknown | 0 | | C:/.../bra...4155.raw | not available | raw | Unknown | Unknown | 0 | | C:/.../bra...4156.raw | not available | raw | Unknown | Unknown | 0 | | C:/.../bra...4157.raw | not available | raw | Unknown | Unknown | 0 | | C:/.../bra...4158.raw | not available | raw | Unknown | Unknown | 0 | | C:/.../bra...4159.raw | not available | raw | Unknown | Unknown | 0 | | C:/.../bra...4160.raw | not available | raw | Unknown | Unknown | 0 | | C:/.../bra...4161.raw | not available | raw | Unknown | Unknown | 0 | | C:/.../bra...4162.raw | not available | raw | Unknown | Unknown | 0 | | C:/.../bra...4163.raw | not available | raw | Unknown | Unknown | 0 | | C:/.../bra...4164.raw | not available | raw | Unknown | Unknown | 0 | | C:/.../bra...4165.raw | not available | raw | Unknown | Unknown | 0 | | C:/.../bra...4166.raw | not available | raw | Unknown | Unknown | 0 | | C:/.../bra...4167.raw | not available | raw | Unknown | Unknown | 0 | | C:/.../bra...4168.raw | not available | raw | Unknown | Unknown | 0 | | C:/.../bra...4169.raw | not available | raw | Unknown | Unknown | 0 | | C:/.../bra...4170.raw | not available | raw | Unknown | Unknown | 0 | | C:/.../bra...4171.raw | not available | raw | Unknown | Unknown | 0 | | C:/.../bra...4172.raw | not available | raw | Unknown | Unknown | 0 | | C:/.../bra...4173.raw | not available | raw | Unknown | Unknown | 0 | | C:/.../bra...4174.raw | not available | raw | Unknown | Unknown | 0 | | C:/.../bra...4175.raw | not available | raw | Unknown | Unknown | 0 | | C:/.../bra...4176.raw | not available | raw | Unknown | Unknown | 0 | | C:/.../bra...4177.raw | not available | raw | Unknown | Unknown | 0 | | C:/.../bra...4178.raw | not available | raw | Unknown | Unknown | 0 | | C:/.../bra...4179.raw | not available | raw | Unknown | Unknown | 0 | | C:/.../bra...4180.raw | not available | raw | Unknown | Unknown | 0 | | C:/.../bra...4181.raw | not available | raw | Unknown | Unknown | 0 | | C:/.../bra...4182.raw | not available | raw | Unknown | Unknown | 0 | | C:/.../bra...4183.raw | not available | raw | Unknown | Unknown | 0 | | C:/.../bra...4184.raw | not available | raw | Unknown | Unknown | 0 | | C:/.../bra...4185.raw | not available | raw | Unknown | Unknown | 0 | | C:/.../bra...4186.raw | not available | raw | Unknown | Unknown | 0 | | C:/.../bra...4187.raw | not available | raw | Unknown | Unknown | 0 | | C:/.../bra...4188.raw | not available | raw | Unknown | Unknown | 0 | | C:/.../bra...4189.raw | not available | raw | Unknown | Unknown | 0 | | C:/.../bra...4190.raw | not available | raw | Unknown | Unknown | 0 | | C:/.../bra...4191.raw | not available | raw | Unknown | Unknown | 0 | | C:/.../bra...4192.raw | not available | raw | Unknown | Unknown | 0 | | C:/.../bra...4193.raw | not available | raw | Unknown | Unknown | 0 | | C:/.../bra...4194.raw | not available | raw | Unknown | Unknown | 0 | | C:/.../bra...4195.raw | not available | raw | Unknown | Unknown | 0 | | C:/.../bra...4196.raw | not available | raw | Unknown | Unknown | 0 | | C:/.../bra...4197.raw | not available | raw | Unknown | Unknown | 0 | | C:/.../bra...4198.raw | not available | raw | Unknown | Unknown | 0 | | C:/.../bra...4199.raw | not available | raw | Unknown | Unknown | 0 | | C:/.../bra...4200.raw | not available | raw | Unknown | Unknown | 0 | | C:/.../bra...4201.raw | not available | raw | Unknown | Unknown | 0 | | C:/.../bra...4202.raw | not available | raw | Unknown | Unknown | 0 | | C:/.../bra...4203.raw | not available | raw | Unknown | Unknown | 0 | | C:/.../bra...4204.raw | not available | raw | Unknown | Unknown | 0 | | C:/.../bra...4205.raw | not available | raw | Unknown | Unknown | 0 | | C:/.../bra...4206.raw | not available | raw | Unknown | Unknown | 0 | | C:/.../bra...4207.raw | not available | raw | Unknown | Unknown | 0 | | C:/.../bra...4208.raw | not available | raw | Unknown | Unknown | 0 | | C:/.../bra...4209.raw | not available | raw | Unknown | Unknown | 0 | | C:/.../bra...4210.raw | not available | raw | Unknown | Unknown | 0 | | C:/.../bra...4211.raw | not available | raw | Unknown | Unknown | 0 | | C:/.../bra...4212.raw | not available | raw | Unknown | Unknown | 0 | | C:/.../bra...4213.raw | not available | raw | Unknown | Unknown | 0 | | C:/.../bra...4214.raw | not available | raw | Unknown | Unknown | 0 | | C:/.../bra...4215.raw | not available | raw | Unknown | Unknown | 0 | | C:/.../bra...4216.raw | not available | raw | Unknown | Unknown | 0 | | C:/.../bra...4217.raw | not available | raw | Unknown | Unknown | 0 | | C:/.../bra...4218.raw | not available | raw | Unknown | Unknown | 0 | | C:/.../bra...4219.raw | not available | raw | Unknown | Unknown | 0 | | C:/.../bra...4220.raw | not available | raw | Unknown | Unknown | 0 | | C:/.../bra...4221.raw | not available | raw | Unknown | Unknown | 0 | | C:/.../bra...4222.raw | not available | raw | Unknown | Unknown | 0 | | C:/.../bra...4223.raw | not available | raw | Unknown | Unknown | 0 | | C:/.../bra...4224.raw | not available | raw | Unknown | Unknown | 0 | | C:/.../bra...4225.raw | not available | raw | Unknown | Unknown | 0 | | C:/.../bra...4226.raw | not available | raw | Unknown | Unknown | 0 | | C:/.../bra...4227.raw | not available | raw | Unknown | Unknown | 0 | | C:/.../bra...4228.raw | not available | raw | Unknown | Unknown | 0 | | C:/.../bra...4229.raw | not available | raw | Unknown | Unknown | 0 | | C:/.../bra...4230.raw | not available | raw | Unknown | Unknown | 0 | | C:/.../bra...4231.raw | not available | raw | Unknown | Unknown | 0 | | C:/.../bra...4232.raw | not available | raw | Unknown | Unknown | 0 | | C:/.../bra...4233.raw | not available | raw | Unknown | Unknown | 0 | | C:/.../bra...4234.raw | not available | raw | Unknown | Unknown | 0 | | C:/.../bra...4235.raw | not available | raw | Unknown | Unknown | 0 | | C:/.../bra...4236.raw | not available | raw | Unknown | Unknown | 0 | | C:/.../bra...4237.raw | not available | raw | Unknown | Unknown | 0 | | C:/.../bra...4238.raw | not available | raw | Unknown | Unknown | 0 | | C:/.../bra...4239.raw | not available | raw | Unknown | Unknown | 0 | | C:/.../bra...4240.raw | not available | raw | Unknown | Unknown | 0 | | C:/.../bra...4241.raw | not available | raw | Unknown | Unknown | 0 | | C:/.../bra...4242.raw | not available | raw | Unknown | Unknown | 0 | | C:/.../bra...4243.raw | not available | raw | Unknown | Unknown | 0 | | C:/.../bra...4244.raw | not available | raw | Unknown | Unknown | 0 | | C:/.../bra...4245.raw | not available | raw | Unknown | Unknown | 0 | | C:/.../bra...4246.raw | not available | raw | Unknown | Unknown | 0 | | C:/.../bra...4247.raw | not available | raw | Unknown | Unknown | 0 | | C:/.../bra...4248.raw | not available | raw | Unknown | Unknown | 0 | | C:/.../bra...4249.raw | not available | raw | Unknown | Unknown | 0 | | C:/.../bra...4250.raw | not available | raw | Unknown | Unknown | 0 | | C:/.../bra...4251.raw | not available | raw | Unknown | Unknown | 0 | | C:/.../bra...4252.raw | not available | raw | Unknown | Unknown | 0 | | C:/.../bra...4253.raw | not available | raw | Unknown | Unknown | 0 | | C:/.../bra...4254.raw | not available | raw | Unknown | Unknown | 0 | | C:/.../bra...4255.raw | not available | raw | Unknown | Unknown | 0 | | C:/.../bra...4256.raw | not available | raw | Unknown | Unknown | 0 | | C:/.../bra...4257.raw | not available | raw | Unknown | Unknown | 0 | | C:/.../bra...4258.raw | not available | raw | Unknown | Unknown | 0 | | C:/.../bra...4259.raw | not available | raw | Unknown | Unknown | 0 | | C:/.../bra...4260.raw | not available | raw | Unknown | Unknown | 0 | | C:/.../bra...4261.raw | not available | raw | Unknown | Unknown | 0 | | C:/.../bra...4262.raw | not available | raw | Unknown | Unknown | 0 | | C:/.../bra...4263.raw | not available | raw | Unknown | Unknown | 0 | | C:/.../bra...4264.raw | not available | raw | Unknown | Unknown | 0 | | C:/.../bra...4265.raw | not available | raw | Unknown | Unknown | 0 | | C:/.../bra...4266.raw | not available | raw | Unknown | Unknown | 0 | | C:/.../bra...4267.raw | not available | raw | Unknown | Unknown | 0 | | C:/.../bra...4268.raw | not available | raw | Unknown | Unknown | 0 | | C:/.../bra...4269.raw | not available | raw | Unknown | Unknown | 0 | | C:/.../bra...4270.raw | not available | raw | Unknown | Unknown | 0 | | C:/.../bra...4271.raw | not available | raw | Unknown | Unknown | 0 | | C:/.../bra...4272.raw | not available | raw | Unknown | Unknown | 0 | | C:/.../bra...4273.raw | not available | raw | Unknown | Unknown | 0 | | C:/.../bra...4274.raw | not available | raw | Unknown | Unknown | 0 | | C:/.../bra...4275.raw | not available | raw | Unknown | Unknown | 0 | | C:/.../bra...4276.raw | not available | raw | Unknown | Unknown | 0 | | C:/.../bra...4277.raw | not available | raw | Unknown | Unknown | 0 | | C:/.../bra...4278.raw | not available | raw | Unknown | Unknown | 0 | | C:/.../bra...4279.raw | not available | raw | Unknown | Unknown | 0 | | C:/.../bra...4280.raw | not available | raw | Unknown | Unknown | 0 | | C:/.../bra...4281.raw | not available | raw | Unknown | Unknown | 0 | | C:/.../bra...4282.raw | not available | raw | Unknown | Unknown | 0 | | C:/.../bra...4283.raw | not available | raw | Unknown | Unknown | 0 | | C:/.../bra...4284.raw | not available | raw | Unknown | Unknown | 0 | | C:/.../bra...4285.raw | not available | raw | Unknown | Unknown | 0 | | C:/.../bra...4286.raw | not available | raw | Unknown | Unknown | 0 | | C:/.../bra...4287.raw | not available | raw | Unknown | Unknown | 0 | | C:/.../bra...4288.raw | not available | raw | Unknown | Unknown | 0 | | C:/.../bra...4289.raw | not available | raw | Unknown | Unknown | 0 | | C:/.../bra...4290.raw | not available | raw | Unknown | Unknown | 0 | | C:/.../bra...4291.raw | not available | raw | Unknown | Unknown | 0 | | C:/.../bra...4292.raw | not available | raw | Unknown | Unknown | 0 | | C:/.../bra...4293.raw | not available | raw | Unknown | Unknown | 0 | | C:/.../bra...4294.raw | not available | raw | Unknown | Unknown | 0 | | C:/.../bra...4295.raw | not available | raw | Unknown | Unknown | 0 | | C:/.../bra...4296.raw | not available | raw | Unknown | Unknown | 0 | | C:/.../bra...4297.raw | not available | raw | Unknown | Unknown | 0 | | C:/.../bra...4298.raw | not available | raw | Unknown | Unknown | 0 | | C:/.../bra...4299.raw | not available | raw | Unknown | Unknown | 0 | | C:/.../bra...4300.raw | not available | raw | Unknown | Unknown | 0 | | C:/.../bra...4301.raw | not available | raw | Unknown | Unknown | 0 | | C:/.../bra...4302.raw | not available | raw | Unknown | Unknown | 0 | | C:/.../bra...4303.raw | not available | raw | Unknown | Unknown | 0 | | C:/.../bra...4304.raw | not available | raw | Unknown | Unknown | 0 | | C:/.../bra...4305.raw | not available | raw | Unknown | Unknown | 0 | | C:/.../bra...4306.raw | not available | raw | Unknown | Unknown | 0 | | C:/.../bra...4307.raw | not available | raw | Unknown | Unknown | 0 | | C:/.../bra...4308.raw | not available | raw | Unknown | Unknown | 0 | | C:/.../bra...4309.raw | not available | raw | Unknown | Unknown | 0 | | C:/.../bra...4310.raw | not available | raw | Unknown | Unknown | 0 | | C:/.../bra...4311.raw | not available | raw | Unknown | Unknown | 0 | | C:/.../bra...4312.raw | not available | raw | Unknown | Unknown | 0 | | C:/.../bra...4313.raw | not available | raw | Unknown | Unknown | 0 | | C:/.../bra...4314.raw | not available | raw | Unknown | Unknown | 0 | | C:/.../bra...4315.raw | not available | raw | Unknown | Unknown | 0 | | C:/.../bra...4316.raw | not available | raw | Unknown | Unknown | 0 | | C:/.../bra...4317.raw | not available | raw | Unknown | Unknown | 0 | | C:/.../bra...4318.raw | not available | raw | Unknown | Unknown | 0 | | C:/.../bra...4319.raw | not available | raw | Unknown | Unknown | 0 | | C:/.../bra...4320.raw | not available | raw | Unknown | Unknown | 0 | | C:/.../bra...4321.raw | not available | raw | Unknown | Unknown | 0 | | C:/.../bra...4322.raw | not available | raw | Unknown | Unknown | 0 | | C:/.../bra...4323.raw | not available | raw | Unknown | Unknown | 0 | | C:/.../bra...4324.raw | not available | raw | Unknown | Unknown | 0 | | C:/.../bra...4325.raw | not available | raw | Unknown | Unknown | 0 | | C:/.../bra...4326.raw | not available | raw | Unknown | Unknown | 0 | | C:/.../bra...4327.raw | not available | raw | Unknown | Unknown | 0 | | C:/.../bra...4328.raw | not available | raw | Unknown | Unknown | 0 | | C:/.../bra...4329.raw | not available | raw | Unknown | Unknown | 0 | | C:/.../bra...4330.raw | not available | raw | Unknown | Unknown | 0 | | C:/.../bra...4331.raw | not available | raw | Unknown | Unknown | 0 | | C:/.../bra...4332.raw | not available | raw | Unknown | Unknown | 0 | | C:/.../bra...4333.raw | not available | raw | Unknown | Unknown | 0 | | C:/.../bra...4334.raw | not available | raw | Unknown | Unknown | 0 | | C:/.../bra...4335.raw | not available | raw | Unknown | Unknown | 0 | | C:/.../bra...4336.raw | not available | raw | Unknown | Unknown | 0 | | C:/.../bra...4337.raw | not available | raw | Unknown | Unknown | 0 | | C:/.../bra...4338.raw | not available | raw | Unknown | Unknown | 0 | | C:/.../bra...4339.raw | not available | raw | Unknown | Unknown | 0 | | C:/.../bra...4340.raw | not available | raw | Unknown | Unknown | 0 | | C:/.../bra...4341.raw | not available | raw | Unknown | Unknown | 0 | | C:/.../bra...4342.raw | not available | raw | Unknown | Unknown | 0 | | C:/.../bra...4343.raw | not available | raw | Unknown | Unknown | 0 | | C:/.../bra...4344.raw | not available | raw | Unknown | Unknown | 0 | | C:/.../bra...4345.raw | not available | raw | Unknown | Unknown | 0 | | C:/.../bra...4346.raw | not available | raw | Unknown | Unknown | 0 | | C:/.../bra...4347.raw | not available | raw | Unknown | Unknown | 0 | | C:/.../bra...4348.raw | not available | raw | Unknown | Unknown | 0 | | C:/.../bra...4349.raw | not available | raw | Unknown | Unknown | 0 | | C:/.../bra...4350.raw | not available | raw | Unknown | Unknown | 0 | | C:/.../bra...4351.raw | not available | raw | Unknown | Unknown | 0 | | C:/.../bra...4352.raw | not available | raw | Unknown | Unknown | 0 | | C:/.../bra...4353.raw | not available | raw | Unknown | Unknown | 0 | | C:/.../bra...4354.raw | not available | raw | Unknown | Unknown | 0 | | C:/.../bra...4355.raw | not available | raw | Unknown | Unknown | 0 | | C:/.../bra...4356.raw | not available | raw | Unknown | Unknown | 0 | | C:/.../bra...4357.raw | not available | raw | Unknown | Unknown | 0 | | C:/.../bra...4358.raw | not available | raw | Unknown | Unknown | 0 | | C:/.../bra...4359.raw | not available | raw | Unknown | Unknown | 0 | | C:/.../bra...4360.raw | not available | raw | Unknown | Unknown | 0 | | C:/.../bra...4361.raw | not available | raw | Unknown | Unknown | 0 | | C:/.../bra...4362.raw | not available | raw | Unknown | Unknown | 0 | | C:/.../bra...4363.raw | not available | raw | Unknown | Unknown | 0 | | C:/.../bra...4364.raw | not available | raw | Unknown | Unknown | 0 | | C:/.../bra...4365.raw | not available | raw | Unknown | Unknown | 0 | | C:/.../bra...4366.raw | not available | raw | Unknown | Unknown | 0 | | C:/.../bra...4367.raw | not available | raw | Unknown | Unknown | 0 | | C:/.../bra...4368.raw | not available | raw | Unknown | Unknown | 0 | | C:/.../bra...4369.raw | not available | raw | Unknown | Unknown | 0 | | C:/.../bra...4370.raw | not available | raw | Unknown | Unknown | 0 | | C:/.../bra...4371.raw | not available | raw | Unknown | Unknown | 0 | | C:/.../bra...4372.raw | not available | raw | Unknown | Unknown | 0 | | C:/.../bra...4373.raw | not available | raw | Unknown | Unknown | 0 | | C:/.../bra...4374.raw | not available | raw | Unknown | Unknown | 0 | | C:/.../bra...4375.raw | not available | raw | Unknown | Unknown | 0 | | C:/.../bra...4376.raw | not available | raw | Unknown | Unknown | 0 | | C:/.../bra...4377.raw | not available | raw | Unknown | Unknown | 0 | | C:/.../bra...4378.raw | not available | raw | Unknown | Unknown | 0 | | C:/.../bra...4379.raw | not available | raw | Unknown | Unknown | 0 | | C:/.../bra...4380.raw | not available | raw | Unknown | Unknown | 0 | | C:/.../bra...4381.raw | not available | raw | Unknown | Unknown | 0 | | C:/.../bra...4382.raw | not available | raw | Unknown | Unknown | 0 | | C:/.../bra...4383.raw | not available | raw | Unknown | Unknown | 0 | | C:/.../bra...4384.raw | not available | raw | Unknown | Unknown | 0 | | C:/.../bra...4385.raw | not available | raw | Unknown | Unknown | 0 | | C:/.../bra...4386.raw | not available | raw | Unknown | Unknown | 0 | | C:/.../bra...4387.raw | not available | raw | Unknown | Unknown | 0 | | C:/.../bra...4388.raw | not available | raw | Unknown | Unknown | 0 | | C:/.../bra...4389.raw | not available | raw | Unknown | Unknown | 0 | | C:/.../bra...4390.raw | not available | raw | Unknown | Unknown | 0 | | C:/.../bra...4391.raw | not available | raw | Unknown | Unknown | 0 | | C:/.../bra...4392.raw | not available | raw | Unknown | Unknown | 0 | | C:/.../bra...4393.raw | not available | raw | Unknown | Unknown | 0 | | C:/.../bra...4394.raw | not available | raw | Unknown | Unknown | 0 | | C:/.../bra...4395.raw | not available | raw | Unknown | Unknown | 0 | | C:/.../bra...4396.raw | not available | raw | Unknown | Unknown | 0 | | C:/.../bra...4397.raw | not available | raw | Unknown | Unknown | 0 | | C:/.../bra...4398.raw | not available | raw | Unknown | Unknown | 0 | | C:/.../bra...4399.raw | not available | raw | Unknown | Unknown | 0 | | C:/.../bra...4400.raw | not available | raw | Unknown | Unknown | 0 | | C:/.../bra...4401.raw | not available | raw | Unknown | Unknown | 0 | | C:/.../bra...4402.raw | not available | raw | Unknown | Unknown | 0 | | C:/.../bra...4403.raw | not available | raw | Unknown | Unknown | 0 | | C:/.../bra...4404.raw | not available | raw | Unknown | Unknown | 0 | | C:/.../bra...4405.raw | not available | raw | Unknown | Unknown | 0 | | C:/.../bra...4406.raw | not available | raw | Unknown | Unknown | 0 | | C:/.../bra...4407.raw | not available | raw | Unknown | Unknown | 0 | | C:/.../bra...4408.raw | not available | raw | Unknown | Unknown | 0 | | C:/.../bra...4409.raw | not available | raw | Unknown | Unknown | 0 | | C:/.../bra...4410.raw | not available | raw | Unknown | Unknown | 0 | | C:/.../bra...4411.raw | not available | raw | Unknown | Unknown | 0 | | C:/.../bra...4412.raw | not available | raw | Unknown | Unknown | 0 | | C:/.../bra...4413.raw | not available | raw | Unknown | Unknown | 0 | | C:/.../bra...4414.raw | not available | raw | Unknown | Unknown | 0 | | C:/.../bra...4415.raw | not available | raw | Unknown | Unknown | 0 | | C:/.../bra...4416.raw | not available | raw | Unknown | Unknown | 0 | | C:/.../bra...4417.raw | not available | raw | Unknown | Unknown | 0 | | C:/.../bra...4418.raw | not available | raw | Unknown | Unknown | 0 | | C:/.../bra...4419.raw | not available | raw | Unknown | Unknown | 0 | | C:/.../bra...4420.raw | not available | raw | Unknown | Unknown | 0 | | C:/.../bra...4421.raw | not available | raw | Unknown | Unknown | 0 | | C:/.../bra...4422.raw | not available | raw | Unknown | Unknown | 0 | | C:/.../bra...4423.raw | not available | raw | Unknown | Unknown | 0 | | C:/.../bra...4424.raw | not available | raw | Unknown | Unknown | 0 | | C:/.../bra...4425.raw | not available | raw | Unknown | Unknown | 0 | | C:/.../bra...4426.raw | not available | raw | Unknown | Unknown | 0 | | C:/.../bra...4427.raw | not available | raw | Unknown | Unknown | 0 | | C:/.../bra...4428.raw | not available | raw | Unknown | Unknown | 0 | | C:/.../bra...4429.raw | not available | raw | Unknown | Unknown | 0 | | C:/.../bra...4430.raw | not available | raw | Unknown | Unknown | 0 | | C:/.../bra...4431.raw | not available | raw | Unknown | Unknown | 0 | | C:/.../bra...4432.raw | not available | raw | Unknown | Unknown | 0 | | C:/.../bra...4433.raw | not available | raw | Unknown | Unknown | 0 | | C:/.../bra...4434.raw | not available | raw | Unknown | Unknown | 0 | | C:/.../bra...4435.raw | not available | raw | Unknown | Unknown | 0 | | C:/.../bra...4436.raw | not available | raw | Unknown | Unknown | 0 | | C:/.../bra...4437.raw | not available | raw | Unknown | Unknown | 0 | | C:/.../bra...4438.raw | not available | raw | Unknown | Unknown | 0 | | C:/.../bra...4439.raw | not available | raw | Unknown | Unknown | 0 | | C:/.../bra...4440.raw | not available | raw | Unknown | Unknown | 0 | | C:/.../bra...4441.raw | not available | raw | Unknown | Unknown | 0 | | C:/.../bra...4442.raw | not available | raw | Unknown | Unknown | 0 | | C:/.../bra...4443.raw | not available | raw | Unknown | Unknown | 0 | | C:/.../bra...4444.raw | not available | raw | Unknown | Unknown | 0 | | C:/.../bra...4445.raw | not available | raw | Unknown | Unknown | 0 | | C:/.../bra...4446.raw | not available | raw | Unknown | Unknown | 0 | | C:/.../bra...4447.raw | not available | raw | Unknown | Unknown | 0 | | C:/.../bra...4448.raw | not available | raw | Unknown | Unknown | 0 | | C:/.../bra...4449.raw | not available | raw | Unknown | Unknown | 0 | | C:/.../bra...4450.raw | not available | raw | Unknown | Unknown | 0 | | C:/.../bra...4451.raw | not available | raw | Unknown | Unknown | 0 | | C:/.../bra...4452.raw | not available | raw | Unknown | Unknown | 0 | | C:/.../bra...4453.raw | not available | raw | Unknown | Unknown | 0 | | C:/.../bra...4454.raw | not available | raw | Unknown | Unknown | 0 | | C:/.../bra...4455.raw | not available | raw | Unknown | Unknown | 0 | | C:/.../bra...4456.raw | not available | raw | Unknown | Unknown | 0 | | C:/.../bra...4457.raw | not available | raw | Unknown | Unknown | 0 | | C:/.../bra...4458.raw | not available | raw | Unknown | Unknown | 0 | | C:/.../bra...4459.raw | not available | raw | Unknown | Unknown | 0 | | C:/.../bra...4460.raw | not available | raw | Unknown | Unknown | 0 | | C:/.../bra...4461.raw | not available | raw | Unknown | Unknown | 0 | | C:/.../bra...4462.raw | not available | raw | Unknown | Unknown | 0 | | C:/.../bra...4463.raw | not available | raw | Unknown | Unknown | 0 | | C:/.../bra...4464.raw | not available | raw | Unknown | Unknown | 0 | | C:/.../bra...4465.raw | not available | raw | Unknown | Unknown | 0 | | C:/.../bra...4466.raw | not available | raw | Unknown | Unknown | 0 | | C:/.../bra...4467.raw | not available | raw | Unknown | Unknown | 0 | | C:/.../bra...4468.raw | not available | raw | Unknown | Unknown | 0 | | C:/.../bra...4469.raw | not available | raw | Unknown | Unknown | 0 | | C:/.../bra...4470.raw | not available | raw | Unknown | Unknown | 0 | | C:/.../bra...4471.raw | not available | raw | Unknown | Unknown | 0 | | C:/.../bra...4472.raw | not available | raw | Unknown | Unknown | 0 | | C:/.../bra...4473.raw | not available | raw | Unknown | Unknown | 0 | | C:/.../bra...4474.raw | not available | raw | Unknown | Unknown | 0 | | C:/.../bra...4475.raw | not available | raw | Unknown | Unknown | 0 | | C:/.../bra...4476.raw | not available | raw | Unknown | Unknown | 0 | | C:/.../bra...4477.raw | not available | raw | Unknown | Unknown | 0 | | C:/.../bra...4478.raw | not available | raw | Unknown | Unknown | 0 | | C:/.../bra...4479.raw | not available | raw | Unknown | Unknown | 0 | | C:/.../bra...4480.raw | not available | raw | Unknown | Unknown | 0 | | C:/.../bra...4481.raw | not available | raw | Unknown | Unknown | 0 | | C:/.../bra...4482.raw | not available | raw | Unknown | Unknown | 0 | | C:/.../bra...4483.raw | not available | raw | Unknown | Unknown | 0 | | C:/.../bra...4484.raw | not available | raw | Unknown | Unknown | 0 | | C:/.../bra...4485.raw | not available | raw | Unknown | Unknown | 0 | | C:/.../bra...4486.raw | not available | raw | Unknown | Unknown | 0 | | C:/.../bra...4487.raw | not available | raw | Unknown | Unknown | 0 | | C:/.../bra...4488.raw | not available | raw | Unknown | Unknown | 0 | | C:/.../bra...4489.raw | not available | raw | Unknown | Unknown | 0 | | C:/.../bra...4490.raw | not available | raw | Unknown | Unknown | 0 | | C:/.../bra...4491.raw | not available | raw | Unknown | Unknown | 0 | | C:/.../bra...4492.raw | not available | raw | Unknown | Unknown | 0 | | C:/.../bra...4493.raw | not available | raw | Unknown | Unknown | 0 | | C:/.../bra...4494.raw | not available | raw | Unknown | Unknown | 0 | | C:/.../bra...4495.raw | not available | raw | Unknown | Unknown | 0 | | C:/.../bra...4496.raw | not available | raw | Unknown | Unknown | 0 | | C:/.../bra...4497.raw | not available | raw | Unknown | Unknown | 0 | | C:/.../bra...4498.raw | not available | raw | Unknown | Unknown | 0 | | C:/.../bra...4499.raw | not available | raw | Unknown | Unknown | 0 | | C:/.../bra...4500.raw | not available | raw | Unknown | Unknown | 0 | | C:/.../bra...4501.raw | not available | raw | Unknown | Unknown | 0 | | C:/.../bra...4502.raw | not available | raw | Unknown | Unknown | 0 | | C:/.../bra...4503.raw | not available | raw | Unknown | Unknown | 0 | | C:/.../bra...4504.raw | not available | raw | Unknown | Unknown | 0 | | C:/.../bra...4505.raw | not available | raw | Unknown | Unknown | 0 | | C:/.../bra...4506.raw | not available | raw | Unknown | Unknown | 0 | | C:/.../bra...4507.raw | not available | raw | Unknown | Unknown | 0 | | C:/.../bra...4508.raw | not available | raw | Unknown | Unknown | 0 | | C:/.../bra...4509.raw | not available | raw | Unknown | Unknown | 0 | | C:/.../bra...4510.raw | not available | raw | Unknown | Unknown | 0 | | C:/.../bra...4511.raw | not available | raw | Unknown | Unknown | 0 | | C:/.../bra...4512.raw | not available | raw | Unknown | Unknown | 0 | | C:/.../bra...4513.raw | not available | raw | Unknown | Unknown | 0 | | C:/.../bra...4514.raw | not available | raw | Unknown | Unknown | 0 | | C:/.../bra...4515.raw | not available | raw | Unknown | Unknown | 0 | | C:/.../bra...4516.raw | not available | raw | Unknown | Unknown | 0 | | C:/.../bra...4517.raw | not available | raw | Unknown | Unknown | 0 | | C:/.../bra...4518.raw | not available | raw | Unknown | Unknown | 0 | | C:/.../bra...4519.raw | not available | raw | Unknown | Unknown | 0 | | C:/.../bra...4520.raw | not available | raw | Unknown | Unknown | 0 | | C:/.../bra...4521.raw | not available | raw | Unknown | Unknown | 0 | | C:/.../bra...4522.raw | not available | raw | Unknown | Unknown | 0 | | C:/.../bra...4523.raw | not available | raw | Unknown | Unknown | 0 | | C:/.../bra...4524.raw | not available | raw | Unknown | Unknown | 0 | | C:/.../bra...4525.raw | not available | raw | Unknown | Unknown | 0 | | C:/.../bra...4526.raw | not available | raw | Unknown | Unknown | 0 | | C:/.../bra...4527.raw | not available | raw | Unknown | Unknown | 0 | | C:/.../bra...4528.raw | not available | raw | Unknown | Unknown | 0 | | C:/.../bra...4529.raw | not available | raw | Unknown | Unknown | 0 | | C:/.../bra...4530.raw | not available | raw | Unknown | Unknown | 0 | | C:/.../bra...4531.raw | not available | raw | Unknown | Unknown | 0 | | C:/.../bra...4532.raw | not available | raw | Unknown | Unknown | 0 | | C:/.../bra...4533.raw | not available | raw | Unknown | Unknown | 0 | | C:/.../bra...4534.raw | not available | raw | Unknown | Unknown | 0 | | C:/.../bra...4535.raw | not available | raw | Unknown | Unknown | 0 | | C:/.../bra...4536.raw | not available | raw | Unknown | Unknown | 0 | | C:/.../bra...4537.raw | not available | raw | Unknown | Unknown | 0 | | C:/.../bra...4538.raw | not available | raw | Unknown | Unknown | 0 | | C:/.../bra...4539.raw | not available | raw | Unknown | Unknown | 0 | | C:/.../bra...4540.raw | not available | raw | Unknown | Unknown | 0 | | C:/.../bra...4541.raw | not available | raw | Unknown | Unknown | 0 | | C:/.../bra...4542.raw | not available | raw | Unknown | Unknown | 0 | | C:/.../bra...4543.raw | not available | raw | Unknown | Unknown | 0 | | C:/.../bra...4544.raw | not available | raw | Unknown | Unknown | 0 | | C:/.../bra...4545.raw | not available | raw | Unknown | Unknown | 0 | | C:/.../bra...4546.raw | not available | raw | Unknown | Unknown | 0 | | C:/.../bra...4547.raw | not available | raw | Unknown | Unknown | 0 | | C:/.../bra...4548.raw | not available | raw | Unknown | Unknown | 0 | | C:/.../bra...4549.raw | not available | raw | Unknown | Unknown | 0 | | C:/.../bra...4550.raw | not available | raw | Unknown | Unknown | 0 | | C:/.../bra...4551.raw | not available | raw | Unknown | Unknown | 0 | | C:/.../bra...4552.raw | not available | raw | Unknown | Unknown | 0 | | C:/.../bra...4553.raw | not available | raw | Unknown | Unknown | 0 | | C:/.../bra...4554.raw | not available | raw | Unknown | Unknown | 0 | | C:/.../bra...4555.raw | not available | raw | Unknown | Unknown | 0 | | C:/.../bra...4556.raw | not available | raw | Unknown | Unknown | 0 | | C:/.../bra...4557.raw | not available | raw | Unknown | Unknown | 0 | | C:/.../bra...4558.raw | not available | raw | Unknown | Unknown | 0 | | C:/.../bra...4559.raw | not available | raw | Unknown | Unknown | 0 | | C:/.../bra...4560.raw | not available | raw | Unknown | Unknown | 0 | | C:/.../bra...4561.raw | not available | raw | Unknown | Unknown | 0 | | C:/.../bra...4562.raw | not available | raw | Unknown | Unknown | 0 | | C:/.../bra...4563.raw | not available | raw | Unknown | Unknown | 0 | | C:/.../bra...4564.raw | not available | raw | Unknown | Unknown | 0 | | C:/.../bra...4565.raw | not available | raw | Unknown | Unknown | 0 | | C:/.../bra...4566.raw | not available | raw | Unknown | Unknown | 0 | | C:/.../bra...4567.raw | not available | raw | Unknown | Unknown | 0 | | C:/.../bra...4568.raw | not available | raw | Unknown | Unknown | 0 | | C:/.../bra...4569.raw | not available | raw | Unknown | Unknown | 0 | | C:/.../bra...4570.raw | not available | raw | Unknown | Unknown | 0 | | C:/.../bra...4571.raw | not available | raw | Unknown | Unknown | 0 | | C:/.../bra...4572.raw | not available | raw | Unknown | Unknown | 0 | | C:/.../bra...4573.raw | not available | raw | Unknown | Unknown | 0 | | C:/.../bra...4574.raw | not available | raw | Unknown | Unknown | 0 | | C:/.../bra...4575.raw | not available | raw | Unknown | Unknown | 0 | | C:/.../bra...4576.raw | not available | raw | Unknown | Unknown | 0 | | C:/.../bra...4577.raw | not available | raw | Unknown | Unknown | 0 | | C:/.../bra...4578.raw | not available | raw | Unknown | Unknown | 0 | | C:/.../bra...4579.raw | not available | raw | Unknown | Unknown | 0 | | C:/.../bra...4580.raw | not available | raw | Unknown | Unknown | 0 | | C:/.../bra...4581.raw | not available | raw | Unknown | Unknown | 0 | | C:/.../bra...4582.raw | not available | raw | Unknown | Unknown | 0 | | C:/.../bra...4583.raw | not available | raw | Unknown | Unknown | 0 | | C:/.../bra...4584.raw | not available | raw | Unknown | Unknown | 0 | | C:/.../bra...4585.raw | not available | raw | Unknown | Unknown | 0 | | C:/.../bra...4586.raw | not available | raw | Unknown | Unknown | 0 | | C:/.../bra...4587.raw | not available | raw | Unknown | Unknown | 0 | | C:/.../bra...4588.raw | not available | raw | Unknown | Unknown | 0 | | C:/.../bra...4589.raw | not available | raw | Unknown | Unknown | 0 | | C:/.../bra...4590.raw | not available | raw | Unknown | Unknown | 0 | | C:/.../bra...4591.raw | not available | raw | Unknown | Unknown | 0 | | C:/.../bra...4592.raw | not available | raw | Unknown | Unknown | 0 | | C:/.../bra...4593.raw | not available | raw | Unknown | Unknown | 0 | | C:/.../bra...4594.raw | not available | raw | Unknown | Unknown | 0 | | C:/.../bra...4595.raw | not available | raw | Unknown | Unknown | 0 | | C:/.../bra...4596.raw | not available | raw | Unknown | Unknown | 0 | | C:/.../bra...4597.raw | not available | raw | Unknown | Unknown | 0 | | C:/.../bra...4598.raw | not available | raw | Unknown | Unknown | 0 | | C:/.../bra...4599.raw | not available | raw | Unknown | Unknown | 0 | | C:/.../bra...4600.raw | not available | raw | Unknown | Unknown | 0 | | C:/.../bra...4601.raw | not available | raw | Unknown | Unknown | 0 | | C:/.../bra...4602.raw | not available | raw | Unknown | Unknown | 0 | | C:/.../bra...4603.raw | not available | raw | Unknown | Unknown | 0 | | C:/.../bra...4604.raw | not available | raw | Unknown | Unknown | 0 | | C:/.../bra...4605.raw | not available | raw | Unknown | Unknown | 0 | | C:/.../bra...4606.raw | not available | raw | Unknown | Unknown | 0 | | C:/.../bra...4607.raw | not available | raw | Unknown | Unknown | 0 | | C:/.../bra...4608.raw | not available | raw | Unknown | Unknown | 0 | | C:/.../bra...4609.raw | not available | raw | Unknown | Unknown | 0 | | C:/.../bra...4610.raw | not available | raw | Unknown | Unknown | 0 | | C:/.../bra...4611.raw | not available | raw | Unknown | Unknown | 0 | | C:/.../bra...4612.raw | not available | raw | Unknown | Unknown | 0 | | C:/.../bra...4613.raw | not available | raw | Unknown | Unknown | 0 | | C:/.../bra...4614.raw | not available | raw | Unknown | Unknown | 0 | | C:/.../bra...4615.raw | not available | raw | Unknown | Unknown | 0 | | C:/.../bra...4616.raw | not available | raw | Unknown | Unknown | 0 | | C:/.../bra...4617.raw | not available | raw | Unknown | Unknown | 0 | | C:/.../bra...4618.raw | not available | raw | Unknown | Unknown | 0 | | C:/.../bra...4619.raw | not available | raw | Unknown | Unknown | 0 | | C:/.../bra...4620.raw | not available | raw | Unknown | Unknown | 0 | | C:/.../bra...4621.raw | not available | raw | Unknown | Unknown | 0 | | C:/.../bra...4622.raw | not available | raw | Unknown | Unknown | 0 | | C:/.../bra...4623.raw | not available | raw | Unknown | Unknown | 0 | | C:/.../bra...4624.raw | not available | raw | Unknown | Unknown | 0 | | C:/.../bra...4625.raw | not available | raw | Unknown | Unknown | 0 | | C:/.../bra...4626.raw | not available | raw | Unknown | Unknown | 0 | | C:/.../bra...4627.raw | not available | raw | Unknown | Unknown | 0 | | C:/.../bra...4628.raw | not available | raw | Unknown | Unknown | 0 | | C:/.../bra...4629.raw | not available | raw | Unknown | Unknown | 0 | | C:/.../bra...4630.raw | not available | raw | Unknown | Unknown | 0 | | C:/.../bra...4631.raw | not available | raw | Unknown | Unknown | 0 | | C:/.../bra...4632.raw | not available | raw | Unknown | Unknown | 0 | | C:/.../bra...4633.raw | not available | raw | Unknown | Unknown | 0 | | C:/.../bra...4634.raw | not available | raw | Unknown | Unknown | 0 | | C:/.../bra...4635.raw | not available | raw | Unknown | Unknown | 0 | | C:/.../bra...4636.raw | not available | raw | Unknown | Unknown | 0 | | C:/.../bra...4637.raw | not available | raw | Unknown | Unknown | 0 | | C:/.../bra...4638.raw | not available | raw | Unknown | Unknown | 0 | | C:/.../bra...4639.raw | not available | raw | Unknown | Unknown | 0 | | C:/.../bra...4640.raw | not available | raw | Unknown | Unknown | 0 | | C:/.../bra...4641.raw | not available | raw | Unknown | Unknown | 0 | | C:/.../bra...4642.raw | not available | raw | Unknown | Unknown | 0 | | C:/.../bra...4643.raw | not available | raw | Unknown | Unknown | 0 | | C:/.../bra...4644.raw | not available | raw | Unknown | Unknown | 0 | | C:/.../bra...4645.raw | not available | raw | Unknown | Unknown | 0 | | C:/.../bra...4646.raw | not available | raw | Unknown | Unknown | 0 | | C:/.../bra...4647.raw | not available | raw | Unknown | Unknown | 0 | | C:/.../bra...4648.raw | not available | raw | Unknown | Unknown | 0 | | C:/.../bra...4649.raw | not available | raw | Unknown | Unknown | 0 | | C:/.../bra...4650.raw | not available | raw | Unknown | Unknown | 0 | | C:/.../bra...4651.raw | not available | raw | Unknown | Unknown | 0 | | C:/.../bra...4652.raw | not available | raw | Unknown | Unknown | 0 | | C:/.../bra...4653.raw | not available | raw | Unknown | Unknown | 0 | | C:/.../bra...4654.raw | not available | raw | Unknown | Unknown | 0 | | C:/.../bra...4655.raw | not available | raw | Unknown | Unknown | 0 | | C:/.../bra...4656.raw | not available | raw | Unknown | Unknown | 0 | | C:/.../bra...4657.raw | not available | raw | Unknown | Unknown | 0 | | C:/.../bra...4658.raw | not available | raw | Unknown | Unknown | 0 | | C:/.../bra...4659.raw | not available | raw | Unknown | Unknown | 0 | | C:/.../bra...4660.raw | not available | raw | Unknown | Unknown | 0 | | C:/.../bra...4661.raw | not available | raw | Unknown | Unknown | 0 | | C:/.../bra...4662.raw | not available | raw | Unknown | Unknown | 0 | | C:/.../bra...4663.raw | not available | raw | Unknown | Unknown | 0 | | C:/.../bra...4664.raw | not available | raw | Unknown | Unknown | 0 | | C:/.../bra...4665.raw | not available | raw | Unknown | Unknown | 0 | | C:/.../bra...4666.raw | not available | raw | Unknown | Unknown | 0 | | C:/.../bra...4667.raw | not available | raw | Unknown | Unknown | 0 | | C:/.../bra...4668.raw | not available | raw | Unknown | Unknown | 0 | | C:/.../bra...4669.raw | not available | raw | Unknown | Unknown | 0 | | C:/.../bra...4670.raw | not available | raw | Unknown | Unknown | 0 | | C:/.../bra...4671.raw | not available | raw | Unknown | Unknown | 0 | | C:/.../bra...4672.raw | not available | raw | Unknown | Unknown | 0 | | C:/.../bra...4673.raw | not available | raw | Unknown | Unknown | 0 | | C:/.../bra...4674.raw | not available | raw | Unknown | Unknown | 0 | | C:/.../bra...4675.raw | not available | raw | Unknown | Unknown | 0 | | C:/.../bra...4676.raw | not available | raw | Unknown | Unknown | 0 | | C:/.../bra...4677.raw | not available | raw | Unknown | Unknown | 0 |  Scanner Manufacturer Information |  |  | | --- | --- | | Name: |  | | Adresse: |  | | Internetseite: |  | | Scanner: |  | | Scansoftware: |  |  Component Information |  |  | | --- | --- | | Beschreibung: |  | | Losnummer: |  | | Seriennummer: |  |  Scan Information |  |  | | --- | --- | | Röhrenspannung: |  | | Röhrenstromstärke: |  | | Scandauer: |  | | Rekonstruktionsdauer: |  | | Gesamtverarbeitungszeit: |  | | Rekonstruktionsalgorithmus: |  | | Scanmethode: |  | | Geometrie: |  | | Integrationszeit: |  | | Filtern: |  | | Projektionsanzahl: |  | | Datum, Zeit: |  | | Benutzer: |  |  Reconstruction Parameters NO RECONSTRUCTION PARAMETERS AVAILABLE  Import Settings |  |  |  |  | | --- | --- | --- | --- | | Source/Type: | Reconstructed Volume | | | | Name: | Vereinigung weiss skelett aus Volumen 1 | | | | Axes swap mode: | XYZT | | | | Mirror axes: | None | | | | Data mapping: | Ramp | | | | Data range source mapping: | -1...1 | | | | Data range destination mapping: | -1...1 | | | | Data type mapping: | Unknown | | | | Voxel skip: | 0 | 0 | 0 | | Auto region of interest: | Off | | Region of interest (min): | 0 | 0 | 0 | | Region of interest (max): | -1 | -1 | -1 | | Slice interpolation mode: | Off | | | | Slice interpolation threshold: | 0 | | | | Resolution [mm]: | 1 | 1 | 1 | | Resampling mode: | Off | | | | Auto histogram mode: | Off | | Lower auto histogram boundary at (%): | 0 | | Upper auto histogram boundary at (%): | 0 |  Analysis Information |  |  | | --- | --- | | Wall thickness: | 0 | | Defect detection: | 0 | | Nominal/actual comparison: | 0 | | Number of reference objects: | 0 |  File List (678) | files (678) | dimensions | format | type | endian | header | | --- | --- | --- | --- | --- | --- | | C:/.../wei...F000.raw | not available | raw | Unknown | Unknown | 0 | | C:/.../wei...F001.raw | not available | raw | Unknown | Unknown | 0 | | C:/.../wei...F002.raw | not available | raw | Unknown | Unknown | 0 | | C:/.../wei...F003.raw | not available | raw | Unknown | Unknown | 0 | | C:/.../wei...F004.raw | not available | raw | Unknown | Unknown | 0 | | C:/.../wei...F005.raw | not available | raw | Unknown | Unknown | 0 | | C:/.../wei...F006.raw | not available | raw | Unknown | Unknown | 0 | | C:/.../wei...F007.raw | not available | raw | Unknown | Unknown | 0 | | C:/.../wei...F008.raw | not available | raw | Unknown | Unknown | 0 | | C:/.../wei...F009.raw | not available | raw | Unknown | Unknown | 0 | | C:/.../wei...F010.raw | not available | raw | Unknown | Unknown | 0 | | C:/.../wei...F011.raw | not available | raw | Unknown | Unknown | 0 | | C:/.../wei...F012.raw | not available | raw | Unknown | Unknown | 0 | | C:/.../wei...F013.raw | not available | raw | Unknown | Unknown | 0 | | C:/.../wei...F014.raw | not available | raw | Unknown | Unknown | 0 | | C:/.../wei...F015.raw | not available | raw | Unknown | Unknown | 0 | | C:/.../wei...F016.raw | not available | raw | Unknown | Unknown | 0 | | C:/.../wei...F017.raw | not available | raw | Unknown | Unknown | 0 | | C:/.../wei...F018.raw | not available | raw | Unknown | Unknown | 0 | | C:/.../wei...F019.raw | not available | raw | Unknown | Unknown | 0 | | C:/.../wei...F020.raw | not available | raw | Unknown | Unknown | 0 | | C:/.../wei...F021.raw | not available | raw | Unknown | Unknown | 0 | | C:/.../wei...F022.raw | not available | raw | Unknown | Unknown | 0 | | C:/.../wei...F023.raw | not available | raw | Unknown | Unknown | 0 | | C:/.../wei...F024.raw | not available | raw | Unknown | Unknown | 0 | | C:/.../wei...F025.raw | not available | raw | Unknown | Unknown | 0 | | C:/.../wei...F026.raw | not available | raw | Unknown | Unknown | 0 | | C:/.../wei...F027.raw | not available | raw | Unknown | Unknown | 0 | | C:/.../wei...F028.raw | not available | raw | Unknown | Unknown | 0 | | C:/.../wei...F029.raw | not available | raw | Unknown | Unknown | 0 | | C:/.../wei...F030.raw | not available | raw | Unknown | Unknown | 0 | | C:/.../wei...F031.raw | not available | raw | Unknown | Unknown | 0 | | C:/.../wei...F032.raw | not available | raw | Unknown | Unknown | 0 | | C:/.../wei...F033.raw | not available | raw | Unknown | Unknown | 0 | | C:/.../wei...F034.raw | not available | raw | Unknown | Unknown | 0 | | C:/.../wei...F035.raw | not available | raw | Unknown | Unknown | 0 | | C:/.../wei...F036.raw | not available | raw | Unknown | Unknown | 0 | | C:/.../wei...F037.raw | not available | raw | Unknown | Unknown | 0 | | C:/.../wei...F038.raw | not available | raw | Unknown | Unknown | 0 | | C:/.../wei...F039.raw | not available | raw | Unknown | Unknown | 0 | | C:/.../wei...F040.raw | not available | raw | Unknown | Unknown | 0 | | C:/.../wei...F041.raw | not available | raw | Unknown | Unknown | 0 | | C:/.../wei...F042.raw | not available | raw | Unknown | Unknown | 0 | | C:/.../wei...F043.raw | not available | raw | Unknown | Unknown | 0 | | C:/.../wei...F044.raw | not available | raw | Unknown | Unknown | 0 | | C:/.../wei...F045.raw | not available | raw | Unknown | Unknown | 0 | | C:/.../wei...F046.raw | not available | raw | Unknown | Unknown | 0 | | C:/.../wei...F047.raw | not available | raw | Unknown | Unknown | 0 | | C:/.../wei...F048.raw | not available | raw | Unknown | Unknown | 0 | | C:/.../wei...F049.raw | not available | raw | Unknown | Unknown | 0 | | C:/.../wei...F050.raw | not available | raw | Unknown | Unknown | 0 | | C:/.../wei...F051.raw | not available | raw | Unknown | Unknown | 0 | | C:/.../wei...F052.raw | not available | raw | Unknown | Unknown | 0 | | C:/.../wei...F053.raw | not available | raw | Unknown | Unknown | 0 | | C:/.../wei...F054.raw | not available | raw | Unknown | Unknown | 0 | | C:/.../wei...F055.raw | not available | raw | Unknown | Unknown | 0 | | C:/.../wei...F056.raw | not available | raw | Unknown | Unknown | 0 | | C:/.../wei...F057.raw | not available | raw | Unknown | Unknown | 0 | | C:/.../wei...F058.raw | not available | raw | Unknown | Unknown | 0 | | C:/.../wei...F059.raw | not available | raw | Unknown | Unknown | 0 | | C:/.../wei...F060.raw | not available | raw | Unknown | Unknown | 0 | | C:/.../wei...F061.raw | not available | raw | Unknown | Unknown | 0 | | C:/.../wei...F062.raw | not available | raw | Unknown | Unknown | 0 | | C:/.../wei...F063.raw | not available | raw | Unknown | Unknown | 0 | | C:/.../wei...F064.raw | not available | raw | Unknown | Unknown | 0 | | C:/.../wei...F065.raw | not available | raw | Unknown | Unknown | 0 | | C:/.../wei...F066.raw | not available | raw | Unknown | Unknown | 0 | | C:/.../wei...F067.raw | not available | raw | Unknown | Unknown | 0 | | C:/.../wei...F068.raw | not available | raw | Unknown | Unknown | 0 | | C:/.../wei...F069.raw | not available | raw | Unknown | Unknown | 0 | | C:/.../wei...F070.raw | not available | raw | Unknown | Unknown | 0 | | C:/.../wei...F071.raw | not available | raw | Unknown | Unknown | 0 | | C:/.../wei...F072.raw | not available | raw | Unknown | Unknown | 0 | | C:/.../wei...F073.raw | not available | raw | Unknown | Unknown | 0 | | C:/.../wei...F074.raw | not available | raw | Unknown | Unknown | 0 | | C:/.../wei...F075.raw | not available | raw | Unknown | Unknown | 0 | | C:/.../wei...F076.raw | not available | raw | Unknown | Unknown | 0 | | C:/.../wei...F077.raw | not available | raw | Unknown | Unknown | 0 | | C:/.../wei...F078.raw | not available | raw | Unknown | Unknown | 0 | | C:/.../wei...F079.raw | not available | raw | Unknown | Unknown | 0 | | C:/.../wei...F080.raw | not available | raw | Unknown | Unknown | 0 | | C:/.../wei...F081.raw | not available | raw | Unknown | Unknown | 0 | | C:/.../wei...F082.raw | not available | raw | Unknown | Unknown | 0 | | C:/.../wei...F083.raw | not available | raw | Unknown | Unknown | 0 | | C:/.../wei...F084.raw | not available | raw | Unknown | Unknown | 0 | | C:/.../wei...F085.raw | not available | raw | Unknown | Unknown | 0 | | C:/.../wei...F086.raw | not available | raw | Unknown | Unknown | 0 | | C:/.../wei...F087.raw | not available | raw | Unknown | Unknown | 0 | | C:/.../wei...F088.raw | not available | raw | Unknown | Unknown | 0 | | C:/.../wei...F089.raw | not available | raw | Unknown | Unknown | 0 | | C:/.../wei...F090.raw | not available | raw | Unknown | Unknown | 0 | | C:/.../wei...F091.raw | not available | raw | Unknown | Unknown | 0 | | C:/.../wei...F092.raw | not available | raw | Unknown | Unknown | 0 | | C:/.../wei...F093.raw | not available | raw | Unknown | Unknown | 0 | | C:/.../wei...F094.raw | not available | raw | Unknown | Unknown | 0 | | C:/.../wei...F095.raw | not available | raw | Unknown | Unknown | 0 | | C:/.../wei...F096.raw | not available | raw | Unknown | Unknown | 0 | | C:/.../wei...F097.raw | not available | raw | Unknown | Unknown | 0 | | C:/.../wei...F098.raw | not available | raw | Unknown | Unknown | 0 | | C:/.../wei...F099.raw | not available | raw | Unknown | Unknown | 0 | | C:/.../wei...F100.raw | not available | raw | Unknown | Unknown | 0 | | C:/.../wei...F101.raw | not available | raw | Unknown | Unknown | 0 | | C:/.../wei...F102.raw | not available | raw | Unknown | Unknown | 0 | | C:/.../wei...F103.raw | not available | raw | Unknown | Unknown | 0 | | C:/.../wei...F104.raw | not available | raw | Unknown | Unknown | 0 | | C:/.../wei...F105.raw | not available | raw | Unknown | Unknown | 0 | | C:/.../wei...F106.raw | not available | raw | Unknown | Unknown | 0 | | C:/.../wei...F107.raw | not available | raw | Unknown | Unknown | 0 | | C:/.../wei...F108.raw | not available | raw | Unknown | Unknown | 0 | | C:/.../wei...F109.raw | not available | raw | Unknown | Unknown | 0 | | C:/.../wei...F110.raw | not available | raw | Unknown | Unknown | 0 | | C:/.../wei...F111.raw | not available | raw | Unknown | Unknown | 0 | | C:/.../wei...F112.raw | not available | raw | Unknown | Unknown | 0 | | C:/.../wei...F113.raw | not available | raw | Unknown | Unknown | 0 | | C:/.../wei...F114.raw | not available | raw | Unknown | Unknown | 0 | | C:/.../wei...F115.raw | not available | raw | Unknown | Unknown | 0 | | C:/.../wei...F116.raw | not available | raw | Unknown | Unknown | 0 | | C:/.../wei...F117.raw | not available | raw | Unknown | Unknown | 0 | | C:/.../wei...F118.raw | not available | raw | Unknown | Unknown | 0 | | C:/.../wei...F119.raw | not available | raw | Unknown | Unknown | 0 | | C:/.../wei...F120.raw | not available | raw | Unknown | Unknown | 0 | | C:/.../wei...F121.raw | not available | raw | Unknown | Unknown | 0 | | C:/.../wei...F122.raw | not available | raw | Unknown | Unknown | 0 | | C:/.../wei...F123.raw | not available | raw | Unknown | Unknown | 0 | | C:/.../wei...F124.raw | not available | raw | Unknown | Unknown | 0 | | C:/.../wei...F125.raw | not available | raw | Unknown | Unknown | 0 | | C:/.../wei...F126.raw | not available | raw | Unknown | Unknown | 0 | | C:/.../wei...F127.raw | not available | raw | Unknown | Unknown | 0 | | C:/.../wei...F128.raw | not available | raw | Unknown | Unknown | 0 | | C:/.../wei...F129.raw | not available | raw | Unknown | Unknown | 0 | | C:/.../wei...F130.raw | not available | raw | Unknown | Unknown | 0 | | C:/.../wei...F131.raw | not available | raw | Unknown | Unknown | 0 | | C:/.../wei...F132.raw | not available | raw | Unknown | Unknown | 0 | | C:/.../wei...F133.raw | not available | raw | Unknown | Unknown | 0 | | C:/.../wei...F134.raw | not available | raw | Unknown | Unknown | 0 | | C:/.../wei...F135.raw | not available | raw | Unknown | Unknown | 0 | | C:/.../wei...F136.raw | not available | raw | Unknown | Unknown | 0 | | C:/.../wei...F137.raw | not available | raw | Unknown | Unknown | 0 | | C:/.../wei...F138.raw | not available | raw | Unknown | Unknown | 0 | | C:/.../wei...F139.raw | not available | raw | Unknown | Unknown | 0 | | C:/.../wei...F140.raw | not available | raw | Unknown | Unknown | 0 | | C:/.../wei...F141.raw | not available | raw | Unknown | Unknown | 0 | | C:/.../wei...F142.raw | not available | raw | Unknown | Unknown | 0 | | C:/.../wei...F143.raw | not available | raw | Unknown | Unknown | 0 | | C:/.../wei...F144.raw | not available | raw | Unknown | Unknown | 0 | | C:/.../wei...F145.raw | not available | raw | Unknown | Unknown | 0 | | C:/.../wei...F146.raw | not available | raw | Unknown | Unknown | 0 | | C:/.../wei...F147.raw | not available | raw | Unknown | Unknown | 0 | | C:/.../wei...F148.raw | not available | raw | Unknown | Unknown | 0 | | C:/.../wei...F149.raw | not available | raw | Unknown | Unknown | 0 | | C:/.../wei...F150.raw | not available | raw | Unknown | Unknown | 0 | | C:/.../wei...F151.raw | not available | raw | Unknown | Unknown | 0 | | C:/.../wei...F152.raw | not available | raw | Unknown | Unknown | 0 | | C:/.../wei...F153.raw | not available | raw | Unknown | Unknown | 0 | | C:/.../wei...F154.raw | not available | raw | Unknown | Unknown | 0 | | C:/.../wei...F155.raw | not available | raw | Unknown | Unknown | 0 | | C:/.../wei...F156.raw | not available | raw | Unknown | Unknown | 0 | | C:/.../wei...F157.raw | not available | raw | Unknown | Unknown | 0 | | C:/.../wei...F158.raw | not available | raw | Unknown | Unknown | 0 | | C:/.../wei...F159.raw | not available | raw | Unknown | Unknown | 0 | | C:/.../wei...F160.raw | not available | raw | Unknown | Unknown | 0 | | C:/.../wei...F161.raw | not available | raw | Unknown | Unknown | 0 | | C:/.../wei...F162.raw | not available | raw | Unknown | Unknown | 0 | | C:/.../wei...F163.raw | not available | raw | Unknown | Unknown | 0 | | C:/.../wei...F164.raw | not available | raw | Unknown | Unknown | 0 | | C:/.../wei...F165.raw | not available | raw | Unknown | Unknown | 0 | | C:/.../wei...F166.raw | not available | raw | Unknown | Unknown | 0 | | C:/.../wei...F167.raw | not available | raw | Unknown | Unknown | 0 | | C:/.../wei...F168.raw | not available | raw | Unknown | Unknown | 0 | | C:/.../wei...F169.raw | not available | raw | Unknown | Unknown | 0 | | C:/.../wei...F170.raw | not available | raw | Unknown | Unknown | 0 | | C:/.../wei...F171.raw | not available | raw | Unknown | Unknown | 0 | | C:/.../wei...F172.raw | not available | raw | Unknown | Unknown | 0 | | C:/.../wei...F173.raw | not available | raw | Unknown | Unknown | 0 | | C:/.../wei...F174.raw | not available | raw | Unknown | Unknown | 0 | | C:/.../wei...F175.raw | not available | raw | Unknown | Unknown | 0 | | C:/.../wei...F176.raw | not available | raw | Unknown | Unknown | 0 | | C:/.../wei...F177.raw | not available | raw | Unknown | Unknown | 0 | | C:/.../wei...F178.raw | not available | raw | Unknown | Unknown | 0 | | C:/.../wei...F179.raw | not available | raw | Unknown | Unknown | 0 | | C:/.../wei...F180.raw | not available | raw | Unknown | Unknown | 0 | | C:/.../wei...F181.raw | not available | raw | Unknown | Unknown | 0 | | C:/.../wei...F182.raw | not available | raw | Unknown | Unknown | 0 | | C:/.../wei...F183.raw | not available | raw | Unknown | Unknown | 0 | | C:/.../wei...F184.raw | not available | raw | Unknown | Unknown | 0 | | C:/.../wei...F185.raw | not available | raw | Unknown | Unknown | 0 | | C:/.../wei...F186.raw | not available | raw | Unknown | Unknown | 0 | | C:/.../wei...F187.raw | not available | raw | Unknown | Unknown | 0 | | C:/.../wei...F188.raw | not available | raw | Unknown | Unknown | 0 | | C:/.../wei...F189.raw | not available | raw | Unknown | Unknown | 0 | | C:/.../wei...F190.raw | not available | raw | Unknown | Unknown | 0 | | C:/.../wei...F191.raw | not available | raw | Unknown | Unknown | 0 | | C:/.../wei...F192.raw | not available | raw | Unknown | Unknown | 0 | | C:/.../wei...F193.raw | not available | raw | Unknown | Unknown | 0 | | C:/.../wei...F194.raw | not available | raw | Unknown | Unknown | 0 | | C:/.../wei...F195.raw | not available | raw | Unknown | Unknown | 0 | | C:/.../wei...F196.raw | not available | raw | Unknown | Unknown | 0 | | C:/.../wei...F197.raw | not available | raw | Unknown | Unknown | 0 | | C:/.../wei...F198.raw | not available | raw | Unknown | Unknown | 0 | | C:/.../wei...F199.raw | not available | raw | Unknown | Unknown | 0 | | C:/.../wei...F200.raw | not available | raw | Unknown | Unknown | 0 | | C:/.../wei...F201.raw | not available | raw | Unknown | Unknown | 0 | | C:/.../wei...F202.raw | not available | raw | Unknown | Unknown | 0 | | C:/.../wei...F203.raw | not available | raw | Unknown | Unknown | 0 | | C:/.../wei...F204.raw | not available | raw | Unknown | Unknown | 0 | | C:/.../wei...F205.raw | not available | raw | Unknown | Unknown | 0 | | C:/.../wei...F206.raw | not available | raw | Unknown | Unknown | 0 | | C:/.../wei...F207.raw | not available | raw | Unknown | Unknown | 0 | | C:/.../wei...F208.raw | not available | raw | Unknown | Unknown | 0 | | C:/.../wei...F209.raw | not available | raw | Unknown | Unknown | 0 | | C:/.../wei...F210.raw | not available | raw | Unknown | Unknown | 0 | | C:/.../wei...F211.raw | not available | raw | Unknown | Unknown | 0 | | C:/.../wei...F212.raw | not available | raw | Unknown | Unknown | 0 | | C:/.../wei...F213.raw | not available | raw | Unknown | Unknown | 0 | | C:/.../wei...F214.raw | not available | raw | Unknown | Unknown | 0 | | C:/.../wei...F215.raw | not available | raw | Unknown | Unknown | 0 | | C:/.../wei...F216.raw | not available | raw | Unknown | Unknown | 0 | | C:/.../wei...F217.raw | not available | raw | Unknown | Unknown | 0 | | C:/.../wei...F218.raw | not available | raw | Unknown | Unknown | 0 | | C:/.../wei...F219.raw | not available | raw | Unknown | Unknown | 0 | | C:/.../wei...F220.raw | not available | raw | Unknown | Unknown | 0 | | C:/.../wei...F221.raw | not available | raw | Unknown | Unknown | 0 | | C:/.../wei...F222.raw | not available | raw | Unknown | Unknown | 0 | | C:/.../wei...F223.raw | not available | raw | Unknown | Unknown | 0 | | C:/.../wei...F224.raw | not available | raw | Unknown | Unknown | 0 | | C:/.../wei...F225.raw | not available | raw | Unknown | Unknown | 0 | | C:/.../wei...F226.raw | not available | raw | Unknown | Unknown | 0 | | C:/.../wei...F227.raw | not available | raw | Unknown | Unknown | 0 | | C:/.../wei...F228.raw | not available | raw | Unknown | Unknown | 0 | | C:/.../wei...F229.raw | not available | raw | Unknown | Unknown | 0 | | C:/.../wei...F230.raw | not available | raw | Unknown | Unknown | 0 | | C:/.../wei...F231.raw | not available | raw | Unknown | Unknown | 0 | | C:/.../wei...F232.raw | not available | raw | Unknown | Unknown | 0 | | C:/.../wei...F233.raw | not available | raw | Unknown | Unknown | 0 | | C:/.../wei...F234.raw | not available | raw | Unknown | Unknown | 0 | | C:/.../wei...F235.raw | not available | raw | Unknown | Unknown | 0 | | C:/.../wei...F236.raw | not available | raw | Unknown | Unknown | 0 | | C:/.../wei...F237.raw | not available | raw | Unknown | Unknown | 0 | | C:/.../wei...F238.raw | not available | raw | Unknown | Unknown | 0 | | C:/.../wei...F239.raw | not available | raw | Unknown | Unknown | 0 | | C:/.../wei...F240.raw | not available | raw | Unknown | Unknown | 0 | | C:/.../wei...F241.raw | not available | raw | Unknown | Unknown | 0 | | C:/.../wei...F242.raw | not available | raw | Unknown | Unknown | 0 | | C:/.../wei...F243.raw | not available | raw | Unknown | Unknown | 0 | | C:/.../wei...F244.raw | not available | raw | Unknown | Unknown | 0 | | C:/.../wei...F245.raw | not available | raw | Unknown | Unknown | 0 | | C:/.../wei...F246.raw | not available | raw | Unknown | Unknown | 0 | | C:/.../wei...F247.raw | not available | raw | Unknown | Unknown | 0 | | C:/.../wei...F248.raw | not available | raw | Unknown | Unknown | 0 | | C:/.../wei...F249.raw | not available | raw | Unknown | Unknown | 0 | | C:/.../wei...F250.raw | not available | raw | Unknown | Unknown | 0 | | C:/.../wei...F251.raw | not available | raw | Unknown | Unknown | 0 | | C:/.../wei...F252.raw | not available | raw | Unknown | Unknown | 0 | | C:/.../wei...F253.raw | not available | raw | Unknown | Unknown | 0 | | C:/.../wei...F254.raw | not available | raw | Unknown | Unknown | 0 | | C:/.../wei...F255.raw | not available | raw | Unknown | Unknown | 0 | | C:/.../wei...F256.raw | not available | raw | Unknown | Unknown | 0 | | C:/.../wei...F257.raw | not available | raw | Unknown | Unknown | 0 | | C:/.../wei...F258.raw | not available | raw | Unknown | Unknown | 0 | | C:/.../wei...F259.raw | not available | raw | Unknown | Unknown | 0 | | C:/.../wei...F260.raw | not available | raw | Unknown | Unknown | 0 | | C:/.../wei...F261.raw | not available | raw | Unknown | Unknown | 0 | | C:/.../wei...F262.raw | not available | raw | Unknown | Unknown | 0 | | C:/.../wei...F263.raw | not available | raw | Unknown | Unknown | 0 | | C:/.../wei...F264.raw | not available | raw | Unknown | Unknown | 0 | | C:/.../wei...F265.raw | not available | raw | Unknown | Unknown | 0 | | C:/.../wei...F266.raw | not available | raw | Unknown | Unknown | 0 | | C:/.../wei...F267.raw | not available | raw | Unknown | Unknown | 0 | | C:/.../wei...F268.raw | not available | raw | Unknown | Unknown | 0 | | C:/.../wei...F269.raw | not available | raw | Unknown | Unknown | 0 | | C:/.../wei...F270.raw | not available | raw | Unknown | Unknown | 0 | | C:/.../wei...F271.raw | not available | raw | Unknown | Unknown | 0 | | C:/.../wei...F272.raw | not available | raw | Unknown | Unknown | 0 | | C:/.../wei...F273.raw | not available | raw | Unknown | Unknown | 0 | | C:/.../wei...F274.raw | not available | raw | Unknown | Unknown | 0 | | C:/.../wei...F275.raw | not available | raw | Unknown | Unknown | 0 | | C:/.../wei...F276.raw | not available | raw | Unknown | Unknown | 0 | | C:/.../wei...F277.raw | not available | raw | Unknown | Unknown | 0 | | C:/.../wei...F278.raw | not available | raw | Unknown | Unknown | 0 | | C:/.../wei...F279.raw | not available | raw | Unknown | Unknown | 0 | | C:/.../wei...F280.raw | not available | raw | Unknown | Unknown | 0 | | C:/.../wei...F281.raw | not available | raw | Unknown | Unknown | 0 | | C:/.../wei...F282.raw | not available | raw | Unknown | Unknown | 0 | | C:/.../wei...F283.raw | not available | raw | Unknown | Unknown | 0 | | C:/.../wei...F284.raw | not available | raw | Unknown | Unknown | 0 | | C:/.../wei...F285.raw | not available | raw | Unknown | Unknown | 0 | | C:/.../wei...F286.raw | not available | raw | Unknown | Unknown | 0 | | C:/.../wei...F287.raw | not available | raw | Unknown | Unknown | 0 | | C:/.../wei...F288.raw | not available | raw | Unknown | Unknown | 0 | | C:/.../wei...F289.raw | not available | raw | Unknown | Unknown | 0 | | C:/.../wei...F290.raw | not available | raw | Unknown | Unknown | 0 | | C:/.../wei...F291.raw | not available | raw | Unknown | Unknown | 0 | | C:/.../wei...F292.raw | not available | raw | Unknown | Unknown | 0 | | C:/.../wei...F293.raw | not available | raw | Unknown | Unknown | 0 | | C:/.../wei...F294.raw | not available | raw | Unknown | Unknown | 0 | | C:/.../wei...F295.raw | not available | raw | Unknown | Unknown | 0 | | C:/.../wei...F296.raw | not available | raw | Unknown | Unknown | 0 | | C:/.../wei...F297.raw | not available | raw | Unknown | Unknown | 0 | | C:/.../wei...F298.raw | not available | raw | Unknown | Unknown | 0 | | C:/.../wei...F299.raw | not available | raw | Unknown | Unknown | 0 | | C:/.../wei...F300.raw | not available | raw | Unknown | Unknown | 0 | | C:/.../wei...F301.raw | not available | raw | Unknown | Unknown | 0 | | C:/.../wei...F302.raw | not available | raw | Unknown | Unknown | 0 | | C:/.../wei...F303.raw | not available | raw | Unknown | Unknown | 0 | | C:/.../wei...F304.raw | not available | raw | Unknown | Unknown | 0 | | C:/.../wei...F305.raw | not available | raw | Unknown | Unknown | 0 | | C:/.../wei...F306.raw | not available | raw | Unknown | Unknown | 0 | | C:/.../wei...F307.raw | not available | raw | Unknown | Unknown | 0 | | C:/.../wei...F308.raw | not available | raw | Unknown | Unknown | 0 | | C:/.../wei...F309.raw | not available | raw | Unknown | Unknown | 0 | | C:/.../wei...F310.raw | not available | raw | Unknown | Unknown | 0 | | C:/.../wei...F311.raw | not available | raw | Unknown | Unknown | 0 | | C:/.../wei...F312.raw | not available | raw | Unknown | Unknown | 0 | | C:/.../wei...F313.raw | not available | raw | Unknown | Unknown | 0 | | C:/.../wei...F314.raw | not available | raw | Unknown | Unknown | 0 | | C:/.../wei...F315.raw | not available | raw | Unknown | Unknown | 0 | | C:/.../wei...F316.raw | not available | raw | Unknown | Unknown | 0 | | C:/.../wei...F317.raw | not available | raw | Unknown | Unknown | 0 | | C:/.../wei...F318.raw | not available | raw | Unknown | Unknown | 0 | | C:/.../wei...F319.raw | not available | raw | Unknown | Unknown | 0 | | C:/.../wei...F320.raw | not available | raw | Unknown | Unknown | 0 | | C:/.../wei...F321.raw | not available | raw | Unknown | Unknown | 0 | | C:/.../wei...F322.raw | not available | raw | Unknown | Unknown | 0 | | C:/.../wei...F323.raw | not available | raw | Unknown | Unknown | 0 | | C:/.../wei...F324.raw | not available | raw | Unknown | Unknown | 0 | | C:/.../wei...F325.raw | not available | raw | Unknown | Unknown | 0 | | C:/.../wei...F326.raw | not available | raw | Unknown | Unknown | 0 | | C:/.../wei...F327.raw | not available | raw | Unknown | Unknown | 0 | | C:/.../wei...F328.raw | not available | raw | Unknown | Unknown | 0 | | C:/.../wei...F329.raw | not available | raw | Unknown | Unknown | 0 | | C:/.../wei...F330.raw | not available | raw | Unknown | Unknown | 0 | | C:/.../wei...F331.raw | not available | raw | Unknown | Unknown | 0 | | C:/.../wei...F332.raw | not available | raw | Unknown | Unknown | 0 | | C:/.../wei...F333.raw | not available | raw | Unknown | Unknown | 0 | | C:/.../wei...F334.raw | not available | raw | Unknown | Unknown | 0 | | C:/.../wei...F335.raw | not available | raw | Unknown | Unknown | 0 | | C:/.../wei...F336.raw | not available | raw | Unknown | Unknown | 0 | | C:/.../wei...F337.raw | not available | raw | Unknown | Unknown | 0 | | C:/.../wei...F338.raw | not available | raw | Unknown | Unknown | 0 | | C:/.../wei...F339.raw | not available | raw | Unknown | Unknown | 0 | | C:/.../wei...F340.raw | not available | raw | Unknown | Unknown | 0 | | C:/.../wei...F341.raw | not available | raw | Unknown | Unknown | 0 | | C:/.../wei...F342.raw | not available | raw | Unknown | Unknown | 0 | | C:/.../wei...F343.raw | not available | raw | Unknown | Unknown | 0 | | C:/.../wei...F344.raw | not available | raw | Unknown | Unknown | 0 | | C:/.../wei...F345.raw | not available | raw | Unknown | Unknown | 0 | | C:/.../wei...F346.raw | not available | raw | Unknown | Unknown | 0 | | C:/.../wei...F347.raw | not available | raw | Unknown | Unknown | 0 | | C:/.../wei...F348.raw | not available | raw | Unknown | Unknown | 0 | | C:/.../wei...F349.raw | not available | raw | Unknown | Unknown | 0 | | C:/.../wei...F350.raw | not available | raw | Unknown | Unknown | 0 | | C:/.../wei...F351.raw | not available | raw | Unknown | Unknown | 0 | | C:/.../wei...F352.raw | not available | raw | Unknown | Unknown | 0 | | C:/.../wei...F353.raw | not available | raw | Unknown | Unknown | 0 | | C:/.../wei...F354.raw | not available | raw | Unknown | Unknown | 0 | | C:/.../wei...F355.raw | not available | raw | Unknown | Unknown | 0 | | C:/.../wei...F356.raw | not available | raw | Unknown | Unknown | 0 | | C:/.../wei...F357.raw | not available | raw | Unknown | Unknown | 0 | | C:/.../wei...F358.raw | not available | raw | Unknown | Unknown | 0 | | C:/.../wei...F359.raw | not available | raw | Unknown | Unknown | 0 | | C:/.../wei...F360.raw | not available | raw | Unknown | Unknown | 0 | | C:/.../wei...F361.raw | not available | raw | Unknown | Unknown | 0 | | C:/.../wei...F362.raw | not available | raw | Unknown | Unknown | 0 | | C:/.../wei...F363.raw | not available | raw | Unknown | Unknown | 0 | | C:/.../wei...F364.raw | not available | raw | Unknown | Unknown | 0 | | C:/.../wei...F365.raw | not available | raw | Unknown | Unknown | 0 | | C:/.../wei...F366.raw | not available | raw | Unknown | Unknown | 0 | | C:/.../wei...F367.raw | not available | raw | Unknown | Unknown | 0 | | C:/.../wei...F368.raw | not available | raw | Unknown | Unknown | 0 | | C:/.../wei...F369.raw | not available | raw | Unknown | Unknown | 0 | | C:/.../wei...F370.raw | not available | raw | Unknown | Unknown | 0 | | C:/.../wei...F371.raw | not available | raw | Unknown | Unknown | 0 | | C:/.../wei...F372.raw | not available | raw | Unknown | Unknown | 0 | | C:/.../wei...F373.raw | not available | raw | Unknown | Unknown | 0 | | C:/.../wei...F374.raw | not available | raw | Unknown | Unknown | 0 | | C:/.../wei...F375.raw | not available | raw | Unknown | Unknown | 0 | | C:/.../wei...F376.raw | not available | raw | Unknown | Unknown | 0 | | C:/.../wei...F377.raw | not available | raw | Unknown | Unknown | 0 | | C:/.../wei...F378.raw | not available | raw | Unknown | Unknown | 0 | | C:/.../wei...F379.raw | not available | raw | Unknown | Unknown | 0 | | C:/.../wei...F380.raw | not available | raw | Unknown | Unknown | 0 | | C:/.../wei...F381.raw | not available | raw | Unknown | Unknown | 0 | | C:/.../wei...F382.raw | not available | raw | Unknown | Unknown | 0 | | C:/.../wei...F383.raw | not available | raw | Unknown | Unknown | 0 | | C:/.../wei...F384.raw | not available | raw | Unknown | Unknown | 0 | | C:/.../wei...F385.raw | not available | raw | Unknown | Unknown | 0 | | C:/.../wei...F386.raw | not available | raw | Unknown | Unknown | 0 | | C:/.../wei...F387.raw | not available | raw | Unknown | Unknown | 0 | | C:/.../wei...F388.raw | not available | raw | Unknown | Unknown | 0 | | C:/.../wei...F389.raw | not available | raw | Unknown | Unknown | 0 | | C:/.../wei...F390.raw | not available | raw | Unknown | Unknown | 0 | | C:/.../wei...F391.raw | not available | raw | Unknown | Unknown | 0 | | C:/.../wei...F392.raw | not available | raw | Unknown | Unknown | 0 | | C:/.../wei...F393.raw | not available | raw | Unknown | Unknown | 0 | | C:/.../wei...F394.raw | not available | raw | Unknown | Unknown | 0 | | C:/.../wei...F395.raw | not available | raw | Unknown | Unknown | 0 | | C:/.../wei...F396.raw | not available | raw | Unknown | Unknown | 0 | | C:/.../wei...F397.raw | not available | raw | Unknown | Unknown | 0 | | C:/.../wei...F398.raw | not available | raw | Unknown | Unknown | 0 | | C:/.../wei...F399.raw | not available | raw | Unknown | Unknown | 0 | | C:/.../wei...F400.raw | not available | raw | Unknown | Unknown | 0 | | C:/.../wei...F401.raw | not available | raw | Unknown | Unknown | 0 | | C:/.../wei...F402.raw | not available | raw | Unknown | Unknown | 0 | | C:/.../wei...F403.raw | not available | raw | Unknown | Unknown | 0 | | C:/.../wei...F404.raw | not available | raw | Unknown | Unknown | 0 | | C:/.../wei...F405.raw | not available | raw | Unknown | Unknown | 0 | | C:/.../wei...F406.raw | not available | raw | Unknown | Unknown | 0 | | C:/.../wei...F407.raw | not available | raw | Unknown | Unknown | 0 | | C:/.../wei...F408.raw | not available | raw | Unknown | Unknown | 0 | | C:/.../wei...F409.raw | not available | raw | Unknown | Unknown | 0 | | C:/.../wei...F410.raw | not available | raw | Unknown | Unknown | 0 | | C:/.../wei...F411.raw | not available | raw | Unknown | Unknown | 0 | | C:/.../wei...F412.raw | not available | raw | Unknown | Unknown | 0 | | C:/.../wei...F413.raw | not available | raw | Unknown | Unknown | 0 | | C:/.../wei...F414.raw | not available | raw | Unknown | Unknown | 0 | | C:/.../wei...F415.raw | not available | raw | Unknown | Unknown | 0 | | C:/.../wei...F416.raw | not available | raw | Unknown | Unknown | 0 | | C:/.../wei...F417.raw | not available | raw | Unknown | Unknown | 0 | | C:/.../wei...F418.raw | not available | raw | Unknown | Unknown | 0 | | C:/.../wei...F419.raw | not available | raw | Unknown | Unknown | 0 | | C:/.../wei...F420.raw | not available | raw | Unknown | Unknown | 0 | | C:/.../wei...F421.raw | not available | raw | Unknown | Unknown | 0 | | C:/.../wei...F422.raw | not available | raw | Unknown | Unknown | 0 | | C:/.../wei...F423.raw | not available | raw | Unknown | Unknown | 0 | | C:/.../wei...F424.raw | not available | raw | Unknown | Unknown | 0 | | C:/.../wei...F425.raw | not available | raw | Unknown | Unknown | 0 | | C:/.../wei...F426.raw | not available | raw | Unknown | Unknown | 0 | | C:/.../wei...F427.raw | not available | raw | Unknown | Unknown | 0 | | C:/.../wei...F428.raw | not available | raw | Unknown | Unknown | 0 | | C:/.../wei...F429.raw | not available | raw | Unknown | Unknown | 0 | | C:/.../wei...F430.raw | not available | raw | Unknown | Unknown | 0 | | C:/.../wei...F431.raw | not available | raw | Unknown | Unknown | 0 | | C:/.../wei...F432.raw | not available | raw | Unknown | Unknown | 0 | | C:/.../wei...F433.raw | not available | raw | Unknown | Unknown | 0 | | C:/.../wei...F434.raw | not available | raw | Unknown | Unknown | 0 | | C:/.../wei...F435.raw | not available | raw | Unknown | Unknown | 0 | | C:/.../wei...F436.raw | not available | raw | Unknown | Unknown | 0 | | C:/.../wei...F437.raw | not available | raw | Unknown | Unknown | 0 | | C:/.../wei...F438.raw | not available | raw | Unknown | Unknown | 0 | | C:/.../wei...F439.raw | not available | raw | Unknown | Unknown | 0 | | C:/.../wei...F440.raw | not available | raw | Unknown | Unknown | 0 | | C:/.../wei...F441.raw | not available | raw | Unknown | Unknown | 0 | | C:/.../wei...F442.raw | not available | raw | Unknown | Unknown | 0 | | C:/.../wei...F443.raw | not available | raw | Unknown | Unknown | 0 | | C:/.../wei...F444.raw | not available | raw | Unknown | Unknown | 0 | | C:/.../wei...F445.raw | not available | raw | Unknown | Unknown | 0 | | C:/.../wei...F446.raw | not available | raw | Unknown | Unknown | 0 | | C:/.../wei...F447.raw | not available | raw | Unknown | Unknown | 0 | | C:/.../wei...F448.raw | not available | raw | Unknown | Unknown | 0 | | C:/.../wei...F449.raw | not available | raw | Unknown | Unknown | 0 | | C:/.../wei...F450.raw | not available | raw | Unknown | Unknown | 0 | | C:/.../wei...F451.raw | not available | raw | Unknown | Unknown | 0 | | C:/.../wei...F452.raw | not available | raw | Unknown | Unknown | 0 | | C:/.../wei...F453.raw | not available | raw | Unknown | Unknown | 0 | | C:/.../wei...F454.raw | not available | raw | Unknown | Unknown | 0 | | C:/.../wei...F455.raw | not available | raw | Unknown | Unknown | 0 | | C:/.../wei...F456.raw | not available | raw | Unknown | Unknown | 0 | | C:/.../wei...F457.raw | not available | raw | Unknown | Unknown | 0 | | C:/.../wei...F458.raw | not available | raw | Unknown | Unknown | 0 | | C:/.../wei...F459.raw | not available | raw | Unknown | Unknown | 0 | | C:/.../wei...F460.raw | not available | raw | Unknown | Unknown | 0 | | C:/.../wei...F461.raw | not available | raw | Unknown | Unknown | 0 | | C:/.../wei...F462.raw | not available | raw | Unknown | Unknown | 0 | | C:/.../wei...F463.raw | not available | raw | Unknown | Unknown | 0 | | C:/.../wei...F464.raw | not available | raw | Unknown | Unknown | 0 | | C:/.../wei...F465.raw | not available | raw | Unknown | Unknown | 0 | | C:/.../wei...F466.raw | not available | raw | Unknown | Unknown | 0 | | C:/.../wei...F467.raw | not available | raw | Unknown | Unknown | 0 | | C:/.../wei...F468.raw | not available | raw | Unknown | Unknown | 0 | | C:/.../wei...F469.raw | not available | raw | Unknown | Unknown | 0 | | C:/.../wei...F470.raw | not available | raw | Unknown | Unknown | 0 | | C:/.../wei...F471.raw | not available | raw | Unknown | Unknown | 0 | | C:/.../wei...F472.raw | not available | raw | Unknown | Unknown | 0 | | C:/.../wei...F473.raw | not available | raw | Unknown | Unknown | 0 | | C:/.../wei...F474.raw | not available | raw | Unknown | Unknown | 0 | | C:/.../wei...F475.raw | not available | raw | Unknown | Unknown | 0 | | C:/.../wei...F476.raw | not available | raw | Unknown | Unknown | 0 | | C:/.../wei...F477.raw | not available | raw | Unknown | Unknown | 0 | | C:/.../wei...F478.raw | not available | raw | Unknown | Unknown | 0 | | C:/.../wei...F479.raw | not available | raw | Unknown | Unknown | 0 | | C:/.../wei...F480.raw | not available | raw | Unknown | Unknown | 0 | | C:/.../wei...F481.raw | not available | raw | Unknown | Unknown | 0 | | C:/.../wei...F482.raw | not available | raw | Unknown | Unknown | 0 | | C:/.../wei...F483.raw | not available | raw | Unknown | Unknown | 0 | | C:/.../wei...F484.raw | not available | raw | Unknown | Unknown | 0 | | C:/.../wei...F485.raw | not available | raw | Unknown | Unknown | 0 | | C:/.../wei...F486.raw | not available | raw | Unknown | Unknown | 0 | | C:/.../wei...F487.raw | not available | raw | Unknown | Unknown | 0 | | C:/.../wei...F488.raw | not available | raw | Unknown | Unknown | 0 | | C:/.../wei...F489.raw | not available | raw | Unknown | Unknown | 0 | | C:/.../wei...F490.raw | not available | raw | Unknown | Unknown | 0 | | C:/.../wei...F491.raw | not available | raw | Unknown | Unknown | 0 | | C:/.../wei...F492.raw | not available | raw | Unknown | Unknown | 0 | | C:/.../wei...F493.raw | not available | raw | Unknown | Unknown | 0 | | C:/.../wei...F494.raw | not available | raw | Unknown | Unknown | 0 | | C:/.../wei...F495.raw | not available | raw | Unknown | Unknown | 0 | | C:/.../wei...F496.raw | not available | raw | Unknown | Unknown | 0 | | C:/.../wei...F497.raw | not available | raw | Unknown | Unknown | 0 | | C:/.../wei...F498.raw | not available | raw | Unknown | Unknown | 0 | | C:/.../wei...F499.raw | not available | raw | Unknown | Unknown | 0 | | C:/.../wei...F500.raw | not available | raw | Unknown | Unknown | 0 | | C:/.../wei...F501.raw | not available | raw | Unknown | Unknown | 0 | | C:/.../wei...F502.raw | not available | raw | Unknown | Unknown | 0 | | C:/.../wei...F503.raw | not available | raw | Unknown | Unknown | 0 | | C:/.../wei...F504.raw | not available | raw | Unknown | Unknown | 0 | | C:/.../wei...F505.raw | not available | raw | Unknown | Unknown | 0 | | C:/.../wei...F506.raw | not available | raw | Unknown | Unknown | 0 | | C:/.../wei...F507.raw | not available | raw | Unknown | Unknown | 0 | | C:/.../wei...F508.raw | not available | raw | Unknown | Unknown | 0 | | C:/.../wei...F509.raw | not available | raw | Unknown | Unknown | 0 | | C:/.../wei...F510.raw | not available | raw | Unknown | Unknown | 0 | | C:/.../wei...F511.raw | not available | raw | Unknown | Unknown | 0 | | C:/.../wei...F512.raw | not available | raw | Unknown | Unknown | 0 | | C:/.../wei...F513.raw | not available | raw | Unknown | Unknown | 0 | | C:/.../wei...F514.raw | not available | raw | Unknown | Unknown | 0 | | C:/.../wei...F515.raw | not available | raw | Unknown | Unknown | 0 | | C:/.../wei...F516.raw | not available | raw | Unknown | Unknown | 0 | | C:/.../wei...F517.raw | not available | raw | Unknown | Unknown | 0 | | C:/.../wei...F518.raw | not available | raw | Unknown | Unknown | 0 | | C:/.../wei...F519.raw | not available | raw | Unknown | Unknown | 0 | | C:/.../wei...F520.raw | not available | raw | Unknown | Unknown | 0 | | C:/.../wei...F521.raw | not available | raw | Unknown | Unknown | 0 | | C:/.../wei...F522.raw | not available | raw | Unknown | Unknown | 0 | | C:/.../wei...F523.raw | not available | raw | Unknown | Unknown | 0 | | C:/.../wei...F524.raw | not available | raw | Unknown | Unknown | 0 | | C:/.../wei...F525.raw | not available | raw | Unknown | Unknown | 0 | | C:/.../wei...F526.raw | not available | raw | Unknown | Unknown | 0 | | C:/.../wei...F527.raw | not available | raw | Unknown | Unknown | 0 | | C:/.../wei...F528.raw | not available | raw | Unknown | Unknown | 0 | | C:/.../wei...F529.raw | not available | raw | Unknown | Unknown | 0 | | C:/.../wei...F530.raw | not available | raw | Unknown | Unknown | 0 | | C:/.../wei...F531.raw | not available | raw | Unknown | Unknown | 0 | | C:/.../wei...F532.raw | not available | raw | Unknown | Unknown | 0 | | C:/.../wei...F533.raw | not available | raw | Unknown | Unknown | 0 | | C:/.../wei...F534.raw | not available | raw | Unknown | Unknown | 0 | | C:/.../wei...F535.raw | not available | raw | Unknown | Unknown | 0 | | C:/.../wei...F536.raw | not available | raw | Unknown | Unknown | 0 | | C:/.../wei...F537.raw | not available | raw | Unknown | Unknown | 0 | | C:/.../wei...F538.raw | not available | raw | Unknown | Unknown | 0 | | C:/.../wei...F539.raw | not available | raw | Unknown | Unknown | 0 | | C:/.../wei...F540.raw | not available | raw | Unknown | Unknown | 0 | | C:/.../wei...F541.raw | not available | raw | Unknown | Unknown | 0 | | C:/.../wei...F542.raw | not available | raw | Unknown | Unknown | 0 | | C:/.../wei...F543.raw | not available | raw | Unknown | Unknown | 0 | | C:/.../wei...F544.raw | not available | raw | Unknown | Unknown | 0 | | C:/.../wei...F545.raw | not available | raw | Unknown | Unknown | 0 | | C:/.../wei...F546.raw | not available | raw | Unknown | Unknown | 0 | | C:/.../wei...F547.raw | not available | raw | Unknown | Unknown | 0 | | C:/.../wei...F548.raw | not available | raw | Unknown | Unknown | 0 | | C:/.../wei...F549.raw | not available | raw | Unknown | Unknown | 0 | | C:/.../wei...F550.raw | not available | raw | Unknown | Unknown | 0 | | C:/.../wei...F551.raw | not available | raw | Unknown | Unknown | 0 | | C:/.../wei...F552.raw | not available | raw | Unknown | Unknown | 0 | | C:/.../wei...F553.raw | not available | raw | Unknown | Unknown | 0 | | C:/.../wei...F554.raw | not available | raw | Unknown | Unknown | 0 | | C:/.../wei...F555.raw | not available | raw | Unknown | Unknown | 0 | | C:/.../wei...F556.raw | not available | raw | Unknown | Unknown | 0 | | C:/.../wei...F557.raw | not available | raw | Unknown | Unknown | 0 | | C:/.../wei...F558.raw | not available | raw | Unknown | Unknown | 0 | | C:/.../wei...F559.raw | not available | raw | Unknown | Unknown | 0 | | C:/.../wei...F560.raw | not available | raw | Unknown | Unknown | 0 | | C:/.../wei...F561.raw | not available | raw | Unknown | Unknown | 0 | | C:/.../wei...F562.raw | not available | raw | Unknown | Unknown | 0 | | C:/.../wei...F563.raw | not available | raw | Unknown | Unknown | 0 | | C:/.../wei...F564.raw | not available | raw | Unknown | Unknown | 0 | | C:/.../wei...F565.raw | not available | raw | Unknown | Unknown | 0 | | C:/.../wei...F566.raw | not available | raw | Unknown | Unknown | 0 | | C:/.../wei...F567.raw | not available | raw | Unknown | Unknown | 0 | | C:/.../wei...F568.raw | not available | raw | Unknown | Unknown | 0 | | C:/.../wei...F569.raw | not available | raw | Unknown | Unknown | 0 | | C:/.../wei...F570.raw | not available | raw | Unknown | Unknown | 0 | | C:/.../wei...F571.raw | not available | raw | Unknown | Unknown | 0 | | C:/.../wei...F572.raw | not available | raw | Unknown | Unknown | 0 | | C:/.../wei...F573.raw | not available | raw | Unknown | Unknown | 0 | | C:/.../wei...F574.raw | not available | raw | Unknown | Unknown | 0 | | C:/.../wei...F575.raw | not available | raw | Unknown | Unknown | 0 | | C:/.../wei...F576.raw | not available | raw | Unknown | Unknown | 0 | | C:/.../wei...F577.raw | not available | raw | Unknown | Unknown | 0 | | C:/.../wei...F578.raw | not available | raw | Unknown | Unknown | 0 | | C:/.../wei...F579.raw | not available | raw | Unknown | Unknown | 0 | | C:/.../wei...F580.raw | not available | raw | Unknown | Unknown | 0 | | C:/.../wei...F581.raw | not available | raw | Unknown | Unknown | 0 | | C:/.../wei...F582.raw | not available | raw | Unknown | Unknown | 0 | | C:/.../wei...F583.raw | not available | raw | Unknown | Unknown | 0 | | C:/.../wei...F584.raw | not available | raw | Unknown | Unknown | 0 | | C:/.../wei...F585.raw | not available | raw | Unknown | Unknown | 0 | | C:/.../wei...F586.raw | not available | raw | Unknown | Unknown | 0 | | C:/.../wei...F587.raw | not available | raw | Unknown | Unknown | 0 | | C:/.../wei...F588.raw | not available | raw | Unknown | Unknown | 0 | | C:/.../wei...F589.raw | not available | raw | Unknown | Unknown | 0 | | C:/.../wei...F590.raw | not available | raw | Unknown | Unknown | 0 | | C:/.../wei...F591.raw | not available | raw | Unknown | Unknown | 0 | | C:/.../wei...F592.raw | not available | raw | Unknown | Unknown | 0 | | C:/.../wei...F593.raw | not available | raw | Unknown | Unknown | 0 | | C:/.../wei...F594.raw | not available | raw | Unknown | Unknown | 0 | | C:/.../wei...F595.raw | not available | raw | Unknown | Unknown | 0 | | C:/.../wei...F596.raw | not available | raw | Unknown | Unknown | 0 | | C:/.../wei...F597.raw | not available | raw | Unknown | Unknown | 0 | | C:/.../wei...F598.raw | not available | raw | Unknown | Unknown | 0 | | C:/.../wei...F599.raw | not available | raw | Unknown | Unknown | 0 | | C:/.../wei...F600.raw | not available | raw | Unknown | Unknown | 0 | | C:/.../wei...F601.raw | not available | raw | Unknown | Unknown | 0 | | C:/.../wei...F602.raw | not available | raw | Unknown | Unknown | 0 | | C:/.../wei...F603.raw | not available | raw | Unknown | Unknown | 0 | | C:/.../wei...F604.raw | not available | raw | Unknown | Unknown | 0 | | C:/.../wei...F605.raw | not available | raw | Unknown | Unknown | 0 | | C:/.../wei...F606.raw | not available | raw | Unknown | Unknown | 0 | | C:/.../wei...F607.raw | not available | raw | Unknown | Unknown | 0 | | C:/.../wei...F608.raw | not available | raw | Unknown | Unknown | 0 | | C:/.../wei...F609.raw | not available | raw | Unknown | Unknown | 0 | | C:/.../wei...F610.raw | not available | raw | Unknown | Unknown | 0 | | C:/.../wei...F611.raw | not available | raw | Unknown | Unknown | 0 | | C:/.../wei...F612.raw | not available | raw | Unknown | Unknown | 0 | | C:/.../wei...F613.raw | not available | raw | Unknown | Unknown | 0 | | C:/.../wei...F614.raw | not available | raw | Unknown | Unknown | 0 | | C:/.../wei...F615.raw | not available | raw | Unknown | Unknown | 0 | | C:/.../wei...F616.raw | not available | raw | Unknown | Unknown | 0 | | C:/.../wei...F617.raw | not available | raw | Unknown | Unknown | 0 | | C:/.../wei...F618.raw | not available | raw | Unknown | Unknown | 0 | | C:/.../wei...F619.raw | not available | raw | Unknown | Unknown | 0 | | C:/.../wei...F620.raw | not available | raw | Unknown | Unknown | 0 | | C:/.../wei...F621.raw | not available | raw | Unknown | Unknown | 0 | | C:/.../wei...F622.raw | not available | raw | Unknown | Unknown | 0 | | C:/.../wei...F623.raw | not available | raw | Unknown | Unknown | 0 | | C:/.../wei...F624.raw | not available | raw | Unknown | Unknown | 0 | | C:/.../wei...F625.raw | not available | raw | Unknown | Unknown | 0 | | C:/.../wei...F626.raw | not available | raw | Unknown | Unknown | 0 | | C:/.../wei...F627.raw | not available | raw | Unknown | Unknown | 0 | | C:/.../wei...F628.raw | not available | raw | Unknown | Unknown | 0 | | C:/.../wei...F629.raw | not available | raw | Unknown | Unknown | 0 | | C:/.../wei...F630.raw | not available | raw | Unknown | Unknown | 0 | | C:/.../wei...F631.raw | not available | raw | Unknown | Unknown | 0 | | C:/.../wei...F632.raw | not available | raw | Unknown | Unknown | 0 | | C:/.../wei...F633.raw | not available | raw | Unknown | Unknown | 0 | | C:/.../wei...F634.raw | not available | raw | Unknown | Unknown | 0 | | C:/.../wei...F635.raw | not available | raw | Unknown | Unknown | 0 | | C:/.../wei...F636.raw | not available | raw | Unknown | Unknown | 0 | | C:/.../wei...F637.raw | not available | raw | Unknown | Unknown | 0 | | C:/.../wei...F638.raw | not available | raw | Unknown | Unknown | 0 | | C:/.../wei...F639.raw | not available | raw | Unknown | Unknown | 0 | | C:/.../wei...F640.raw | not available | raw | Unknown | Unknown | 0 | | C:/.../wei...F641.raw | not available | raw | Unknown | Unknown | 0 | | C:/.../wei...F642.raw | not available | raw | Unknown | Unknown | 0 | | C:/.../wei...F643.raw | not available | raw | Unknown | Unknown | 0 | | C:/.../wei...F644.raw | not available | raw | Unknown | Unknown | 0 | | C:/.../wei...F645.raw | not available | raw | Unknown | Unknown | 0 | | C:/.../wei...F646.raw | not available | raw | Unknown | Unknown | 0 | | C:/.../wei...F647.raw | not available | raw | Unknown | Unknown | 0 | | C:/.../wei...F648.raw | not available | raw | Unknown | Unknown | 0 | | C:/.../wei...F649.raw | not available | raw | Unknown | Unknown | 0 | | C:/.../wei...F650.raw | not available | raw | Unknown | Unknown | 0 | | C:/.../wei...F651.raw | not available | raw | Unknown | Unknown | 0 | | C:/.../wei...F652.raw | not available | raw | Unknown | Unknown | 0 | | C:/.../wei...F653.raw | not available | raw | Unknown | Unknown | 0 | | C:/.../wei...F654.raw | not available | raw | Unknown | Unknown | 0 | | C:/.../wei...F655.raw | not available | raw | Unknown | Unknown | 0 | | C:/.../wei...F656.raw | not available | raw | Unknown | Unknown | 0 | | C:/.../wei...F657.raw | not available | raw | Unknown | Unknown | 0 | | C:/.../wei...F658.raw | not available | raw | Unknown | Unknown | 0 | | C:/.../wei...F659.raw | not available | raw | Unknown | Unknown | 0 | | C:/.../wei...F660.raw | not available | raw | Unknown | Unknown | 0 | | C:/.../wei...F661.raw | not available | raw | Unknown | Unknown | 0 | | C:/.../wei...F662.raw | not available | raw | Unknown | Unknown | 0 | | C:/.../wei...F663.raw | not available | raw | Unknown | Unknown | 0 | | C:/.../wei...F664.raw | not available | raw | Unknown | Unknown | 0 | | C:/.../wei...F665.raw | not available | raw | Unknown | Unknown | 0 | | C:/.../wei...F666.raw | not available | raw | Unknown | Unknown | 0 | | C:/.../wei...F667.raw | not available | raw | Unknown | Unknown | 0 | | C:/.../wei...F668.raw | not available | raw | Unknown | Unknown | 0 | | C:/.../wei...F669.raw | not available | raw | Unknown | Unknown | 0 | | C:/.../wei...F670.raw | not available | raw | Unknown | Unknown | 0 | | C:/.../wei...F671.raw | not available | raw | Unknown | Unknown | 0 | | C:/.../wei...F672.raw | not available | raw | Unknown | Unknown | 0 | | C:/.../wei...F673.raw | not available | raw | Unknown | Unknown | 0 | | C:/.../wei...F674.raw | not available | raw | Unknown | Unknown | 0 | | C:/.../wei...F675.raw | not available | raw | Unknown | Unknown | 0 | | C:/.../wei...F676.raw | not available | raw | Unknown | Unknown | 0 | | C:/.../wei...F677.raw | not available | raw | Unknown | Unknown | 0 |  Scanner Manufacturer Information |  |  | | --- | --- | | Name: |  | | Adresse: |  | | Internetseite: |  | | Scanner: |  | | Scansoftware: |  |  Component Information |  |  | | --- | --- | | Beschreibung: |  | | Losnummer: |  | | Seriennummer: |  |  Scan Information |  |  | | --- | --- | | Röhrenspannung: |  | | Röhrenstromstärke: |  | | Scandauer: |  | | Rekonstruktionsdauer: |  | | Gesamtverarbeitungszeit: |  | | Rekonstruktionsalgorithmus: |  | | Scanmethode: |  | | Geometrie: |  | | Integrationszeit: |  | | Filtern: |  | | Projektionsanzahl: |  | | Datum, Zeit: |  | | Benutzer: |  |  Reconstruction Parameters NO RECONSTRUCTION PARAMETERS AVAILABLE  Import Settings |  |  |  |  | | --- | --- | --- | --- | | Source/Type: | Reconstructed Volume | | | | Name: | Vereinigung braun weiss skelett aus Volumen 1 | | | | Axes swap mode: | XYZT | | | | Mirror axes: | None | | | | Data mapping: | Ramp | | | | Data range source mapping: | -1...1 | | | | Data range destination mapping: | -1...1 | | | | Data type mapping: | Unknown | | | | Voxel skip: | 0 | 0 | 0 | | Auto region of interest: | Off | | Region of interest (min): | 0 | 0 | 0 | | Region of interest (max): | -1 | -1 | -1 | | Slice interpolation mode: | Off | | | | Slice interpolation threshold: | 0 | | | | Resolution [mm]: | 1 | 1 | 1 | | Resampling mode: | Off | | | | Auto histogram mode: | Off | | Lower auto histogram boundary at (%): | 0 | | Upper auto histogram boundary at (%): | 0 |  Analysis Information |  |  | | --- | --- | | Wall thickness: | 0 | | Defect detection: | 0 | | Nominal/actual comparison: | 0 | | Number of reference objects: | 0 |  File List (678) | files (678) | dimensions | format | type | endian | header | | --- | --- | --- | --- | --- | --- | | C:/.../bra...4000.raw | not available | raw | Unknown | Unknown | 0 | | C:/.../bra...4001.raw | not available | raw | Unknown | Unknown | 0 | | C:/.../bra...4002.raw | not available | raw | Unknown | Unknown | 0 | | C:/.../bra...4003.raw | not available | raw | Unknown | Unknown | 0 | | C:/.../bra...4004.raw | not available | raw | Unknown | Unknown | 0 | | C:/.../bra...4005.raw | not available | raw | Unknown | Unknown | 0 | | C:/.../bra...4006.raw | not available | raw | Unknown | Unknown | 0 | | C:/.../bra...4007.raw | not available | raw | Unknown | Unknown | 0 | | C:/.../bra...4008.raw | not available | raw | Unknown | Unknown | 0 | | C:/.../bra...4009.raw | not available | raw | Unknown | Unknown | 0 | | C:/.../bra...4010.raw | not available | raw | Unknown | Unknown | 0 | | C:/.../bra...4011.raw | not available | raw | Unknown | Unknown | 0 | | C:/.../bra...4012.raw | not available | raw | Unknown | Unknown | 0 | | C:/.../bra...4013.raw | not available | raw | Unknown | Unknown | 0 | | C:/.../bra...4014.raw | not available | raw | Unknown | Unknown | 0 | | C:/.../bra...4015.raw | not available | raw | Unknown | Unknown | 0 | | C:/.../bra...4016.raw | not available | raw | Unknown | Unknown | 0 | | C:/.../bra...4017.raw | not available | raw | Unknown | Unknown | 0 | | C:/.../bra...4018.raw | not available | raw | Unknown | Unknown | 0 | | C:/.../bra...4019.raw | not available | raw | Unknown | Unknown | 0 | | C:/.../bra...4020.raw | not available | raw | Unknown | Unknown | 0 | | C:/.../bra...4021.raw | not available | raw | Unknown | Unknown | 0 | | C:/.../bra...4022.raw | not available | raw | Unknown | Unknown | 0 | | C:/.../bra...4023.raw | not available | raw | Unknown | Unknown | 0 | | C:/.../bra...4024.raw | not available | raw | Unknown | Unknown | 0 | | C:/.../bra...4025.raw | not available | raw | Unknown | Unknown | 0 | | C:/.../bra...4026.raw | not available | raw | Unknown | Unknown | 0 | | C:/.../bra...4027.raw | not available | raw | Unknown | Unknown | 0 | | C:/.../bra...4028.raw | not available | raw | Unknown | Unknown | 0 | | C:/.../bra...4029.raw | not available | raw | Unknown | Unknown | 0 | | C:/.../bra...4030.raw | not available | raw | Unknown | Unknown | 0 | | C:/.../bra...4031.raw | not available | raw | Unknown | Unknown | 0 | | C:/.../bra...4032.raw | not available | raw | Unknown | Unknown | 0 | | C:/.../bra...4033.raw | not available | raw | Unknown | Unknown | 0 | | C:/.../bra...4034.raw | not available | raw | Unknown | Unknown | 0 | | C:/.../bra...4035.raw | not available | raw | Unknown | Unknown | 0 | | C:/.../bra...4036.raw | not available | raw | Unknown | Unknown | 0 | | C:/.../bra...4037.raw | not available | raw | Unknown | Unknown | 0 | | C:/.../bra...4038.raw | not available | raw | Unknown | Unknown | 0 | | C:/.../bra...4039.raw | not available | raw | Unknown | Unknown | 0 | | C:/.../bra...4040.raw | not available | raw | Unknown | Unknown | 0 | | C:/.../bra...4041.raw | not available | raw | Unknown | Unknown | 0 | | C:/.../bra...4042.raw | not available | raw | Unknown | Unknown | 0 | | C:/.../bra...4043.raw | not available | raw | Unknown | Unknown | 0 | | C:/.../bra...4044.raw | not available | raw | Unknown | Unknown | 0 | | C:/.../bra...4045.raw | not available | raw | Unknown | Unknown | 0 | | C:/.../bra...4046.raw | not available | raw | Unknown | Unknown | 0 | | C:/.../bra...4047.raw | not available | raw | Unknown | Unknown | 0 | | C:/.../bra...4048.raw | not available | raw | Unknown | Unknown | 0 | | C:/.../bra...4049.raw | not available | raw | Unknown | Unknown | 0 | | C:/.../bra...4050.raw | not available | raw | Unknown | Unknown | 0 | | C:/.../bra...4051.raw | not available | raw | Unknown | Unknown | 0 | | C:/.../bra...4052.raw | not available | raw | Unknown | Unknown | 0 | | C:/.../bra...4053.raw | not available | raw | Unknown | Unknown | 0 | | C:/.../bra...4054.raw | not available | raw | Unknown | Unknown | 0 | | C:/.../bra...4055.raw | not available | raw | Unknown | Unknown | 0 | | C:/.../bra...4056.raw | not available | raw | Unknown | Unknown | 0 | | C:/.../bra...4057.raw | not available | raw | Unknown | Unknown | 0 | | C:/.../bra...4058.raw | not available | raw | Unknown | Unknown | 0 | | C:/.../bra...4059.raw | not available | raw | Unknown | Unknown | 0 | | C:/.../bra...4060.raw | not available | raw | Unknown | Unknown | 0 | | C:/.../bra...4061.raw | not available | raw | Unknown | Unknown | 0 | | C:/.../bra...4062.raw | not available | raw | Unknown | Unknown | 0 | | C:/.../bra...4063.raw | not available | raw | Unknown | Unknown | 0 | | C:/.../bra...4064.raw | not available | raw | Unknown | Unknown | 0 | | C:/.../bra...4065.raw | not available | raw | Unknown | Unknown | 0 | | C:/.../bra...4066.raw | not available | raw | Unknown | Unknown | 0 | | C:/.../bra...4067.raw | not available | raw | Unknown | Unknown | 0 | | C:/.../bra...4068.raw | not available | raw | Unknown | Unknown | 0 | | C:/.../bra...4069.raw | not available | raw | Unknown | Unknown | 0 | | C:/.../bra...4070.raw | not available | raw | Unknown | Unknown | 0 | | C:/.../bra...4071.raw | not available | raw | Unknown | Unknown | 0 | | C:/.../bra...4072.raw | not available | raw | Unknown | Unknown | 0 | | C:/.../bra...4073.raw | not available | raw | Unknown | Unknown | 0 | | C:/.../bra...4074.raw | not available | raw | Unknown | Unknown | 0 | | C:/.../bra...4075.raw | not available | raw | Unknown | Unknown | 0 | | C:/.../bra...4076.raw | not available | raw | Unknown | Unknown | 0 | | C:/.../bra...4077.raw | not available | raw | Unknown | Unknown | 0 | | C:/.../bra...4078.raw | not available | raw | Unknown | Unknown | 0 | | C:/.../bra...4079.raw | not available | raw | Unknown | Unknown | 0 | | C:/.../bra...4080.raw | not available | raw | Unknown | Unknown | 0 | | C:/.../bra...4081.raw | not available | raw | Unknown | Unknown | 0 | | C:/.../bra...4082.raw | not available | raw | Unknown | Unknown | 0 | | C:/.../bra...4083.raw | not available | raw | Unknown | Unknown | 0 | | C:/.../bra...4084.raw | not available | raw | Unknown | Unknown | 0 | | C:/.../bra...4085.raw | not available | raw | Unknown | Unknown | 0 | | C:/.../bra...4086.raw | not available | raw | Unknown | Unknown | 0 | | C:/.../bra...4087.raw | not available | raw | Unknown | Unknown | 0 | | C:/.../bra...4088.raw | not available | raw | Unknown | Unknown | 0 | | C:/.../bra...4089.raw | not available | raw | Unknown | Unknown | 0 | | C:/.../bra...4090.raw | not available | raw | Unknown | Unknown | 0 | | C:/.../bra...4091.raw | not available | raw | Unknown | Unknown | 0 | | C:/.../bra...4092.raw | not available | raw | Unknown | Unknown | 0 | | C:/.../bra...4093.raw | not available | raw | Unknown | Unknown | 0 | | C:/.../bra...4094.raw | not available | raw | Unknown | Unknown | 0 | | C:/.../bra...4095.raw | not available | raw | Unknown | Unknown | 0 | | C:/.../bra...4096.raw | not available | raw | Unknown | Unknown | 0 | | C:/.../bra...4097.raw | not available | raw | Unknown | Unknown | 0 | | C:/.../bra...4098.raw | not available | raw | Unknown | Unknown | 0 | | C:/.../bra...4099.raw | not available | raw | Unknown | Unknown | 0 | | C:/.../bra...4100.raw | not available | raw | Unknown | Unknown | 0 | | C:/.../bra...4101.raw | not available | raw | Unknown | Unknown | 0 | | C:/.../bra...4102.raw | not available | raw | Unknown | Unknown | 0 | | C:/.../bra...4103.raw | not available | raw | Unknown | Unknown | 0 | | C:/.../bra...4104.raw | not available | raw | Unknown | Unknown | 0 | | C:/.../bra...4105.raw | not available | raw | Unknown | Unknown | 0 | | C:/.../bra...4106.raw | not available | raw | Unknown | Unknown | 0 | | C:/.../bra...4107.raw | not available | raw | Unknown | Unknown | 0 | | C:/.../bra...4108.raw | not available | raw | Unknown | Unknown | 0 | | C:/.../bra...4109.raw | not available | raw | Unknown | Unknown | 0 | | C:/.../bra...4110.raw | not available | raw | Unknown | Unknown | 0 | | C:/.../bra...4111.raw | not available | raw | Unknown | Unknown | 0 | | C:/.../bra...4112.raw | not available | raw | Unknown | Unknown | 0 | | C:/.../bra...4113.raw | not available | raw | Unknown | Unknown | 0 | | C:/.../bra...4114.raw | not available | raw | Unknown | Unknown | 0 | | C:/.../bra...4115.raw | not available | raw | Unknown | Unknown | 0 | | C:/.../bra...4116.raw | not available | raw | Unknown | Unknown | 0 | | C:/.../bra...4117.raw | not available | raw | Unknown | Unknown | 0 | | C:/.../bra...4118.raw | not available | raw | Unknown | Unknown | 0 | | C:/.../bra...4119.raw | not available | raw | Unknown | Unknown | 0 | | C:/.../bra...4120.raw | not available | raw | Unknown | Unknown | 0 | | C:/.../bra...4121.raw | not available | raw | Unknown | Unknown | 0 | | C:/.../bra...4122.raw | not available | raw | Unknown | Unknown | 0 | | C:/.../bra...4123.raw | not available | raw | Unknown | Unknown | 0 | | C:/.../bra...4124.raw | not available | raw | Unknown | Unknown | 0 | | C:/.../bra...4125.raw | not available | raw | Unknown | Unknown | 0 | | C:/.../bra...4126.raw | not available | raw | Unknown | Unknown | 0 | | C:/.../bra...4127.raw | not available | raw | Unknown | Unknown | 0 | | C:/.../bra...4128.raw | not available | raw | Unknown | Unknown | 0 | | C:/.../bra...4129.raw | not available | raw | Unknown | Unknown | 0 | | C:/.../bra...4130.raw | not available | raw | Unknown | Unknown | 0 | | C:/.../bra...4131.raw | not available | raw | Unknown | Unknown | 0 | | C:/.../bra...4132.raw | not available | raw | Unknown | Unknown | 0 | | C:/.../bra...4133.raw | not available | raw | Unknown | Unknown | 0 | | C:/.../bra...4134.raw | not available | raw | Unknown | Unknown | 0 | | C:/.../bra...4135.raw | not available | raw | Unknown | Unknown | 0 | | C:/.../bra...4136.raw | not available | raw | Unknown | Unknown | 0 | | C:/.../bra...4137.raw | not available | raw | Unknown | Unknown | 0 | | C:/.../bra...4138.raw | not available | raw | Unknown | Unknown | 0 | | C:/.../bra...4139.raw | not available | raw | Unknown | Unknown | 0 | | C:/.../bra...4140.raw | not available | raw | Unknown | Unknown | 0 | | C:/.../bra...4141.raw | not available | raw | Unknown | Unknown | 0 | | C:/.../bra...4142.raw | not available | raw | Unknown | Unknown | 0 | | C:/.../bra...4143.raw | not available | raw | Unknown | Unknown | 0 | | C:/.../bra...4144.raw | not available | raw | Unknown | Unknown | 0 | | C:/.../bra...4145.raw | not available | raw | Unknown | Unknown | 0 | | C:/.../bra...4146.raw | not available | raw | Unknown | Unknown | 0 | | C:/.../bra...4147.raw | not available | raw | Unknown | Unknown | 0 | | C:/.../bra...4148.raw | not available | raw | Unknown | Unknown | 0 | | C:/.../bra...4149.raw | not available | raw | Unknown | Unknown | 0 | | C:/.../bra...4150.raw | not available | raw | Unknown | Unknown | 0 | | C:/.../bra...4151.raw | not available | raw | Unknown | Unknown | 0 | | C:/.../bra...4152.raw | not available | raw | Unknown | Unknown | 0 | | C:/.../bra...4153.raw | not available | raw | Unknown | Unknown | 0 | | C:/.../bra...4154.raw | not available | raw | Unknown | Unknown | 0 | | C:/.../bra...4155.raw | not available | raw | Unknown | Unknown | 0 | | C:/.../bra...4156.raw | not available | raw | Unknown | Unknown | 0 | | C:/.../bra...4157.raw | not available | raw | Unknown | Unknown | 0 | | C:/.../bra...4158.raw | not available | raw | Unknown | Unknown | 0 | | C:/.../bra...4159.raw | not available | raw | Unknown | Unknown | 0 | | C:/.../bra...4160.raw | not available | raw | Unknown | Unknown | 0 | | C:/.../bra...4161.raw | not available | raw | Unknown | Unknown | 0 | | C:/.../bra...4162.raw | not available | raw | Unknown | Unknown | 0 | | C:/.../bra...4163.raw | not available | raw | Unknown | Unknown | 0 | | C:/.../bra...4164.raw | not available | raw | Unknown | Unknown | 0 | | C:/.../bra...4165.raw | not available | raw | Unknown | Unknown | 0 | | C:/.../bra...4166.raw | not available | raw | Unknown | Unknown | 0 | | C:/.../bra...4167.raw | not available | raw | Unknown | Unknown | 0 | | C:/.../bra...4168.raw | not available | raw | Unknown | Unknown | 0 | | C:/.../bra...4169.raw | not available | raw | Unknown | Unknown | 0 | | C:/.../bra...4170.raw | not available | raw | Unknown | Unknown | 0 | | C:/.../bra...4171.raw | not available | raw | Unknown | Unknown | 0 | | C:/.../bra...4172.raw | not available | raw | Unknown | Unknown | 0 | | C:/.../bra...4173.raw | not available | raw | Unknown | Unknown | 0 | | C:/.../bra...4174.raw | not available | raw | Unknown | Unknown | 0 | | C:/.../bra...4175.raw | not available | raw | Unknown | Unknown | 0 | | C:/.../bra...4176.raw | not available | raw | Unknown | Unknown | 0 | | C:/.../bra...4177.raw | not available | raw | Unknown | Unknown | 0 | | C:/.../bra...4178.raw | not available | raw | Unknown | Unknown | 0 | | C:/.../bra...4179.raw | not available | raw | Unknown | Unknown | 0 | | C:/.../bra...4180.raw | not available | raw | Unknown | Unknown | 0 | | C:/.../bra...4181.raw | not available | raw | Unknown | Unknown | 0 | | C:/.../bra...4182.raw | not available | raw | Unknown | Unknown | 0 | | C:/.../bra...4183.raw | not available | raw | Unknown | Unknown | 0 | | C:/.../bra...4184.raw | not available | raw | Unknown | Unknown | 0 | | C:/.../bra...4185.raw | not available | raw | Unknown | Unknown | 0 | | C:/.../bra...4186.raw | not available | raw | Unknown | Unknown | 0 | | C:/.../bra...4187.raw | not available | raw | Unknown | Unknown | 0 | | C:/.../bra...4188.raw | not available | raw | Unknown | Unknown | 0 | | C:/.../bra...4189.raw | not available | raw | Unknown | Unknown | 0 | | C:/.../bra...4190.raw | not available | raw | Unknown | Unknown | 0 | | C:/.../bra...4191.raw | not available | raw | Unknown | Unknown | 0 | | C:/.../bra...4192.raw | not available | raw | Unknown | Unknown | 0 | | C:/.../bra...4193.raw | not available | raw | Unknown | Unknown | 0 | | C:/.../bra...4194.raw | not available | raw | Unknown | Unknown | 0 | | C:/.../bra...4195.raw | not available | raw | Unknown | Unknown | 0 | | C:/.../bra...4196.raw | not available | raw | Unknown | Unknown | 0 | | C:/.../bra...4197.raw | not available | raw | Unknown | Unknown | 0 | | C:/.../bra...4198.raw | not available | raw | Unknown | Unknown | 0 | | C:/.../bra...4199.raw | not available | raw | Unknown | Unknown | 0 | | C:/.../bra...4200.raw | not available | raw | Unknown | Unknown | 0 | | C:/.../bra...4201.raw | not available | raw | Unknown | Unknown | 0 | | C:/.../bra...4202.raw | not available | raw | Unknown | Unknown | 0 | | C:/.../bra...4203.raw | not available | raw | Unknown | Unknown | 0 | | C:/.../bra...4204.raw | not available | raw | Unknown | Unknown | 0 | | C:/.../bra...4205.raw | not available | raw | Unknown | Unknown | 0 | | C:/.../bra...4206.raw | not available | raw | Unknown | Unknown | 0 | | C:/.../bra...4207.raw | not available | raw | Unknown | Unknown | 0 | | C:/.../bra...4208.raw | not available | raw | Unknown | Unknown | 0 | | C:/.../bra...4209.raw | not available | raw | Unknown | Unknown | 0 | | C:/.../bra...4210.raw | not available | raw | Unknown | Unknown | 0 | | C:/.../bra...4211.raw | not available | raw | Unknown | Unknown | 0 | | C:/.../bra...4212.raw | not available | raw | Unknown | Unknown | 0 | | C:/.../bra...4213.raw | not available | raw | Unknown | Unknown | 0 | | C:/.../bra...4214.raw | not available | raw | Unknown | Unknown | 0 | | C:/.../bra...4215.raw | not available | raw | Unknown | Unknown | 0 | | C:/.../bra...4216.raw | not available | raw | Unknown | Unknown | 0 | | C:/.../bra...4217.raw | not available | raw | Unknown | Unknown | 0 | | C:/.../bra...4218.raw | not available | raw | Unknown | Unknown | 0 | | C:/.../bra...4219.raw | not available | raw | Unknown | Unknown | 0 | | C:/.../bra...4220.raw | not available | raw | Unknown | Unknown | 0 | | C:/.../bra...4221.raw | not available | raw | Unknown | Unknown | 0 | | C:/.../bra...4222.raw | not available | raw | Unknown | Unknown | 0 | | C:/.../bra...4223.raw | not available | raw | Unknown | Unknown | 0 | | C:/.../bra...4224.raw | not available | raw | Unknown | Unknown | 0 | | C:/.../bra...4225.raw | not available | raw | Unknown | Unknown | 0 | | C:/.../bra...4226.raw | not available | raw | Unknown | Unknown | 0 | | C:/.../bra...4227.raw | not available | raw | Unknown | Unknown | 0 | | C:/.../bra...4228.raw | not available | raw | Unknown | Unknown | 0 | | C:/.../bra...4229.raw | not available | raw | Unknown | Unknown | 0 | | C:/.../bra...4230.raw | not available | raw | Unknown | Unknown | 0 | | C:/.../bra...4231.raw | not available | raw | Unknown | Unknown | 0 | | C:/.../bra...4232.raw | not available | raw | Unknown | Unknown | 0 | | C:/.../bra...4233.raw | not available | raw | Unknown | Unknown | 0 | | C:/.../bra...4234.raw | not available | raw | Unknown | Unknown | 0 | | C:/.../bra...4235.raw | not available | raw | Unknown | Unknown | 0 | | C:/.../bra...4236.raw | not available | raw | Unknown | Unknown | 0 | | C:/.../bra...4237.raw | not available | raw | Unknown | Unknown | 0 | | C:/.../bra...4238.raw | not available | raw | Unknown | Unknown | 0 | | C:/.../bra...4239.raw | not available | raw | Unknown | Unknown | 0 | | C:/.../bra...4240.raw | not available | raw | Unknown | Unknown | 0 | | C:/.../bra...4241.raw | not available | raw | Unknown | Unknown | 0 | | C:/.../bra...4242.raw | not available | raw | Unknown | Unknown | 0 | | C:/.../bra...4243.raw | not available | raw | Unknown | Unknown | 0 | | C:/.../bra...4244.raw | not available | raw | Unknown | Unknown | 0 | | C:/.../bra...4245.raw | not available | raw | Unknown | Unknown | 0 | | C:/.../bra...4246.raw | not available | raw | Unknown | Unknown | 0 | | C:/.../bra...4247.raw | not available | raw | Unknown | Unknown | 0 | | C:/.../bra...4248.raw | not available | raw | Unknown | Unknown | 0 | | C:/.../bra...4249.raw | not available | raw | Unknown | Unknown | 0 | | C:/.../bra...4250.raw | not available | raw | Unknown | Unknown | 0 | | C:/.../bra...4251.raw | not available | raw | Unknown | Unknown | 0 | | C:/.../bra...4252.raw | not available | raw | Unknown | Unknown | 0 | | C:/.../bra...4253.raw | not available | raw | Unknown | Unknown | 0 | | C:/.../bra...4254.raw | not available | raw | Unknown | Unknown | 0 | | C:/.../bra...4255.raw | not available | raw | Unknown | Unknown | 0 | | C:/.../bra...4256.raw | not available | raw | Unknown | Unknown | 0 | | C:/.../bra...4257.raw | not available | raw | Unknown | Unknown | 0 | | C:/.../bra...4258.raw | not available | raw | Unknown | Unknown | 0 | | C:/.../bra...4259.raw | not available | raw | Unknown | Unknown | 0 | | C:/.../bra...4260.raw | not available | raw | Unknown | Unknown | 0 | | C:/.../bra...4261.raw | not available | raw | Unknown | Unknown | 0 | | C:/.../bra...4262.raw | not available | raw | Unknown | Unknown | 0 | | C:/.../bra...4263.raw | not available | raw | Unknown | Unknown | 0 | | C:/.../bra...4264.raw | not available | raw | Unknown | Unknown | 0 | | C:/.../bra...4265.raw | not available | raw | Unknown | Unknown | 0 | | C:/.../bra...4266.raw | not available | raw | Unknown | Unknown | 0 | | C:/.../bra...4267.raw | not available | raw | Unknown | Unknown | 0 | | C:/.../bra...4268.raw | not available | raw | Unknown | Unknown | 0 | | C:/.../bra...4269.raw | not available | raw | Unknown | Unknown | 0 | | C:/.../bra...4270.raw | not available | raw | Unknown | Unknown | 0 | | C:/.../bra...4271.raw | not available | raw | Unknown | Unknown | 0 | | C:/.../bra...4272.raw | not available | raw | Unknown | Unknown | 0 | | C:/.../bra...4273.raw | not available | raw | Unknown | Unknown | 0 | | C:/.../bra...4274.raw | not available | raw | Unknown | Unknown | 0 | | C:/.../bra...4275.raw | not available | raw | Unknown | Unknown | 0 | | C:/.../bra...4276.raw | not available | raw | Unknown | Unknown | 0 | | C:/.../bra...4277.raw | not available | raw | Unknown | Unknown | 0 | | C:/.../bra...4278.raw | not available | raw | Unknown | Unknown | 0 | | C:/.../bra...4279.raw | not available | raw | Unknown | Unknown | 0 | | C:/.../bra...4280.raw | not available | raw | Unknown | Unknown | 0 | | C:/.../bra...4281.raw | not available | raw | Unknown | Unknown | 0 | | C:/.../bra...4282.raw | not available | raw | Unknown | Unknown | 0 | | C:/.../bra...4283.raw | not available | raw | Unknown | Unknown | 0 | | C:/.../bra...4284.raw | not available | raw | Unknown | Unknown | 0 | | C:/.../bra...4285.raw | not available | raw | Unknown | Unknown | 0 | | C:/.../bra...4286.raw | not available | raw | Unknown | Unknown | 0 | | C:/.../bra...4287.raw | not available | raw | Unknown | Unknown | 0 | | C:/.../bra...4288.raw | not available | raw | Unknown | Unknown | 0 | | C:/.../bra...4289.raw | not available | raw | Unknown | Unknown | 0 | | C:/.../bra...4290.raw | not available | raw | Unknown | Unknown | 0 | | C:/.../bra...4291.raw | not available | raw | Unknown | Unknown | 0 | | C:/.../bra...4292.raw | not available | raw | Unknown | Unknown | 0 | | C:/.../bra...4293.raw | not available | raw | Unknown | Unknown | 0 | | C:/.../bra...4294.raw | not available | raw | Unknown | Unknown | 0 | | C:/.../bra...4295.raw | not available | raw | Unknown | Unknown | 0 | | C:/.../bra...4296.raw | not available | raw | Unknown | Unknown | 0 | | C:/.../bra...4297.raw | not available | raw | Unknown | Unknown | 0 | | C:/.../bra...4298.raw | not available | raw | Unknown | Unknown | 0 | | C:/.../bra...4299.raw | not available | raw | Unknown | Unknown | 0 | | C:/.../bra...4300.raw | not available | raw | Unknown | Unknown | 0 | | C:/.../bra...4301.raw | not available | raw | Unknown | Unknown | 0 | | C:/.../bra...4302.raw | not available | raw | Unknown | Unknown | 0 | | C:/.../bra...4303.raw | not available | raw | Unknown | Unknown | 0 | | C:/.../bra...4304.raw | not available | raw | Unknown | Unknown | 0 | | C:/.../bra...4305.raw | not available | raw | Unknown | Unknown | 0 | | C:/.../bra...4306.raw | not available | raw | Unknown | Unknown | 0 | | C:/.../bra...4307.raw | not available | raw | Unknown | Unknown | 0 | | C:/.../bra...4308.raw | not available | raw | Unknown | Unknown | 0 | | C:/.../bra...4309.raw | not available | raw | Unknown | Unknown | 0 | | C:/.../bra...4310.raw | not available | raw | Unknown | Unknown | 0 | | C:/.../bra...4311.raw | not available | raw | Unknown | Unknown | 0 | | C:/.../bra...4312.raw | not available | raw | Unknown | Unknown | 0 | | C:/.../bra...4313.raw | not available | raw | Unknown | Unknown | 0 | | C:/.../bra...4314.raw | not available | raw | Unknown | Unknown | 0 | | C:/.../bra...4315.raw | not available | raw | Unknown | Unknown | 0 | | C:/.../bra...4316.raw | not available | raw | Unknown | Unknown | 0 | | C:/.../bra...4317.raw | not available | raw | Unknown | Unknown | 0 | | C:/.../bra...4318.raw | not available | raw | Unknown | Unknown | 0 | | C:/.../bra...4319.raw | not available | raw | Unknown | Unknown | 0 | | C:/.../bra...4320.raw | not available | raw | Unknown | Unknown | 0 | | C:/.../bra...4321.raw | not available | raw | Unknown | Unknown | 0 | | C:/.../bra...4322.raw | not available | raw | Unknown | Unknown | 0 | | C:/.../bra...4323.raw | not available | raw | Unknown | Unknown | 0 | | C:/.../bra...4324.raw | not available | raw | Unknown | Unknown | 0 | | C:/.../bra...4325.raw | not available | raw | Unknown | Unknown | 0 | | C:/.../bra...4326.raw | not available | raw | Unknown | Unknown | 0 | | C:/.../bra...4327.raw | not available | raw | Unknown | Unknown | 0 | | C:/.../bra...4328.raw | not available | raw | Unknown | Unknown | 0 | | C:/.../bra...4329.raw | not available | raw | Unknown | Unknown | 0 | | C:/.../bra...4330.raw | not available | raw | Unknown | Unknown | 0 | | C:/.../bra...4331.raw | not available | raw | Unknown | Unknown | 0 | | C:/.../bra...4332.raw | not available | raw | Unknown | Unknown | 0 | | C:/.../bra...4333.raw | not available | raw | Unknown | Unknown | 0 | | C:/.../bra...4334.raw | not available | raw | Unknown | Unknown | 0 | | C:/.../bra...4335.raw | not available | raw | Unknown | Unknown | 0 | | C:/.../bra...4336.raw | not available | raw | Unknown | Unknown | 0 | | C:/.../bra...4337.raw | not available | raw | Unknown | Unknown | 0 | | C:/.../bra...4338.raw | not available | raw | Unknown | Unknown | 0 | | C:/.../bra...4339.raw | not available | raw | Unknown | Unknown | 0 | | C:/.../bra...4340.raw | not available | raw | Unknown | Unknown | 0 | | C:/.../bra...4341.raw | not available | raw | Unknown | Unknown | 0 | | C:/.../bra...4342.raw | not available | raw | Unknown | Unknown | 0 | | C:/.../bra...4343.raw | not available | raw | Unknown | Unknown | 0 | | C:/.../bra...4344.raw | not available | raw | Unknown | Unknown | 0 | | C:/.../bra...4345.raw | not available | raw | Unknown | Unknown | 0 | | C:/.../bra...4346.raw | not available | raw | Unknown | Unknown | 0 | | C:/.../bra...4347.raw | not available | raw | Unknown | Unknown | 0 | | C:/.../bra...4348.raw | not available | raw | Unknown | Unknown | 0 | | C:/.../bra...4349.raw | not available | raw | Unknown | Unknown | 0 | | C:/.../bra...4350.raw | not available | raw | Unknown | Unknown | 0 | | C:/.../bra...4351.raw | not available | raw | Unknown | Unknown | 0 | | C:/.../bra...4352.raw | not available | raw | Unknown | Unknown | 0 | | C:/.../bra...4353.raw | not available | raw | Unknown | Unknown | 0 | | C:/.../bra...4354.raw | not available | raw | Unknown | Unknown | 0 | | C:/.../bra...4355.raw | not available | raw | Unknown | Unknown | 0 | | C:/.../bra...4356.raw | not available | raw | Unknown | Unknown | 0 | | C:/.../bra...4357.raw | not available | raw | Unknown | Unknown | 0 | | C:/.../bra...4358.raw | not available | raw | Unknown | Unknown | 0 | | C:/.../bra...4359.raw | not available | raw | Unknown | Unknown | 0 | | C:/.../bra...4360.raw | not available | raw | Unknown | Unknown | 0 | | C:/.../bra...4361.raw | not available | raw | Unknown | Unknown | 0 | | C:/.../bra...4362.raw | not available | raw | Unknown | Unknown | 0 | | C:/.../bra...4363.raw | not available | raw | Unknown | Unknown | 0 | | C:/.../bra...4364.raw | not available | raw | Unknown | Unknown | 0 | | C:/.../bra...4365.raw | not available | raw | Unknown | Unknown | 0 | | C:/.../bra...4366.raw | not available | raw | Unknown | Unknown | 0 | | C:/.../bra...4367.raw | not available | raw | Unknown | Unknown | 0 | | C:/.../bra...4368.raw | not available | raw | Unknown | Unknown | 0 | | C:/.../bra...4369.raw | not available | raw | Unknown | Unknown | 0 | | C:/.../bra...4370.raw | not available | raw | Unknown | Unknown | 0 | | C:/.../bra...4371.raw | not available | raw | Unknown | Unknown | 0 | | C:/.../bra...4372.raw | not available | raw | Unknown | Unknown | 0 | | C:/.../bra...4373.raw | not available | raw | Unknown | Unknown | 0 | | C:/.../bra...4374.raw | not available | raw | Unknown | Unknown | 0 | | C:/.../bra...4375.raw | not available | raw | Unknown | Unknown | 0 | | C:/.../bra...4376.raw | not available | raw | Unknown | Unknown | 0 | | C:/.../bra...4377.raw | not available | raw | Unknown | Unknown | 0 | | C:/.../bra...4378.raw | not available | raw | Unknown | Unknown | 0 | | C:/.../bra...4379.raw | not available | raw | Unknown | Unknown | 0 | | C:/.../bra...4380.raw | not available | raw | Unknown | Unknown | 0 | | C:/.../bra...4381.raw | not available | raw | Unknown | Unknown | 0 | | C:/.../bra...4382.raw | not available | raw | Unknown | Unknown | 0 | | C:/.../bra...4383.raw | not available | raw | Unknown | Unknown | 0 | | C:/.../bra...4384.raw | not available | raw | Unknown | Unknown | 0 | | C:/.../bra...4385.raw | not available | raw | Unknown | Unknown | 0 | | C:/.../bra...4386.raw | not available | raw | Unknown | Unknown | 0 | | C:/.../bra...4387.raw | not available | raw | Unknown | Unknown | 0 | | C:/.../bra...4388.raw | not available | raw | Unknown | Unknown | 0 | | C:/.../bra...4389.raw | not available | raw | Unknown | Unknown | 0 | | C:/.../bra...4390.raw | not available | raw | Unknown | Unknown | 0 | | C:/.../bra...4391.raw | not available | raw | Unknown | Unknown | 0 | | C:/.../bra...4392.raw | not available | raw | Unknown | Unknown | 0 | | C:/.../bra...4393.raw | not available | raw | Unknown | Unknown | 0 | | C:/.../bra...4394.raw | not available | raw | Unknown | Unknown | 0 | | C:/.../bra...4395.raw | not available | raw | Unknown | Unknown | 0 | | C:/.../bra...4396.raw | not available | raw | Unknown | Unknown | 0 | | C:/.../bra...4397.raw | not available | raw | Unknown | Unknown | 0 | | C:/.../bra...4398.raw | not available | raw | Unknown | Unknown | 0 | | C:/.../bra...4399.raw | not available | raw | Unknown | Unknown | 0 | | C:/.../bra...4400.raw | not available | raw | Unknown | Unknown | 0 | | C:/.../bra...4401.raw | not available | raw | Unknown | Unknown | 0 | | C:/.../bra...4402.raw | not available | raw | Unknown | Unknown | 0 | | C:/.../bra...4403.raw | not available | raw | Unknown | Unknown | 0 | | C:/.../bra...4404.raw | not available | raw | Unknown | Unknown | 0 | | C:/.../bra...4405.raw | not available | raw | Unknown | Unknown | 0 | | C:/.../bra...4406.raw | not available | raw | Unknown | Unknown | 0 | | C:/.../bra...4407.raw | not available | raw | Unknown | Unknown | 0 | | C:/.../bra...4408.raw | not available | raw | Unknown | Unknown | 0 | | C:/.../bra...4409.raw | not available | raw | Unknown | Unknown | 0 | | C:/.../bra...4410.raw | not available | raw | Unknown | Unknown | 0 | | C:/.../bra...4411.raw | not available | raw | Unknown | Unknown | 0 | | C:/.../bra...4412.raw | not available | raw | Unknown | Unknown | 0 | | C:/.../bra...4413.raw | not available | raw | Unknown | Unknown | 0 | | C:/.../bra...4414.raw | not available | raw | Unknown | Unknown | 0 | | C:/.../bra...4415.raw | not available | raw | Unknown | Unknown | 0 | | C:/.../bra...4416.raw | not available | raw | Unknown | Unknown | 0 | | C:/.../bra...4417.raw | not available | raw | Unknown | Unknown | 0 | | C:/.../bra...4418.raw | not available | raw | Unknown | Unknown | 0 | | C:/.../bra...4419.raw | not available | raw | Unknown | Unknown | 0 | | C:/.../bra...4420.raw | not available | raw | Unknown | Unknown | 0 | | C:/.../bra...4421.raw | not available | raw | Unknown | Unknown | 0 | | C:/.../bra...4422.raw | not available | raw | Unknown | Unknown | 0 | | C:/.../bra...4423.raw | not available | raw | Unknown | Unknown | 0 | | C:/.../bra...4424.raw | not available | raw | Unknown | Unknown | 0 | | C:/.../bra...4425.raw | not available | raw | Unknown | Unknown | 0 | | C:/.../bra...4426.raw | not available | raw | Unknown | Unknown | 0 | | C:/.../bra...4427.raw | not available | raw | Unknown | Unknown | 0 | | C:/.../bra...4428.raw | not available | raw | Unknown | Unknown | 0 | | C:/.../bra...4429.raw | not available | raw | Unknown | Unknown | 0 | | C:/.../bra...4430.raw | not available | raw | Unknown | Unknown | 0 | | C:/.../bra...4431.raw | not available | raw | Unknown | Unknown | 0 | | C:/.../bra...4432.raw | not available | raw | Unknown | Unknown | 0 | | C:/.../bra...4433.raw | not available | raw | Unknown | Unknown | 0 | | C:/.../bra...4434.raw | not available | raw | Unknown | Unknown | 0 | | C:/.../bra...4435.raw | not available | raw | Unknown | Unknown | 0 | | C:/.../bra...4436.raw | not available | raw | Unknown | Unknown | 0 | | C:/.../bra...4437.raw | not available | raw | Unknown | Unknown | 0 | | C:/.../bra...4438.raw | not available | raw | Unknown | Unknown | 0 | | C:/.../bra...4439.raw | not available | raw | Unknown | Unknown | 0 | | C:/.../bra...4440.raw | not available | raw | Unknown | Unknown | 0 | | C:/.../bra...4441.raw | not available | raw | Unknown | Unknown | 0 | | C:/.../bra...4442.raw | not available | raw | Unknown | Unknown | 0 | | C:/.../bra...4443.raw | not available | raw | Unknown | Unknown | 0 | | C:/.../bra...4444.raw | not available | raw | Unknown | Unknown | 0 | | C:/.../bra...4445.raw | not available | raw | Unknown | Unknown | 0 | | C:/.../bra...4446.raw | not available | raw | Unknown | Unknown | 0 | | C:/.../bra...4447.raw | not available | raw | Unknown | Unknown | 0 | | C:/.../bra...4448.raw | not available | raw | Unknown | Unknown | 0 | | C:/.../bra...4449.raw | not available | raw | Unknown | Unknown | 0 | | C:/.../bra...4450.raw | not available | raw | Unknown | Unknown | 0 | | C:/.../bra...4451.raw | not available | raw | Unknown | Unknown | 0 | | C:/.../bra...4452.raw | not available | raw | Unknown | Unknown | 0 | | C:/.../bra...4453.raw | not available | raw | Unknown | Unknown | 0 | | C:/.../bra...4454.raw | not available | raw | Unknown | Unknown | 0 | | C:/.../bra...4455.raw | not available | raw | Unknown | Unknown | 0 | | C:/.../bra...4456.raw | not available | raw | Unknown | Unknown | 0 | | C:/.../bra...4457.raw | not available | raw | Unknown | Unknown | 0 | | C:/.../bra...4458.raw | not available | raw | Unknown | Unknown | 0 | | C:/.../bra...4459.raw | not available | raw | Unknown | Unknown | 0 | | C:/.../bra...4460.raw | not available | raw | Unknown | Unknown | 0 | | C:/.../bra...4461.raw | not available | raw | Unknown | Unknown | 0 | | C:/.../bra...4462.raw | not available | raw | Unknown | Unknown | 0 | | C:/.../bra...4463.raw | not available | raw | Unknown | Unknown | 0 | | C:/.../bra...4464.raw | not available | raw | Unknown | Unknown | 0 | | C:/.../bra...4465.raw | not available | raw | Unknown | Unknown | 0 | | C:/.../bra...4466.raw | not available | raw | Unknown | Unknown | 0 | | C:/.../bra...4467.raw | not available | raw | Unknown | Unknown | 0 | | C:/.../bra...4468.raw | not available | raw | Unknown | Unknown | 0 | | C:/.../bra...4469.raw | not available | raw | Unknown | Unknown | 0 | | C:/.../bra...4470.raw | not available | raw | Unknown | Unknown | 0 | | C:/.../bra...4471.raw | not available | raw | Unknown | Unknown | 0 | | C:/.../bra...4472.raw | not available | raw | Unknown | Unknown | 0 | | C:/.../bra...4473.raw | not available | raw | Unknown | Unknown | 0 | | C:/.../bra...4474.raw | not available | raw | Unknown | Unknown | 0 | | C:/.../bra...4475.raw | not available | raw | Unknown | Unknown | 0 | | C:/.../bra...4476.raw | not available | raw | Unknown | Unknown | 0 | | C:/.../bra...4477.raw | not available | raw | Unknown | Unknown | 0 | | C:/.../bra...4478.raw | not available | raw | Unknown | Unknown | 0 | | C:/.../bra...4479.raw | not available | raw | Unknown | Unknown | 0 | | C:/.../bra...4480.raw | not available | raw | Unknown | Unknown | 0 | | C:/.../bra...4481.raw | not available | raw | Unknown | Unknown | 0 | | C:/.../bra...4482.raw | not available | raw | Unknown | Unknown | 0 | | C:/.../bra...4483.raw | not available | raw | Unknown | Unknown | 0 | | C:/.../bra...4484.raw | not available | raw | Unknown | Unknown | 0 | | C:/.../bra...4485.raw | not available | raw | Unknown | Unknown | 0 | | C:/.../bra...4486.raw | not available | raw | Unknown | Unknown | 0 | | C:/.../bra...4487.raw | not available | raw | Unknown | Unknown | 0 | | C:/.../bra...4488.raw | not available | raw | Unknown | Unknown | 0 | | C:/.../bra...4489.raw | not available | raw | Unknown | Unknown | 0 | | C:/.../bra...4490.raw | not available | raw | Unknown | Unknown | 0 | | C:/.../bra...4491.raw | not available | raw | Unknown | Unknown | 0 | | C:/.../bra...4492.raw | not available | raw | Unknown | Unknown | 0 | | C:/.../bra...4493.raw | not available | raw | Unknown | Unknown | 0 | | C:/.../bra...4494.raw | not available | raw | Unknown | Unknown | 0 | | C:/.../bra...4495.raw | not available | raw | Unknown | Unknown | 0 | | C:/.../bra...4496.raw | not available | raw | Unknown | Unknown | 0 | | C:/.../bra...4497.raw | not available | raw | Unknown | Unknown | 0 | | C:/.../bra...4498.raw | not available | raw | Unknown | Unknown | 0 | | C:/.../bra...4499.raw | not available | raw | Unknown | Unknown | 0 | | C:/.../bra...4500.raw | not available | raw | Unknown | Unknown | 0 | | C:/.../bra...4501.raw | not available | raw | Unknown | Unknown | 0 | | C:/.../bra...4502.raw | not available | raw | Unknown | Unknown | 0 | | C:/.../bra...4503.raw | not available | raw | Unknown | Unknown | 0 | | C:/.../bra...4504.raw | not available | raw | Unknown | Unknown | 0 | | C:/.../bra...4505.raw | not available | raw | Unknown | Unknown | 0 | | C:/.../bra...4506.raw | not available | raw | Unknown | Unknown | 0 | | C:/.../bra...4507.raw | not available | raw | Unknown | Unknown | 0 | | C:/.../bra...4508.raw | not available | raw | Unknown | Unknown | 0 | | C:/.../bra...4509.raw | not available | raw | Unknown | Unknown | 0 | | C:/.../bra...4510.raw | not available | raw | Unknown | Unknown | 0 | | C:/.../bra...4511.raw | not available | raw | Unknown | Unknown | 0 | | C:/.../bra...4512.raw | not available | raw | Unknown | Unknown | 0 | | C:/.../bra...4513.raw | not available | raw | Unknown | Unknown | 0 | | C:/.../bra...4514.raw | not available | raw | Unknown | Unknown | 0 | | C:/.../bra...4515.raw | not available | raw | Unknown | Unknown | 0 | | C:/.../bra...4516.raw | not available | raw | Unknown | Unknown | 0 | | C:/.../bra...4517.raw | not available | raw | Unknown | Unknown | 0 | | C:/.../bra...4518.raw | not available | raw | Unknown | Unknown | 0 | | C:/.../bra...4519.raw | not available | raw | Unknown | Unknown | 0 | | C:/.../bra...4520.raw | not available | raw | Unknown | Unknown | 0 | | C:/.../bra...4521.raw | not available | raw | Unknown | Unknown | 0 | | C:/.../bra...4522.raw | not available | raw | Unknown | Unknown | 0 | | C:/.../bra...4523.raw | not available | raw | Unknown | Unknown | 0 | | C:/.../bra...4524.raw | not available | raw | Unknown | Unknown | 0 | | C:/.../bra...4525.raw | not available | raw | Unknown | Unknown | 0 | | C:/.../bra...4526.raw | not available | raw | Unknown | Unknown | 0 | | C:/.../bra...4527.raw | not available | raw | Unknown | Unknown | 0 | | C:/.../bra...4528.raw | not available | raw | Unknown | Unknown | 0 | | C:/.../bra...4529.raw | not available | raw | Unknown | Unknown | 0 | | C:/.../bra...4530.raw | not available | raw | Unknown | Unknown | 0 | | C:/.../bra...4531.raw | not available | raw | Unknown | Unknown | 0 | | C:/.../bra...4532.raw | not available | raw | Unknown | Unknown | 0 | | C:/.../bra...4533.raw | not available | raw | Unknown | Unknown | 0 | | C:/.../bra...4534.raw | not available | raw | Unknown | Unknown | 0 | | C:/.../bra...4535.raw | not available | raw | Unknown | Unknown | 0 | | C:/.../bra...4536.raw | not available | raw | Unknown | Unknown | 0 | | C:/.../bra...4537.raw | not available | raw | Unknown | Unknown | 0 | | C:/.../bra...4538.raw | not available | raw | Unknown | Unknown | 0 | | C:/.../bra...4539.raw | not available | raw | Unknown | Unknown | 0 | | C:/.../bra...4540.raw | not available | raw | Unknown | Unknown | 0 | | C:/.../bra...4541.raw | not available | raw | Unknown | Unknown | 0 | | C:/.../bra...4542.raw | not available | raw | Unknown | Unknown | 0 | | C:/.../bra...4543.raw | not available | raw | Unknown | Unknown | 0 | | C:/.../bra...4544.raw | not available | raw | Unknown | Unknown | 0 | | C:/.../bra...4545.raw | not available | raw | Unknown | Unknown | 0 | | C:/.../bra...4546.raw | not available | raw | Unknown | Unknown | 0 | | C:/.../bra...4547.raw | not available | raw | Unknown | Unknown | 0 | | C:/.../bra...4548.raw | not available | raw | Unknown | Unknown | 0 | | C:/.../bra...4549.raw | not available | raw | Unknown | Unknown | 0 | | C:/.../bra...4550.raw | not available | raw | Unknown | Unknown | 0 | | C:/.../bra...4551.raw | not available | raw | Unknown | Unknown | 0 | | C:/.../bra...4552.raw | not available | raw | Unknown | Unknown | 0 | | C:/.../bra...4553.raw | not available | raw | Unknown | Unknown | 0 | | C:/.../bra...4554.raw | not available | raw | Unknown | Unknown | 0 | | C:/.../bra...4555.raw | not available | raw | Unknown | Unknown | 0 | | C:/.../bra...4556.raw | not available | raw | Unknown | Unknown | 0 | | C:/.../bra...4557.raw | not available | raw | Unknown | Unknown | 0 | | C:/.../bra...4558.raw | not available | raw | Unknown | Unknown | 0 | | C:/.../bra...4559.raw | not available | raw | Unknown | Unknown | 0 | | C:/.../bra...4560.raw | not available | raw | Unknown | Unknown | 0 | | C:/.../bra...4561.raw | not available | raw | Unknown | Unknown | 0 | | C:/.../bra...4562.raw | not available | raw | Unknown | Unknown | 0 | | C:/.../bra...4563.raw | not available | raw | Unknown | Unknown | 0 | | C:/.../bra...4564.raw | not available | raw | Unknown | Unknown | 0 | | C:/.../bra...4565.raw | not available | raw | Unknown | Unknown | 0 | | C:/.../bra...4566.raw | not available | raw | Unknown | Unknown | 0 | | C:/.../bra...4567.raw | not available | raw | Unknown | Unknown | 0 | | C:/.../bra...4568.raw | not available | raw | Unknown | Unknown | 0 | | C:/.../bra...4569.raw | not available | raw | Unknown | Unknown | 0 | | C:/.../bra...4570.raw | not available | raw | Unknown | Unknown | 0 | | C:/.../bra...4571.raw | not available | raw | Unknown | Unknown | 0 | | C:/.../bra...4572.raw | not available | raw | Unknown | Unknown | 0 | | C:/.../bra...4573.raw | not available | raw | Unknown | Unknown | 0 | | C:/.../bra...4574.raw | not available | raw | Unknown | Unknown | 0 | | C:/.../bra...4575.raw | not available | raw | Unknown | Unknown | 0 | | C:/.../bra...4576.raw | not available | raw | Unknown | Unknown | 0 | | C:/.../bra...4577.raw | not available | raw | Unknown | Unknown | 0 | | C:/.../bra...4578.raw | not available | raw | Unknown | Unknown | 0 | | C:/.../bra...4579.raw | not available | raw | Unknown | Unknown | 0 | | C:/.../bra...4580.raw | not available | raw | Unknown | Unknown | 0 | | C:/.../bra...4581.raw | not available | raw | Unknown | Unknown | 0 | | C:/.../bra...4582.raw | not available | raw | Unknown | Unknown | 0 | | C:/.../bra...4583.raw | not available | raw | Unknown | Unknown | 0 | | C:/.../bra...4584.raw | not available | raw | Unknown | Unknown | 0 | | C:/.../bra...4585.raw | not available | raw | Unknown | Unknown | 0 | | C:/.../bra...4586.raw | not available | raw | Unknown | Unknown | 0 | | C:/.../bra...4587.raw | not available | raw | Unknown | Unknown | 0 | | C:/.../bra...4588.raw | not available | raw | Unknown | Unknown | 0 | | C:/.../bra...4589.raw | not available | raw | Unknown | Unknown | 0 | | C:/.../bra...4590.raw | not available | raw | Unknown | Unknown | 0 | | C:/.../bra...4591.raw | not available | raw | Unknown | Unknown | 0 | | C:/.../bra...4592.raw | not available | raw | Unknown | Unknown | 0 | | C:/.../bra...4593.raw | not available | raw | Unknown | Unknown | 0 | | C:/.../bra...4594.raw | not available | raw | Unknown | Unknown | 0 | | C:/.../bra...4595.raw | not available | raw | Unknown | Unknown | 0 | | C:/.../bra...4596.raw | not available | raw | Unknown | Unknown | 0 | | C:/.../bra...4597.raw | not available | raw | Unknown | Unknown | 0 | | C:/.../bra...4598.raw | not available | raw | Unknown | Unknown | 0 | | C:/.../bra...4599.raw | not available | raw | Unknown | Unknown | 0 | | C:/.../bra...4600.raw | not available | raw | Unknown | Unknown | 0 | | C:/.../bra...4601.raw | not available | raw | Unknown | Unknown | 0 | | C:/.../bra...4602.raw | not available | raw | Unknown | Unknown | 0 | | C:/.../bra...4603.raw | not available | raw | Unknown | Unknown | 0 | | C:/.../bra...4604.raw | not available | raw | Unknown | Unknown | 0 | | C:/.../bra...4605.raw | not available | raw | Unknown | Unknown | 0 | | C:/.../bra...4606.raw | not available | raw | Unknown | Unknown | 0 | | C:/.../bra...4607.raw | not available | raw | Unknown | Unknown | 0 | | C:/.../bra...4608.raw | not available | raw | Unknown | Unknown | 0 | | C:/.../bra...4609.raw | not available | raw | Unknown | Unknown | 0 | | C:/.../bra...4610.raw | not available | raw | Unknown | Unknown | 0 | | C:/.../bra...4611.raw | not available | raw | Unknown | Unknown | 0 | | C:/.../bra...4612.raw | not available | raw | Unknown | Unknown | 0 | | C:/.../bra...4613.raw | not available | raw | Unknown | Unknown | 0 | | C:/.../bra...4614.raw | not available | raw | Unknown | Unknown | 0 | | C:/.../bra...4615.raw | not available | raw | Unknown | Unknown | 0 | | C:/.../bra...4616.raw | not available | raw | Unknown | Unknown | 0 | | C:/.../bra...4617.raw | not available | raw | Unknown | Unknown | 0 | | C:/.../bra...4618.raw | not available | raw | Unknown | Unknown | 0 | | C:/.../bra...4619.raw | not available | raw | Unknown | Unknown | 0 | | C:/.../bra...4620.raw | not available | raw | Unknown | Unknown | 0 | | C:/.../bra...4621.raw | not available | raw | Unknown | Unknown | 0 | | C:/.../bra...4622.raw | not available | raw | Unknown | Unknown | 0 | | C:/.../bra...4623.raw | not available | raw | Unknown | Unknown | 0 | | C:/.../bra...4624.raw | not available | raw | Unknown | Unknown | 0 | | C:/.../bra...4625.raw | not available | raw | Unknown | Unknown | 0 | | C:/.../bra...4626.raw | not available | raw | Unknown | Unknown | 0 | | C:/.../bra...4627.raw | not available | raw | Unknown | Unknown | 0 | | C:/.../bra...4628.raw | not available | raw | Unknown | Unknown | 0 | | C:/.../bra...4629.raw | not available | raw | Unknown | Unknown | 0 | | C:/.../bra...4630.raw | not available | raw | Unknown | Unknown | 0 | | C:/.../bra...4631.raw | not available | raw | Unknown | Unknown | 0 | | C:/.../bra...4632.raw | not available | raw | Unknown | Unknown | 0 | | C:/.../bra...4633.raw | not available | raw | Unknown | Unknown | 0 | | C:/.../bra...4634.raw | not available | raw | Unknown | Unknown | 0 | | C:/.../bra...4635.raw | not available | raw | Unknown | Unknown | 0 | | C:/.../bra...4636.raw | not available | raw | Unknown | Unknown | 0 | | C:/.../bra...4637.raw | not available | raw | Unknown | Unknown | 0 | | C:/.../bra...4638.raw | not available | raw | Unknown | Unknown | 0 | | C:/.../bra...4639.raw | not available | raw | Unknown | Unknown | 0 | | C:/.../bra...4640.raw | not available | raw | Unknown | Unknown | 0 | | C:/.../bra...4641.raw | not available | raw | Unknown | Unknown | 0 | | C:/.../bra...4642.raw | not available | raw | Unknown | Unknown | 0 | | C:/.../bra...4643.raw | not available | raw | Unknown | Unknown | 0 | | C:/.../bra...4644.raw | not available | raw | Unknown | Unknown | 0 | | C:/.../bra...4645.raw | not available | raw | Unknown | Unknown | 0 | | C:/.../bra...4646.raw | not available | raw | Unknown | Unknown | 0 | | C:/.../bra...4647.raw | not available | raw | Unknown | Unknown | 0 | | C:/.../bra...4648.raw | not available | raw | Unknown | Unknown | 0 | | C:/.../bra...4649.raw | not available | raw | Unknown | Unknown | 0 | | C:/.../bra...4650.raw | not available | raw | Unknown | Unknown | 0 | | C:/.../bra...4651.raw | not available | raw | Unknown | Unknown | 0 | | C:/.../bra...4652.raw | not available | raw | Unknown | Unknown | 0 | | C:/.../bra...4653.raw | not available | raw | Unknown | Unknown | 0 | | C:/.../bra...4654.raw | not available | raw | Unknown | Unknown | 0 | | C:/.../bra...4655.raw | not available | raw | Unknown | Unknown | 0 | | C:/.../bra...4656.raw | not available | raw | Unknown | Unknown | 0 | | C:/.../bra...4657.raw | not available | raw | Unknown | Unknown | 0 | | C:/.../bra...4658.raw | not available | raw | Unknown | Unknown | 0 | | C:/.../bra...4659.raw | not available | raw | Unknown | Unknown | 0 | | C:/.../bra...4660.raw | not available | raw | Unknown | Unknown | 0 | | C:/.../bra...4661.raw | not available | raw | Unknown | Unknown | 0 | | C:/.../bra...4662.raw | not available | raw | Unknown | Unknown | 0 | | C:/.../bra...4663.raw | not available | raw | Unknown | Unknown | 0 | | C:/.../bra...4664.raw | not available | raw | Unknown | Unknown | 0 | | C:/.../bra...4665.raw | not available | raw | Unknown | Unknown | 0 | | C:/.../bra...4666.raw | not available | raw | Unknown | Unknown | 0 | | C:/.../bra...4667.raw | not available | raw | Unknown | Unknown | 0 | | C:/.../bra...4668.raw | not available | raw | Unknown | Unknown | 0 | | C:/.../bra...4669.raw | not available | raw | Unknown | Unknown | 0 | | C:/.../bra...4670.raw | not available | raw | Unknown | Unknown | 0 | | C:/.../bra...4671.raw | not available | raw | Unknown | Unknown | 0 | | C:/.../bra...4672.raw | not available | raw | Unknown | Unknown | 0 | | C:/.../bra...4673.raw | not available | raw | Unknown | Unknown | 0 | | C:/.../bra...4674.raw | not available | raw | Unknown | Unknown | 0 | | C:/.../bra...4675.raw | not available | raw | Unknown | Unknown | 0 | | C:/.../bra...4676.raw | not available | raw | Unknown | Unknown | 0 | | C:/.../bra...4677.raw | not available | raw | Unknown | Unknown | 0 |  Scanner Manufacturer Information |  |  | | --- | --- | | Name: |  | | Adresse: |  | | Internetseite: |  | | Scanner: |  | | Scansoftware: |  |  Component Information |  |  | | --- | --- | | Beschreibung: |  | | Losnummer: |  | | Seriennummer: |  |  Scan Information |  |  | | --- | --- | | Röhrenspannung: |  | | Röhrenstromstärke: |  | | Scandauer: |  | | Rekonstruktionsdauer: |  | | Gesamtverarbeitungszeit: |  | | Rekonstruktionsalgorithmus: |  | | Scanmethode: |  | | Geometrie: |  | | Integrationszeit: |  | | Filtern: |  | | Projektionsanzahl: |  | | Datum, Zeit: |  | | Benutzer: |  |  Reconstruction Parameters NO RECONSTRUCTION PARAMETERS AVAILABLE  Import Settings |  |  |  |  | | --- | --- | --- | --- | | Source/Type: | Reconstructed Volume | | | | Name: | weisses Fett aus Volumen 1 | | | | Axes swap mode: | XYZT | | | | Mirror axes: | None | | | | Data mapping: | Ramp | | | | Data range source mapping: | -1...1 | | | | Data range destination mapping: | -1...1 | | | | Data type mapping: | Unknown | | | | Voxel skip: | 0 | 0 | 0 | | Auto region of interest: | Off | | Region of interest (min): | 0 | 0 | 0 | | Region of interest (max): | -1 | -1 | -1 | | Slice interpolation mode: | Off | | | | Slice interpolation threshold: | 0 | | | | Resolution [mm]: | 1 | 1 | 1 | | Resampling mode: | Off | | | | Auto histogram mode: | Off | | Lower auto histogram boundary at (%): | 0 | | Upper auto histogram boundary at (%): | 0 |  Analysis Information |  |  | | --- | --- | | Wall thickness: | 0 | | Defect detection: | 0 | | Nominal/actual comparison: | 0 | | Number of reference objects: | 0 |  File List (659) | files (659) | dimensions | format | type | endian | header | | --- | --- | --- | --- | --- | --- | | C:/.../wat...E000.raw | not available | raw | Unknown | Unknown | 0 | | C:/.../wat...E001.raw | not available | raw | Unknown | Unknown | 0 | | C:/.../wat...E002.raw | not available | raw | Unknown | Unknown | 0 | | C:/.../wat...E003.raw | not available | raw | Unknown | Unknown | 0 | | C:/.../wat...E004.raw | not available | raw | Unknown | Unknown | 0 | | C:/.../wat...E005.raw | not available | raw | Unknown | Unknown | 0 | | C:/.../wat...E006.raw | not available | raw | Unknown | Unknown | 0 | | C:/.../wat...E007.raw | not available | raw | Unknown | Unknown | 0 | | C:/.../wat...E008.raw | not available | raw | Unknown | Unknown | 0 | | C:/.../wat...E009.raw | not available | raw | Unknown | Unknown | 0 | | C:/.../wat...E010.raw | not available | raw | Unknown | Unknown | 0 | | C:/.../wat...E011.raw | not available | raw | Unknown | Unknown | 0 | | C:/.../wat...E012.raw | not available | raw | Unknown | Unknown | 0 | | C:/.../wat...E013.raw | not available | raw | Unknown | Unknown | 0 | | C:/.../wat...E014.raw | not available | raw | Unknown | Unknown | 0 | | C:/.../wat...E015.raw | not available | raw | Unknown | Unknown | 0 | | C:/.../wat...E016.raw | not available | raw | Unknown | Unknown | 0 | | C:/.../wat...E017.raw | not available | raw | Unknown | Unknown | 0 | | C:/.../wat...E018.raw | not available | raw | Unknown | Unknown | 0 | | C:/.../wat...E019.raw | not available | raw | Unknown | Unknown | 0 | | C:/.../wat...E020.raw | not available | raw | Unknown | Unknown | 0 | | C:/.../wat...E021.raw | not available | raw | Unknown | Unknown | 0 | | C:/.../wat...E022.raw | not available | raw | Unknown | Unknown | 0 | | C:/.../wat...E023.raw | not available | raw | Unknown | Unknown | 0 | | C:/.../wat...E024.raw | not available | raw | Unknown | Unknown | 0 | | C:/.../wat...E025.raw | not available | raw | Unknown | Unknown | 0 | | C:/.../wat...E026.raw | not available | raw | Unknown | Unknown | 0 | | C:/.../wat...E027.raw | not available | raw | Unknown | Unknown | 0 | | C:/.../wat...E028.raw | not available | raw | Unknown | Unknown | 0 | | C:/.../wat...E029.raw | not available | raw | Unknown | Unknown | 0 | | C:/.../wat...E030.raw | not available | raw | Unknown | Unknown | 0 | | C:/.../wat...E031.raw | not available | raw | Unknown | Unknown | 0 | | C:/.../wat...E032.raw | not available | raw | Unknown | Unknown | 0 | | C:/.../wat...E033.raw | not available | raw | Unknown | Unknown | 0 | | C:/.../wat...E034.raw | not available | raw | Unknown | Unknown | 0 | | C:/.../wat...E035.raw | not available | raw | Unknown | Unknown | 0 | | C:/.../wat...E036.raw | not available | raw | Unknown | Unknown | 0 | | C:/.../wat...E037.raw | not available | raw | Unknown | Unknown | 0 | | C:/.../wat...E038.raw | not available | raw | Unknown | Unknown | 0 | | C:/.../wat...E039.raw | not available | raw | Unknown | Unknown | 0 | | C:/.../wat...E040.raw | not available | raw | Unknown | Unknown | 0 | | C:/.../wat...E041.raw | not available | raw | Unknown | Unknown | 0 | | C:/.../wat...E042.raw | not available | raw | Unknown | Unknown | 0 | | C:/.../wat...E043.raw | not available | raw | Unknown | Unknown | 0 | | C:/.../wat...E044.raw | not available | raw | Unknown | Unknown | 0 | | C:/.../wat...E045.raw | not available | raw | Unknown | Unknown | 0 | | C:/.../wat...E046.raw | not available | raw | Unknown | Unknown | 0 | | C:/.../wat...E047.raw | not available | raw | Unknown | Unknown | 0 | | C:/.../wat...E048.raw | not available | raw | Unknown | Unknown | 0 | | C:/.../wat...E049.raw | not available | raw | Unknown | Unknown | 0 | | C:/.../wat...E050.raw | not available | raw | Unknown | Unknown | 0 | | C:/.../wat...E051.raw | not available | raw | Unknown | Unknown | 0 | | C:/.../wat...E052.raw | not available | raw | Unknown | Unknown | 0 | | C:/.../wat...E053.raw | not available | raw | Unknown | Unknown | 0 | | C:/.../wat...E054.raw | not available | raw | Unknown | Unknown | 0 | | C:/.../wat...E055.raw | not available | raw | Unknown | Unknown | 0 | | C:/.../wat...E056.raw | not available | raw | Unknown | Unknown | 0 | | C:/.../wat...E057.raw | not available | raw | Unknown | Unknown | 0 | | C:/.../wat...E058.raw | not available | raw | Unknown | Unknown | 0 | | C:/.../wat...E059.raw | not available | raw | Unknown | Unknown | 0 | | C:/.../wat...E060.raw | not available | raw | Unknown | Unknown | 0 | | C:/.../wat...E061.raw | not available | raw | Unknown | Unknown | 0 | | C:/.../wat...E062.raw | not available | raw | Unknown | Unknown | 0 | | C:/.../wat...E063.raw | not available | raw | Unknown | Unknown | 0 | | C:/.../wat...E064.raw | not available | raw | Unknown | Unknown | 0 | | C:/.../wat...E065.raw | not available | raw | Unknown | Unknown | 0 | | C:/.../wat...E066.raw | not available | raw | Unknown | Unknown | 0 | | C:/.../wat...E067.raw | not available | raw | Unknown | Unknown | 0 | | C:/.../wat...E068.raw | not available | raw | Unknown | Unknown | 0 | | C:/.../wat...E069.raw | not available | raw | Unknown | Unknown | 0 | | C:/.../wat...E070.raw | not available | raw | Unknown | Unknown | 0 | | C:/.../wat...E071.raw | not available | raw | Unknown | Unknown | 0 | | C:/.../wat...E072.raw | not available | raw | Unknown | Unknown | 0 | | C:/.../wat...E073.raw | not available | raw | Unknown | Unknown | 0 | | C:/.../wat...E074.raw | not available | raw | Unknown | Unknown | 0 | | C:/.../wat...E075.raw | not available | raw | Unknown | Unknown | 0 | | C:/.../wat...E076.raw | not available | raw | Unknown | Unknown | 0 | | C:/.../wat...E077.raw | not available | raw | Unknown | Unknown | 0 | | C:/.../wat...E078.raw | not available | raw | Unknown | Unknown | 0 | | C:/.../wat...E079.raw | not available | raw | Unknown | Unknown | 0 | | C:/.../wat...E080.raw | not available | raw | Unknown | Unknown | 0 | | C:/.../wat...E081.raw | not available | raw | Unknown | Unknown | 0 | | C:/.../wat...E082.raw | not available | raw | Unknown | Unknown | 0 | | C:/.../wat...E083.raw | not available | raw | Unknown | Unknown | 0 | | C:/.../wat...E084.raw | not available | raw | Unknown | Unknown | 0 | | C:/.../wat...E085.raw | not available | raw | Unknown | Unknown | 0 | | C:/.../wat...E086.raw | not available | raw | Unknown | Unknown | 0 | | C:/.../wat...E087.raw | not available | raw | Unknown | Unknown | 0 | | C:/.../wat...E088.raw | not available | raw | Unknown | Unknown | 0 | | C:/.../wat...E089.raw | not available | raw | Unknown | Unknown | 0 | | C:/.../wat...E090.raw | not available | raw | Unknown | Unknown | 0 | | C:/.../wat...E091.raw | not available | raw | Unknown | Unknown | 0 | | C:/.../wat...E092.raw | not available | raw | Unknown | Unknown | 0 | | C:/.../wat...E093.raw | not available | raw | Unknown | Unknown | 0 | | C:/.../wat...E094.raw | not available | raw | Unknown | Unknown | 0 | | C:/.../wat...E095.raw | not available | raw | Unknown | Unknown | 0 | | C:/.../wat...E096.raw | not available | raw | Unknown | Unknown | 0 | | C:/.../wat...E097.raw | not available | raw | Unknown | Unknown | 0 | | C:/.../wat...E098.raw | not available | raw | Unknown | Unknown | 0 | | C:/.../wat...E099.raw | not available | raw | Unknown | Unknown | 0 | | C:/.../wat...E100.raw | not available | raw | Unknown | Unknown | 0 | | C:/.../wat...E101.raw | not available | raw | Unknown | Unknown | 0 | | C:/.../wat...E102.raw | not available | raw | Unknown | Unknown | 0 | | C:/.../wat...E103.raw | not available | raw | Unknown | Unknown | 0 | | C:/.../wat...E104.raw | not available | raw | Unknown | Unknown | 0 | | C:/.../wat...E105.raw | not available | raw | Unknown | Unknown | 0 | | C:/.../wat...E106.raw | not available | raw | Unknown | Unknown | 0 | | C:/.../wat...E107.raw | not available | raw | Unknown | Unknown | 0 | | C:/.../wat...E108.raw | not available | raw | Unknown | Unknown | 0 | | C:/.../wat...E109.raw | not available | raw | Unknown | Unknown | 0 | | C:/.../wat...E110.raw | not available | raw | Unknown | Unknown | 0 | | C:/.../wat...E111.raw | not available | raw | Unknown | Unknown | 0 | | C:/.../wat...E112.raw | not available | raw | Unknown | Unknown | 0 | | C:/.../wat...E113.raw | not available | raw | Unknown | Unknown | 0 | | C:/.../wat...E114.raw | not available | raw | Unknown | Unknown | 0 | | C:/.../wat...E115.raw | not available | raw | Unknown | Unknown | 0 | | C:/.../wat...E116.raw | not available | raw | Unknown | Unknown | 0 | | C:/.../wat...E117.raw | not available | raw | Unknown | Unknown | 0 | | C:/.../wat...E118.raw | not available | raw | Unknown | Unknown | 0 | | C:/.../wat...E119.raw | not available | raw | Unknown | Unknown | 0 | | C:/.../wat...E120.raw | not available | raw | Unknown | Unknown | 0 | | C:/.../wat...E121.raw | not available | raw | Unknown | Unknown | 0 | | C:/.../wat...E122.raw | not available | raw | Unknown | Unknown | 0 | | C:/.../wat...E123.raw | not available | raw | Unknown | Unknown | 0 | | C:/.../wat...E124.raw | not available | raw | Unknown | Unknown | 0 | | C:/.../wat...E125.raw | not available | raw | Unknown | Unknown | 0 | | C:/.../wat...E126.raw | not available | raw | Unknown | Unknown | 0 | | C:/.../wat...E127.raw | not available | raw | Unknown | Unknown | 0 | | C:/.../wat...E128.raw | not available | raw | Unknown | Unknown | 0 | | C:/.../wat...E129.raw | not available | raw | Unknown | Unknown | 0 | | C:/.../wat...E130.raw | not available | raw | Unknown | Unknown | 0 | | C:/.../wat...E131.raw | not available | raw | Unknown | Unknown | 0 | | C:/.../wat...E132.raw | not available | raw | Unknown | Unknown | 0 | | C:/.../wat...E133.raw | not available | raw | Unknown | Unknown | 0 | | C:/.../wat...E134.raw | not available | raw | Unknown | Unknown | 0 | | C:/.../wat...E135.raw | not available | raw | Unknown | Unknown | 0 | | C:/.../wat...E136.raw | not available | raw | Unknown | Unknown | 0 | | C:/.../wat...E137.raw | not available | raw | Unknown | Unknown | 0 | | C:/.../wat...E138.raw | not available | raw | Unknown | Unknown | 0 | | C:/.../wat...E139.raw | not available | raw | Unknown | Unknown | 0 | | C:/.../wat...E140.raw | not available | raw | Unknown | Unknown | 0 | | C:/.../wat...E141.raw | not available | raw | Unknown | Unknown | 0 | | C:/.../wat...E142.raw | not available | raw | Unknown | Unknown | 0 | | C:/.../wat...E143.raw | not available | raw | Unknown | Unknown | 0 | | C:/.../wat...E144.raw | not available | raw | Unknown | Unknown | 0 | | C:/.../wat...E145.raw | not available | raw | Unknown | Unknown | 0 | | C:/.../wat...E146.raw | not available | raw | Unknown | Unknown | 0 | | C:/.../wat...E147.raw | not available | raw | Unknown | Unknown | 0 | | C:/.../wat...E148.raw | not available | raw | Unknown | Unknown | 0 | | C:/.../wat...E149.raw | not available | raw | Unknown | Unknown | 0 | | C:/.../wat...E150.raw | not available | raw | Unknown | Unknown | 0 | | C:/.../wat...E151.raw | not available | raw | Unknown | Unknown | 0 | | C:/.../wat...E152.raw | not available | raw | Unknown | Unknown | 0 | | C:/.../wat...E153.raw | not available | raw | Unknown | Unknown | 0 | | C:/.../wat...E154.raw | not available | raw | Unknown | Unknown | 0 | | C:/.../wat...E155.raw | not available | raw | Unknown | Unknown | 0 | | C:/.../wat...E156.raw | not available | raw | Unknown | Unknown | 0 | | C:/.../wat...E157.raw | not available | raw | Unknown | Unknown | 0 | | C:/.../wat...E158.raw | not available | raw | Unknown | Unknown | 0 | | C:/.../wat...E159.raw | not available | raw | Unknown | Unknown | 0 | | C:/.../wat...E160.raw | not available | raw | Unknown | Unknown | 0 | | C:/.../wat...E161.raw | not available | raw | Unknown | Unknown | 0 | | C:/.../wat...E162.raw | not available | raw | Unknown | Unknown | 0 | | C:/.../wat...E163.raw | not available | raw | Unknown | Unknown | 0 | | C:/.../wat...E164.raw | not available | raw | Unknown | Unknown | 0 | | C:/.../wat...E165.raw | not available | raw | Unknown | Unknown | 0 | | C:/.../wat...E166.raw | not available | raw | Unknown | Unknown | 0 | | C:/.../wat...E167.raw | not available | raw | Unknown | Unknown | 0 | | C:/.../wat...E168.raw | not available | raw | Unknown | Unknown | 0 | | C:/.../wat...E169.raw | not available | raw | Unknown | Unknown | 0 | | C:/.../wat...E170.raw | not available | raw | Unknown | Unknown | 0 | | C:/.../wat...E171.raw | not available | raw | Unknown | Unknown | 0 | | C:/.../wat...E172.raw | not available | raw | Unknown | Unknown | 0 | | C:/.../wat...E173.raw | not available | raw | Unknown | Unknown | 0 | | C:/.../wat...E174.raw | not available | raw | Unknown | Unknown | 0 | | C:/.../wat...E175.raw | not available | raw | Unknown | Unknown | 0 | | C:/.../wat...E176.raw | not available | raw | Unknown | Unknown | 0 | | C:/.../wat...E177.raw | not available | raw | Unknown | Unknown | 0 | | C:/.../wat...E178.raw | not available | raw | Unknown | Unknown | 0 | | C:/.../wat...E179.raw | not available | raw | Unknown | Unknown | 0 | | C:/.../wat...E180.raw | not available | raw | Unknown | Unknown | 0 | | C:/.../wat...E181.raw | not available | raw | Unknown | Unknown | 0 | | C:/.../wat...E182.raw | not available | raw | Unknown | Unknown | 0 | | C:/.../wat...E183.raw | not available | raw | Unknown | Unknown | 0 | | C:/.../wat...E184.raw | not available | raw | Unknown | Unknown | 0 | | C:/.../wat...E185.raw | not available | raw | Unknown | Unknown | 0 | | C:/.../wat...E186.raw | not available | raw | Unknown | Unknown | 0 | | C:/.../wat...E187.raw | not available | raw | Unknown | Unknown | 0 | | C:/.../wat...E188.raw | not available | raw | Unknown | Unknown | 0 | | C:/.../wat...E189.raw | not available | raw | Unknown | Unknown | 0 | | C:/.../wat...E190.raw | not available | raw | Unknown | Unknown | 0 | | C:/.../wat...E191.raw | not available | raw | Unknown | Unknown | 0 | | C:/.../wat...E192.raw | not available | raw | Unknown | Unknown | 0 | | C:/.../wat...E193.raw | not available | raw | Unknown | Unknown | 0 | | C:/.../wat...E194.raw | not available | raw | Unknown | Unknown | 0 | | C:/.../wat...E195.raw | not available | raw | Unknown | Unknown | 0 | | C:/.../wat...E196.raw | not available | raw | Unknown | Unknown | 0 | | C:/.../wat...E197.raw | not available | raw | Unknown | Unknown | 0 | | C:/.../wat...E198.raw | not available | raw | Unknown | Unknown | 0 | | C:/.../wat...E199.raw | not available | raw | Unknown | Unknown | 0 | | C:/.../wat...E200.raw | not available | raw | Unknown | Unknown | 0 | | C:/.../wat...E201.raw | not available | raw | Unknown | Unknown | 0 | | C:/.../wat...E202.raw | not available | raw | Unknown | Unknown | 0 | | C:/.../wat...E203.raw | not available | raw | Unknown | Unknown | 0 | | C:/.../wat...E204.raw | not available | raw | Unknown | Unknown | 0 | | C:/.../wat...E205.raw | not available | raw | Unknown | Unknown | 0 | | C:/.../wat...E206.raw | not available | raw | Unknown | Unknown | 0 | | C:/.../wat...E207.raw | not available | raw | Unknown | Unknown | 0 | | C:/.../wat...E208.raw | not available | raw | Unknown | Unknown | 0 | | C:/.../wat...E209.raw | not available | raw | Unknown | Unknown | 0 | | C:/.../wat...E210.raw | not available | raw | Unknown | Unknown | 0 | | C:/.../wat...E211.raw | not available | raw | Unknown | Unknown | 0 | | C:/.../wat...E212.raw | not available | raw | Unknown | Unknown | 0 | | C:/.../wat...E213.raw | not available | raw | Unknown | Unknown | 0 | | C:/.../wat...E214.raw | not available | raw | Unknown | Unknown | 0 | | C:/.../wat...E215.raw | not available | raw | Unknown | Unknown | 0 | | C:/.../wat...E216.raw | not available | raw | Unknown | Unknown | 0 | | C:/.../wat...E217.raw | not available | raw | Unknown | Unknown | 0 | | C:/.../wat...E218.raw | not available | raw | Unknown | Unknown | 0 | | C:/.../wat...E219.raw | not available | raw | Unknown | Unknown | 0 | | C:/.../wat...E220.raw | not available | raw | Unknown | Unknown | 0 | | C:/.../wat...E221.raw | not available | raw | Unknown | Unknown | 0 | | C:/.../wat...E222.raw | not available | raw | Unknown | Unknown | 0 | | C:/.../wat...E223.raw | not available | raw | Unknown | Unknown | 0 | | C:/.../wat...E224.raw | not available | raw | Unknown | Unknown | 0 | | C:/.../wat...E225.raw | not available | raw | Unknown | Unknown | 0 | | C:/.../wat...E226.raw | not available | raw | Unknown | Unknown | 0 | | C:/.../wat...E227.raw | not available | raw | Unknown | Unknown | 0 | | C:/.../wat...E228.raw | not available | raw | Unknown | Unknown | 0 | | C:/.../wat...E229.raw | not available | raw | Unknown | Unknown | 0 | | C:/.../wat...E230.raw | not available | raw | Unknown | Unknown | 0 | | C:/.../wat...E231.raw | not available | raw | Unknown | Unknown | 0 | | C:/.../wat...E232.raw | not available | raw | Unknown | Unknown | 0 | | C:/.../wat...E233.raw | not available | raw | Unknown | Unknown | 0 | | C:/.../wat...E234.raw | not available | raw | Unknown | Unknown | 0 | | C:/.../wat...E235.raw | not available | raw | Unknown | Unknown | 0 | | C:/.../wat...E236.raw | not available | raw | Unknown | Unknown | 0 | | C:/.../wat...E237.raw | not available | raw | Unknown | Unknown | 0 | | C:/.../wat...E238.raw | not available | raw | Unknown | Unknown | 0 | | C:/.../wat...E239.raw | not available | raw | Unknown | Unknown | 0 | | C:/.../wat...E240.raw | not available | raw | Unknown | Unknown | 0 | | C:/.../wat...E241.raw | not available | raw | Unknown | Unknown | 0 | | C:/.../wat...E242.raw | not available | raw | Unknown | Unknown | 0 | | C:/.../wat...E243.raw | not available | raw | Unknown | Unknown | 0 | | C:/.../wat...E244.raw | not available | raw | Unknown | Unknown | 0 | | C:/.../wat...E245.raw | not available | raw | Unknown | Unknown | 0 | | C:/.../wat...E246.raw | not available | raw | Unknown | Unknown | 0 | | C:/.../wat...E247.raw | not available | raw | Unknown | Unknown | 0 | | C:/.../wat...E248.raw | not available | raw | Unknown | Unknown | 0 | | C:/.../wat...E249.raw | not available | raw | Unknown | Unknown | 0 | | C:/.../wat...E250.raw | not available | raw | Unknown | Unknown | 0 | | C:/.../wat...E251.raw | not available | raw | Unknown | Unknown | 0 | | C:/.../wat...E252.raw | not available | raw | Unknown | Unknown | 0 | | C:/.../wat...E253.raw | not available | raw | Unknown | Unknown | 0 | | C:/.../wat...E254.raw | not available | raw | Unknown | Unknown | 0 | | C:/.../wat...E255.raw | not available | raw | Unknown | Unknown | 0 | | C:/.../wat...E256.raw | not available | raw | Unknown | Unknown | 0 | | C:/.../wat...E257.raw | not available | raw | Unknown | Unknown | 0 | | C:/.../wat...E258.raw | not available | raw | Unknown | Unknown | 0 | | C:/.../wat...E259.raw | not available | raw | Unknown | Unknown | 0 | | C:/.../wat...E260.raw | not available | raw | Unknown | Unknown | 0 | | C:/.../wat...E261.raw | not available | raw | Unknown | Unknown | 0 | | C:/.../wat...E262.raw | not available | raw | Unknown | Unknown | 0 | | C:/.../wat...E263.raw | not available | raw | Unknown | Unknown | 0 | | C:/.../wat...E264.raw | not available | raw | Unknown | Unknown | 0 | | C:/.../wat...E265.raw | not available | raw | Unknown | Unknown | 0 | | C:/.../wat...E266.raw | not available | raw | Unknown | Unknown | 0 | | C:/.../wat...E267.raw | not available | raw | Unknown | Unknown | 0 | | C:/.../wat...E268.raw | not available | raw | Unknown | Unknown | 0 | | C:/.../wat...E269.raw | not available | raw | Unknown | Unknown | 0 | | C:/.../wat...E270.raw | not available | raw | Unknown | Unknown | 0 | | C:/.../wat...E271.raw | not available | raw | Unknown | Unknown | 0 | | C:/.../wat...E272.raw | not available | raw | Unknown | Unknown | 0 | | C:/.../wat...E273.raw | not available | raw | Unknown | Unknown | 0 | | C:/.../wat...E274.raw | not available | raw | Unknown | Unknown | 0 | | C:/.../wat...E275.raw | not available | raw | Unknown | Unknown | 0 | | C:/.../wat...E276.raw | not available | raw | Unknown | Unknown | 0 | | C:/.../wat...E277.raw | not available | raw | Unknown | Unknown | 0 | | C:/.../wat...E278.raw | not available | raw | Unknown | Unknown | 0 | | C:/.../wat...E279.raw | not available | raw | Unknown | Unknown | 0 | | C:/.../wat...E280.raw | not available | raw | Unknown | Unknown | 0 | | C:/.../wat...E281.raw | not available | raw | Unknown | Unknown | 0 | | C:/.../wat...E282.raw | not available | raw | Unknown | Unknown | 0 | | C:/.../wat...E283.raw | not available | raw | Unknown | Unknown | 0 | | C:/.../wat...E284.raw | not available | raw | Unknown | Unknown | 0 | | C:/.../wat...E285.raw | not available | raw | Unknown | Unknown | 0 | | C:/.../wat...E286.raw | not available | raw | Unknown | Unknown | 0 | | C:/.../wat...E287.raw | not available | raw | Unknown | Unknown | 0 | | C:/.../wat...E288.raw | not available | raw | Unknown | Unknown | 0 | | C:/.../wat...E289.raw | not available | raw | Unknown | Unknown | 0 | | C:/.../wat...E290.raw | not available | raw | Unknown | Unknown | 0 | | C:/.../wat...E291.raw | not available | raw | Unknown | Unknown | 0 | | C:/.../wat...E292.raw | not available | raw | Unknown | Unknown | 0 | | C:/.../wat...E293.raw | not available | raw | Unknown | Unknown | 0 | | C:/.../wat...E294.raw | not available | raw | Unknown | Unknown | 0 | | C:/.../wat...E295.raw | not available | raw | Unknown | Unknown | 0 | | C:/.../wat...E296.raw | not available | raw | Unknown | Unknown | 0 | | C:/.../wat...E297.raw | not available | raw | Unknown | Unknown | 0 | | C:/.../wat...E298.raw | not available | raw | Unknown | Unknown | 0 | | C:/.../wat...E299.raw | not available | raw | Unknown | Unknown | 0 | | C:/.../wat...E300.raw | not available | raw | Unknown | Unknown | 0 | | C:/.../wat...E301.raw | not available | raw | Unknown | Unknown | 0 | | C:/.../wat...E302.raw | not available | raw | Unknown | Unknown | 0 | | C:/.../wat...E303.raw | not available | raw | Unknown | Unknown | 0 | | C:/.../wat...E304.raw | not available | raw | Unknown | Unknown | 0 | | C:/.../wat...E305.raw | not available | raw | Unknown | Unknown | 0 | | C:/.../wat...E306.raw | not available | raw | Unknown | Unknown | 0 | | C:/.../wat...E307.raw | not available | raw | Unknown | Unknown | 0 | | C:/.../wat...E308.raw | not available | raw | Unknown | Unknown | 0 | | C:/.../wat...E309.raw | not available | raw | Unknown | Unknown | 0 | | C:/.../wat...E310.raw | not available | raw | Unknown | Unknown | 0 | | C:/.../wat...E311.raw | not available | raw | Unknown | Unknown | 0 | | C:/.../wat...E312.raw | not available | raw | Unknown | Unknown | 0 | | C:/.../wat...E313.raw | not available | raw | Unknown | Unknown | 0 | | C:/.../wat...E314.raw | not available | raw | Unknown | Unknown | 0 | | C:/.../wat...E315.raw | not available | raw | Unknown | Unknown | 0 | | C:/.../wat...E316.raw | not available | raw | Unknown | Unknown | 0 | | C:/.../wat...E317.raw | not available | raw | Unknown | Unknown | 0 | | C:/.../wat...E318.raw | not available | raw | Unknown | Unknown | 0 | | C:/.../wat...E319.raw | not available | raw | Unknown | Unknown | 0 | | C:/.../wat...E320.raw | not available | raw | Unknown | Unknown | 0 | | C:/.../wat...E321.raw | not available | raw | Unknown | Unknown | 0 | | C:/.../wat...E322.raw | not available | raw | Unknown | Unknown | 0 | | C:/.../wat...E323.raw | not available | raw | Unknown | Unknown | 0 | | C:/.../wat...E324.raw | not available | raw | Unknown | Unknown | 0 | | C:/.../wat...E325.raw | not available | raw | Unknown | Unknown | 0 | | C:/.../wat...E326.raw | not available | raw | Unknown | Unknown | 0 | | C:/.../wat...E327.raw | not available | raw | Unknown | Unknown | 0 | | C:/.../wat...E328.raw | not available | raw | Unknown | Unknown | 0 | | C:/.../wat...E329.raw | not available | raw | Unknown | Unknown | 0 | | C:/.../wat...E330.raw | not available | raw | Unknown | Unknown | 0 | | C:/.../wat...E331.raw | not available | raw | Unknown | Unknown | 0 | | C:/.../wat...E332.raw | not available | raw | Unknown | Unknown | 0 | | C:/.../wat...E333.raw | not available | raw | Unknown | Unknown | 0 | | C:/.../wat...E334.raw | not available | raw | Unknown | Unknown | 0 | | C:/.../wat...E335.raw | not available | raw | Unknown | Unknown | 0 | | C:/.../wat...E336.raw | not available | raw | Unknown | Unknown | 0 | | C:/.../wat...E337.raw | not available | raw | Unknown | Unknown | 0 | | C:/.../wat...E338.raw | not available | raw | Unknown | Unknown | 0 | | C:/.../wat...E339.raw | not available | raw | Unknown | Unknown | 0 | | C:/.../wat...E340.raw | not available | raw | Unknown | Unknown | 0 | | C:/.../wat...E341.raw | not available | raw | Unknown | Unknown | 0 | | C:/.../wat...E342.raw | not available | raw | Unknown | Unknown | 0 | | C:/.../wat...E343.raw | not available | raw | Unknown | Unknown | 0 | | C:/.../wat...E344.raw | not available | raw | Unknown | Unknown | 0 | | C:/.../wat...E345.raw | not available | raw | Unknown | Unknown | 0 | | C:/.../wat...E346.raw | not available | raw | Unknown | Unknown | 0 | | C:/.../wat...E347.raw | not available | raw | Unknown | Unknown | 0 | | C:/.../wat...E348.raw | not available | raw | Unknown | Unknown | 0 | | C:/.../wat...E349.raw | not available | raw | Unknown | Unknown | 0 | | C:/.../wat...E350.raw | not available | raw | Unknown | Unknown | 0 | | C:/.../wat...E351.raw | not available | raw | Unknown | Unknown | 0 | | C:/.../wat...E352.raw | not available | raw | Unknown | Unknown | 0 | | C:/.../wat...E353.raw | not available | raw | Unknown | Unknown | 0 | | C:/.../wat...E354.raw | not available | raw | Unknown | Unknown | 0 | | C:/.../wat...E355.raw | not available | raw | Unknown | Unknown | 0 | | C:/.../wat...E356.raw | not available | raw | Unknown | Unknown | 0 | | C:/.../wat...E357.raw | not available | raw | Unknown | Unknown | 0 | | C:/.../wat...E358.raw | not available | raw | Unknown | Unknown | 0 | | C:/.../wat...E359.raw | not available | raw | Unknown | Unknown | 0 | | C:/.../wat...E360.raw | not available | raw | Unknown | Unknown | 0 | | C:/.../wat...E361.raw | not available | raw | Unknown | Unknown | 0 | | C:/.../wat...E362.raw | not available | raw | Unknown | Unknown | 0 | | C:/.../wat...E363.raw | not available | raw | Unknown | Unknown | 0 | | C:/.../wat...E364.raw | not available | raw | Unknown | Unknown | 0 | | C:/.../wat...E365.raw | not available | raw | Unknown | Unknown | 0 | | C:/.../wat...E366.raw | not available | raw | Unknown | Unknown | 0 | | C:/.../wat...E367.raw | not available | raw | Unknown | Unknown | 0 | | C:/.../wat...E368.raw | not available | raw | Unknown | Unknown | 0 | | C:/.../wat...E369.raw | not available | raw | Unknown | Unknown | 0 | | C:/.../wat...E370.raw | not available | raw | Unknown | Unknown | 0 | | C:/.../wat...E371.raw | not available | raw | Unknown | Unknown | 0 | | C:/.../wat...E372.raw | not available | raw | Unknown | Unknown | 0 | | C:/.../wat...E373.raw | not available | raw | Unknown | Unknown | 0 | | C:/.../wat...E374.raw | not available | raw | Unknown | Unknown | 0 | | C:/.../wat...E375.raw | not available | raw | Unknown | Unknown | 0 | | C:/.../wat...E376.raw | not available | raw | Unknown | Unknown | 0 | | C:/.../wat...E377.raw | not available | raw | Unknown | Unknown | 0 | | C:/.../wat...E378.raw | not available | raw | Unknown | Unknown | 0 | | C:/.../wat...E379.raw | not available | raw | Unknown | Unknown | 0 | | C:/.../wat...E380.raw | not available | raw | Unknown | Unknown | 0 | | C:/.../wat...E381.raw | not available | raw | Unknown | Unknown | 0 | | C:/.../wat...E382.raw | not available | raw | Unknown | Unknown | 0 | | C:/.../wat...E383.raw | not available | raw | Unknown | Unknown | 0 | | C:/.../wat...E384.raw | not available | raw | Unknown | Unknown | 0 | | C:/.../wat...E385.raw | not available | raw | Unknown | Unknown | 0 | | C:/.../wat...E386.raw | not available | raw | Unknown | Unknown | 0 | | C:/.../wat...E387.raw | not available | raw | Unknown | Unknown | 0 | | C:/.../wat...E388.raw | not available | raw | Unknown | Unknown | 0 | | C:/.../wat...E389.raw | not available | raw | Unknown | Unknown | 0 | | C:/.../wat...E390.raw | not available | raw | Unknown | Unknown | 0 | | C:/.../wat...E391.raw | not available | raw | Unknown | Unknown | 0 | | C:/.../wat...E392.raw | not available | raw | Unknown | Unknown | 0 | | C:/.../wat...E393.raw | not available | raw | Unknown | Unknown | 0 | | C:/.../wat...E394.raw | not available | raw | Unknown | Unknown | 0 | | C:/.../wat...E395.raw | not available | raw | Unknown | Unknown | 0 | | C:/.../wat...E396.raw | not available | raw | Unknown | Unknown | 0 | | C:/.../wat...E397.raw | not available | raw | Unknown | Unknown | 0 | | C:/.../wat...E398.raw | not available | raw | Unknown | Unknown | 0 | | C:/.../wat...E399.raw | not available | raw | Unknown | Unknown | 0 | | C:/.../wat...E400.raw | not available | raw | Unknown | Unknown | 0 | | C:/.../wat...E401.raw | not available | raw | Unknown | Unknown | 0 | | C:/.../wat...E402.raw | not available | raw | Unknown | Unknown | 0 | | C:/.../wat...E403.raw | not available | raw | Unknown | Unknown | 0 | | C:/.../wat...E404.raw | not available | raw | Unknown | Unknown | 0 | | C:/.../wat...E405.raw | not available | raw | Unknown | Unknown | 0 | | C:/.../wat...E406.raw | not available | raw | Unknown | Unknown | 0 | | C:/.../wat...E407.raw | not available | raw | Unknown | Unknown | 0 | | C:/.../wat...E408.raw | not available | raw | Unknown | Unknown | 0 | | C:/.../wat...E409.raw | not available | raw | Unknown | Unknown | 0 | | C:/.../wat...E410.raw | not available | raw | Unknown | Unknown | 0 | | C:/.../wat...E411.raw | not available | raw | Unknown | Unknown | 0 | | C:/.../wat...E412.raw | not available | raw | Unknown | Unknown | 0 | | C:/.../wat...E413.raw | not available | raw | Unknown | Unknown | 0 | | C:/.../wat...E414.raw | not available | raw | Unknown | Unknown | 0 | | C:/.../wat...E415.raw | not available | raw | Unknown | Unknown | 0 | | C:/.../wat...E416.raw | not available | raw | Unknown | Unknown | 0 | | C:/.../wat...E417.raw | not available | raw | Unknown | Unknown | 0 | | C:/.../wat...E418.raw | not available | raw | Unknown | Unknown | 0 | | C:/.../wat...E419.raw | not available | raw | Unknown | Unknown | 0 | | C:/.../wat...E420.raw | not available | raw | Unknown | Unknown | 0 | | C:/.../wat...E421.raw | not available | raw | Unknown | Unknown | 0 | | C:/.../wat...E422.raw | not available | raw | Unknown | Unknown | 0 | | C:/.../wat...E423.raw | not available | raw | Unknown | Unknown | 0 | | C:/.../wat...E424.raw | not available | raw | Unknown | Unknown | 0 | | C:/.../wat...E425.raw | not available | raw | Unknown | Unknown | 0 | | C:/.../wat...E426.raw | not available | raw | Unknown | Unknown | 0 | | C:/.../wat...E427.raw | not available | raw | Unknown | Unknown | 0 | | C:/.../wat...E428.raw | not available | raw | Unknown | Unknown | 0 | | C:/.../wat...E429.raw | not available | raw | Unknown | Unknown | 0 | | C:/.../wat...E430.raw | not available | raw | Unknown | Unknown | 0 | | C:/.../wat...E431.raw | not available | raw | Unknown | Unknown | 0 | | C:/.../wat...E432.raw | not available | raw | Unknown | Unknown | 0 | | C:/.../wat...E433.raw | not available | raw | Unknown | Unknown | 0 | | C:/.../wat...E434.raw | not available | raw | Unknown | Unknown | 0 | | C:/.../wat...E435.raw | not available | raw | Unknown | Unknown | 0 | | C:/.../wat...E436.raw | not available | raw | Unknown | Unknown | 0 | | C:/.../wat...E437.raw | not available | raw | Unknown | Unknown | 0 | | C:/.../wat...E438.raw | not available | raw | Unknown | Unknown | 0 | | C:/.../wat...E439.raw | not available | raw | Unknown | Unknown | 0 | | C:/.../wat...E440.raw | not available | raw | Unknown | Unknown | 0 | | C:/.../wat...E441.raw | not available | raw | Unknown | Unknown | 0 | | C:/.../wat...E442.raw | not available | raw | Unknown | Unknown | 0 | | C:/.../wat...E443.raw | not available | raw | Unknown | Unknown | 0 | | C:/.../wat...E444.raw | not available | raw | Unknown | Unknown | 0 | | C:/.../wat...E445.raw | not available | raw | Unknown | Unknown | 0 | | C:/.../wat...E446.raw | not available | raw | Unknown | Unknown | 0 | | C:/.../wat...E447.raw | not available | raw | Unknown | Unknown | 0 | | C:/.../wat...E448.raw | not available | raw | Unknown | Unknown | 0 | | C:/.../wat...E449.raw | not available | raw | Unknown | Unknown | 0 | | C:/.../wat...E450.raw | not available | raw | Unknown | Unknown | 0 | | C:/.../wat...E451.raw | not available | raw | Unknown | Unknown | 0 | | C:/.../wat...E452.raw | not available | raw | Unknown | Unknown | 0 | | C:/.../wat...E453.raw | not available | raw | Unknown | Unknown | 0 | | C:/.../wat...E454.raw | not available | raw | Unknown | Unknown | 0 | | C:/.../wat...E455.raw | not available | raw | Unknown | Unknown | 0 | | C:/.../wat...E456.raw | not available | raw | Unknown | Unknown | 0 | | C:/.../wat...E457.raw | not available | raw | Unknown | Unknown | 0 | | C:/.../wat...E458.raw | not available | raw | Unknown | Unknown | 0 | | C:/.../wat...E459.raw | not available | raw | Unknown | Unknown | 0 | | C:/.../wat...E460.raw | not available | raw | Unknown | Unknown | 0 | | C:/.../wat...E461.raw | not available | raw | Unknown | Unknown | 0 | | C:/.../wat...E462.raw | not available | raw | Unknown | Unknown | 0 | | C:/.../wat...E463.raw | not available | raw | Unknown | Unknown | 0 | | C:/.../wat...E464.raw | not available | raw | Unknown | Unknown | 0 | | C:/.../wat...E465.raw | not available | raw | Unknown | Unknown | 0 | | C:/.../wat...E466.raw | not available | raw | Unknown | Unknown | 0 | | C:/.../wat...E467.raw | not available | raw | Unknown | Unknown | 0 | | C:/.../wat...E468.raw | not available | raw | Unknown | Unknown | 0 | | C:/.../wat...E469.raw | not available | raw | Unknown | Unknown | 0 | | C:/.../wat...E470.raw | not available | raw | Unknown | Unknown | 0 | | C:/.../wat...E471.raw | not available | raw | Unknown | Unknown | 0 | | C:/.../wat...E472.raw | not available | raw | Unknown | Unknown | 0 | | C:/.../wat...E473.raw | not available | raw | Unknown | Unknown | 0 | | C:/.../wat...E474.raw | not available | raw | Unknown | Unknown | 0 | | C:/.../wat...E475.raw | not available | raw | Unknown | Unknown | 0 | | C:/.../wat...E476.raw | not available | raw | Unknown | Unknown | 0 | | C:/.../wat...E477.raw | not available | raw | Unknown | Unknown | 0 | | C:/.../wat...E478.raw | not available | raw | Unknown | Unknown | 0 | | C:/.../wat...E479.raw | not available | raw | Unknown | Unknown | 0 | | C:/.../wat...E480.raw | not available | raw | Unknown | Unknown | 0 | | C:/.../wat...E481.raw | not available | raw | Unknown | Unknown | 0 | | C:/.../wat...E482.raw | not available | raw | Unknown | Unknown | 0 | | C:/.../wat...E483.raw | not available | raw | Unknown | Unknown | 0 | | C:/.../wat...E484.raw | not available | raw | Unknown | Unknown | 0 | | C:/.../wat...E485.raw | not available | raw | Unknown | Unknown | 0 | | C:/.../wat...E486.raw | not available | raw | Unknown | Unknown | 0 | | C:/.../wat...E487.raw | not available | raw | Unknown | Unknown | 0 | | C:/.../wat...E488.raw | not available | raw | Unknown | Unknown | 0 | | C:/.../wat...E489.raw | not available | raw | Unknown | Unknown | 0 | | C:/.../wat...E490.raw | not available | raw | Unknown | Unknown | 0 | | C:/.../wat...E491.raw | not available | raw | Unknown | Unknown | 0 | | C:/.../wat...E492.raw | not available | raw | Unknown | Unknown | 0 | | C:/.../wat...E493.raw | not available | raw | Unknown | Unknown | 0 | | C:/.../wat...E494.raw | not available | raw | Unknown | Unknown | 0 | | C:/.../wat...E495.raw | not available | raw | Unknown | Unknown | 0 | | C:/.../wat...E496.raw | not available | raw | Unknown | Unknown | 0 | | C:/.../wat...E497.raw | not available | raw | Unknown | Unknown | 0 | | C:/.../wat...E498.raw | not available | raw | Unknown | Unknown | 0 | | C:/.../wat...E499.raw | not available | raw | Unknown | Unknown | 0 | | C:/.../wat...E500.raw | not available | raw | Unknown | Unknown | 0 | | C:/.../wat...E501.raw | not available | raw | Unknown | Unknown | 0 | | C:/.../wat...E502.raw | not available | raw | Unknown | Unknown | 0 | | C:/.../wat...E503.raw | not available | raw | Unknown | Unknown | 0 | | C:/.../wat...E504.raw | not available | raw | Unknown | Unknown | 0 | | C:/.../wat...E505.raw | not available | raw | Unknown | Unknown | 0 | | C:/.../wat...E506.raw | not available | raw | Unknown | Unknown | 0 | | C:/.../wat...E507.raw | not available | raw | Unknown | Unknown | 0 | | C:/.../wat...E508.raw | not available | raw | Unknown | Unknown | 0 | | C:/.../wat...E509.raw | not available | raw | Unknown | Unknown | 0 | | C:/.../wat...E510.raw | not available | raw | Unknown | Unknown | 0 | | C:/.../wat...E511.raw | not available | raw | Unknown | Unknown | 0 | | C:/.../wat...E512.raw | not available | raw | Unknown | Unknown | 0 | | C:/.../wat...E513.raw | not available | raw | Unknown | Unknown | 0 | | C:/.../wat...E514.raw | not available | raw | Unknown | Unknown | 0 | | C:/.../wat...E515.raw | not available | raw | Unknown | Unknown | 0 | | C:/.../wat...E516.raw | not available | raw | Unknown | Unknown | 0 | | C:/.../wat...E517.raw | not available | raw | Unknown | Unknown | 0 | | C:/.../wat...E518.raw | not available | raw | Unknown | Unknown | 0 | | C:/.../wat...E519.raw | not available | raw | Unknown | Unknown | 0 | | C:/.../wat...E520.raw | not available | raw | Unknown | Unknown | 0 | | C:/.../wat...E521.raw | not available | raw | Unknown | Unknown | 0 | | C:/.../wat...E522.raw | not available | raw | Unknown | Unknown | 0 | | C:/.../wat...E523.raw | not available | raw | Unknown | Unknown | 0 | | C:/.../wat...E524.raw | not available | raw | Unknown | Unknown | 0 | | C:/.../wat...E525.raw | not available | raw | Unknown | Unknown | 0 | | C:/.../wat...E526.raw | not available | raw | Unknown | Unknown | 0 | | C:/.../wat...E527.raw | not available | raw | Unknown | Unknown | 0 | | C:/.../wat...E528.raw | not available | raw | Unknown | Unknown | 0 | | C:/.../wat...E529.raw | not available | raw | Unknown | Unknown | 0 | | C:/.../wat...E530.raw | not available | raw | Unknown | Unknown | 0 | | C:/.../wat...E531.raw | not available | raw | Unknown | Unknown | 0 | | C:/.../wat...E532.raw | not available | raw | Unknown | Unknown | 0 | | C:/.../wat...E533.raw | not available | raw | Unknown | Unknown | 0 | | C:/.../wat...E534.raw | not available | raw | Unknown | Unknown | 0 | | C:/.../wat...E535.raw | not available | raw | Unknown | Unknown | 0 | | C:/.../wat...E536.raw | not available | raw | Unknown | Unknown | 0 | | C:/.../wat...E537.raw | not available | raw | Unknown | Unknown | 0 | | C:/.../wat...E538.raw | not available | raw | Unknown | Unknown | 0 | | C:/.../wat...E539.raw | not available | raw | Unknown | Unknown | 0 | | C:/.../wat...E540.raw | not available | raw | Unknown | Unknown | 0 | | C:/.../wat...E541.raw | not available | raw | Unknown | Unknown | 0 | | C:/.../wat...E542.raw | not available | raw | Unknown | Unknown | 0 | | C:/.../wat...E543.raw | not available | raw | Unknown | Unknown | 0 | | C:/.../wat...E544.raw | not available | raw | Unknown | Unknown | 0 | | C:/.../wat...E545.raw | not available | raw | Unknown | Unknown | 0 | | C:/.../wat...E546.raw | not available | raw | Unknown | Unknown | 0 | | C:/.../wat...E547.raw | not available | raw | Unknown | Unknown | 0 | | C:/.../wat...E548.raw | not available | raw | Unknown | Unknown | 0 | | C:/.../wat...E549.raw | not available | raw | Unknown | Unknown | 0 | | C:/.../wat...E550.raw | not available | raw | Unknown | Unknown | 0 | | C:/.../wat...E551.raw | not available | raw | Unknown | Unknown | 0 | | C:/.../wat...E552.raw | not available | raw | Unknown | Unknown | 0 | | C:/.../wat...E553.raw | not available | raw | Unknown | Unknown | 0 | | C:/.../wat...E554.raw | not available | raw | Unknown | Unknown | 0 | | C:/.../wat...E555.raw | not available | raw | Unknown | Unknown | 0 | | C:/.../wat...E556.raw | not available | raw | Unknown | Unknown | 0 | | C:/.../wat...E557.raw | not available | raw | Unknown | Unknown | 0 | | C:/.../wat...E558.raw | not available | raw | Unknown | Unknown | 0 | | C:/.../wat...E559.raw | not available | raw | Unknown | Unknown | 0 | | C:/.../wat...E560.raw | not available | raw | Unknown | Unknown | 0 | | C:/.../wat...E561.raw | not available | raw | Unknown | Unknown | 0 | | C:/.../wat...E562.raw | not available | raw | Unknown | Unknown | 0 | | C:/.../wat...E563.raw | not available | raw | Unknown | Unknown | 0 | | C:/.../wat...E564.raw | not available | raw | Unknown | Unknown | 0 | | C:/.../wat...E565.raw | not available | raw | Unknown | Unknown | 0 | | C:/.../wat...E566.raw | not available | raw | Unknown | Unknown | 0 | | C:/.../wat...E567.raw | not available | raw | Unknown | Unknown | 0 | | C:/.../wat...E568.raw | not available | raw | Unknown | Unknown | 0 | | C:/.../wat...E569.raw | not available | raw | Unknown | Unknown | 0 | | C:/.../wat...E570.raw | not available | raw | Unknown | Unknown | 0 | | C:/.../wat...E571.raw | not available | raw | Unknown | Unknown | 0 | | C:/.../wat...E572.raw | not available | raw | Unknown | Unknown | 0 | | C:/.../wat...E573.raw | not available | raw | Unknown | Unknown | 0 | | C:/.../wat...E574.raw | not available | raw | Unknown | Unknown | 0 | | C:/.../wat...E575.raw | not available | raw | Unknown | Unknown | 0 | | C:/.../wat...E576.raw | not available | raw | Unknown | Unknown | 0 | | C:/.../wat...E577.raw | not available | raw | Unknown | Unknown | 0 | | C:/.../wat...E578.raw | not available | raw | Unknown | Unknown | 0 | | C:/.../wat...E579.raw | not available | raw | Unknown | Unknown | 0 | | C:/.../wat...E580.raw | not available | raw | Unknown | Unknown | 0 | | C:/.../wat...E581.raw | not available | raw | Unknown | Unknown | 0 | | C:/.../wat...E582.raw | not available | raw | Unknown | Unknown | 0 | | C:/.../wat...E583.raw | not available | raw | Unknown | Unknown | 0 | | C:/.../wat...E584.raw | not available | raw | Unknown | Unknown | 0 | | C:/.../wat...E585.raw | not available | raw | Unknown | Unknown | 0 | | C:/.../wat...E586.raw | not available | raw | Unknown | Unknown | 0 | | C:/.../wat...E587.raw | not available | raw | Unknown | Unknown | 0 | | C:/.../wat...E588.raw | not available | raw | Unknown | Unknown | 0 | | C:/.../wat...E589.raw | not available | raw | Unknown | Unknown | 0 | | C:/.../wat...E590.raw | not available | raw | Unknown | Unknown | 0 | | C:/.../wat...E591.raw | not available | raw | Unknown | Unknown | 0 | | C:/.../wat...E592.raw | not available | raw | Unknown | Unknown | 0 | | C:/.../wat...E593.raw | not available | raw | Unknown | Unknown | 0 | | C:/.../wat...E594.raw | not available | raw | Unknown | Unknown | 0 | | C:/.../wat...E595.raw | not available | raw | Unknown | Unknown | 0 | | C:/.../wat...E596.raw | not available | raw | Unknown | Unknown | 0 | | C:/.../wat...E597.raw | not available | raw | Unknown | Unknown | 0 | | C:/.../wat...E598.raw | not available | raw | Unknown | Unknown | 0 | | C:/.../wat...E599.raw | not available | raw | Unknown | Unknown | 0 | | C:/.../wat...E600.raw | not available | raw | Unknown | Unknown | 0 | | C:/.../wat...E601.raw | not available | raw | Unknown | Unknown | 0 | | C:/.../wat...E602.raw | not available | raw | Unknown | Unknown | 0 | | C:/.../wat...E603.raw | not available | raw | Unknown | Unknown | 0 | | C:/.../wat...E604.raw | not available | raw | Unknown | Unknown | 0 | | C:/.../wat...E605.raw | not available | raw | Unknown | Unknown | 0 | | C:/.../wat...E606.raw | not available | raw | Unknown | Unknown | 0 | | C:/.../wat...E607.raw | not available | raw | Unknown | Unknown | 0 | | C:/.../wat...E608.raw | not available | raw | Unknown | Unknown | 0 | | C:/.../wat...E609.raw | not available | raw | Unknown | Unknown | 0 | | C:/.../wat...E610.raw | not available | raw | Unknown | Unknown | 0 | | C:/.../wat...E611.raw | not available | raw | Unknown | Unknown | 0 | | C:/.../wat...E612.raw | not available | raw | Unknown | Unknown | 0 | | C:/.../wat...E613.raw | not available | raw | Unknown | Unknown | 0 | | C:/.../wat...E614.raw | not available | raw | Unknown | Unknown | 0 | | C:/.../wat...E615.raw | not available | raw | Unknown | Unknown | 0 | | C:/.../wat...E616.raw | not available | raw | Unknown | Unknown | 0 | | C:/.../wat...E617.raw | not available | raw | Unknown | Unknown | 0 | | C:/.../wat...E618.raw | not available | raw | Unknown | Unknown | 0 | | C:/.../wat...E619.raw | not available | raw | Unknown | Unknown | 0 | | C:/.../wat...E620.raw | not available | raw | Unknown | Unknown | 0 | | C:/.../wat...E621.raw | not available | raw | Unknown | Unknown | 0 | | C:/.../wat...E622.raw | not available | raw | Unknown | Unknown | 0 | | C:/.../wat...E623.raw | not available | raw | Unknown | Unknown | 0 | | C:/.../wat...E624.raw | not available | raw | Unknown | Unknown | 0 | | C:/.../wat...E625.raw | not available | raw | Unknown | Unknown | 0 | | C:/.../wat...E626.raw | not available | raw | Unknown | Unknown | 0 | | C:/.../wat...E627.raw | not available | raw | Unknown | Unknown | 0 | | C:/.../wat...E628.raw | not available | raw | Unknown | Unknown | 0 | | C:/.../wat...E629.raw | not available | raw | Unknown | Unknown | 0 | | C:/.../wat...E630.raw | not available | raw | Unknown | Unknown | 0 | | C:/.../wat...E631.raw | not available | raw | Unknown | Unknown | 0 | | C:/.../wat...E632.raw | not available | raw | Unknown | Unknown | 0 | | C:/.../wat...E633.raw | not available | raw | Unknown | Unknown | 0 | | C:/.../wat...E634.raw | not available | raw | Unknown | Unknown | 0 | | C:/.../wat...E635.raw | not available | raw | Unknown | Unknown | 0 | | C:/.../wat...E636.raw | not available | raw | Unknown | Unknown | 0 | | C:/.../wat...E637.raw | not available | raw | Unknown | Unknown | 0 | | C:/.../wat...E638.raw | not available | raw | Unknown | Unknown | 0 | | C:/.../wat...E639.raw | not available | raw | Unknown | Unknown | 0 | | C:/.../wat...E640.raw | not available | raw | Unknown | Unknown | 0 | | C:/.../wat...E641.raw | not available | raw | Unknown | Unknown | 0 | | C:/.../wat...E642.raw | not available | raw | Unknown | Unknown | 0 | | C:/.../wat...E643.raw | not available | raw | Unknown | Unknown | 0 | | C:/.../wat...E644.raw | not available | raw | Unknown | Unknown | 0 | | C:/.../wat...E645.raw | not available | raw | Unknown | Unknown | 0 | | C:/.../wat...E646.raw | not available | raw | Unknown | Unknown | 0 | | C:/.../wat...E647.raw | not available | raw | Unknown | Unknown | 0 | | C:/.../wat...E648.raw | not available | raw | Unknown | Unknown | 0 | | C:/.../wat...E649.raw | not available | raw | Unknown | Unknown | 0 | | C:/.../wat...E650.raw | not available | raw | Unknown | Unknown | 0 | | C:/.../wat...E651.raw | not available | raw | Unknown | Unknown | 0 | | C:/.../wat...E652.raw | not available | raw | Unknown | Unknown | 0 | | C:/.../wat...E653.raw | not available | raw | Unknown | Unknown | 0 | | C:/.../wat...E654.raw | not available | raw | Unknown | Unknown | 0 | | C:/.../wat...E655.raw | not available | raw | Unknown | Unknown | 0 | | C:/.../wat...E656.raw | not available | raw | Unknown | Unknown | 0 | | C:/.../wat...E657.raw | not available | raw | Unknown | Unknown | 0 | | C:/.../wat...E658.raw | not available | raw | Unknown | Unknown | 0 |  Scanner Manufacturer Information |  |  | | --- | --- | | Name: |  | | Adresse: |  | | Internetseite: |  | | Scanner: |  | | Scansoftware: |  |  Component Information |  |  | | --- | --- | | Beschreibung: |  | | Losnummer: |  | | Seriennummer: |  |  Scan Information |  |  | | --- | --- | | Röhrenspannung: |  | | Röhrenstromstärke: |  | | Scandauer: |  | | Rekonstruktionsdauer: |  | | Gesamtverarbeitungszeit: |  | | Rekonstruktionsalgorithmus: |  | | Scanmethode: |  | | Geometrie: |  | | Integrationszeit: |  | | Filtern: |  | | Projektionsanzahl: |  | | Datum, Zeit: |  | | Benutzer: |  |  Reconstruction Parameters NO RECONSTRUCTION PARAMETERS AVAILABLE  Import Settings |  |  |  |  | | --- | --- | --- | --- | | Source/Type: | Reconstructed Volume | | | | Name: | skelett aus Volumen 1 | | | | Axes swap mode: | XYZT | | | | Mirror axes: | None | | | | Data mapping: | Ramp | | | | Data range source mapping: | -1...1 | | | | Data range destination mapping: | -1...1 | | | | Data type mapping: | Unknown | | | | Voxel skip: | 0 | 0 | 0 | | Auto region of interest: | Off | | Region of interest (min): | 0 | 0 | 0 | | Region of interest (max): | -1 | -1 | -1 | | Slice interpolation mode: | Off | | | | Slice interpolation threshold: | 0 | | | | Resolution [mm]: | 1 | 1 | 1 | | Resampling mode: | Off | | | | Auto histogram mode: | Off | | Lower auto histogram boundary at (%): | 0 | | Upper auto histogram boundary at (%): | 0 |  Analysis Information |  |  | | --- | --- | | Wall thickness: | 0 | | Defect detection: | 0 | | Nominal/actual comparison: | 0 | | Number of reference objects: | 0 |  File List (678) | files (678) | dimensions | format | type | endian | header | | --- | --- | --- | --- | --- | --- | | C:/.../ske...3000.raw | not available | raw | Unknown | Unknown | 0 | | C:/.../ske...3001.raw | not available | raw | Unknown | Unknown | 0 | | C:/.../ske...3002.raw | not available | raw | Unknown | Unknown | 0 | | C:/.../ske...3003.raw | not available | raw | Unknown | Unknown | 0 | | C:/.../ske...3004.raw | not available | raw | Unknown | Unknown | 0 | | C:/.../ske...3005.raw | not available | raw | Unknown | Unknown | 0 | | C:/.../ske...3006.raw | not available | raw | Unknown | Unknown | 0 | | C:/.../ske...3007.raw | not available | raw | Unknown | Unknown | 0 | | C:/.../ske...3008.raw | not available | raw | Unknown | Unknown | 0 | | C:/.../ske...3009.raw | not available | raw | Unknown | Unknown | 0 | | C:/.../ske...3010.raw | not available | raw | Unknown | Unknown | 0 | | C:/.../ske...3011.raw | not available | raw | Unknown | Unknown | 0 | | C:/.../ske...3012.raw | not available | raw | Unknown | Unknown | 0 | | C:/.../ske...3013.raw | not available | raw | Unknown | Unknown | 0 | | C:/.../ske...3014.raw | not available | raw | Unknown | Unknown | 0 | | C:/.../ske...3015.raw | not available | raw | Unknown | Unknown | 0 | | C:/.../ske...3016.raw | not available | raw | Unknown | Unknown | 0 | | C:/.../ske...3017.raw | not available | raw | Unknown | Unknown | 0 | | C:/.../ske...3018.raw | not available | raw | Unknown | Unknown | 0 | | C:/.../ske...3019.raw | not available | raw | Unknown | Unknown | 0 | | C:/.../ske...3020.raw | not available | raw | Unknown | Unknown | 0 | | C:/.../ske...3021.raw | not available | raw | Unknown | Unknown | 0 | | C:/.../ske...3022.raw | not available | raw | Unknown | Unknown | 0 | | C:/.../ske...3023.raw | not available | raw | Unknown | Unknown | 0 | | C:/.../ske...3024.raw | not available | raw | Unknown | Unknown | 0 | | C:/.../ske...3025.raw | not available | raw | Unknown | Unknown | 0 | | C:/.../ske...3026.raw | not available | raw | Unknown | Unknown | 0 | | C:/.../ske...3027.raw | not available | raw | Unknown | Unknown | 0 | | C:/.../ske...3028.raw | not available | raw | Unknown | Unknown | 0 | | C:/.../ske...3029.raw | not available | raw | Unknown | Unknown | 0 | | C:/.../ske...3030.raw | not available | raw | Unknown | Unknown | 0 | | C:/.../ske...3031.raw | not available | raw | Unknown | Unknown | 0 | | C:/.../ske...3032.raw | not available | raw | Unknown | Unknown | 0 | | C:/.../ske...3033.raw | not available | raw | Unknown | Unknown | 0 | | C:/.../ske...3034.raw | not available | raw | Unknown | Unknown | 0 | | C:/.../ske...3035.raw | not available | raw | Unknown | Unknown | 0 | | C:/.../ske...3036.raw | not available | raw | Unknown | Unknown | 0 | | C:/.../ske...3037.raw | not available | raw | Unknown | Unknown | 0 | | C:/.../ske...3038.raw | not available | raw | Unknown | Unknown | 0 | | C:/.../ske...3039.raw | not available | raw | Unknown | Unknown | 0 | | C:/.../ske...3040.raw | not available | raw | Unknown | Unknown | 0 | | C:/.../ske...3041.raw | not available | raw | Unknown | Unknown | 0 | | C:/.../ske...3042.raw | not available | raw | Unknown | Unknown | 0 | | C:/.../ske...3043.raw | not available | raw | Unknown | Unknown | 0 | | C:/.../ske...3044.raw | not available | raw | Unknown | Unknown | 0 | | C:/.../ske...3045.raw | not available | raw | Unknown | Unknown | 0 | | C:/.../ske...3046.raw | not available | raw | Unknown | Unknown | 0 | | C:/.../ske...3047.raw | not available | raw | Unknown | Unknown | 0 | | C:/.../ske...3048.raw | not available | raw | Unknown | Unknown | 0 | | C:/.../ske...3049.raw | not available | raw | Unknown | Unknown | 0 | | C:/.../ske...3050.raw | not available | raw | Unknown | Unknown | 0 | | C:/.../ske...3051.raw | not available | raw | Unknown | Unknown | 0 | | C:/.../ske...3052.raw | not available | raw | Unknown | Unknown | 0 | | C:/.../ske...3053.raw | not available | raw | Unknown | Unknown | 0 | | C:/.../ske...3054.raw | not available | raw | Unknown | Unknown | 0 | | C:/.../ske...3055.raw | not available | raw | Unknown | Unknown | 0 | | C:/.../ske...3056.raw | not available | raw | Unknown | Unknown | 0 | | C:/.../ske...3057.raw | not available | raw | Unknown | Unknown | 0 | | C:/.../ske...3058.raw | not available | raw | Unknown | Unknown | 0 | | C:/.../ske...3059.raw | not available | raw | Unknown | Unknown | 0 | | C:/.../ske...3060.raw | not available | raw | Unknown | Unknown | 0 | | C:/.../ske...3061.raw | not available | raw | Unknown | Unknown | 0 | | C:/.../ske...3062.raw | not available | raw | Unknown | Unknown | 0 | | C:/.../ske...3063.raw | not available | raw | Unknown | Unknown | 0 | | C:/.../ske...3064.raw | not available | raw | Unknown | Unknown | 0 | | C:/.../ske...3065.raw | not available | raw | Unknown | Unknown | 0 | | C:/.../ske...3066.raw | not available | raw | Unknown | Unknown | 0 | | C:/.../ske...3067.raw | not available | raw | Unknown | Unknown | 0 | | C:/.../ske...3068.raw | not available | raw | Unknown | Unknown | 0 | | C:/.../ske...3069.raw | not available | raw | Unknown | Unknown | 0 | | C:/.../ske...3070.raw | not available | raw | Unknown | Unknown | 0 | | C:/.../ske...3071.raw | not available | raw | Unknown | Unknown | 0 | | C:/.../ske...3072.raw | not available | raw | Unknown | Unknown | 0 | | C:/.../ske...3073.raw | not available | raw | Unknown | Unknown | 0 | | C:/.../ske...3074.raw | not available | raw | Unknown | Unknown | 0 | | C:/.../ske...3075.raw | not available | raw | Unknown | Unknown | 0 | | C:/.../ske...3076.raw | not available | raw | Unknown | Unknown | 0 | | C:/.../ske...3077.raw | not available | raw | Unknown | Unknown | 0 | | C:/.../ske...3078.raw | not available | raw | Unknown | Unknown | 0 | | C:/.../ske...3079.raw | not available | raw | Unknown | Unknown | 0 | | C:/.../ske...3080.raw | not available | raw | Unknown | Unknown | 0 | | C:/.../ske...3081.raw | not available | raw | Unknown | Unknown | 0 | | C:/.../ske...3082.raw | not available | raw | Unknown | Unknown | 0 | | C:/.../ske...3083.raw | not available | raw | Unknown | Unknown | 0 | | C:/.../ske...3084.raw | not available | raw | Unknown | Unknown | 0 | | C:/.../ske...3085.raw | not available | raw | Unknown | Unknown | 0 | | C:/.../ske...3086.raw | not available | raw | Unknown | Unknown | 0 | | C:/.../ske...3087.raw | not available | raw | Unknown | Unknown | 0 | | C:/.../ske...3088.raw | not available | raw | Unknown | Unknown | 0 | | C:/.../ske...3089.raw | not available | raw | Unknown | Unknown | 0 | | C:/.../ske...3090.raw | not available | raw | Unknown | Unknown | 0 | | C:/.../ske...3091.raw | not available | raw | Unknown | Unknown | 0 | | C:/.../ske...3092.raw | not available | raw | Unknown | Unknown | 0 | | C:/.../ske...3093.raw | not available | raw | Unknown | Unknown | 0 | | C:/.../ske...3094.raw | not available | raw | Unknown | Unknown | 0 | | C:/.../ske...3095.raw | not available | raw | Unknown | Unknown | 0 | | C:/.../ske...3096.raw | not available | raw | Unknown | Unknown | 0 | | C:/.../ske...3097.raw | not available | raw | Unknown | Unknown | 0 | | C:/.../ske...3098.raw | not available | raw | Unknown | Unknown | 0 | | C:/.../ske...3099.raw | not available | raw | Unknown | Unknown | 0 | | C:/.../ske...3100.raw | not available | raw | Unknown | Unknown | 0 | | C:/.../ske...3101.raw | not available | raw | Unknown | Unknown | 0 | | C:/.../ske...3102.raw | not available | raw | Unknown | Unknown | 0 | | C:/.../ske...3103.raw | not available | raw | Unknown | Unknown | 0 | | C:/.../ske...3104.raw | not available | raw | Unknown | Unknown | 0 | | C:/.../ske...3105.raw | not available | raw | Unknown | Unknown | 0 | | C:/.../ske...3106.raw | not available | raw | Unknown | Unknown | 0 | | C:/.../ske...3107.raw | not available | raw | Unknown | Unknown | 0 | | C:/.../ske...3108.raw | not available | raw | Unknown | Unknown | 0 | | C:/.../ske...3109.raw | not available | raw | Unknown | Unknown | 0 | | C:/.../ske...3110.raw | not available | raw | Unknown | Unknown | 0 | | C:/.../ske...3111.raw | not available | raw | Unknown | Unknown | 0 | | C:/.../ske...3112.raw | not available | raw | Unknown | Unknown | 0 | | C:/.../ske...3113.raw | not available | raw | Unknown | Unknown | 0 | | C:/.../ske...3114.raw | not available | raw | Unknown | Unknown | 0 | | C:/.../ske...3115.raw | not available | raw | Unknown | Unknown | 0 | | C:/.../ske...3116.raw | not available | raw | Unknown | Unknown | 0 | | C:/.../ske...3117.raw | not available | raw | Unknown | Unknown | 0 | | C:/.../ske...3118.raw | not available | raw | Unknown | Unknown | 0 | | C:/.../ske...3119.raw | not available | raw | Unknown | Unknown | 0 | | C:/.../ske...3120.raw | not available | raw | Unknown | Unknown | 0 | | C:/.../ske...3121.raw | not available | raw | Unknown | Unknown | 0 | | C:/.../ske...3122.raw | not available | raw | Unknown | Unknown | 0 | | C:/.../ske...3123.raw | not available | raw | Unknown | Unknown | 0 | | C:/.../ske...3124.raw | not available | raw | Unknown | Unknown | 0 | | C:/.../ske...3125.raw | not available | raw | Unknown | Unknown | 0 | | C:/.../ske...3126.raw | not available | raw | Unknown | Unknown | 0 | | C:/.../ske...3127.raw | not available | raw | Unknown | Unknown | 0 | | C:/.../ske...3128.raw | not available | raw | Unknown | Unknown | 0 | | C:/.../ske...3129.raw | not available | raw | Unknown | Unknown | 0 | | C:/.../ske...3130.raw | not available | raw | Unknown | Unknown | 0 | | C:/.../ske...3131.raw | not available | raw | Unknown | Unknown | 0 | | C:/.../ske...3132.raw | not available | raw | Unknown | Unknown | 0 | | C:/.../ske...3133.raw | not available | raw | Unknown | Unknown | 0 | | C:/.../ske...3134.raw | not available | raw | Unknown | Unknown | 0 | | C:/.../ske...3135.raw | not available | raw | Unknown | Unknown | 0 | | C:/.../ske...3136.raw | not available | raw | Unknown | Unknown | 0 | | C:/.../ske...3137.raw | not available | raw | Unknown | Unknown | 0 | | C:/.../ske...3138.raw | not available | raw | Unknown | Unknown | 0 | | C:/.../ske...3139.raw | not available | raw | Unknown | Unknown | 0 | | C:/.../ske...3140.raw | not available | raw | Unknown | Unknown | 0 | | C:/.../ske...3141.raw | not available | raw | Unknown | Unknown | 0 | | C:/.../ske...3142.raw | not available | raw | Unknown | Unknown | 0 | | C:/.../ske...3143.raw | not available | raw | Unknown | Unknown | 0 | | C:/.../ske...3144.raw | not available | raw | Unknown | Unknown | 0 | | C:/.../ske...3145.raw | not available | raw | Unknown | Unknown | 0 | | C:/.../ske...3146.raw | not available | raw | Unknown | Unknown | 0 | | C:/.../ske...3147.raw | not available | raw | Unknown | Unknown | 0 | | C:/.../ske...3148.raw | not available | raw | Unknown | Unknown | 0 | | C:/.../ske...3149.raw | not available | raw | Unknown | Unknown | 0 | | C:/.../ske...3150.raw | not available | raw | Unknown | Unknown | 0 | | C:/.../ske...3151.raw | not available | raw | Unknown | Unknown | 0 | | C:/.../ske...3152.raw | not available | raw | Unknown | Unknown | 0 | | C:/.../ske...3153.raw | not available | raw | Unknown | Unknown | 0 | | C:/.../ske...3154.raw | not available | raw | Unknown | Unknown | 0 | | C:/.../ske...3155.raw | not available | raw | Unknown | Unknown | 0 | | C:/.../ske...3156.raw | not available | raw | Unknown | Unknown | 0 | | C:/.../ske...3157.raw | not available | raw | Unknown | Unknown | 0 | | C:/.../ske...3158.raw | not available | raw | Unknown | Unknown | 0 | | C:/.../ske...3159.raw | not available | raw | Unknown | Unknown | 0 | | C:/.../ske...3160.raw | not available | raw | Unknown | Unknown | 0 | | C:/.../ske...3161.raw | not available | raw | Unknown | Unknown | 0 | | C:/.../ske...3162.raw | not available | raw | Unknown | Unknown | 0 | | C:/.../ske...3163.raw | not available | raw | Unknown | Unknown | 0 | | C:/.../ske...3164.raw | not available | raw | Unknown | Unknown | 0 | | C:/.../ske...3165.raw | not available | raw | Unknown | Unknown | 0 | | C:/.../ske...3166.raw | not available | raw | Unknown | Unknown | 0 | | C:/.../ske...3167.raw | not available | raw | Unknown | Unknown | 0 | | C:/.../ske...3168.raw | not available | raw | Unknown | Unknown | 0 | | C:/.../ske...3169.raw | not available | raw | Unknown | Unknown | 0 | | C:/.../ske...3170.raw | not available | raw | Unknown | Unknown | 0 | | C:/.../ske...3171.raw | not available | raw | Unknown | Unknown | 0 | | C:/.../ske...3172.raw | not available | raw | Unknown | Unknown | 0 | | C:/.../ske...3173.raw | not available | raw | Unknown | Unknown | 0 | | C:/.../ske...3174.raw | not available | raw | Unknown | Unknown | 0 | | C:/.../ske...3175.raw | not available | raw | Unknown | Unknown | 0 | | C:/.../ske...3176.raw | not available | raw | Unknown | Unknown | 0 | | C:/.../ske...3177.raw | not available | raw | Unknown | Unknown | 0 | | C:/.../ske...3178.raw | not available | raw | Unknown | Unknown | 0 | | C:/.../ske...3179.raw | not available | raw | Unknown | Unknown | 0 | | C:/.../ske...3180.raw | not available | raw | Unknown | Unknown | 0 | | C:/.../ske...3181.raw | not available | raw | Unknown | Unknown | 0 | | C:/.../ske...3182.raw | not available | raw | Unknown | Unknown | 0 | | C:/.../ske...3183.raw | not available | raw | Unknown | Unknown | 0 | | C:/.../ske...3184.raw | not available | raw | Unknown | Unknown | 0 | | C:/.../ske...3185.raw | not available | raw | Unknown | Unknown | 0 | | C:/.../ske...3186.raw | not available | raw | Unknown | Unknown | 0 | | C:/.../ske...3187.raw | not available | raw | Unknown | Unknown | 0 | | C:/.../ske...3188.raw | not available | raw | Unknown | Unknown | 0 | | C:/.../ske...3189.raw | not available | raw | Unknown | Unknown | 0 | | C:/.../ske...3190.raw | not available | raw | Unknown | Unknown | 0 | | C:/.../ske...3191.raw | not available | raw | Unknown | Unknown | 0 | | C:/.../ske...3192.raw | not available | raw | Unknown | Unknown | 0 | | C:/.../ske...3193.raw | not available | raw | Unknown | Unknown | 0 | | C:/.../ske...3194.raw | not available | raw | Unknown | Unknown | 0 | | C:/.../ske...3195.raw | not available | raw | Unknown | Unknown | 0 | | C:/.../ske...3196.raw | not available | raw | Unknown | Unknown | 0 | | C:/.../ske...3197.raw | not available | raw | Unknown | Unknown | 0 | | C:/.../ske...3198.raw | not available | raw | Unknown | Unknown | 0 | | C:/.../ske...3199.raw | not available | raw | Unknown | Unknown | 0 | | C:/.../ske...3200.raw | not available | raw | Unknown | Unknown | 0 | | C:/.../ske...3201.raw | not available | raw | Unknown | Unknown | 0 | | C:/.../ske...3202.raw | not available | raw | Unknown | Unknown | 0 | | C:/.../ske...3203.raw | not available | raw | Unknown | Unknown | 0 | | C:/.../ske...3204.raw | not available | raw | Unknown | Unknown | 0 | | C:/.../ske...3205.raw | not available | raw | Unknown | Unknown | 0 | | C:/.../ske...3206.raw | not available | raw | Unknown | Unknown | 0 | | C:/.../ske...3207.raw | not available | raw | Unknown | Unknown | 0 | | C:/.../ske...3208.raw | not available | raw | Unknown | Unknown | 0 | | C:/.../ske...3209.raw | not available | raw | Unknown | Unknown | 0 | | C:/.../ske...3210.raw | not available | raw | Unknown | Unknown | 0 | | C:/.../ske...3211.raw | not available | raw | Unknown | Unknown | 0 | | C:/.../ske...3212.raw | not available | raw | Unknown | Unknown | 0 | | C:/.../ske...3213.raw | not available | raw | Unknown | Unknown | 0 | | C:/.../ske...3214.raw | not available | raw | Unknown | Unknown | 0 | | C:/.../ske...3215.raw | not available | raw | Unknown | Unknown | 0 | | C:/.../ske...3216.raw | not available | raw | Unknown | Unknown | 0 | | C:/.../ske...3217.raw | not available | raw | Unknown | Unknown | 0 | | C:/.../ske...3218.raw | not available | raw | Unknown | Unknown | 0 | | C:/.../ske...3219.raw | not available | raw | Unknown | Unknown | 0 | | C:/.../ske...3220.raw | not available | raw | Unknown | Unknown | 0 | | C:/.../ske...3221.raw | not available | raw | Unknown | Unknown | 0 | | C:/.../ske...3222.raw | not available | raw | Unknown | Unknown | 0 | | C:/.../ske...3223.raw | not available | raw | Unknown | Unknown | 0 | | C:/.../ske...3224.raw | not available | raw | Unknown | Unknown | 0 | | C:/.../ske...3225.raw | not available | raw | Unknown | Unknown | 0 | | C:/.../ske...3226.raw | not available | raw | Unknown | Unknown | 0 | | C:/.../ske...3227.raw | not available | raw | Unknown | Unknown | 0 | | C:/.../ske...3228.raw | not available | raw | Unknown | Unknown | 0 | | C:/.../ske...3229.raw | not available | raw | Unknown | Unknown | 0 | | C:/.../ske...3230.raw | not available | raw | Unknown | Unknown | 0 | | C:/.../ske...3231.raw | not available | raw | Unknown | Unknown | 0 | | C:/.../ske...3232.raw | not available | raw | Unknown | Unknown | 0 | | C:/.../ske...3233.raw | not available | raw | Unknown | Unknown | 0 | | C:/.../ske...3234.raw | not available | raw | Unknown | Unknown | 0 | | C:/.../ske...3235.raw | not available | raw | Unknown | Unknown | 0 | | C:/.../ske...3236.raw | not available | raw | Unknown | Unknown | 0 | | C:/.../ske...3237.raw | not available | raw | Unknown | Unknown | 0 | | C:/.../ske...3238.raw | not available | raw | Unknown | Unknown | 0 | | C:/.../ske...3239.raw | not available | raw | Unknown | Unknown | 0 | | C:/.../ske...3240.raw | not available | raw | Unknown | Unknown | 0 | | C:/.../ske...3241.raw | not available | raw | Unknown | Unknown | 0 | | C:/.../ske...3242.raw | not available | raw | Unknown | Unknown | 0 | | C:/.../ske...3243.raw | not available | raw | Unknown | Unknown | 0 | | C:/.../ske...3244.raw | not available | raw | Unknown | Unknown | 0 | | C:/.../ske...3245.raw | not available | raw | Unknown | Unknown | 0 | | C:/.../ske...3246.raw | not available | raw | Unknown | Unknown | 0 | | C:/.../ske...3247.raw | not available | raw | Unknown | Unknown | 0 | | C:/.../ske...3248.raw | not available | raw | Unknown | Unknown | 0 | | C:/.../ske...3249.raw | not available | raw | Unknown | Unknown | 0 | | C:/.../ske...3250.raw | not available | raw | Unknown | Unknown | 0 | | C:/.../ske...3251.raw | not available | raw | Unknown | Unknown | 0 | | C:/.../ske...3252.raw | not available | raw | Unknown | Unknown | 0 | | C:/.../ske...3253.raw | not available | raw | Unknown | Unknown | 0 | | C:/.../ske...3254.raw | not available | raw | Unknown | Unknown | 0 | | C:/.../ske...3255.raw | not available | raw | Unknown | Unknown | 0 | | C:/.../ske...3256.raw | not available | raw | Unknown | Unknown | 0 | | C:/.../ske...3257.raw | not available | raw | Unknown | Unknown | 0 | | C:/.../ske...3258.raw | not available | raw | Unknown | Unknown | 0 | | C:/.../ske...3259.raw | not available | raw | Unknown | Unknown | 0 | | C:/.../ske...3260.raw | not available | raw | Unknown | Unknown | 0 | | C:/.../ske...3261.raw | not available | raw | Unknown | Unknown | 0 | | C:/.../ske...3262.raw | not available | raw | Unknown | Unknown | 0 | | C:/.../ske...3263.raw | not available | raw | Unknown | Unknown | 0 | | C:/.../ske...3264.raw | not available | raw | Unknown | Unknown | 0 | | C:/.../ske...3265.raw | not available | raw | Unknown | Unknown | 0 | | C:/.../ske...3266.raw | not available | raw | Unknown | Unknown | 0 | | C:/.../ske...3267.raw | not available | raw | Unknown | Unknown | 0 | | C:/.../ske...3268.raw | not available | raw | Unknown | Unknown | 0 | | C:/.../ske...3269.raw | not available | raw | Unknown | Unknown | 0 | | C:/.../ske...3270.raw | not available | raw | Unknown | Unknown | 0 | | C:/.../ske...3271.raw | not available | raw | Unknown | Unknown | 0 | | C:/.../ske...3272.raw | not available | raw | Unknown | Unknown | 0 | | C:/.../ske...3273.raw | not available | raw | Unknown | Unknown | 0 | | C:/.../ske...3274.raw | not available | raw | Unknown | Unknown | 0 | | C:/.../ske...3275.raw | not available | raw | Unknown | Unknown | 0 | | C:/.../ske...3276.raw | not available | raw | Unknown | Unknown | 0 | | C:/.../ske...3277.raw | not available | raw | Unknown | Unknown | 0 | | C:/.../ske...3278.raw | not available | raw | Unknown | Unknown | 0 | | C:/.../ske...3279.raw | not available | raw | Unknown | Unknown | 0 | | C:/.../ske...3280.raw | not available | raw | Unknown | Unknown | 0 | | C:/.../ske...3281.raw | not available | raw | Unknown | Unknown | 0 | | C:/.../ske...3282.raw | not available | raw | Unknown | Unknown | 0 | | C:/.../ske...3283.raw | not available | raw | Unknown | Unknown | 0 | | C:/.../ske...3284.raw | not available | raw | Unknown | Unknown | 0 | | C:/.../ske...3285.raw | not available | raw | Unknown | Unknown | 0 | | C:/.../ske...3286.raw | not available | raw | Unknown | Unknown | 0 | | C:/.../ske...3287.raw | not available | raw | Unknown | Unknown | 0 | | C:/.../ske...3288.raw | not available | raw | Unknown | Unknown | 0 | | C:/.../ske...3289.raw | not available | raw | Unknown | Unknown | 0 | | C:/.../ske...3290.raw | not available | raw | Unknown | Unknown | 0 | | C:/.../ske...3291.raw | not available | raw | Unknown | Unknown | 0 | | C:/.../ske...3292.raw | not available | raw | Unknown | Unknown | 0 | | C:/.../ske...3293.raw | not available | raw | Unknown | Unknown | 0 | | C:/.../ske...3294.raw | not available | raw | Unknown | Unknown | 0 | | C:/.../ske...3295.raw | not available | raw | Unknown | Unknown | 0 | | C:/.../ske...3296.raw | not available | raw | Unknown | Unknown | 0 | | C:/.../ske...3297.raw | not available | raw | Unknown | Unknown | 0 | | C:/.../ske...3298.raw | not available | raw | Unknown | Unknown | 0 | | C:/.../ske...3299.raw | not available | raw | Unknown | Unknown | 0 | | C:/.../ske...3300.raw | not available | raw | Unknown | Unknown | 0 | | C:/.../ske...3301.raw | not available | raw | Unknown | Unknown | 0 | | C:/.../ske...3302.raw | not available | raw | Unknown | Unknown | 0 | | C:/.../ske...3303.raw | not available | raw | Unknown | Unknown | 0 | | C:/.../ske...3304.raw | not available | raw | Unknown | Unknown | 0 | | C:/.../ske...3305.raw | not available | raw | Unknown | Unknown | 0 | | C:/.../ske...3306.raw | not available | raw | Unknown | Unknown | 0 | | C:/.../ske...3307.raw | not available | raw | Unknown | Unknown | 0 | | C:/.../ske...3308.raw | not available | raw | Unknown | Unknown | 0 | | C:/.../ske...3309.raw | not available | raw | Unknown | Unknown | 0 | | C:/.../ske...3310.raw | not available | raw | Unknown | Unknown | 0 | | C:/.../ske...3311.raw | not available | raw | Unknown | Unknown | 0 | | C:/.../ske...3312.raw | not available | raw | Unknown | Unknown | 0 | | C:/.../ske...3313.raw | not available | raw | Unknown | Unknown | 0 | | C:/.../ske...3314.raw | not available | raw | Unknown | Unknown | 0 | | C:/.../ske...3315.raw | not available | raw | Unknown | Unknown | 0 | | C:/.../ske...3316.raw | not available | raw | Unknown | Unknown | 0 | | C:/.../ske...3317.raw | not available | raw | Unknown | Unknown | 0 | | C:/.../ske...3318.raw | not available | raw | Unknown | Unknown | 0 | | C:/.../ske...3319.raw | not available | raw | Unknown | Unknown | 0 | | C:/.../ske...3320.raw | not available | raw | Unknown | Unknown | 0 | | C:/.../ske...3321.raw | not available | raw | Unknown | Unknown | 0 | | C:/.../ske...3322.raw | not available | raw | Unknown | Unknown | 0 | | C:/.../ske...3323.raw | not available | raw | Unknown | Unknown | 0 | | C:/.../ske...3324.raw | not available | raw | Unknown | Unknown | 0 | | C:/.../ske...3325.raw | not available | raw | Unknown | Unknown | 0 | | C:/.../ske...3326.raw | not available | raw | Unknown | Unknown | 0 | | C:/.../ske...3327.raw | not available | raw | Unknown | Unknown | 0 | | C:/.../ske...3328.raw | not available | raw | Unknown | Unknown | 0 | | C:/.../ske...3329.raw | not available | raw | Unknown | Unknown | 0 | | C:/.../ske...3330.raw | not available | raw | Unknown | Unknown | 0 | | C:/.../ske...3331.raw | not available | raw | Unknown | Unknown | 0 | | C:/.../ske...3332.raw | not available | raw | Unknown | Unknown | 0 | | C:/.../ske...3333.raw | not available | raw | Unknown | Unknown | 0 | | C:/.../ske...3334.raw | not available | raw | Unknown | Unknown | 0 | | C:/.../ske...3335.raw | not available | raw | Unknown | Unknown | 0 | | C:/.../ske...3336.raw | not available | raw | Unknown | Unknown | 0 | | C:/.../ske...3337.raw | not available | raw | Unknown | Unknown | 0 | | C:/.../ske...3338.raw | not available | raw | Unknown | Unknown | 0 | | C:/.../ske...3339.raw | not available | raw | Unknown | Unknown | 0 | | C:/.../ske...3340.raw | not available | raw | Unknown | Unknown | 0 | | C:/.../ske...3341.raw | not available | raw | Unknown | Unknown | 0 | | C:/.../ske...3342.raw | not available | raw | Unknown | Unknown | 0 | | C:/.../ske...3343.raw | not available | raw | Unknown | Unknown | 0 | | C:/.../ske...3344.raw | not available | raw | Unknown | Unknown | 0 | | C:/.../ske...3345.raw | not available | raw | Unknown | Unknown | 0 | | C:/.../ske...3346.raw | not available | raw | Unknown | Unknown | 0 | | C:/.../ske...3347.raw | not available | raw | Unknown | Unknown | 0 | | C:/.../ske...3348.raw | not available | raw | Unknown | Unknown | 0 | | C:/.../ske...3349.raw | not available | raw | Unknown | Unknown | 0 | | C:/.../ske...3350.raw | not available | raw | Unknown | Unknown | 0 | | C:/.../ske...3351.raw | not available | raw | Unknown | Unknown | 0 | | C:/.../ske...3352.raw | not available | raw | Unknown | Unknown | 0 | | C:/.../ske...3353.raw | not available | raw | Unknown | Unknown | 0 | | C:/.../ske...3354.raw | not available | raw | Unknown | Unknown | 0 | | C:/.../ske...3355.raw | not available | raw | Unknown | Unknown | 0 | | C:/.../ske...3356.raw | not available | raw | Unknown | Unknown | 0 | | C:/.../ske...3357.raw | not available | raw | Unknown | Unknown | 0 | | C:/.../ske...3358.raw | not available | raw | Unknown | Unknown | 0 | | C:/.../ske...3359.raw | not available | raw | Unknown | Unknown | 0 | | C:/.../ske...3360.raw | not available | raw | Unknown | Unknown | 0 | | C:/.../ske...3361.raw | not available | raw | Unknown | Unknown | 0 | | C:/.../ske...3362.raw | not available | raw | Unknown | Unknown | 0 | | C:/.../ske...3363.raw | not available | raw | Unknown | Unknown | 0 | | C:/.../ske...3364.raw | not available | raw | Unknown | Unknown | 0 | | C:/.../ske...3365.raw | not available | raw | Unknown | Unknown | 0 | | C:/.../ske...3366.raw | not available | raw | Unknown | Unknown | 0 | | C:/.../ske...3367.raw | not available | raw | Unknown | Unknown | 0 | | C:/.../ske...3368.raw | not available | raw | Unknown | Unknown | 0 | | C:/.../ske...3369.raw | not available | raw | Unknown | Unknown | 0 | | C:/.../ske...3370.raw | not available | raw | Unknown | Unknown | 0 | | C:/.../ske...3371.raw | not available | raw | Unknown | Unknown | 0 | | C:/.../ske...3372.raw | not available | raw | Unknown | Unknown | 0 | | C:/.../ske...3373.raw | not available | raw | Unknown | Unknown | 0 | | C:/.../ske...3374.raw | not available | raw | Unknown | Unknown | 0 | | C:/.../ske...3375.raw | not available | raw | Unknown | Unknown | 0 | | C:/.../ske...3376.raw | not available | raw | Unknown | Unknown | 0 | | C:/.../ske...3377.raw | not available | raw | Unknown | Unknown | 0 | | C:/.../ske...3378.raw | not available | raw | Unknown | Unknown | 0 | | C:/.../ske...3379.raw | not available | raw | Unknown | Unknown | 0 | | C:/.../ske...3380.raw | not available | raw | Unknown | Unknown | 0 | | C:/.../ske...3381.raw | not available | raw | Unknown | Unknown | 0 | | C:/.../ske...3382.raw | not available | raw | Unknown | Unknown | 0 | | C:/.../ske...3383.raw | not available | raw | Unknown | Unknown | 0 | | C:/.../ske...3384.raw | not available | raw | Unknown | Unknown | 0 | | C:/.../ske...3385.raw | not available | raw | Unknown | Unknown | 0 | | C:/.../ske...3386.raw | not available | raw | Unknown | Unknown | 0 | | C:/.../ske...3387.raw | not available | raw | Unknown | Unknown | 0 | | C:/.../ske...3388.raw | not available | raw | Unknown | Unknown | 0 | | C:/.../ske...3389.raw | not available | raw | Unknown | Unknown | 0 | | C:/.../ske...3390.raw | not available | raw | Unknown | Unknown | 0 | | C:/.../ske...3391.raw | not available | raw | Unknown | Unknown | 0 | | C:/.../ske...3392.raw | not available | raw | Unknown | Unknown | 0 | | C:/.../ske...3393.raw | not available | raw | Unknown | Unknown | 0 | | C:/.../ske...3394.raw | not available | raw | Unknown | Unknown | 0 | | C:/.../ske...3395.raw | not available | raw | Unknown | Unknown | 0 | | C:/.../ske...3396.raw | not available | raw | Unknown | Unknown | 0 | | C:/.../ske...3397.raw | not available | raw | Unknown | Unknown | 0 | | C:/.../ske...3398.raw | not available | raw | Unknown | Unknown | 0 | | C:/.../ske...3399.raw | not available | raw | Unknown | Unknown | 0 | | C:/.../ske...3400.raw | not available | raw | Unknown | Unknown | 0 | | C:/.../ske...3401.raw | not available | raw | Unknown | Unknown | 0 | | C:/.../ske...3402.raw | not available | raw | Unknown | Unknown | 0 | | C:/.../ske...3403.raw | not available | raw | Unknown | Unknown | 0 | | C:/.../ske...3404.raw | not available | raw | Unknown | Unknown | 0 | | C:/.../ske...3405.raw | not available | raw | Unknown | Unknown | 0 | | C:/.../ske...3406.raw | not available | raw | Unknown | Unknown | 0 | | C:/.../ske...3407.raw | not available | raw | Unknown | Unknown | 0 | | C:/.../ske...3408.raw | not available | raw | Unknown | Unknown | 0 | | C:/.../ske...3409.raw | not available | raw | Unknown | Unknown | 0 | | C:/.../ske...3410.raw | not available | raw | Unknown | Unknown | 0 | | C:/.../ske...3411.raw | not available | raw | Unknown | Unknown | 0 | | C:/.../ske...3412.raw | not available | raw | Unknown | Unknown | 0 | | C:/.../ske...3413.raw | not available | raw | Unknown | Unknown | 0 | | C:/.../ske...3414.raw | not available | raw | Unknown | Unknown | 0 | | C:/.../ske...3415.raw | not available | raw | Unknown | Unknown | 0 | | C:/.../ske...3416.raw | not available | raw | Unknown | Unknown | 0 | | C:/.../ske...3417.raw | not available | raw | Unknown | Unknown | 0 | | C:/.../ske...3418.raw | not available | raw | Unknown | Unknown | 0 | | C:/.../ske...3419.raw | not available | raw | Unknown | Unknown | 0 | | C:/.../ske...3420.raw | not available | raw | Unknown | Unknown | 0 | | C:/.../ske...3421.raw | not available | raw | Unknown | Unknown | 0 | | C:/.../ske...3422.raw | not available | raw | Unknown | Unknown | 0 | | C:/.../ske...3423.raw | not available | raw | Unknown | Unknown | 0 | | C:/.../ske...3424.raw | not available | raw | Unknown | Unknown | 0 | | C:/.../ske...3425.raw | not available | raw | Unknown | Unknown | 0 | | C:/.../ske...3426.raw | not available | raw | Unknown | Unknown | 0 | | C:/.../ske...3427.raw | not available | raw | Unknown | Unknown | 0 | | C:/.../ske...3428.raw | not available | raw | Unknown | Unknown | 0 | | C:/.../ske...3429.raw | not available | raw | Unknown | Unknown | 0 | | C:/.../ske...3430.raw | not available | raw | Unknown | Unknown | 0 | | C:/.../ske...3431.raw | not available | raw | Unknown | Unknown | 0 | | C:/.../ske...3432.raw | not available | raw | Unknown | Unknown | 0 | | C:/.../ske...3433.raw | not available | raw | Unknown | Unknown | 0 | | C:/.../ske...3434.raw | not available | raw | Unknown | Unknown | 0 | | C:/.../ske...3435.raw | not available | raw | Unknown | Unknown | 0 | | C:/.../ske...3436.raw | not available | raw | Unknown | Unknown | 0 | | C:/.../ske...3437.raw | not available | raw | Unknown | Unknown | 0 | | C:/.../ske...3438.raw | not available | raw | Unknown | Unknown | 0 | | C:/.../ske...3439.raw | not available | raw | Unknown | Unknown | 0 | | C:/.../ske...3440.raw | not available | raw | Unknown | Unknown | 0 | | C:/.../ske...3441.raw | not available | raw | Unknown | Unknown | 0 | | C:/.../ske...3442.raw | not available | raw | Unknown | Unknown | 0 | | C:/.../ske...3443.raw | not available | raw | Unknown | Unknown | 0 | | C:/.../ske...3444.raw | not available | raw | Unknown | Unknown | 0 | | C:/.../ske...3445.raw | not available | raw | Unknown | Unknown | 0 | | C:/.../ske...3446.raw | not available | raw | Unknown | Unknown | 0 | | C:/.../ske...3447.raw | not available | raw | Unknown | Unknown | 0 | | C:/.../ske...3448.raw | not available | raw | Unknown | Unknown | 0 | | C:/.../ske...3449.raw | not available | raw | Unknown | Unknown | 0 | | C:/.../ske...3450.raw | not available | raw | Unknown | Unknown | 0 | | C:/.../ske...3451.raw | not available | raw | Unknown | Unknown | 0 | | C:/.../ske...3452.raw | not available | raw | Unknown | Unknown | 0 | | C:/.../ske...3453.raw | not available | raw | Unknown | Unknown | 0 | | C:/.../ske...3454.raw | not available | raw | Unknown | Unknown | 0 | | C:/.../ske...3455.raw | not available | raw | Unknown | Unknown | 0 | | C:/.../ske...3456.raw | not available | raw | Unknown | Unknown | 0 | | C:/.../ske...3457.raw | not available | raw | Unknown | Unknown | 0 | | C:/.../ske...3458.raw | not available | raw | Unknown | Unknown | 0 | | C:/.../ske...3459.raw | not available | raw | Unknown | Unknown | 0 | | C:/.../ske...3460.raw | not available | raw | Unknown | Unknown | 0 | | C:/.../ske...3461.raw | not available | raw | Unknown | Unknown | 0 | | C:/.../ske...3462.raw | not available | raw | Unknown | Unknown | 0 | | C:/.../ske...3463.raw | not available | raw | Unknown | Unknown | 0 | | C:/.../ske...3464.raw | not available | raw | Unknown | Unknown | 0 | | C:/.../ske...3465.raw | not available | raw | Unknown | Unknown | 0 | | C:/.../ske...3466.raw | not available | raw | Unknown | Unknown | 0 | | C:/.../ske...3467.raw | not available | raw | Unknown | Unknown | 0 | | C:/.../ske...3468.raw | not available | raw | Unknown | Unknown | 0 | | C:/.../ske...3469.raw | not available | raw | Unknown | Unknown | 0 | | C:/.../ske...3470.raw | not available | raw | Unknown | Unknown | 0 | | C:/.../ske...3471.raw | not available | raw | Unknown | Unknown | 0 | | C:/.../ske...3472.raw | not available | raw | Unknown | Unknown | 0 | | C:/.../ske...3473.raw | not available | raw | Unknown | Unknown | 0 | | C:/.../ske...3474.raw | not available | raw | Unknown | Unknown | 0 | | C:/.../ske...3475.raw | not available | raw | Unknown | Unknown | 0 | | C:/.../ske...3476.raw | not available | raw | Unknown | Unknown | 0 | | C:/.../ske...3477.raw | not available | raw | Unknown | Unknown | 0 | | C:/.../ske...3478.raw | not available | raw | Unknown | Unknown | 0 | | C:/.../ske...3479.raw | not available | raw | Unknown | Unknown | 0 | | C:/.../ske...3480.raw | not available | raw | Unknown | Unknown | 0 | | C:/.../ske...3481.raw | not available | raw | Unknown | Unknown | 0 | | C:/.../ske...3482.raw | not available | raw | Unknown | Unknown | 0 | | C:/.../ske...3483.raw | not available | raw | Unknown | Unknown | 0 | | C:/.../ske...3484.raw | not available | raw | Unknown | Unknown | 0 | | C:/.../ske...3485.raw | not available | raw | Unknown | Unknown | 0 | | C:/.../ske...3486.raw | not available | raw | Unknown | Unknown | 0 | | C:/.../ske...3487.raw | not available | raw | Unknown | Unknown | 0 | | C:/.../ske...3488.raw | not available | raw | Unknown | Unknown | 0 | | C:/.../ske...3489.raw | not available | raw | Unknown | Unknown | 0 | | C:/.../ske...3490.raw | not available | raw | Unknown | Unknown | 0 | | C:/.../ske...3491.raw | not available | raw | Unknown | Unknown | 0 | | C:/.../ske...3492.raw | not available | raw | Unknown | Unknown | 0 | | C:/.../ske...3493.raw | not available | raw | Unknown | Unknown | 0 | | C:/.../ske...3494.raw | not available | raw | Unknown | Unknown | 0 | | C:/.../ske...3495.raw | not available | raw | Unknown | Unknown | 0 | | C:/.../ske...3496.raw | not available | raw | Unknown | Unknown | 0 | | C:/.../ske...3497.raw | not available | raw | Unknown | Unknown | 0 | | C:/.../ske...3498.raw | not available | raw | Unknown | Unknown | 0 | | C:/.../ske...3499.raw | not available | raw | Unknown | Unknown | 0 | | C:/.../ske...3500.raw | not available | raw | Unknown | Unknown | 0 | | C:/.../ske...3501.raw | not available | raw | Unknown | Unknown | 0 | | C:/.../ske...3502.raw | not available | raw | Unknown | Unknown | 0 | | C:/.../ske...3503.raw | not available | raw | Unknown | Unknown | 0 | | C:/.../ske...3504.raw | not available | raw | Unknown | Unknown | 0 | | C:/.../ske...3505.raw | not available | raw | Unknown | Unknown | 0 | | C:/.../ske...3506.raw | not available | raw | Unknown | Unknown | 0 | | C:/.../ske...3507.raw | not available | raw | Unknown | Unknown | 0 | | C:/.../ske...3508.raw | not available | raw | Unknown | Unknown | 0 | | C:/.../ske...3509.raw | not available | raw | Unknown | Unknown | 0 | | C:/.../ske...3510.raw | not available | raw | Unknown | Unknown | 0 | | C:/.../ske...3511.raw | not available | raw | Unknown | Unknown | 0 | | C:/.../ske...3512.raw | not available | raw | Unknown | Unknown | 0 | | C:/.../ske...3513.raw | not available | raw | Unknown | Unknown | 0 | | C:/.../ske...3514.raw | not available | raw | Unknown | Unknown | 0 | | C:/.../ske...3515.raw | not available | raw | Unknown | Unknown | 0 | | C:/.../ske...3516.raw | not available | raw | Unknown | Unknown | 0 | | C:/.../ske...3517.raw | not available | raw | Unknown | Unknown | 0 | | C:/.../ske...3518.raw | not available | raw | Unknown | Unknown | 0 | | C:/.../ske...3519.raw | not available | raw | Unknown | Unknown | 0 | | C:/.../ske...3520.raw | not available | raw | Unknown | Unknown | 0 | | C:/.../ske...3521.raw | not available | raw | Unknown | Unknown | 0 | | C:/.../ske...3522.raw | not available | raw | Unknown | Unknown | 0 | | C:/.../ske...3523.raw | not available | raw | Unknown | Unknown | 0 | | C:/.../ske...3524.raw | not available | raw | Unknown | Unknown | 0 | | C:/.../ske...3525.raw | not available | raw | Unknown | Unknown | 0 | | C:/.../ske...3526.raw | not available | raw | Unknown | Unknown | 0 | | C:/.../ske...3527.raw | not available | raw | Unknown | Unknown | 0 | | C:/.../ske...3528.raw | not available | raw | Unknown | Unknown | 0 | | C:/.../ske...3529.raw | not available | raw | Unknown | Unknown | 0 | | C:/.../ske...3530.raw | not available | raw | Unknown | Unknown | 0 | | C:/.../ske...3531.raw | not available | raw | Unknown | Unknown | 0 | | C:/.../ske...3532.raw | not available | raw | Unknown | Unknown | 0 | | C:/.../ske...3533.raw | not available | raw | Unknown | Unknown | 0 | | C:/.../ske...3534.raw | not available | raw | Unknown | Unknown | 0 | | C:/.../ske...3535.raw | not available | raw | Unknown | Unknown | 0 | | C:/.../ske...3536.raw | not available | raw | Unknown | Unknown | 0 | | C:/.../ske...3537.raw | not available | raw | Unknown | Unknown | 0 | | C:/.../ske...3538.raw | not available | raw | Unknown | Unknown | 0 | | C:/.../ske...3539.raw | not available | raw | Unknown | Unknown | 0 | | C:/.../ske...3540.raw | not available | raw | Unknown | Unknown | 0 | | C:/.../ske...3541.raw | not available | raw | Unknown | Unknown | 0 | | C:/.../ske...3542.raw | not available | raw | Unknown | Unknown | 0 | | C:/.../ske...3543.raw | not available | raw | Unknown | Unknown | 0 | | C:/.../ske...3544.raw | not available | raw | Unknown | Unknown | 0 | | C:/.../ske...3545.raw | not available | raw | Unknown | Unknown | 0 | | C:/.../ske...3546.raw | not available | raw | Unknown | Unknown | 0 | | C:/.../ske...3547.raw | not available | raw | Unknown | Unknown | 0 | | C:/.../ske...3548.raw | not available | raw | Unknown | Unknown | 0 | | C:/.../ske...3549.raw | not available | raw | Unknown | Unknown | 0 | | C:/.../ske...3550.raw | not available | raw | Unknown | Unknown | 0 | | C:/.../ske...3551.raw | not available | raw | Unknown | Unknown | 0 | | C:/.../ske...3552.raw | not available | raw | Unknown | Unknown | 0 | | C:/.../ske...3553.raw | not available | raw | Unknown | Unknown | 0 | | C:/.../ske...3554.raw | not available | raw | Unknown | Unknown | 0 | | C:/.../ske...3555.raw | not available | raw | Unknown | Unknown | 0 | | C:/.../ske...3556.raw | not available | raw | Unknown | Unknown | 0 | | C:/.../ske...3557.raw | not available | raw | Unknown | Unknown | 0 | | C:/.../ske...3558.raw | not available | raw | Unknown | Unknown | 0 | | C:/.../ske...3559.raw | not available | raw | Unknown | Unknown | 0 | | C:/.../ske...3560.raw | not available | raw | Unknown | Unknown | 0 | | C:/.../ske...3561.raw | not available | raw | Unknown | Unknown | 0 | | C:/.../ske...3562.raw | not available | raw | Unknown | Unknown | 0 | | C:/.../ske...3563.raw | not available | raw | Unknown | Unknown | 0 | | C:/.../ske...3564.raw | not available | raw | Unknown | Unknown | 0 | | C:/.../ske...3565.raw | not available | raw | Unknown | Unknown | 0 | | C:/.../ske...3566.raw | not available | raw | Unknown | Unknown | 0 | | C:/.../ske...3567.raw | not available | raw | Unknown | Unknown | 0 | | C:/.../ske...3568.raw | not available | raw | Unknown | Unknown | 0 | | C:/.../ske...3569.raw | not available | raw | Unknown | Unknown | 0 | | C:/.../ske...3570.raw | not available | raw | Unknown | Unknown | 0 | | C:/.../ske...3571.raw | not available | raw | Unknown | Unknown | 0 | | C:/.../ske...3572.raw | not available | raw | Unknown | Unknown | 0 | | C:/.../ske...3573.raw | not available | raw | Unknown | Unknown | 0 | | C:/.../ske...3574.raw | not available | raw | Unknown | Unknown | 0 | | C:/.../ske...3575.raw | not available | raw | Unknown | Unknown | 0 | | C:/.../ske...3576.raw | not available | raw | Unknown | Unknown | 0 | | C:/.../ske...3577.raw | not available | raw | Unknown | Unknown | 0 | | C:/.../ske...3578.raw | not available | raw | Unknown | Unknown | 0 | | C:/.../ske...3579.raw | not available | raw | Unknown | Unknown | 0 | | C:/.../ske...3580.raw | not available | raw | Unknown | Unknown | 0 | | C:/.../ske...3581.raw | not available | raw | Unknown | Unknown | 0 | | C:/.../ske...3582.raw | not available | raw | Unknown | Unknown | 0 | | C:/.../ske...3583.raw | not available | raw | Unknown | Unknown | 0 | | C:/.../ske...3584.raw | not available | raw | Unknown | Unknown | 0 | | C:/.../ske...3585.raw | not available | raw | Unknown | Unknown | 0 | | C:/.../ske...3586.raw | not available | raw | Unknown | Unknown | 0 | | C:/.../ske...3587.raw | not available | raw | Unknown | Unknown | 0 | | C:/.../ske...3588.raw | not available | raw | Unknown | Unknown | 0 | | C:/.../ske...3589.raw | not available | raw | Unknown | Unknown | 0 | | C:/.../ske...3590.raw | not available | raw | Unknown | Unknown | 0 | | C:/.../ske...3591.raw | not available | raw | Unknown | Unknown | 0 | | C:/.../ske...3592.raw | not available | raw | Unknown | Unknown | 0 | | C:/.../ske...3593.raw | not available | raw | Unknown | Unknown | 0 | | C:/.../ske...3594.raw | not available | raw | Unknown | Unknown | 0 | | C:/.../ske...3595.raw | not available | raw | Unknown | Unknown | 0 | | C:/.../ske...3596.raw | not available | raw | Unknown | Unknown | 0 | | C:/.../ske...3597.raw | not available | raw | Unknown | Unknown | 0 | | C:/.../ske...3598.raw | not available | raw | Unknown | Unknown | 0 | | C:/.../ske...3599.raw | not available | raw | Unknown | Unknown | 0 | | C:/.../ske...3600.raw | not available | raw | Unknown | Unknown | 0 | | C:/.../ske...3601.raw | not available | raw | Unknown | Unknown | 0 | | C:/.../ske...3602.raw | not available | raw | Unknown | Unknown | 0 | | C:/.../ske...3603.raw | not available | raw | Unknown | Unknown | 0 | | C:/.../ske...3604.raw | not available | raw | Unknown | Unknown | 0 | | C:/.../ske...3605.raw | not available | raw | Unknown | Unknown | 0 | | C:/.../ske...3606.raw | not available | raw | Unknown | Unknown | 0 | | C:/.../ske...3607.raw | not available | raw | Unknown | Unknown | 0 | | C:/.../ske...3608.raw | not available | raw | Unknown | Unknown | 0 | | C:/.../ske...3609.raw | not available | raw | Unknown | Unknown | 0 | | C:/.../ske...3610.raw | not available | raw | Unknown | Unknown | 0 | | C:/.../ske...3611.raw | not available | raw | Unknown | Unknown | 0 | | C:/.../ske...3612.raw | not available | raw | Unknown | Unknown | 0 | | C:/.../ske...3613.raw | not available | raw | Unknown | Unknown | 0 | | C:/.../ske...3614.raw | not available | raw | Unknown | Unknown | 0 | | C:/.../ske...3615.raw | not available | raw | Unknown | Unknown | 0 | | C:/.../ske...3616.raw | not available | raw | Unknown | Unknown | 0 | | C:/.../ske...3617.raw | not available | raw | Unknown | Unknown | 0 | | C:/.../ske...3618.raw | not available | raw | Unknown | Unknown | 0 | | C:/.../ske...3619.raw | not available | raw | Unknown | Unknown | 0 | | C:/.../ske...3620.raw | not available | raw | Unknown | Unknown | 0 | | C:/.../ske...3621.raw | not available | raw | Unknown | Unknown | 0 | | C:/.../ske...3622.raw | not available | raw | Unknown | Unknown | 0 | | C:/.../ske...3623.raw | not available | raw | Unknown | Unknown | 0 | | C:/.../ske...3624.raw | not available | raw | Unknown | Unknown | 0 | | C:/.../ske...3625.raw | not available | raw | Unknown | Unknown | 0 | | C:/.../ske...3626.raw | not available | raw | Unknown | Unknown | 0 | | C:/.../ske...3627.raw | not available | raw | Unknown | Unknown | 0 | | C:/.../ske...3628.raw | not available | raw | Unknown | Unknown | 0 | | C:/.../ske...3629.raw | not available | raw | Unknown | Unknown | 0 | | C:/.../ske...3630.raw | not available | raw | Unknown | Unknown | 0 | | C:/.../ske...3631.raw | not available | raw | Unknown | Unknown | 0 | | C:/.../ske...3632.raw | not available | raw | Unknown | Unknown | 0 | | C:/.../ske...3633.raw | not available | raw | Unknown | Unknown | 0 | | C:/.../ske...3634.raw | not available | raw | Unknown | Unknown | 0 | | C:/.../ske...3635.raw | not available | raw | Unknown | Unknown | 0 | | C:/.../ske...3636.raw | not available | raw | Unknown | Unknown | 0 | | C:/.../ske...3637.raw | not available | raw | Unknown | Unknown | 0 | | C:/.../ske...3638.raw | not available | raw | Unknown | Unknown | 0 | | C:/.../ske...3639.raw | not available | raw | Unknown | Unknown | 0 | | C:/.../ske...3640.raw | not available | raw | Unknown | Unknown | 0 | | C:/.../ske...3641.raw | not available | raw | Unknown | Unknown | 0 | | C:/.../ske...3642.raw | not available | raw | Unknown | Unknown | 0 | | C:/.../ske...3643.raw | not available | raw | Unknown | Unknown | 0 | | C:/.../ske...3644.raw | not available | raw | Unknown | Unknown | 0 | | C:/.../ske...3645.raw | not available | raw | Unknown | Unknown | 0 | | C:/.../ske...3646.raw | not available | raw | Unknown | Unknown | 0 | | C:/.../ske...3647.raw | not available | raw | Unknown | Unknown | 0 | | C:/.../ske...3648.raw | not available | raw | Unknown | Unknown | 0 | | C:/.../ske...3649.raw | not available | raw | Unknown | Unknown | 0 | | C:/.../ske...3650.raw | not available | raw | Unknown | Unknown | 0 | | C:/.../ske...3651.raw | not available | raw | Unknown | Unknown | 0 | | C:/.../ske...3652.raw | not available | raw | Unknown | Unknown | 0 | | C:/.../ske...3653.raw | not available | raw | Unknown | Unknown | 0 | | C:/.../ske...3654.raw | not available | raw | Unknown | Unknown | 0 | | C:/.../ske...3655.raw | not available | raw | Unknown | Unknown | 0 | | C:/.../ske...3656.raw | not available | raw | Unknown | Unknown | 0 | | C:/.../ske...3657.raw | not available | raw | Unknown | Unknown | 0 | | C:/.../ske...3658.raw | not available | raw | Unknown | Unknown | 0 | | C:/.../ske...3659.raw | not available | raw | Unknown | Unknown | 0 | | C:/.../ske...3660.raw | not available | raw | Unknown | Unknown | 0 | | C:/.../ske...3661.raw | not available | raw | Unknown | Unknown | 0 | | C:/.../ske...3662.raw | not available | raw | Unknown | Unknown | 0 | | C:/.../ske...3663.raw | not available | raw | Unknown | Unknown | 0 | | C:/.../ske...3664.raw | not available | raw | Unknown | Unknown | 0 | | C:/.../ske...3665.raw | not available | raw | Unknown | Unknown | 0 | | C:/.../ske...3666.raw | not available | raw | Unknown | Unknown | 0 | | C:/.../ske...3667.raw | not available | raw | Unknown | Unknown | 0 | | C:/.../ske...3668.raw | not available | raw | Unknown | Unknown | 0 | | C:/.../ske...3669.raw | not available | raw | Unknown | Unknown | 0 | | C:/.../ske...3670.raw | not available | raw | Unknown | Unknown | 0 | | C:/.../ske...3671.raw | not available | raw | Unknown | Unknown | 0 | | C:/.../ske...3672.raw | not available | raw | Unknown | Unknown | 0 | | C:/.../ske...3673.raw | not available | raw | Unknown | Unknown | 0 | | C:/.../ske...3674.raw | not available | raw | Unknown | Unknown | 0 | | C:/.../ske...3675.raw | not available | raw | Unknown | Unknown | 0 | | C:/.../ske...3676.raw | not available | raw | Unknown | Unknown | 0 | | C:/.../ske...3677.raw | not available | raw | Unknown | Unknown | 0 |  Scanner Manufacturer Information |  |  | | --- | --- | | Name: |  | | Adresse: |  | | Internetseite: |  | | Scanner: |  | | Scansoftware: |  |  Component Information |  |  | | --- | --- | | Beschreibung: |  | | Losnummer: |  | | Seriennummer: |  |  Scan Information |  |  | | --- | --- | | Röhrenspannung: |  | | Röhrenstromstärke: |  | | Scandauer: |  | | Rekonstruktionsdauer: |  | | Gesamtverarbeitungszeit: |  | | Rekonstruktionsalgorithmus: |  | | Scanmethode: |  | | Geometrie: |  | | Integrationszeit: |  | | Filtern: |  | | Projektionsanzahl: |  | | Datum, Zeit: |  | | Benutzer: |  |  Reconstruction Parameters NO RECONSTRUCTION PARAMETERS AVAILABLE  Import Settings |  |  |  |  | | --- | --- | --- | --- | | Source/Type: | Reconstructed Volume | | | | Name: | Braunes Fett aus Volumen 1 | | | | Axes swap mode: | XYZT | | | | Mirror axes: | None | | | | Data mapping: | Ramp | | | | Data range source mapping: | -1...1 | | | | Data range destination mapping: | -1...1 | | | | Data type mapping: | Unknown | | | | Voxel skip: | 0 | 0 | 0 | | Auto region of interest: | Off | | Region of interest (min): | 0 | 0 | 0 | | Region of interest (max): | -1 | -1 | -1 | | Slice interpolation mode: | Off | | | | Slice interpolation threshold: | 0 | | | | Resolution [mm]: | 1 | 1 | 1 | | Resampling mode: | Off | | | | Auto histogram mode: | Off | | Lower auto histogram boundary at (%): | 0 | | Upper auto histogram boundary at (%): | 0 |  Analysis Information |  |  | | --- | --- | | Wall thickness: | 0 | | Defect detection: | 0 | | Nominal/actual comparison: | 0 | | Number of reference objects: | 0 |  File List (486) | files (486) | dimensions | format | type | endian | header | | --- | --- | --- | --- | --- | --- | | C:/.../bat...E000.raw | not available | raw | Unknown | Unknown | 0 | | C:/.../bat...E001.raw | not available | raw | Unknown | Unknown | 0 | | C:/.../bat...E002.raw | not available | raw | Unknown | Unknown | 0 | | C:/.../bat...E003.raw | not available | raw | Unknown | Unknown | 0 | | C:/.../bat...E004.raw | not available | raw | Unknown | Unknown | 0 | | C:/.../bat...E005.raw | not available | raw | Unknown | Unknown | 0 | | C:/.../bat...E006.raw | not available | raw | Unknown | Unknown | 0 | | C:/.../bat...E007.raw | not available | raw | Unknown | Unknown | 0 | | C:/.../bat...E008.raw | not available | raw | Unknown | Unknown | 0 | | C:/.../bat...E009.raw | not available | raw | Unknown | Unknown | 0 | | C:/.../bat...E010.raw | not available | raw | Unknown | Unknown | 0 | | C:/.../bat...E011.raw | not available | raw | Unknown | Unknown | 0 | | C:/.../bat...E012.raw | not available | raw | Unknown | Unknown | 0 | | C:/.../bat...E013.raw | not available | raw | Unknown | Unknown | 0 | | C:/.../bat...E014.raw | not available | raw | Unknown | Unknown | 0 | | C:/.../bat...E015.raw | not available | raw | Unknown | Unknown | 0 | | C:/.../bat...E016.raw | not available | raw | Unknown | Unknown | 0 | | C:/.../bat...E017.raw | not available | raw | Unknown | Unknown | 0 | | C:/.../bat...E018.raw | not available | raw | Unknown | Unknown | 0 | | C:/.../bat...E019.raw | not available | raw | Unknown | Unknown | 0 | | C:/.../bat...E020.raw | not available | raw | Unknown | Unknown | 0 | | C:/.../bat...E021.raw | not available | raw | Unknown | Unknown | 0 | | C:/.../bat...E022.raw | not available | raw | Unknown | Unknown | 0 | | C:/.../bat...E023.raw | not available | raw | Unknown | Unknown | 0 | | C:/.../bat...E024.raw | not available | raw | Unknown | Unknown | 0 | | C:/.../bat...E025.raw | not available | raw | Unknown | Unknown | 0 | | C:/.../bat...E026.raw | not available | raw | Unknown | Unknown | 0 | | C:/.../bat...E027.raw | not available | raw | Unknown | Unknown | 0 | | C:/.../bat...E028.raw | not available | raw | Unknown | Unknown | 0 | | C:/.../bat...E029.raw | not available | raw | Unknown | Unknown | 0 | | C:/.../bat...E030.raw | not available | raw | Unknown | Unknown | 0 | | C:/.../bat...E031.raw | not available | raw | Unknown | Unknown | 0 | | C:/.../bat...E032.raw | not available | raw | Unknown | Unknown | 0 | | C:/.../bat...E033.raw | not available | raw | Unknown | Unknown | 0 | | C:/.../bat...E034.raw | not available | raw | Unknown | Unknown | 0 | | C:/.../bat...E035.raw | not available | raw | Unknown | Unknown | 0 | | C:/.../bat...E036.raw | not available | raw | Unknown | Unknown | 0 | | C:/.../bat...E037.raw | not available | raw | Unknown | Unknown | 0 | | C:/.../bat...E038.raw | not available | raw | Unknown | Unknown | 0 | | C:/.../bat...E039.raw | not available | raw | Unknown | Unknown | 0 | | C:/.../bat...E040.raw | not available | raw | Unknown | Unknown | 0 | | C:/.../bat...E041.raw | not available | raw | Unknown | Unknown | 0 | | C:/.../bat...E042.raw | not available | raw | Unknown | Unknown | 0 | | C:/.../bat...E043.raw | not available | raw | Unknown | Unknown | 0 | | C:/.../bat...E044.raw | not available | raw | Unknown | Unknown | 0 | | C:/.../bat...E045.raw | not available | raw | Unknown | Unknown | 0 | | C:/.../bat...E046.raw | not available | raw | Unknown | Unknown | 0 | | C:/.../bat...E047.raw | not available | raw | Unknown | Unknown | 0 | | C:/.../bat...E048.raw | not available | raw | Unknown | Unknown | 0 | | C:/.../bat...E049.raw | not available | raw | Unknown | Unknown | 0 | | C:/.../bat...E050.raw | not available | raw | Unknown | Unknown | 0 | | C:/.../bat...E051.raw | not available | raw | Unknown | Unknown | 0 | | C:/.../bat...E052.raw | not available | raw | Unknown | Unknown | 0 | | C:/.../bat...E053.raw | not available | raw | Unknown | Unknown | 0 | | C:/.../bat...E054.raw | not available | raw | Unknown | Unknown | 0 | | C:/.../bat...E055.raw | not available | raw | Unknown | Unknown | 0 | | C:/.../bat...E056.raw | not available | raw | Unknown | Unknown | 0 | | C:/.../bat...E057.raw | not available | raw | Unknown | Unknown | 0 | | C:/.../bat...E058.raw | not available | raw | Unknown | Unknown | 0 | | C:/.../bat...E059.raw | not available | raw | Unknown | Unknown | 0 | | C:/.../bat...E060.raw | not available | raw | Unknown | Unknown | 0 | | C:/.../bat...E061.raw | not available | raw | Unknown | Unknown | 0 | | C:/.../bat...E062.raw | not available | raw | Unknown | Unknown | 0 | | C:/.../bat...E063.raw | not available | raw | Unknown | Unknown | 0 | | C:/.../bat...E064.raw | not available | raw | Unknown | Unknown | 0 | | C:/.../bat...E065.raw | not available | raw | Unknown | Unknown | 0 | | C:/.../bat...E066.raw | not available | raw | Unknown | Unknown | 0 | | C:/.../bat...E067.raw | not available | raw | Unknown | Unknown | 0 | | C:/.../bat...E068.raw | not available | raw | Unknown | Unknown | 0 | | C:/.../bat...E069.raw | not available | raw | Unknown | Unknown | 0 | | C:/.../bat...E070.raw | not available | raw | Unknown | Unknown | 0 | | C:/.../bat...E071.raw | not available | raw | Unknown | Unknown | 0 | | C:/.../bat...E072.raw | not available | raw | Unknown | Unknown | 0 | | C:/.../bat...E073.raw | not available | raw | Unknown | Unknown | 0 | | C:/.../bat...E074.raw | not available | raw | Unknown | Unknown | 0 | | C:/.../bat...E075.raw | not available | raw | Unknown | Unknown | 0 | | C:/.../bat...E076.raw | not available | raw | Unknown | Unknown | 0 | | C:/.../bat...E077.raw | not available | raw | Unknown | Unknown | 0 | | C:/.../bat...E078.raw | not available | raw | Unknown | Unknown | 0 | | C:/.../bat...E079.raw | not available | raw | Unknown | Unknown | 0 | | C:/.../bat...E080.raw | not available | raw | Unknown | Unknown | 0 | | C:/.../bat...E081.raw | not available | raw | Unknown | Unknown | 0 | | C:/.../bat...E082.raw | not available | raw | Unknown | Unknown | 0 | | C:/.../bat...E083.raw | not available | raw | Unknown | Unknown | 0 | | C:/.../bat...E084.raw | not available | raw | Unknown | Unknown | 0 | | C:/.../bat...E085.raw | not available | raw | Unknown | Unknown | 0 | | C:/.../bat...E086.raw | not available | raw | Unknown | Unknown | 0 | | C:/.../bat...E087.raw | not available | raw | Unknown | Unknown | 0 | | C:/.../bat...E088.raw | not available | raw | Unknown | Unknown | 0 | | C:/.../bat...E089.raw | not available | raw | Unknown | Unknown | 0 | | C:/.../bat...E090.raw | not available | raw | Unknown | Unknown | 0 | | C:/.../bat...E091.raw | not available | raw | Unknown | Unknown | 0 | | C:/.../bat...E092.raw | not available | raw | Unknown | Unknown | 0 | | C:/.../bat...E093.raw | not available | raw | Unknown | Unknown | 0 | | C:/.../bat...E094.raw | not available | raw | Unknown | Unknown | 0 | | C:/.../bat...E095.raw | not available | raw | Unknown | Unknown | 0 | | C:/.../bat...E096.raw | not available | raw | Unknown | Unknown | 0 | | C:/.../bat...E097.raw | not available | raw | Unknown | Unknown | 0 | | C:/.../bat...E098.raw | not available | raw | Unknown | Unknown | 0 | | C:/.../bat...E099.raw | not available | raw | Unknown | Unknown | 0 | | C:/.../bat...E100.raw | not available | raw | Unknown | Unknown | 0 | | C:/.../bat...E101.raw | not available | raw | Unknown | Unknown | 0 | | C:/.../bat...E102.raw | not available | raw | Unknown | Unknown | 0 | | C:/.../bat...E103.raw | not available | raw | Unknown | Unknown | 0 | | C:/.../bat...E104.raw | not available | raw | Unknown | Unknown | 0 | | C:/.../bat...E105.raw | not available | raw | Unknown | Unknown | 0 | | C:/.../bat...E106.raw | not available | raw | Unknown | Unknown | 0 | | C:/.../bat...E107.raw | not available | raw | Unknown | Unknown | 0 | | C:/.../bat...E108.raw | not available | raw | Unknown | Unknown | 0 | | C:/.../bat...E109.raw | not available | raw | Unknown | Unknown | 0 | | C:/.../bat...E110.raw | not available | raw | Unknown | Unknown | 0 | | C:/.../bat...E111.raw | not available | raw | Unknown | Unknown | 0 | | C:/.../bat...E112.raw | not available | raw | Unknown | Unknown | 0 | | C:/.../bat...E113.raw | not available | raw | Unknown | Unknown | 0 | | C:/.../bat...E114.raw | not available | raw | Unknown | Unknown | 0 | | C:/.../bat...E115.raw | not available | raw | Unknown | Unknown | 0 | | C:/.../bat...E116.raw | not available | raw | Unknown | Unknown | 0 | | C:/.../bat...E117.raw | not available | raw | Unknown | Unknown | 0 | | C:/.../bat...E118.raw | not available | raw | Unknown | Unknown | 0 | | C:/.../bat...E119.raw | not available | raw | Unknown | Unknown | 0 | | C:/.../bat...E120.raw | not available | raw | Unknown | Unknown | 0 | | C:/.../bat...E121.raw | not available | raw | Unknown | Unknown | 0 | | C:/.../bat...E122.raw | not available | raw | Unknown | Unknown | 0 | | C:/.../bat...E123.raw | not available | raw | Unknown | Unknown | 0 | | C:/.../bat...E124.raw | not available | raw | Unknown | Unknown | 0 | | C:/.../bat...E125.raw | not available | raw | Unknown | Unknown | 0 | | C:/.../bat...E126.raw | not available | raw | Unknown | Unknown | 0 | | C:/.../bat...E127.raw | not available | raw | Unknown | Unknown | 0 | | C:/.../bat...E128.raw | not available | raw | Unknown | Unknown | 0 | | C:/.../bat...E129.raw | not available | raw | Unknown | Unknown | 0 | | C:/.../bat...E130.raw | not available | raw | Unknown | Unknown | 0 | | C:/.../bat...E131.raw | not available | raw | Unknown | Unknown | 0 | | C:/.../bat...E132.raw | not available | raw | Unknown | Unknown | 0 | | C:/.../bat...E133.raw | not available | raw | Unknown | Unknown | 0 | | C:/.../bat...E134.raw | not available | raw | Unknown | Unknown | 0 | | C:/.../bat...E135.raw | not available | raw | Unknown | Unknown | 0 | | C:/.../bat...E136.raw | not available | raw | Unknown | Unknown | 0 | | C:/.../bat...E137.raw | not available | raw | Unknown | Unknown | 0 | | C:/.../bat...E138.raw | not available | raw | Unknown | Unknown | 0 | | C:/.../bat...E139.raw | not available | raw | Unknown | Unknown | 0 | | C:/.../bat...E140.raw | not available | raw | Unknown | Unknown | 0 | | C:/.../bat...E141.raw | not available | raw | Unknown | Unknown | 0 | | C:/.../bat...E142.raw | not available | raw | Unknown | Unknown | 0 | | C:/.../bat...E143.raw | not available | raw | Unknown | Unknown | 0 | | C:/.../bat...E144.raw | not available | raw | Unknown | Unknown | 0 | | C:/.../bat...E145.raw | not available | raw | Unknown | Unknown | 0 | | C:/.../bat...E146.raw | not available | raw | Unknown | Unknown | 0 | | C:/.../bat...E147.raw | not available | raw | Unknown | Unknown | 0 | | C:/.../bat...E148.raw | not available | raw | Unknown | Unknown | 0 | | C:/.../bat...E149.raw | not available | raw | Unknown | Unknown | 0 | | C:/.../bat...E150.raw | not available | raw | Unknown | Unknown | 0 | | C:/.../bat...E151.raw | not available | raw | Unknown | Unknown | 0 | | C:/.../bat...E152.raw | not available | raw | Unknown | Unknown | 0 | | C:/.../bat...E153.raw | not available | raw | Unknown | Unknown | 0 | | C:/.../bat...E154.raw | not available | raw | Unknown | Unknown | 0 | | C:/.../bat...E155.raw | not available | raw | Unknown | Unknown | 0 | | C:/.../bat...E156.raw | not available | raw | Unknown | Unknown | 0 | | C:/.../bat...E157.raw | not available | raw | Unknown | Unknown | 0 | | C:/.../bat...E158.raw | not available | raw | Unknown | Unknown | 0 | | C:/.../bat...E159.raw | not available | raw | Unknown | Unknown | 0 | | C:/.../bat...E160.raw | not available | raw | Unknown | Unknown | 0 | | C:/.../bat...E161.raw | not available | raw | Unknown | Unknown | 0 | | C:/.../bat...E162.raw | not available | raw | Unknown | Unknown | 0 | | C:/.../bat...E163.raw | not available | raw | Unknown | Unknown | 0 | | C:/.../bat...E164.raw | not available | raw | Unknown | Unknown | 0 | | C:/.../bat...E165.raw | not available | raw | Unknown | Unknown | 0 | | C:/.../bat...E166.raw | not available | raw | Unknown | Unknown | 0 | | C:/.../bat...E167.raw | not available | raw | Unknown | Unknown | 0 | | C:/.../bat...E168.raw | not available | raw | Unknown | Unknown | 0 | | C:/.../bat...E169.raw | not available | raw | Unknown | Unknown | 0 | | C:/.../bat...E170.raw | not available | raw | Unknown | Unknown | 0 | | C:/.../bat...E171.raw | not available | raw | Unknown | Unknown | 0 | | C:/.../bat...E172.raw | not available | raw | Unknown | Unknown | 0 | | C:/.../bat...E173.raw | not available | raw | Unknown | Unknown | 0 | | C:/.../bat...E174.raw | not available | raw | Unknown | Unknown | 0 | | C:/.../bat...E175.raw | not available | raw | Unknown | Unknown | 0 | | C:/.../bat...E176.raw | not available | raw | Unknown | Unknown | 0 | | C:/.../bat...E177.raw | not available | raw | Unknown | Unknown | 0 | | C:/.../bat...E178.raw | not available | raw | Unknown | Unknown | 0 | | C:/.../bat...E179.raw | not available | raw | Unknown | Unknown | 0 | | C:/.../bat...E180.raw | not available | raw | Unknown | Unknown | 0 | | C:/.../bat...E181.raw | not available | raw | Unknown | Unknown | 0 | | C:/.../bat...E182.raw | not available | raw | Unknown | Unknown | 0 | | C:/.../bat...E183.raw | not available | raw | Unknown | Unknown | 0 | | C:/.../bat...E184.raw | not available | raw | Unknown | Unknown | 0 | | C:/.../bat...E185.raw | not available | raw | Unknown | Unknown | 0 | | C:/.../bat...E186.raw | not available | raw | Unknown | Unknown | 0 | | C:/.../bat...E187.raw | not available | raw | Unknown | Unknown | 0 | | C:/.../bat...E188.raw | not available | raw | Unknown | Unknown | 0 | | C:/.../bat...E189.raw | not available | raw | Unknown | Unknown | 0 | | C:/.../bat...E190.raw | not available | raw | Unknown | Unknown | 0 | | C:/.../bat...E191.raw | not available | raw | Unknown | Unknown | 0 | | C:/.../bat...E192.raw | not available | raw | Unknown | Unknown | 0 | | C:/.../bat...E193.raw | not available | raw | Unknown | Unknown | 0 | | C:/.../bat...E194.raw | not available | raw | Unknown | Unknown | 0 | | C:/.../bat...E195.raw | not available | raw | Unknown | Unknown | 0 | | C:/.../bat...E196.raw | not available | raw | Unknown | Unknown | 0 | | C:/.../bat...E197.raw | not available | raw | Unknown | Unknown | 0 | | C:/.../bat...E198.raw | not available | raw | Unknown | Unknown | 0 | | C:/.../bat...E199.raw | not available | raw | Unknown | Unknown | 0 | | C:/.../bat...E200.raw | not available | raw | Unknown | Unknown | 0 | | C:/.../bat...E201.raw | not available | raw | Unknown | Unknown | 0 | | C:/.../bat...E202.raw | not available | raw | Unknown | Unknown | 0 | | C:/.../bat...E203.raw | not available | raw | Unknown | Unknown | 0 | | C:/.../bat...E204.raw | not available | raw | Unknown | Unknown | 0 | | C:/.../bat...E205.raw | not available | raw | Unknown | Unknown | 0 | | C:/.../bat...E206.raw | not available | raw | Unknown | Unknown | 0 | | C:/.../bat...E207.raw | not available | raw | Unknown | Unknown | 0 | | C:/.../bat...E208.raw | not available | raw | Unknown | Unknown | 0 | | C:/.../bat...E209.raw | not available | raw | Unknown | Unknown | 0 | | C:/.../bat...E210.raw | not available | raw | Unknown | Unknown | 0 | | C:/.../bat...E211.raw | not available | raw | Unknown | Unknown | 0 | | C:/.../bat...E212.raw | not available | raw | Unknown | Unknown | 0 | | C:/.../bat...E213.raw | not available | raw | Unknown | Unknown | 0 | | C:/.../bat...E214.raw | not available | raw | Unknown | Unknown | 0 | | C:/.../bat...E215.raw | not available | raw | Unknown | Unknown | 0 | | C:/.../bat...E216.raw | not available | raw | Unknown | Unknown | 0 | | C:/.../bat...E217.raw | not available | raw | Unknown | Unknown | 0 | | C:/.../bat...E218.raw | not available | raw | Unknown | Unknown | 0 | | C:/.../bat...E219.raw | not available | raw | Unknown | Unknown | 0 | | C:/.../bat...E220.raw | not available | raw | Unknown | Unknown | 0 | | C:/.../bat...E221.raw | not available | raw | Unknown | Unknown | 0 | | C:/.../bat...E222.raw | not available | raw | Unknown | Unknown | 0 | | C:/.../bat...E223.raw | not available | raw | Unknown | Unknown | 0 | | C:/.../bat...E224.raw | not available | raw | Unknown | Unknown | 0 | | C:/.../bat...E225.raw | not available | raw | Unknown | Unknown | 0 | | C:/.../bat...E226.raw | not available | raw | Unknown | Unknown | 0 | | C:/.../bat...E227.raw | not available | raw | Unknown | Unknown | 0 | | C:/.../bat...E228.raw | not available | raw | Unknown | Unknown | 0 | | C:/.../bat...E229.raw | not available | raw | Unknown | Unknown | 0 | | C:/.../bat...E230.raw | not available | raw | Unknown | Unknown | 0 | | C:/.../bat...E231.raw | not available | raw | Unknown | Unknown | 0 | | C:/.../bat...E232.raw | not available | raw | Unknown | Unknown | 0 | | C:/.../bat...E233.raw | not available | raw | Unknown | Unknown | 0 | | C:/.../bat...E234.raw | not available | raw | Unknown | Unknown | 0 | | C:/.../bat...E235.raw | not available | raw | Unknown | Unknown | 0 | | C:/.../bat...E236.raw | not available | raw | Unknown | Unknown | 0 | | C:/.../bat...E237.raw | not available | raw | Unknown | Unknown | 0 | | C:/.../bat...E238.raw | not available | raw | Unknown | Unknown | 0 | | C:/.../bat...E239.raw | not available | raw | Unknown | Unknown | 0 | | C:/.../bat...E240.raw | not available | raw | Unknown | Unknown | 0 | | C:/.../bat...E241.raw | not available | raw | Unknown | Unknown | 0 | | C:/.../bat...E242.raw | not available | raw | Unknown | Unknown | 0 | | C:/.../bat...E243.raw | not available | raw | Unknown | Unknown | 0 | | C:/.../bat...E244.raw | not available | raw | Unknown | Unknown | 0 | | C:/.../bat...E245.raw | not available | raw | Unknown | Unknown | 0 | | C:/.../bat...E246.raw | not available | raw | Unknown | Unknown | 0 | | C:/.../bat...E247.raw | not available | raw | Unknown | Unknown | 0 | | C:/.../bat...E248.raw | not available | raw | Unknown | Unknown | 0 | | C:/.../bat...E249.raw | not available | raw | Unknown | Unknown | 0 | | C:/.../bat...E250.raw | not available | raw | Unknown | Unknown | 0 | | C:/.../bat...E251.raw | not available | raw | Unknown | Unknown | 0 | | C:/.../bat...E252.raw | not available | raw | Unknown | Unknown | 0 | | C:/.../bat...E253.raw | not available | raw | Unknown | Unknown | 0 | | C:/.../bat...E254.raw | not available | raw | Unknown | Unknown | 0 | | C:/.../bat...E255.raw | not available | raw | Unknown | Unknown | 0 | | C:/.../bat...E256.raw | not available | raw | Unknown | Unknown | 0 | | C:/.../bat...E257.raw | not available | raw | Unknown | Unknown | 0 | | C:/.../bat...E258.raw | not available | raw | Unknown | Unknown | 0 | | C:/.../bat...E259.raw | not available | raw | Unknown | Unknown | 0 | | C:/.../bat...E260.raw | not available | raw | Unknown | Unknown | 0 | | C:/.../bat...E261.raw | not available | raw | Unknown | Unknown | 0 | | C:/.../bat...E262.raw | not available | raw | Unknown | Unknown | 0 | | C:/.../bat...E263.raw | not available | raw | Unknown | Unknown | 0 | | C:/.../bat...E264.raw | not available | raw | Unknown | Unknown | 0 | | C:/.../bat...E265.raw | not available | raw | Unknown | Unknown | 0 | | C:/.../bat...E266.raw | not available | raw | Unknown | Unknown | 0 | | C:/.../bat...E267.raw | not available | raw | Unknown | Unknown | 0 | | C:/.../bat...E268.raw | not available | raw | Unknown | Unknown | 0 | | C:/.../bat...E269.raw | not available | raw | Unknown | Unknown | 0 | | C:/.../bat...E270.raw | not available | raw | Unknown | Unknown | 0 | | C:/.../bat...E271.raw | not available | raw | Unknown | Unknown | 0 | | C:/.../bat...E272.raw | not available | raw | Unknown | Unknown | 0 | | C:/.../bat...E273.raw | not available | raw | Unknown | Unknown | 0 | | C:/.../bat...E274.raw | not available | raw | Unknown | Unknown | 0 | | C:/.../bat...E275.raw | not available | raw | Unknown | Unknown | 0 | | C:/.../bat...E276.raw | not available | raw | Unknown | Unknown | 0 | | C:/.../bat...E277.raw | not available | raw | Unknown | Unknown | 0 | | C:/.../bat...E278.raw | not available | raw | Unknown | Unknown | 0 | | C:/.../bat...E279.raw | not available | raw | Unknown | Unknown | 0 | | C:/.../bat...E280.raw | not available | raw | Unknown | Unknown | 0 | | C:/.../bat...E281.raw | not available | raw | Unknown | Unknown | 0 | | C:/.../bat...E282.raw | not available | raw | Unknown | Unknown | 0 | | C:/.../bat...E283.raw | not available | raw | Unknown | Unknown | 0 | | C:/.../bat...E284.raw | not available | raw | Unknown | Unknown | 0 | | C:/.../bat...E285.raw | not available | raw | Unknown | Unknown | 0 | | C:/.../bat...E286.raw | not available | raw | Unknown | Unknown | 0 | | C:/.../bat...E287.raw | not available | raw | Unknown | Unknown | 0 | | C:/.../bat...E288.raw | not available | raw | Unknown | Unknown | 0 | | C:/.../bat...E289.raw | not available | raw | Unknown | Unknown | 0 | | C:/.../bat...E290.raw | not available | raw | Unknown | Unknown | 0 | | C:/.../bat...E291.raw | not available | raw | Unknown | Unknown | 0 | | C:/.../bat...E292.raw | not available | raw | Unknown | Unknown | 0 | | C:/.../bat...E293.raw | not available | raw | Unknown | Unknown | 0 | | C:/.../bat...E294.raw | not available | raw | Unknown | Unknown | 0 | | C:/.../bat...E295.raw | not available | raw | Unknown | Unknown | 0 | | C:/.../bat...E296.raw | not available | raw | Unknown | Unknown | 0 | | C:/.../bat...E297.raw | not available | raw | Unknown | Unknown | 0 | | C:/.../bat...E298.raw | not available | raw | Unknown | Unknown | 0 | | C:/.../bat...E299.raw | not available | raw | Unknown | Unknown | 0 | | C:/.../bat...E300.raw | not available | raw | Unknown | Unknown | 0 | | C:/.../bat...E301.raw | not available | raw | Unknown | Unknown | 0 | | C:/.../bat...E302.raw | not available | raw | Unknown | Unknown | 0 | | C:/.../bat...E303.raw | not available | raw | Unknown | Unknown | 0 | | C:/.../bat...E304.raw | not available | raw | Unknown | Unknown | 0 | | C:/.../bat...E305.raw | not available | raw | Unknown | Unknown | 0 | | C:/.../bat...E306.raw | not available | raw | Unknown | Unknown | 0 | | C:/.../bat...E307.raw | not available | raw | Unknown | Unknown | 0 | | C:/.../bat...E308.raw | not available | raw | Unknown | Unknown | 0 | | C:/.../bat...E309.raw | not available | raw | Unknown | Unknown | 0 | | C:/.../bat...E310.raw | not available | raw | Unknown | Unknown | 0 | | C:/.../bat...E311.raw | not available | raw | Unknown | Unknown | 0 | | C:/.../bat...E312.raw | not available | raw | Unknown | Unknown | 0 | | C:/.../bat...E313.raw | not available | raw | Unknown | Unknown | 0 | | C:/.../bat...E314.raw | not available | raw | Unknown | Unknown | 0 | | C:/.../bat...E315.raw | not available | raw | Unknown | Unknown | 0 | | C:/.../bat...E316.raw | not available | raw | Unknown | Unknown | 0 | | C:/.../bat...E317.raw | not available | raw | Unknown | Unknown | 0 | | C:/.../bat...E318.raw | not available | raw | Unknown | Unknown | 0 | | C:/.../bat...E319.raw | not available | raw | Unknown | Unknown | 0 | | C:/.../bat...E320.raw | not available | raw | Unknown | Unknown | 0 | | C:/.../bat...E321.raw | not available | raw | Unknown | Unknown | 0 | | C:/.../bat...E322.raw | not available | raw | Unknown | Unknown | 0 | | C:/.../bat...E323.raw | not available | raw | Unknown | Unknown | 0 | | C:/.../bat...E324.raw | not available | raw | Unknown | Unknown | 0 | | C:/.../bat...E325.raw | not available | raw | Unknown | Unknown | 0 | | C:/.../bat...E326.raw | not available | raw | Unknown | Unknown | 0 | | C:/.../bat...E327.raw | not available | raw | Unknown | Unknown | 0 | | C:/.../bat...E328.raw | not available | raw | Unknown | Unknown | 0 | | C:/.../bat...E329.raw | not available | raw | Unknown | Unknown | 0 | | C:/.../bat...E330.raw | not available | raw | Unknown | Unknown | 0 | | C:/.../bat...E331.raw | not available | raw | Unknown | Unknown | 0 | | C:/.../bat...E332.raw | not available | raw | Unknown | Unknown | 0 | | C:/.../bat...E333.raw | not available | raw | Unknown | Unknown | 0 | | C:/.../bat...E334.raw | not available | raw | Unknown | Unknown | 0 | | C:/.../bat...E335.raw | not available | raw | Unknown | Unknown | 0 | | C:/.../bat...E336.raw | not available | raw | Unknown | Unknown | 0 | | C:/.../bat...E337.raw | not available | raw | Unknown | Unknown | 0 | | C:/.../bat...E338.raw | not available | raw | Unknown | Unknown | 0 | | C:/.../bat...E339.raw | not available | raw | Unknown | Unknown | 0 | | C:/.../bat...E340.raw | not available | raw | Unknown | Unknown | 0 | | C:/.../bat...E341.raw | not available | raw | Unknown | Unknown | 0 | | C:/.../bat...E342.raw | not available | raw | Unknown | Unknown | 0 | | C:/.../bat...E343.raw | not available | raw | Unknown | Unknown | 0 | | C:/.../bat...E344.raw | not available | raw | Unknown | Unknown | 0 | | C:/.../bat...E345.raw | not available | raw | Unknown | Unknown | 0 | | C:/.../bat...E346.raw | not available | raw | Unknown | Unknown | 0 | | C:/.../bat...E347.raw | not available | raw | Unknown | Unknown | 0 | | C:/.../bat...E348.raw | not available | raw | Unknown | Unknown | 0 | | C:/.../bat...E349.raw | not available | raw | Unknown | Unknown | 0 | | C:/.../bat...E350.raw | not available | raw | Unknown | Unknown | 0 | | C:/.../bat...E351.raw | not available | raw | Unknown | Unknown | 0 | | C:/.../bat...E352.raw | not available | raw | Unknown | Unknown | 0 | | C:/.../bat...E353.raw | not available | raw | Unknown | Unknown | 0 | | C:/.../bat...E354.raw | not available | raw | Unknown | Unknown | 0 | | C:/.../bat...E355.raw | not available | raw | Unknown | Unknown | 0 | | C:/.../bat...E356.raw | not available | raw | Unknown | Unknown | 0 | | C:/.../bat...E357.raw | not available | raw | Unknown | Unknown | 0 | | C:/.../bat...E358.raw | not available | raw | Unknown | Unknown | 0 | | C:/.../bat...E359.raw | not available | raw | Unknown | Unknown | 0 | | C:/.../bat...E360.raw | not available | raw | Unknown | Unknown | 0 | | C:/.../bat...E361.raw | not available | raw | Unknown | Unknown | 0 | | C:/.../bat...E362.raw | not available | raw | Unknown | Unknown | 0 | | C:/.../bat...E363.raw | not available | raw | Unknown | Unknown | 0 | | C:/.../bat...E364.raw | not available | raw | Unknown | Unknown | 0 | | C:/.../bat...E365.raw | not available | raw | Unknown | Unknown | 0 | | C:/.../bat...E366.raw | not available | raw | Unknown | Unknown | 0 | | C:/.../bat...E367.raw | not available | raw | Unknown | Unknown | 0 | | C:/.../bat...E368.raw | not available | raw | Unknown | Unknown | 0 | | C:/.../bat...E369.raw | not available | raw | Unknown | Unknown | 0 | | C:/.../bat...E370.raw | not available | raw | Unknown | Unknown | 0 | | C:/.../bat...E371.raw | not available | raw | Unknown | Unknown | 0 | | C:/.../bat...E372.raw | not available | raw | Unknown | Unknown | 0 | | C:/.../bat...E373.raw | not available | raw | Unknown | Unknown | 0 | | C:/.../bat...E374.raw | not available | raw | Unknown | Unknown | 0 | | C:/.../bat...E375.raw | not available | raw | Unknown | Unknown | 0 | | C:/.../bat...E376.raw | not available | raw | Unknown | Unknown | 0 | | C:/.../bat...E377.raw | not available | raw | Unknown | Unknown | 0 | | C:/.../bat...E378.raw | not available | raw | Unknown | Unknown | 0 | | C:/.../bat...E379.raw | not available | raw | Unknown | Unknown | 0 | | C:/.../bat...E380.raw | not available | raw | Unknown | Unknown | 0 | | C:/.../bat...E381.raw | not available | raw | Unknown | Unknown | 0 | | C:/.../bat...E382.raw | not available | raw | Unknown | Unknown | 0 | | C:/.../bat...E383.raw | not available | raw | Unknown | Unknown | 0 | | C:/.../bat...E384.raw | not available | raw | Unknown | Unknown | 0 | | C:/.../bat...E385.raw | not available | raw | Unknown | Unknown | 0 | | C:/.../bat...E386.raw | not available | raw | Unknown | Unknown | 0 | | C:/.../bat...E387.raw | not available | raw | Unknown | Unknown | 0 | | C:/.../bat...E388.raw | not available | raw | Unknown | Unknown | 0 | | C:/.../bat...E389.raw | not available | raw | Unknown | Unknown | 0 | | C:/.../bat...E390.raw | not available | raw | Unknown | Unknown | 0 | | C:/.../bat...E391.raw | not available | raw | Unknown | Unknown | 0 | | C:/.../bat...E392.raw | not available | raw | Unknown | Unknown | 0 | | C:/.../bat...E393.raw | not available | raw | Unknown | Unknown | 0 | | C:/.../bat...E394.raw | not available | raw | Unknown | Unknown | 0 | | C:/.../bat...E395.raw | not available | raw | Unknown | Unknown | 0 | | C:/.../bat...E396.raw | not available | raw | Unknown | Unknown | 0 | | C:/.../bat...E397.raw | not available | raw | Unknown | Unknown | 0 | | C:/.../bat...E398.raw | not available | raw | Unknown | Unknown | 0 | | C:/.../bat...E399.raw | not available | raw | Unknown | Unknown | 0 | | C:/.../bat...E400.raw | not available | raw | Unknown | Unknown | 0 | | C:/.../bat...E401.raw | not available | raw | Unknown | Unknown | 0 | | C:/.../bat...E402.raw | not available | raw | Unknown | Unknown | 0 | | C:/.../bat...E403.raw | not available | raw | Unknown | Unknown | 0 | | C:/.../bat...E404.raw | not available | raw | Unknown | Unknown | 0 | | C:/.../bat...E405.raw | not available | raw | Unknown | Unknown | 0 | | C:/.../bat...E406.raw | not available | raw | Unknown | Unknown | 0 | | C:/.../bat...E407.raw | not available | raw | Unknown | Unknown | 0 | | C:/.../bat...E408.raw | not available | raw | Unknown | Unknown | 0 | | C:/.../bat...E409.raw | not available | raw | Unknown | Unknown | 0 | | C:/.../bat...E410.raw | not available | raw | Unknown | Unknown | 0 | | C:/.../bat...E411.raw | not available | raw | Unknown | Unknown | 0 | | C:/.../bat...E412.raw | not available | raw | Unknown | Unknown | 0 | | C:/.../bat...E413.raw | not available | raw | Unknown | Unknown | 0 | | C:/.../bat...E414.raw | not available | raw | Unknown | Unknown | 0 | | C:/.../bat...E415.raw | not available | raw | Unknown | Unknown | 0 | | C:/.../bat...E416.raw | not available | raw | Unknown | Unknown | 0 | | C:/.../bat...E417.raw | not available | raw | Unknown | Unknown | 0 | | C:/.../bat...E418.raw | not available | raw | Unknown | Unknown | 0 | | C:/.../bat...E419.raw | not available | raw | Unknown | Unknown | 0 | | C:/.../bat...E420.raw | not available | raw | Unknown | Unknown | 0 | | C:/.../bat...E421.raw | not available | raw | Unknown | Unknown | 0 | | C:/.../bat...E422.raw | not available | raw | Unknown | Unknown | 0 | | C:/.../bat...E423.raw | not available | raw | Unknown | Unknown | 0 | | C:/.../bat...E424.raw | not available | raw | Unknown | Unknown | 0 | | C:/.../bat...E425.raw | not available | raw | Unknown | Unknown | 0 | | C:/.../bat...E426.raw | not available | raw | Unknown | Unknown | 0 | | C:/.../bat...E427.raw | not available | raw | Unknown | Unknown | 0 | | C:/.../bat...E428.raw | not available | raw | Unknown | Unknown | 0 | | C:/.../bat...E429.raw | not available | raw | Unknown | Unknown | 0 | | C:/.../bat...E430.raw | not available | raw | Unknown | Unknown | 0 | | C:/.../bat...E431.raw | not available | raw | Unknown | Unknown | 0 | | C:/.../bat...E432.raw | not available | raw | Unknown | Unknown | 0 | | C:/.../bat...E433.raw | not available | raw | Unknown | Unknown | 0 | | C:/.../bat...E434.raw | not available | raw | Unknown | Unknown | 0 | | C:/.../bat...E435.raw | not available | raw | Unknown | Unknown | 0 | | C:/.../bat...E436.raw | not available | raw | Unknown | Unknown | 0 | | C:/.../bat...E437.raw | not available | raw | Unknown | Unknown | 0 | | C:/.../bat...E438.raw | not available | raw | Unknown | Unknown | 0 | | C:/.../bat...E439.raw | not available | raw | Unknown | Unknown | 0 | | C:/.../bat...E440.raw | not available | raw | Unknown | Unknown | 0 | | C:/.../bat...E441.raw | not available | raw | Unknown | Unknown | 0 | | C:/.../bat...E442.raw | not available | raw | Unknown | Unknown | 0 | | C:/.../bat...E443.raw | not available | raw | Unknown | Unknown | 0 | | C:/.../bat...E444.raw | not available | raw | Unknown | Unknown | 0 | | C:/.../bat...E445.raw | not available | raw | Unknown | Unknown | 0 | | C:/.../bat...E446.raw | not available | raw | Unknown | Unknown | 0 | | C:/.../bat...E447.raw | not available | raw | Unknown | Unknown | 0 | | C:/.../bat...E448.raw | not available | raw | Unknown | Unknown | 0 | | C:/.../bat...E449.raw | not available | raw | Unknown | Unknown | 0 | | C:/.../bat...E450.raw | not available | raw | Unknown | Unknown | 0 | | C:/.../bat...E451.raw | not available | raw | Unknown | Unknown | 0 | | C:/.../bat...E452.raw | not available | raw | Unknown | Unknown | 0 | | C:/.../bat...E453.raw | not available | raw | Unknown | Unknown | 0 | | C:/.../bat...E454.raw | not available | raw | Unknown | Unknown | 0 | | C:/.../bat...E455.raw | not available | raw | Unknown | Unknown | 0 | | C:/.../bat...E456.raw | not available | raw | Unknown | Unknown | 0 | | C:/.../bat...E457.raw | not available | raw | Unknown | Unknown | 0 | | C:/.../bat...E458.raw | not available | raw | Unknown | Unknown | 0 | | C:/.../bat...E459.raw | not available | raw | Unknown | Unknown | 0 | | C:/.../bat...E460.raw | not available | raw | Unknown | Unknown | 0 | | C:/.../bat...E461.raw | not available | raw | Unknown | Unknown | 0 | | C:/.../bat...E462.raw | not available | raw | Unknown | Unknown | 0 | | C:/.../bat...E463.raw | not available | raw | Unknown | Unknown | 0 | | C:/.../bat...E464.raw | not available | raw | Unknown | Unknown | 0 | | C:/.../bat...E465.raw | not available | raw | Unknown | Unknown | 0 | | C:/.../bat...E466.raw | not available | raw | Unknown | Unknown | 0 | | C:/.../bat...E467.raw | not available | raw | Unknown | Unknown | 0 | | C:/.../bat...E468.raw | not available | raw | Unknown | Unknown | 0 | | C:/.../bat...E469.raw | not available | raw | Unknown | Unknown | 0 | | C:/.../bat...E470.raw | not available | raw | Unknown | Unknown | 0 | | C:/.../bat...E471.raw | not available | raw | Unknown | Unknown | 0 | | C:/.../bat...E472.raw | not available | raw | Unknown | Unknown | 0 | | C:/.../bat...E473.raw | not available | raw | Unknown | Unknown | 0 | | C:/.../bat...E474.raw | not available | raw | Unknown | Unknown | 0 | | C:/.../bat...E475.raw | not available | raw | Unknown | Unknown | 0 | | C:/.../bat...E476.raw | not available | raw | Unknown | Unknown | 0 | | C:/.../bat...E477.raw | not available | raw | Unknown | Unknown | 0 | | C:/.../bat...E478.raw | not available | raw | Unknown | Unknown | 0 | | C:/.../bat...E479.raw | not available | raw | Unknown | Unknown | 0 | | C:/.../bat...E480.raw | not available | raw | Unknown | Unknown | 0 | | C:/.../bat...E481.raw | not available | raw | Unknown | Unknown | 0 | | C:/.../bat...E482.raw | not available | raw | Unknown | Unknown | 0 | | C:/.../bat...E483.raw | not available | raw | Unknown | Unknown | 0 | | C:/.../bat...E484.raw | not available | raw | Unknown | Unknown | 0 | | C:/.../bat...E485.raw | not available | raw | Unknown | Unknown | 0 |  Scanner Manufacturer Information |  |  | | --- | --- | | Name: |  | | Adresse: |  | | Internetseite: |  | | Scanner: |  | | Scansoftware: |  |  Component Information |  |  | | --- | --- | | Beschreibung: |  | | Losnummer: |  | | Seriennummer: |  |  Scan Information |  |  | | --- | --- | | Röhrenspannung: |  | | Röhrenstromstärke: |  | | Scandauer: |  | | Rekonstruktionsdauer: |  | | Gesamtverarbeitungszeit: |  | | Rekonstruktionsalgorithmus: |  | | Scanmethode: |  | | Geometrie: |  | | Integrationszeit: |  | | Filtern: |  | | Projektionsanzahl: |  | | Datum, Zeit: |  | | Benutzer: |  |  Reconstruction Parameters NO RECONSTRUCTION PARAMETERS AVAILABLE  Import Settings |  |  |  |  | | --- | --- | --- | --- | | Source/Type: | Reconstructed Volume | | | | Name: | skelett 2 aus Volumen 1 | | | | Axes swap mode: | XYZT | | | | Mirror axes: | None | | | | Data mapping: | Ramp | | | | Data range source mapping: | -1...1 | | | | Data range destination mapping: | -1...1 | | | | Data type mapping: | Unknown | | | | Voxel skip: | 0 | 0 | 0 | | Auto region of interest: | Off | | Region of interest (min): | 0 | 0 | 0 | | Region of interest (max): | -1 | -1 | -1 | | Slice interpolation mode: | Off | | | | Slice interpolation threshold: | 0 | | | | Resolution [mm]: | 1 | 1 | 1 | | Resampling mode: | Off | | | | Auto histogram mode: | Off | | Lower auto histogram boundary at (%): | 0 | | Upper auto histogram boundary at (%): | 0 |  Analysis Information |  |  | | --- | --- | | Wall thickness: | 0 | | Defect detection: | 0 | | Nominal/actual comparison: | 0 | | Number of reference objects: | 0 |  File List (678) | files (678) | dimensions | format | type | endian | header | | --- | --- | --- | --- | --- | --- | | C:/.../mou...D000.raw | not available | raw | Unknown | Unknown | 0 | | C:/.../mou...D001.raw | not available | raw | Unknown | Unknown | 0 | | C:/.../mou...D002.raw | not available | raw | Unknown | Unknown | 0 | | C:/.../mou...D003.raw | not available | raw | Unknown | Unknown | 0 | | C:/.../mou...D004.raw | not available | raw | Unknown | Unknown | 0 | | C:/.../mou...D005.raw | not available | raw | Unknown | Unknown | 0 | | C:/.../mou...D006.raw | not available | raw | Unknown | Unknown | 0 | | C:/.../mou...D007.raw | not available | raw | Unknown | Unknown | 0 | | C:/.../mou...D008.raw | not available | raw | Unknown | Unknown | 0 | | C:/.../mou...D009.raw | not available | raw | Unknown | Unknown | 0 | | C:/.../mou...D010.raw | not available | raw | Unknown | Unknown | 0 | | C:/.../mou...D011.raw | not available | raw | Unknown | Unknown | 0 | | C:/.../mou...D012.raw | not available | raw | Unknown | Unknown | 0 | | C:/.../mou...D013.raw | not available | raw | Unknown | Unknown | 0 | | C:/.../mou...D014.raw | not available | raw | Unknown | Unknown | 0 | | C:/.../mou...D015.raw | not available | raw | Unknown | Unknown | 0 | | C:/.../mou...D016.raw | not available | raw | Unknown | Unknown | 0 | | C:/.../mou...D017.raw | not available | raw | Unknown | Unknown | 0 | | C:/.../mou...D018.raw | not available | raw | Unknown | Unknown | 0 | | C:/.../mou...D019.raw | not available | raw | Unknown | Unknown | 0 | | C:/.../mou...D020.raw | not available | raw | Unknown | Unknown | 0 | | C:/.../mou...D021.raw | not available | raw | Unknown | Unknown | 0 | | C:/.../mou...D022.raw | not available | raw | Unknown | Unknown | 0 | | C:/.../mou...D023.raw | not available | raw | Unknown | Unknown | 0 | | C:/.../mou...D024.raw | not available | raw | Unknown | Unknown | 0 | | C:/.../mou...D025.raw | not available | raw | Unknown | Unknown | 0 | | C:/.../mou...D026.raw | not available | raw | Unknown | Unknown | 0 | | C:/.../mou...D027.raw | not available | raw | Unknown | Unknown | 0 | | C:/.../mou...D028.raw | not available | raw | Unknown | Unknown | 0 | | C:/.../mou...D029.raw | not available | raw | Unknown | Unknown | 0 | | C:/.../mou...D030.raw | not available | raw | Unknown | Unknown | 0 | | C:/.../mou...D031.raw | not available | raw | Unknown | Unknown | 0 | | C:/.../mou...D032.raw | not available | raw | Unknown | Unknown | 0 | | C:/.../mou...D033.raw | not available | raw | Unknown | Unknown | 0 | | C:/.../mou...D034.raw | not available | raw | Unknown | Unknown | 0 | | C:/.../mou...D035.raw | not available | raw | Unknown | Unknown | 0 | | C:/.../mou...D036.raw | not available | raw | Unknown | Unknown | 0 | | C:/.../mou...D037.raw | not available | raw | Unknown | Unknown | 0 | | C:/.../mou...D038.raw | not available | raw | Unknown | Unknown | 0 | | C:/.../mou...D039.raw | not available | raw | Unknown | Unknown | 0 | | C:/.../mou...D040.raw | not available | raw | Unknown | Unknown | 0 | | C:/.../mou...D041.raw | not available | raw | Unknown | Unknown | 0 | | C:/.../mou...D042.raw | not available | raw | Unknown | Unknown | 0 | | C:/.../mou...D043.raw | not available | raw | Unknown | Unknown | 0 | | C:/.../mou...D044.raw | not available | raw | Unknown | Unknown | 0 | | C:/.../mou...D045.raw | not available | raw | Unknown | Unknown | 0 | | C:/.../mou...D046.raw | not available | raw | Unknown | Unknown | 0 | | C:/.../mou...D047.raw | not available | raw | Unknown | Unknown | 0 | | C:/.../mou...D048.raw | not available | raw | Unknown | Unknown | 0 | | C:/.../mou...D049.raw | not available | raw | Unknown | Unknown | 0 | | C:/.../mou...D050.raw | not available | raw | Unknown | Unknown | 0 | | C:/.../mou...D051.raw | not available | raw | Unknown | Unknown | 0 | | C:/.../mou...D052.raw | not available | raw | Unknown | Unknown | 0 | | C:/.../mou...D053.raw | not available | raw | Unknown | Unknown | 0 | | C:/.../mou...D054.raw | not available | raw | Unknown | Unknown | 0 | | C:/.../mou...D055.raw | not available | raw | Unknown | Unknown | 0 | | C:/.../mou...D056.raw | not available | raw | Unknown | Unknown | 0 | | C:/.../mou...D057.raw | not available | raw | Unknown | Unknown | 0 | | C:/.../mou...D058.raw | not available | raw | Unknown | Unknown | 0 | | C:/.../mou...D059.raw | not available | raw | Unknown | Unknown | 0 | | C:/.../mou...D060.raw | not available | raw | Unknown | Unknown | 0 | | C:/.../mou...D061.raw | not available | raw | Unknown | Unknown | 0 | | C:/.../mou...D062.raw | not available | raw | Unknown | Unknown | 0 | | C:/.../mou...D063.raw | not available | raw | Unknown | Unknown | 0 | | C:/.../mou...D064.raw | not available | raw | Unknown | Unknown | 0 | | C:/.../mou...D065.raw | not available | raw | Unknown | Unknown | 0 | | C:/.../mou...D066.raw | not available | raw | Unknown | Unknown | 0 | | C:/.../mou...D067.raw | not available | raw | Unknown | Unknown | 0 | | C:/.../mou...D068.raw | not available | raw | Unknown | Unknown | 0 | | C:/.../mou...D069.raw | not available | raw | Unknown | Unknown | 0 | | C:/.../mou...D070.raw | not available | raw | Unknown | Unknown | 0 | | C:/.../mou...D071.raw | not available | raw | Unknown | Unknown | 0 | | C:/.../mou...D072.raw | not available | raw | Unknown | Unknown | 0 | | C:/.../mou...D073.raw | not available | raw | Unknown | Unknown | 0 | | C:/.../mou...D074.raw | not available | raw | Unknown | Unknown | 0 | | C:/.../mou...D075.raw | not available | raw | Unknown | Unknown | 0 | | C:/.../mou...D076.raw | not available | raw | Unknown | Unknown | 0 | | C:/.../mou...D077.raw | not available | raw | Unknown | Unknown | 0 | | C:/.../mou...D078.raw | not available | raw | Unknown | Unknown | 0 | | C:/.../mou...D079.raw | not available | raw | Unknown | Unknown | 0 | | C:/.../mou...D080.raw | not available | raw | Unknown | Unknown | 0 | | C:/.../mou...D081.raw | not available | raw | Unknown | Unknown | 0 | | C:/.../mou...D082.raw | not available | raw | Unknown | Unknown | 0 | | C:/.../mou...D083.raw | not available | raw | Unknown | Unknown | 0 | | C:/.../mou...D084.raw | not available | raw | Unknown | Unknown | 0 | | C:/.../mou...D085.raw | not available | raw | Unknown | Unknown | 0 | | C:/.../mou...D086.raw | not available | raw | Unknown | Unknown | 0 | | C:/.../mou...D087.raw | not available | raw | Unknown | Unknown | 0 | | C:/.../mou...D088.raw | not available | raw | Unknown | Unknown | 0 | | C:/.../mou...D089.raw | not available | raw | Unknown | Unknown | 0 | | C:/.../mou...D090.raw | not available | raw | Unknown | Unknown | 0 | | C:/.../mou...D091.raw | not available | raw | Unknown | Unknown | 0 | | C:/.../mou...D092.raw | not available | raw | Unknown | Unknown | 0 | | C:/.../mou...D093.raw | not available | raw | Unknown | Unknown | 0 | | C:/.../mou...D094.raw | not available | raw | Unknown | Unknown | 0 | | C:/.../mou...D095.raw | not available | raw | Unknown | Unknown | 0 | | C:/.../mou...D096.raw | not available | raw | Unknown | Unknown | 0 | | C:/.../mou...D097.raw | not available | raw | Unknown | Unknown | 0 | | C:/.../mou...D098.raw | not available | raw | Unknown | Unknown | 0 | | C:/.../mou...D099.raw | not available | raw | Unknown | Unknown | 0 | | C:/.../mou...D100.raw | not available | raw | Unknown | Unknown | 0 | | C:/.../mou...D101.raw | not available | raw | Unknown | Unknown | 0 | | C:/.../mou...D102.raw | not available | raw | Unknown | Unknown | 0 | | C:/.../mou...D103.raw | not available | raw | Unknown | Unknown | 0 | | C:/.../mou...D104.raw | not available | raw | Unknown | Unknown | 0 | | C:/.../mou...D105.raw | not available | raw | Unknown | Unknown | 0 | | C:/.../mou...D106.raw | not available | raw | Unknown | Unknown | 0 | | C:/.../mou...D107.raw | not available | raw | Unknown | Unknown | 0 | | C:/.../mou...D108.raw | not available | raw | Unknown | Unknown | 0 | | C:/.../mou...D109.raw | not available | raw | Unknown | Unknown | 0 | | C:/.../mou...D110.raw | not available | raw | Unknown | Unknown | 0 | | C:/.../mou...D111.raw | not available | raw | Unknown | Unknown | 0 | | C:/.../mou...D112.raw | not available | raw | Unknown | Unknown | 0 | | C:/.../mou...D113.raw | not available | raw | Unknown | Unknown | 0 | | C:/.../mou...D114.raw | not available | raw | Unknown | Unknown | 0 | | C:/.../mou...D115.raw | not available | raw | Unknown | Unknown | 0 | | C:/.../mou...D116.raw | not available | raw | Unknown | Unknown | 0 | | C:/.../mou...D117.raw | not available | raw | Unknown | Unknown | 0 | | C:/.../mou...D118.raw | not available | raw | Unknown | Unknown | 0 | | C:/.../mou...D119.raw | not available | raw | Unknown | Unknown | 0 | | C:/.../mou...D120.raw | not available | raw | Unknown | Unknown | 0 | | C:/.../mou...D121.raw | not available | raw | Unknown | Unknown | 0 | | C:/.../mou...D122.raw | not available | raw | Unknown | Unknown | 0 | | C:/.../mou...D123.raw | not available | raw | Unknown | Unknown | 0 | | C:/.../mou...D124.raw | not available | raw | Unknown | Unknown | 0 | | C:/.../mou...D125.raw | not available | raw | Unknown | Unknown | 0 | | C:/.../mou...D126.raw | not available | raw | Unknown | Unknown | 0 | | C:/.../mou...D127.raw | not available | raw | Unknown | Unknown | 0 | | C:/.../mou...D128.raw | not available | raw | Unknown | Unknown | 0 | | C:/.../mou...D129.raw | not available | raw | Unknown | Unknown | 0 | | C:/.../mou...D130.raw | not available | raw | Unknown | Unknown | 0 | | C:/.../mou...D131.raw | not available | raw | Unknown | Unknown | 0 | | C:/.../mou...D132.raw | not available | raw | Unknown | Unknown | 0 | | C:/.../mou...D133.raw | not available | raw | Unknown | Unknown | 0 | | C:/.../mou...D134.raw | not available | raw | Unknown | Unknown | 0 | | C:/.../mou...D135.raw | not available | raw | Unknown | Unknown | 0 | | C:/.../mou...D136.raw | not available | raw | Unknown | Unknown | 0 | | C:/.../mou...D137.raw | not available | raw | Unknown | Unknown | 0 | | C:/.../mou...D138.raw | not available | raw | Unknown | Unknown | 0 | | C:/.../mou...D139.raw | not available | raw | Unknown | Unknown | 0 | | C:/.../mou...D140.raw | not available | raw | Unknown | Unknown | 0 | | C:/.../mou...D141.raw | not available | raw | Unknown | Unknown | 0 | | C:/.../mou...D142.raw | not available | raw | Unknown | Unknown | 0 | | C:/.../mou...D143.raw | not available | raw | Unknown | Unknown | 0 | | C:/.../mou...D144.raw | not available | raw | Unknown | Unknown | 0 | | C:/.../mou...D145.raw | not available | raw | Unknown | Unknown | 0 | | C:/.../mou...D146.raw | not available | raw | Unknown | Unknown | 0 | | C:/.../mou...D147.raw | not available | raw | Unknown | Unknown | 0 | | C:/.../mou...D148.raw | not available | raw | Unknown | Unknown | 0 | | C:/.../mou...D149.raw | not available | raw | Unknown | Unknown | 0 | | C:/.../mou...D150.raw | not available | raw | Unknown | Unknown | 0 | | C:/.../mou...D151.raw | not available | raw | Unknown | Unknown | 0 | | C:/.../mou...D152.raw | not available | raw | Unknown | Unknown | 0 | | C:/.../mou...D153.raw | not available | raw | Unknown | Unknown | 0 | | C:/.../mou...D154.raw | not available | raw | Unknown | Unknown | 0 | | C:/.../mou...D155.raw | not available | raw | Unknown | Unknown | 0 | | C:/.../mou...D156.raw | not available | raw | Unknown | Unknown | 0 | | C:/.../mou...D157.raw | not available | raw | Unknown | Unknown | 0 | | C:/.../mou...D158.raw | not available | raw | Unknown | Unknown | 0 | | C:/.../mou...D159.raw | not available | raw | Unknown | Unknown | 0 | | C:/.../mou...D160.raw | not available | raw | Unknown | Unknown | 0 | | C:/.../mou...D161.raw | not available | raw | Unknown | Unknown | 0 | | C:/.../mou...D162.raw | not available | raw | Unknown | Unknown | 0 | | C:/.../mou...D163.raw | not available | raw | Unknown | Unknown | 0 | | C:/.../mou...D164.raw | not available | raw | Unknown | Unknown | 0 | | C:/.../mou...D165.raw | not available | raw | Unknown | Unknown | 0 | | C:/.../mou...D166.raw | not available | raw | Unknown | Unknown | 0 | | C:/.../mou...D167.raw | not available | raw | Unknown | Unknown | 0 | | C:/.../mou...D168.raw | not available | raw | Unknown | Unknown | 0 | | C:/.../mou...D169.raw | not available | raw | Unknown | Unknown | 0 | | C:/.../mou...D170.raw | not available | raw | Unknown | Unknown | 0 | | C:/.../mou...D171.raw | not available | raw | Unknown | Unknown | 0 | | C:/.../mou...D172.raw | not available | raw | Unknown | Unknown | 0 | | C:/.../mou...D173.raw | not available | raw | Unknown | Unknown | 0 | | C:/.../mou...D174.raw | not available | raw | Unknown | Unknown | 0 | | C:/.../mou...D175.raw | not available | raw | Unknown | Unknown | 0 | | C:/.../mou...D176.raw | not available | raw | Unknown | Unknown | 0 | | C:/.../mou...D177.raw | not available | raw | Unknown | Unknown | 0 | | C:/.../mou...D178.raw | not available | raw | Unknown | Unknown | 0 | | C:/.../mou...D179.raw | not available | raw | Unknown | Unknown | 0 | | C:/.../mou...D180.raw | not available | raw | Unknown | Unknown | 0 | | C:/.../mou...D181.raw | not available | raw | Unknown | Unknown | 0 | | C:/.../mou...D182.raw | not available | raw | Unknown | Unknown | 0 | | C:/.../mou...D183.raw | not available | raw | Unknown | Unknown | 0 | | C:/.../mou...D184.raw | not available | raw | Unknown | Unknown | 0 | | C:/.../mou...D185.raw | not available | raw | Unknown | Unknown | 0 | | C:/.../mou...D186.raw | not available | raw | Unknown | Unknown | 0 | | C:/.../mou...D187.raw | not available | raw | Unknown | Unknown | 0 | | C:/.../mou...D188.raw | not available | raw | Unknown | Unknown | 0 | | C:/.../mou...D189.raw | not available | raw | Unknown | Unknown | 0 | | C:/.../mou...D190.raw | not available | raw | Unknown | Unknown | 0 | | C:/.../mou...D191.raw | not available | raw | Unknown | Unknown | 0 | | C:/.../mou...D192.raw | not available | raw | Unknown | Unknown | 0 | | C:/.../mou...D193.raw | not available | raw | Unknown | Unknown | 0 | | C:/.../mou...D194.raw | not available | raw | Unknown | Unknown | 0 | | C:/.../mou...D195.raw | not available | raw | Unknown | Unknown | 0 | | C:/.../mou...D196.raw | not available | raw | Unknown | Unknown | 0 | | C:/.../mou...D197.raw | not available | raw | Unknown | Unknown | 0 | | C:/.../mou...D198.raw | not available | raw | Unknown | Unknown | 0 | | C:/.../mou...D199.raw | not available | raw | Unknown | Unknown | 0 | | C:/.../mou...D200.raw | not available | raw | Unknown | Unknown | 0 | | C:/.../mou...D201.raw | not available | raw | Unknown | Unknown | 0 | | C:/.../mou...D202.raw | not available | raw | Unknown | Unknown | 0 | | C:/.../mou...D203.raw | not available | raw | Unknown | Unknown | 0 | | C:/.../mou...D204.raw | not available | raw | Unknown | Unknown | 0 | | C:/.../mou...D205.raw | not available | raw | Unknown | Unknown | 0 | | C:/.../mou...D206.raw | not available | raw | Unknown | Unknown | 0 | | C:/.../mou...D207.raw | not available | raw | Unknown | Unknown | 0 | | C:/.../mou...D208.raw | not available | raw | Unknown | Unknown | 0 | | C:/.../mou...D209.raw | not available | raw | Unknown | Unknown | 0 | | C:/.../mou...D210.raw | not available | raw | Unknown | Unknown | 0 | | C:/.../mou...D211.raw | not available | raw | Unknown | Unknown | 0 | | C:/.../mou...D212.raw | not available | raw | Unknown | Unknown | 0 | | C:/.../mou...D213.raw | not available | raw | Unknown | Unknown | 0 | | C:/.../mou...D214.raw | not available | raw | Unknown | Unknown | 0 | | C:/.../mou...D215.raw | not available | raw | Unknown | Unknown | 0 | | C:/.../mou...D216.raw | not available | raw | Unknown | Unknown | 0 | | C:/.../mou...D217.raw | not available | raw | Unknown | Unknown | 0 | | C:/.../mou...D218.raw | not available | raw | Unknown | Unknown | 0 | | C:/.../mou...D219.raw | not available | raw | Unknown | Unknown | 0 | | C:/.../mou...D220.raw | not available | raw | Unknown | Unknown | 0 | | C:/.../mou...D221.raw | not available | raw | Unknown | Unknown | 0 | | C:/.../mou...D222.raw | not available | raw | Unknown | Unknown | 0 | | C:/.../mou...D223.raw | not available | raw | Unknown | Unknown | 0 | | C:/.../mou...D224.raw | not available | raw | Unknown | Unknown | 0 | | C:/.../mou...D225.raw | not available | raw | Unknown | Unknown | 0 | | C:/.../mou...D226.raw | not available | raw | Unknown | Unknown | 0 | | C:/.../mou...D227.raw | not available | raw | Unknown | Unknown | 0 | | C:/.../mou...D228.raw | not available | raw | Unknown | Unknown | 0 | | C:/.../mou...D229.raw | not available | raw | Unknown | Unknown | 0 | | C:/.../mou...D230.raw | not available | raw | Unknown | Unknown | 0 | | C:/.../mou...D231.raw | not available | raw | Unknown | Unknown | 0 | | C:/.../mou...D232.raw | not available | raw | Unknown | Unknown | 0 | | C:/.../mou...D233.raw | not available | raw | Unknown | Unknown | 0 | | C:/.../mou...D234.raw | not available | raw | Unknown | Unknown | 0 | | C:/.../mou...D235.raw | not available | raw | Unknown | Unknown | 0 | | C:/.../mou...D236.raw | not available | raw | Unknown | Unknown | 0 | | C:/.../mou...D237.raw | not available | raw | Unknown | Unknown | 0 | | C:/.../mou...D238.raw | not available | raw | Unknown | Unknown | 0 | | C:/.../mou...D239.raw | not available | raw | Unknown | Unknown | 0 | | C:/.../mou...D240.raw | not available | raw | Unknown | Unknown | 0 | | C:/.../mou...D241.raw | not available | raw | Unknown | Unknown | 0 | | C:/.../mou...D242.raw | not available | raw | Unknown | Unknown | 0 | | C:/.../mou...D243.raw | not available | raw | Unknown | Unknown | 0 | | C:/.../mou...D244.raw | not available | raw | Unknown | Unknown | 0 | | C:/.../mou...D245.raw | not available | raw | Unknown | Unknown | 0 | | C:/.../mou...D246.raw | not available | raw | Unknown | Unknown | 0 | | C:/.../mou...D247.raw | not available | raw | Unknown | Unknown | 0 | | C:/.../mou...D248.raw | not available | raw | Unknown | Unknown | 0 | | C:/.../mou...D249.raw | not available | raw | Unknown | Unknown | 0 | | C:/.../mou...D250.raw | not available | raw | Unknown | Unknown | 0 | | C:/.../mou...D251.raw | not available | raw | Unknown | Unknown | 0 | | C:/.../mou...D252.raw | not available | raw | Unknown | Unknown | 0 | | C:/.../mou...D253.raw | not available | raw | Unknown | Unknown | 0 | | C:/.../mou...D254.raw | not available | raw | Unknown | Unknown | 0 | | C:/.../mou...D255.raw | not available | raw | Unknown | Unknown | 0 | | C:/.../mou...D256.raw | not available | raw | Unknown | Unknown | 0 | | C:/.../mou...D257.raw | not available | raw | Unknown | Unknown | 0 | | C:/.../mou...D258.raw | not available | raw | Unknown | Unknown | 0 | | C:/.../mou...D259.raw | not available | raw | Unknown | Unknown | 0 | | C:/.../mou...D260.raw | not available | raw | Unknown | Unknown | 0 | | C:/.../mou...D261.raw | not available | raw | Unknown | Unknown | 0 | | C:/.../mou...D262.raw | not available | raw | Unknown | Unknown | 0 | | C:/.../mou...D263.raw | not available | raw | Unknown | Unknown | 0 | | C:/.../mou...D264.raw | not available | raw | Unknown | Unknown | 0 | | C:/.../mou...D265.raw | not available | raw | Unknown | Unknown | 0 | | C:/.../mou...D266.raw | not available | raw | Unknown | Unknown | 0 | | C:/.../mou...D267.raw | not available | raw | Unknown | Unknown | 0 | | C:/.../mou...D268.raw | not available | raw | Unknown | Unknown | 0 | | C:/.../mou...D269.raw | not available | raw | Unknown | Unknown | 0 | | C:/.../mou...D270.raw | not available | raw | Unknown | Unknown | 0 | | C:/.../mou...D271.raw | not available | raw | Unknown | Unknown | 0 | | C:/.../mou...D272.raw | not available | raw | Unknown | Unknown | 0 | | C:/.../mou...D273.raw | not available | raw | Unknown | Unknown | 0 | | C:/.../mou...D274.raw | not available | raw | Unknown | Unknown | 0 | | C:/.../mou...D275.raw | not available | raw | Unknown | Unknown | 0 | | C:/.../mou...D276.raw | not available | raw | Unknown | Unknown | 0 | | C:/.../mou...D277.raw | not available | raw | Unknown | Unknown | 0 | | C:/.../mou...D278.raw | not available | raw | Unknown | Unknown | 0 | | C:/.../mou...D279.raw | not available | raw | Unknown | Unknown | 0 | | C:/.../mou...D280.raw | not available | raw | Unknown | Unknown | 0 | | C:/.../mou...D281.raw | not available | raw | Unknown | Unknown | 0 | | C:/.../mou...D282.raw | not available | raw | Unknown | Unknown | 0 | | C:/.../mou...D283.raw | not available | raw | Unknown | Unknown | 0 | | C:/.../mou...D284.raw | not available | raw | Unknown | Unknown | 0 | | C:/.../mou...D285.raw | not available | raw | Unknown | Unknown | 0 | | C:/.../mou...D286.raw | not available | raw | Unknown | Unknown | 0 | | C:/.../mou...D287.raw | not available | raw | Unknown | Unknown | 0 | | C:/.../mou...D288.raw | not available | raw | Unknown | Unknown | 0 | | C:/.../mou...D289.raw | not available | raw | Unknown | Unknown | 0 | | C:/.../mou...D290.raw | not available | raw | Unknown | Unknown | 0 | | C:/.../mou...D291.raw | not available | raw | Unknown | Unknown | 0 | | C:/.../mou...D292.raw | not available | raw | Unknown | Unknown | 0 | | C:/.../mou...D293.raw | not available | raw | Unknown | Unknown | 0 | | C:/.../mou...D294.raw | not available | raw | Unknown | Unknown | 0 | | C:/.../mou...D295.raw | not available | raw | Unknown | Unknown | 0 | | C:/.../mou...D296.raw | not available | raw | Unknown | Unknown | 0 | | C:/.../mou...D297.raw | not available | raw | Unknown | Unknown | 0 | | C:/.../mou...D298.raw | not available | raw | Unknown | Unknown | 0 | | C:/.../mou...D299.raw | not available | raw | Unknown | Unknown | 0 | | C:/.../mou...D300.raw | not available | raw | Unknown | Unknown | 0 | | C:/.../mou...D301.raw | not available | raw | Unknown | Unknown | 0 | | C:/.../mou...D302.raw | not available | raw | Unknown | Unknown | 0 | | C:/.../mou...D303.raw | not available | raw | Unknown | Unknown | 0 | | C:/.../mou...D304.raw | not available | raw | Unknown | Unknown | 0 | | C:/.../mou...D305.raw | not available | raw | Unknown | Unknown | 0 | | C:/.../mou...D306.raw | not available | raw | Unknown | Unknown | 0 | | C:/.../mou...D307.raw | not available | raw | Unknown | Unknown | 0 | | C:/.../mou...D308.raw | not available | raw | Unknown | Unknown | 0 | | C:/.../mou...D309.raw | not available | raw | Unknown | Unknown | 0 | | C:/.../mou...D310.raw | not available | raw | Unknown | Unknown | 0 | | C:/.../mou...D311.raw | not available | raw | Unknown | Unknown | 0 | | C:/.../mou...D312.raw | not available | raw | Unknown | Unknown | 0 | | C:/.../mou...D313.raw | not available | raw | Unknown | Unknown | 0 | | C:/.../mou...D314.raw | not available | raw | Unknown | Unknown | 0 | | C:/.../mou...D315.raw | not available | raw | Unknown | Unknown | 0 | | C:/.../mou...D316.raw | not available | raw | Unknown | Unknown | 0 | | C:/.../mou...D317.raw | not available | raw | Unknown | Unknown | 0 | | C:/.../mou...D318.raw | not available | raw | Unknown | Unknown | 0 | | C:/.../mou...D319.raw | not available | raw | Unknown | Unknown | 0 | | C:/.../mou...D320.raw | not available | raw | Unknown | Unknown | 0 | | C:/.../mou...D321.raw | not available | raw | Unknown | Unknown | 0 | | C:/.../mou...D322.raw | not available | raw | Unknown | Unknown | 0 | | C:/.../mou...D323.raw | not available | raw | Unknown | Unknown | 0 | | C:/.../mou...D324.raw | not available | raw | Unknown | Unknown | 0 | | C:/.../mou...D325.raw | not available | raw | Unknown | Unknown | 0 | | C:/.../mou...D326.raw | not available | raw | Unknown | Unknown | 0 | | C:/.../mou...D327.raw | not available | raw | Unknown | Unknown | 0 | | C:/.../mou...D328.raw | not available | raw | Unknown | Unknown | 0 | | C:/.../mou...D329.raw | not available | raw | Unknown | Unknown | 0 | | C:/.../mou...D330.raw | not available | raw | Unknown | Unknown | 0 | | C:/.../mou...D331.raw | not available | raw | Unknown | Unknown | 0 | | C:/.../mou...D332.raw | not available | raw | Unknown | Unknown | 0 | | C:/.../mou...D333.raw | not available | raw | Unknown | Unknown | 0 | | C:/.../mou...D334.raw | not available | raw | Unknown | Unknown | 0 | | C:/.../mou...D335.raw | not available | raw | Unknown | Unknown | 0 | | C:/.../mou...D336.raw | not available | raw | Unknown | Unknown | 0 | | C:/.../mou...D337.raw | not available | raw | Unknown | Unknown | 0 | | C:/.../mou...D338.raw | not available | raw | Unknown | Unknown | 0 | | C:/.../mou...D339.raw | not available | raw | Unknown | Unknown | 0 | | C:/.../mou...D340.raw | not available | raw | Unknown | Unknown | 0 | | C:/.../mou...D341.raw | not available | raw | Unknown | Unknown | 0 | | C:/.../mou...D342.raw | not available | raw | Unknown | Unknown | 0 | | C:/.../mou...D343.raw | not available | raw | Unknown | Unknown | 0 | | C:/.../mou...D344.raw | not available | raw | Unknown | Unknown | 0 | | C:/.../mou...D345.raw | not available | raw | Unknown | Unknown | 0 | | C:/.../mou...D346.raw | not available | raw | Unknown | Unknown | 0 | | C:/.../mou...D347.raw | not available | raw | Unknown | Unknown | 0 | | C:/.../mou...D348.raw | not available | raw | Unknown | Unknown | 0 | | C:/.../mou...D349.raw | not available | raw | Unknown | Unknown | 0 | | C:/.../mou...D350.raw | not available | raw | Unknown | Unknown | 0 | | C:/.../mou...D351.raw | not available | raw | Unknown | Unknown | 0 | | C:/.../mou...D352.raw | not available | raw | Unknown | Unknown | 0 | | C:/.../mou...D353.raw | not available | raw | Unknown | Unknown | 0 | | C:/.../mou...D354.raw | not available | raw | Unknown | Unknown | 0 | | C:/.../mou...D355.raw | not available | raw | Unknown | Unknown | 0 | | C:/.../mou...D356.raw | not available | raw | Unknown | Unknown | 0 | | C:/.../mou...D357.raw | not available | raw | Unknown | Unknown | 0 | | C:/.../mou...D358.raw | not available | raw | Unknown | Unknown | 0 | | C:/.../mou...D359.raw | not available | raw | Unknown | Unknown | 0 | | C:/.../mou...D360.raw | not available | raw | Unknown | Unknown | 0 | | C:/.../mou...D361.raw | not available | raw | Unknown | Unknown | 0 | | C:/.../mou...D362.raw | not available | raw | Unknown | Unknown | 0 | | C:/.../mou...D363.raw | not available | raw | Unknown | Unknown | 0 | | C:/.../mou...D364.raw | not available | raw | Unknown | Unknown | 0 | | C:/.../mou...D365.raw | not available | raw | Unknown | Unknown | 0 | | C:/.../mou...D366.raw | not available | raw | Unknown | Unknown | 0 | | C:/.../mou...D367.raw | not available | raw | Unknown | Unknown | 0 | | C:/.../mou...D368.raw | not available | raw | Unknown | Unknown | 0 | | C:/.../mou...D369.raw | not available | raw | Unknown | Unknown | 0 | | C:/.../mou...D370.raw | not available | raw | Unknown | Unknown | 0 | | C:/.../mou...D371.raw | not available | raw | Unknown | Unknown | 0 | | C:/.../mou...D372.raw | not available | raw | Unknown | Unknown | 0 | | C:/.../mou...D373.raw | not available | raw | Unknown | Unknown | 0 | | C:/.../mou...D374.raw | not available | raw | Unknown | Unknown | 0 | | C:/.../mou...D375.raw | not available | raw | Unknown | Unknown | 0 | | C:/.../mou...D376.raw | not available | raw | Unknown | Unknown | 0 | | C:/.../mou...D377.raw | not available | raw | Unknown | Unknown | 0 | | C:/.../mou...D378.raw | not available | raw | Unknown | Unknown | 0 | | C:/.../mou...D379.raw | not available | raw | Unknown | Unknown | 0 | | C:/.../mou...D380.raw | not available | raw | Unknown | Unknown | 0 | | C:/.../mou...D381.raw | not available | raw | Unknown | Unknown | 0 | | C:/.../mou...D382.raw | not available | raw | Unknown | Unknown | 0 | | C:/.../mou...D383.raw | not available | raw | Unknown | Unknown | 0 | | C:/.../mou...D384.raw | not available | raw | Unknown | Unknown | 0 | | C:/.../mou...D385.raw | not available | raw | Unknown | Unknown | 0 | | C:/.../mou...D386.raw | not available | raw | Unknown | Unknown | 0 | | C:/.../mou...D387.raw | not available | raw | Unknown | Unknown | 0 | | C:/.../mou...D388.raw | not available | raw | Unknown | Unknown | 0 | | C:/.../mou...D389.raw | not available | raw | Unknown | Unknown | 0 | | C:/.../mou...D390.raw | not available | raw | Unknown | Unknown | 0 | | C:/.../mou...D391.raw | not available | raw | Unknown | Unknown | 0 | | C:/.../mou...D392.raw | not available | raw | Unknown | Unknown | 0 | | C:/.../mou...D393.raw | not available | raw | Unknown | Unknown | 0 | | C:/.../mou...D394.raw | not available | raw | Unknown | Unknown | 0 | | C:/.../mou...D395.raw | not available | raw | Unknown | Unknown | 0 | | C:/.../mou...D396.raw | not available | raw | Unknown | Unknown | 0 | | C:/.../mou...D397.raw | not available | raw | Unknown | Unknown | 0 | | C:/.../mou...D398.raw | not available | raw | Unknown | Unknown | 0 | | C:/.../mou...D399.raw | not available | raw | Unknown | Unknown | 0 | | C:/.../mou...D400.raw | not available | raw | Unknown | Unknown | 0 | | C:/.../mou...D401.raw | not available | raw | Unknown | Unknown | 0 | | C:/.../mou...D402.raw | not available | raw | Unknown | Unknown | 0 | | C:/.../mou...D403.raw | not available | raw | Unknown | Unknown | 0 | | C:/.../mou...D404.raw | not available | raw | Unknown | Unknown | 0 | | C:/.../mou...D405.raw | not available | raw | Unknown | Unknown | 0 | | C:/.../mou...D406.raw | not available | raw | Unknown | Unknown | 0 | | C:/.../mou...D407.raw | not available | raw | Unknown | Unknown | 0 | | C:/.../mou...D408.raw | not available | raw | Unknown | Unknown | 0 | | C:/.../mou...D409.raw | not available | raw | Unknown | Unknown | 0 | | C:/.../mou...D410.raw | not available | raw | Unknown | Unknown | 0 | | C:/.../mou...D411.raw | not available | raw | Unknown | Unknown | 0 | | C:/.../mou...D412.raw | not available | raw | Unknown | Unknown | 0 | | C:/.../mou...D413.raw | not available | raw | Unknown | Unknown | 0 | | C:/.../mou...D414.raw | not available | raw | Unknown | Unknown | 0 | | C:/.../mou...D415.raw | not available | raw | Unknown | Unknown | 0 | | C:/.../mou...D416.raw | not available | raw | Unknown | Unknown | 0 | | C:/.../mou...D417.raw | not available | raw | Unknown | Unknown | 0 | | C:/.../mou...D418.raw | not available | raw | Unknown | Unknown | 0 | | C:/.../mou...D419.raw | not available | raw | Unknown | Unknown | 0 | | C:/.../mou...D420.raw | not available | raw | Unknown | Unknown | 0 | | C:/.../mou...D421.raw | not available | raw | Unknown | Unknown | 0 | | C:/.../mou...D422.raw | not available | raw | Unknown | Unknown | 0 | | C:/.../mou...D423.raw | not available | raw | Unknown | Unknown | 0 | | C:/.../mou...D424.raw | not available | raw | Unknown | Unknown | 0 | | C:/.../mou...D425.raw | not available | raw | Unknown | Unknown | 0 | | C:/.../mou...D426.raw | not available | raw | Unknown | Unknown | 0 | | C:/.../mou...D427.raw | not available | raw | Unknown | Unknown | 0 | | C:/.../mou...D428.raw | not available | raw | Unknown | Unknown | 0 | | C:/.../mou...D429.raw | not available | raw | Unknown | Unknown | 0 | | C:/.../mou...D430.raw | not available | raw | Unknown | Unknown | 0 | | C:/.../mou...D431.raw | not available | raw | Unknown | Unknown | 0 | | C:/.../mou...D432.raw | not available | raw | Unknown | Unknown | 0 | | C:/.../mou...D433.raw | not available | raw | Unknown | Unknown | 0 | | C:/.../mou...D434.raw | not available | raw | Unknown | Unknown | 0 | | C:/.../mou...D435.raw | not available | raw | Unknown | Unknown | 0 | | C:/.../mou...D436.raw | not available | raw | Unknown | Unknown | 0 | | C:/.../mou...D437.raw | not available | raw | Unknown | Unknown | 0 | | C:/.../mou...D438.raw | not available | raw | Unknown | Unknown | 0 | | C:/.../mou...D439.raw | not available | raw | Unknown | Unknown | 0 | | C:/.../mou...D440.raw | not available | raw | Unknown | Unknown | 0 | | C:/.../mou...D441.raw | not available | raw | Unknown | Unknown | 0 | | C:/.../mou...D442.raw | not available | raw | Unknown | Unknown | 0 | | C:/.../mou...D443.raw | not available | raw | Unknown | Unknown | 0 | | C:/.../mou...D444.raw | not available | raw | Unknown | Unknown | 0 | | C:/.../mou...D445.raw | not available | raw | Unknown | Unknown | 0 | | C:/.../mou...D446.raw | not available | raw | Unknown | Unknown | 0 | | C:/.../mou...D447.raw | not available | raw | Unknown | Unknown | 0 | | C:/.../mou...D448.raw | not available | raw | Unknown | Unknown | 0 | | C:/.../mou...D449.raw | not available | raw | Unknown | Unknown | 0 | | C:/.../mou...D450.raw | not available | raw | Unknown | Unknown | 0 | | C:/.../mou...D451.raw | not available | raw | Unknown | Unknown | 0 | | C:/.../mou...D452.raw | not available | raw | Unknown | Unknown | 0 | | C:/.../mou...D453.raw | not available | raw | Unknown | Unknown | 0 | | C:/.../mou...D454.raw | not available | raw | Unknown | Unknown | 0 | | C:/.../mou...D455.raw | not available | raw | Unknown | Unknown | 0 | | C:/.../mou...D456.raw | not available | raw | Unknown | Unknown | 0 | | C:/.../mou...D457.raw | not available | raw | Unknown | Unknown | 0 | | C:/.../mou...D458.raw | not available | raw | Unknown | Unknown | 0 | | C:/.../mou...D459.raw | not available | raw | Unknown | Unknown | 0 | | C:/.../mou...D460.raw | not available | raw | Unknown | Unknown | 0 | | C:/.../mou...D461.raw | not available | raw | Unknown | Unknown | 0 | | C:/.../mou...D462.raw | not available | raw | Unknown | Unknown | 0 | | C:/.../mou...D463.raw | not available | raw | Unknown | Unknown | 0 | | C:/.../mou...D464.raw | not available | raw | Unknown | Unknown | 0 | | C:/.../mou...D465.raw | not available | raw | Unknown | Unknown | 0 | | C:/.../mou...D466.raw | not available | raw | Unknown | Unknown | 0 | | C:/.../mou...D467.raw | not available | raw | Unknown | Unknown | 0 | | C:/.../mou...D468.raw | not available | raw | Unknown | Unknown | 0 | | C:/.../mou...D469.raw | not available | raw | Unknown | Unknown | 0 | | C:/.../mou...D470.raw | not available | raw | Unknown | Unknown | 0 | | C:/.../mou...D471.raw | not available | raw | Unknown | Unknown | 0 | | C:/.../mou...D472.raw | not available | raw | Unknown | Unknown | 0 | | C:/.../mou...D473.raw | not available | raw | Unknown | Unknown | 0 | | C:/.../mou...D474.raw | not available | raw | Unknown | Unknown | 0 | | C:/.../mou...D475.raw | not available | raw | Unknown | Unknown | 0 | | C:/.../mou...D476.raw | not available | raw | Unknown | Unknown | 0 | | C:/.../mou...D477.raw | not available | raw | Unknown | Unknown | 0 | | C:/.../mou...D478.raw | not available | raw | Unknown | Unknown | 0 | | C:/.../mou...D479.raw | not available | raw | Unknown | Unknown | 0 | | C:/.../mou...D480.raw | not available | raw | Unknown | Unknown | 0 | | C:/.../mou...D481.raw | not available | raw | Unknown | Unknown | 0 | | C:/.../mou...D482.raw | not available | raw | Unknown | Unknown | 0 | | C:/.../mou...D483.raw | not available | raw | Unknown | Unknown | 0 | | C:/.../mou...D484.raw | not available | raw | Unknown | Unknown | 0 | | C:/.../mou...D485.raw | not available | raw | Unknown | Unknown | 0 | | C:/.../mou...D486.raw | not available | raw | Unknown | Unknown | 0 | | C:/.../mou...D487.raw | not available | raw | Unknown | Unknown | 0 | | C:/.../mou...D488.raw | not available | raw | Unknown | Unknown | 0 | | C:/.../mou...D489.raw | not available | raw | Unknown | Unknown | 0 | | C:/.../mou...D490.raw | not available | raw | Unknown | Unknown | 0 | | C:/.../mou...D491.raw | not available | raw | Unknown | Unknown | 0 | | C:/.../mou...D492.raw | not available | raw | Unknown | Unknown | 0 | | C:/.../mou...D493.raw | not available | raw | Unknown | Unknown | 0 | | C:/.../mou...D494.raw | not available | raw | Unknown | Unknown | 0 | | C:/.../mou...D495.raw | not available | raw | Unknown | Unknown | 0 | | C:/.../mou...D496.raw | not available | raw | Unknown | Unknown | 0 | | C:/.../mou...D497.raw | not available | raw | Unknown | Unknown | 0 | | C:/.../mou...D498.raw | not available | raw | Unknown | Unknown | 0 | | C:/.../mou...D499.raw | not available | raw | Unknown | Unknown | 0 | | C:/.../mou...D500.raw | not available | raw | Unknown | Unknown | 0 | | C:/.../mou...D501.raw | not available | raw | Unknown | Unknown | 0 | | C:/.../mou...D502.raw | not available | raw | Unknown | Unknown | 0 | | C:/.../mou...D503.raw | not available | raw | Unknown | Unknown | 0 | | C:/.../mou...D504.raw | not available | raw | Unknown | Unknown | 0 | | C:/.../mou...D505.raw | not available | raw | Unknown | Unknown | 0 | | C:/.../mou...D506.raw | not available | raw | Unknown | Unknown | 0 | | C:/.../mou...D507.raw | not available | raw | Unknown | Unknown | 0 | | C:/.../mou...D508.raw | not available | raw | Unknown | Unknown | 0 | | C:/.../mou...D509.raw | not available | raw | Unknown | Unknown | 0 | | C:/.../mou...D510.raw | not available | raw | Unknown | Unknown | 0 | | C:/.../mou...D511.raw | not available | raw | Unknown | Unknown | 0 | | C:/.../mou...D512.raw | not available | raw | Unknown | Unknown | 0 | | C:/.../mou...D513.raw | not available | raw | Unknown | Unknown | 0 | | C:/.../mou...D514.raw | not available | raw | Unknown | Unknown | 0 | | C:/.../mou...D515.raw | not available | raw | Unknown | Unknown | 0 | | C:/.../mou...D516.raw | not available | raw | Unknown | Unknown | 0 | | C:/.../mou...D517.raw | not available | raw | Unknown | Unknown | 0 | | C:/.../mou...D518.raw | not available | raw | Unknown | Unknown | 0 | | C:/.../mou...D519.raw | not available | raw | Unknown | Unknown | 0 | | C:/.../mou...D520.raw | not available | raw | Unknown | Unknown | 0 | | C:/.../mou...D521.raw | not available | raw | Unknown | Unknown | 0 | | C:/.../mou...D522.raw | not available | raw | Unknown | Unknown | 0 | | C:/.../mou...D523.raw | not available | raw | Unknown | Unknown | 0 | | C:/.../mou...D524.raw | not available | raw | Unknown | Unknown | 0 | | C:/.../mou...D525.raw | not available | raw | Unknown | Unknown | 0 | | C:/.../mou...D526.raw | not available | raw | Unknown | Unknown | 0 | | C:/.../mou...D527.raw | not available | raw | Unknown | Unknown | 0 | | C:/.../mou...D528.raw | not available | raw | Unknown | Unknown | 0 | | C:/.../mou...D529.raw | not available | raw | Unknown | Unknown | 0 | | C:/.../mou...D530.raw | not available | raw | Unknown | Unknown | 0 | | C:/.../mou...D531.raw | not available | raw | Unknown | Unknown | 0 | | C:/.../mou...D532.raw | not available | raw | Unknown | Unknown | 0 | | C:/.../mou...D533.raw | not available | raw | Unknown | Unknown | 0 | | C:/.../mou...D534.raw | not available | raw | Unknown | Unknown | 0 | | C:/.../mou...D535.raw | not available | raw | Unknown | Unknown | 0 | | C:/.../mou...D536.raw | not available | raw | Unknown | Unknown | 0 | | C:/.../mou...D537.raw | not available | raw | Unknown | Unknown | 0 | | C:/.../mou...D538.raw | not available | raw | Unknown | Unknown | 0 | | C:/.../mou...D539.raw | not available | raw | Unknown | Unknown | 0 | | C:/.../mou...D540.raw | not available | raw | Unknown | Unknown | 0 | | C:/.../mou...D541.raw | not available | raw | Unknown | Unknown | 0 | | C:/.../mou...D542.raw | not available | raw | Unknown | Unknown | 0 | | C:/.../mou...D543.raw | not available | raw | Unknown | Unknown | 0 | | C:/.../mou...D544.raw | not available | raw | Unknown | Unknown | 0 | | C:/.../mou...D545.raw | not available | raw | Unknown | Unknown | 0 | | C:/.../mou...D546.raw | not available | raw | Unknown | Unknown | 0 | | C:/.../mou...D547.raw | not available | raw | Unknown | Unknown | 0 | | C:/.../mou...D548.raw | not available | raw | Unknown | Unknown | 0 | | C:/.../mou...D549.raw | not available | raw | Unknown | Unknown | 0 | | C:/.../mou...D550.raw | not available | raw | Unknown | Unknown | 0 | | C:/.../mou...D551.raw | not available | raw | Unknown | Unknown | 0 | | C:/.../mou...D552.raw | not available | raw | Unknown | Unknown | 0 | | C:/.../mou...D553.raw | not available | raw | Unknown | Unknown | 0 | | C:/.../mou...D554.raw | not available | raw | Unknown | Unknown | 0 | | C:/.../mou...D555.raw | not available | raw | Unknown | Unknown | 0 | | C:/.../mou...D556.raw | not available | raw | Unknown | Unknown | 0 | | C:/.../mou...D557.raw | not available | raw | Unknown | Unknown | 0 | | C:/.../mou...D558.raw | not available | raw | Unknown | Unknown | 0 | | C:/.../mou...D559.raw | not available | raw | Unknown | Unknown | 0 | | C:/.../mou...D560.raw | not available | raw | Unknown | Unknown | 0 | | C:/.../mou...D561.raw | not available | raw | Unknown | Unknown | 0 | | C:/.../mou...D562.raw | not available | raw | Unknown | Unknown | 0 | | C:/.../mou...D563.raw | not available | raw | Unknown | Unknown | 0 | | C:/.../mou...D564.raw | not available | raw | Unknown | Unknown | 0 | | C:/.../mou...D565.raw | not available | raw | Unknown | Unknown | 0 | | C:/.../mou...D566.raw | not available | raw | Unknown | Unknown | 0 | | C:/.../mou...D567.raw | not available | raw | Unknown | Unknown | 0 | | C:/.../mou...D568.raw | not available | raw | Unknown | Unknown | 0 | | C:/.../mou...D569.raw | not available | raw | Unknown | Unknown | 0 | | C:/.../mou...D570.raw | not available | raw | Unknown | Unknown | 0 | | C:/.../mou...D571.raw | not available | raw | Unknown | Unknown | 0 | | C:/.../mou...D572.raw | not available | raw | Unknown | Unknown | 0 | | C:/.../mou...D573.raw | not available | raw | Unknown | Unknown | 0 | | C:/.../mou...D574.raw | not available | raw | Unknown | Unknown | 0 | | C:/.../mou...D575.raw | not available | raw | Unknown | Unknown | 0 | | C:/.../mou...D576.raw | not available | raw | Unknown | Unknown | 0 | | C:/.../mou...D577.raw | not available | raw | Unknown | Unknown | 0 | | C:/.../mou...D578.raw | not available | raw | Unknown | Unknown | 0 | | C:/.../mou...D579.raw | not available | raw | Unknown | Unknown | 0 | | C:/.../mou...D580.raw | not available | raw | Unknown | Unknown | 0 | | C:/.../mou...D581.raw | not available | raw | Unknown | Unknown | 0 | | C:/.../mou...D582.raw | not available | raw | Unknown | Unknown | 0 | | C:/.../mou...D583.raw | not available | raw | Unknown | Unknown | 0 | | C:/.../mou...D584.raw | not available | raw | Unknown | Unknown | 0 | | C:/.../mou...D585.raw | not available | raw | Unknown | Unknown | 0 | | C:/.../mou...D586.raw | not available | raw | Unknown | Unknown | 0 | | C:/.../mou...D587.raw | not available | raw | Unknown | Unknown | 0 | | C:/.../mou...D588.raw | not available | raw | Unknown | Unknown | 0 | | C:/.../mou...D589.raw | not available | raw | Unknown | Unknown | 0 | | C:/.../mou...D590.raw | not available | raw | Unknown | Unknown | 0 | | C:/.../mou...D591.raw | not available | raw | Unknown | Unknown | 0 | | C:/.../mou...D592.raw | not available | raw | Unknown | Unknown | 0 | | C:/.../mou...D593.raw | not available | raw | Unknown | Unknown | 0 | | C:/.../mou...D594.raw | not available | raw | Unknown | Unknown | 0 | | C:/.../mou...D595.raw | not available | raw | Unknown | Unknown | 0 | | C:/.../mou...D596.raw | not available | raw | Unknown | Unknown | 0 | | C:/.../mou...D597.raw | not available | raw | Unknown | Unknown | 0 | | C:/.../mou...D598.raw | not available | raw | Unknown | Unknown | 0 | | C:/.../mou...D599.raw | not available | raw | Unknown | Unknown | 0 | | C:/.../mou...D600.raw | not available | raw | Unknown | Unknown | 0 | | C:/.../mou...D601.raw | not available | raw | Unknown | Unknown | 0 | | C:/.../mou...D602.raw | not available | raw | Unknown | Unknown | 0 | | C:/.../mou...D603.raw | not available | raw | Unknown | Unknown | 0 | | C:/.../mou...D604.raw | not available | raw | Unknown | Unknown | 0 | | C:/.../mou...D605.raw | not available | raw | Unknown | Unknown | 0 | | C:/.../mou...D606.raw | not available | raw | Unknown | Unknown | 0 | | C:/.../mou...D607.raw | not available | raw | Unknown | Unknown | 0 | | C:/.../mou...D608.raw | not available | raw | Unknown | Unknown | 0 | | C:/.../mou...D609.raw | not available | raw | Unknown | Unknown | 0 | | C:/.../mou...D610.raw | not available | raw | Unknown | Unknown | 0 | | C:/.../mou...D611.raw | not available | raw | Unknown | Unknown | 0 | | C:/.../mou...D612.raw | not available | raw | Unknown | Unknown | 0 | | C:/.../mou...D613.raw | not available | raw | Unknown | Unknown | 0 | | C:/.../mou...D614.raw | not available | raw | Unknown | Unknown | 0 | | C:/.../mou...D615.raw | not available | raw | Unknown | Unknown | 0 | | C:/.../mou...D616.raw | not available | raw | Unknown | Unknown | 0 | | C:/.../mou...D617.raw | not available | raw | Unknown | Unknown | 0 | | C:/.../mou...D618.raw | not available | raw | Unknown | Unknown | 0 | | C:/.../mou...D619.raw | not available | raw | Unknown | Unknown | 0 | | C:/.../mou...D620.raw | not available | raw | Unknown | Unknown | 0 | | C:/.../mou...D621.raw | not available | raw | Unknown | Unknown | 0 | | C:/.../mou...D622.raw | not available | raw | Unknown | Unknown | 0 | | C:/.../mou...D623.raw | not available | raw | Unknown | Unknown | 0 | | C:/.../mou...D624.raw | not available | raw | Unknown | Unknown | 0 | | C:/.../mou...D625.raw | not available | raw | Unknown | Unknown | 0 | | C:/.../mou...D626.raw | not available | raw | Unknown | Unknown | 0 | | C:/.../mou...D627.raw | not available | raw | Unknown | Unknown | 0 | | C:/.../mou...D628.raw | not available | raw | Unknown | Unknown | 0 | | C:/.../mou...D629.raw | not available | raw | Unknown | Unknown | 0 | | C:/.../mou...D630.raw | not available | raw | Unknown | Unknown | 0 | | C:/.../mou...D631.raw | not available | raw | Unknown | Unknown | 0 | | C:/.../mou...D632.raw | not available | raw | Unknown | Unknown | 0 | | C:/.../mou...D633.raw | not available | raw | Unknown | Unknown | 0 | | C:/.../mou...D634.raw | not available | raw | Unknown | Unknown | 0 | | C:/.../mou...D635.raw | not available | raw | Unknown | Unknown | 0 | | C:/.../mou...D636.raw | not available | raw | Unknown | Unknown | 0 | | C:/.../mou...D637.raw | not available | raw | Unknown | Unknown | 0 | | C:/.../mou...D638.raw | not available | raw | Unknown | Unknown | 0 | | C:/.../mou...D639.raw | not available | raw | Unknown | Unknown | 0 | | C:/.../mou...D640.raw | not available | raw | Unknown | Unknown | 0 | | C:/.../mou...D641.raw | not available | raw | Unknown | Unknown | 0 | | C:/.../mou...D642.raw | not available | raw | Unknown | Unknown | 0 | | C:/.../mou...D643.raw | not available | raw | Unknown | Unknown | 0 | | C:/.../mou...D644.raw | not available | raw | Unknown | Unknown | 0 | | C:/.../mou...D645.raw | not available | raw | Unknown | Unknown | 0 | | C:/.../mou...D646.raw | not available | raw | Unknown | Unknown | 0 | | C:/.../mou...D647.raw | not available | raw | Unknown | Unknown | 0 | | C:/.../mou...D648.raw | not available | raw | Unknown | Unknown | 0 | | C:/.../mou...D649.raw | not available | raw | Unknown | Unknown | 0 | | C:/.../mou...D650.raw | not available | raw | Unknown | Unknown | 0 | | C:/.../mou...D651.raw | not available | raw | Unknown | Unknown | 0 | | C:/.../mou...D652.raw | not available | raw | Unknown | Unknown | 0 | | C:/.../mou...D653.raw | not available | raw | Unknown | Unknown | 0 | | C:/.../mou...D654.raw | not available | raw | Unknown | Unknown | 0 | | C:/.../mou...D655.raw | not available | raw | Unknown | Unknown | 0 | | C:/.../mou...D656.raw | not available | raw | Unknown | Unknown | 0 | | C:/.../mou...D657.raw | not available | raw | Unknown | Unknown | 0 | | C:/.../mou...D658.raw | not available | raw | Unknown | Unknown | 0 | | C:/.../mou...D659.raw | not available | raw | Unknown | Unknown | 0 | | C:/.../mou...D660.raw | not available | raw | Unknown | Unknown | 0 | | C:/.../mou...D661.raw | not available | raw | Unknown | Unknown | 0 | | C:/.../mou...D662.raw | not available | raw | Unknown | Unknown | 0 | | C:/.../mou...D663.raw | not available | raw | Unknown | Unknown | 0 | | C:/.../mou...D664.raw | not available | raw | Unknown | Unknown | 0 | | C:/.../mou...D665.raw | not available | raw | Unknown | Unknown | 0 | | C:/.../mou...D666.raw | not available | raw | Unknown | Unknown | 0 | | C:/.../mou...D667.raw | not available | raw | Unknown | Unknown | 0 | | C:/.../mou...D668.raw | not available | raw | Unknown | Unknown | 0 | | C:/.../mou...D669.raw | not available | raw | Unknown | Unknown | 0 | | C:/.../mou...D670.raw | not available | raw | Unknown | Unknown | 0 | | C:/.../mou...D671.raw | not available | raw | Unknown | Unknown | 0 | | C:/.../mou...D672.raw | not available | raw | Unknown | Unknown | 0 | | C:/.../mou...D673.raw | not available | raw | Unknown | Unknown | 0 | | C:/.../mou...D674.raw | not available | raw | Unknown | Unknown | 0 | | C:/.../mou...D675.raw | not available | raw | Unknown | Unknown | 0 | | C:/.../mou...D676.raw | not available | raw | Unknown | Unknown | 0 | | C:/.../mou...D677.raw | not available | raw | Unknown | Unknown | 0 |  Scanner Manufacturer Information |  |  | | --- | --- | | Name: |  | | Adresse: |  | | Internetseite: |  | | Scanner: |  | | Scansoftware: |  |  Component Information |  |  | | --- | --- | | Beschreibung: |  | | Losnummer: |  | | Seriennummer: |  |  Scan Information |  |  | | --- | --- | | Röhrenspannung: |  | | Röhrenstromstärke: |  | | Scandauer: |  | | Rekonstruktionsdauer: |  | | Gesamtverarbeitungszeit: |  | | Rekonstruktionsalgorithmus: |  | | Scanmethode: |  | | Geometrie: |  | | Integrationszeit: |  | | Filtern: |  | | Projektionsanzahl: |  | | Datum, Zeit: |  | | Benutzer: |  |  Reconstruction Parameters NO RECONSTRUCTION PARAMETERS AVAILABLE  Import Settings |  |  |  |  | | --- | --- | --- | --- | | Source/Type: | Reconstructed Volume | | | | Name: | Region 1 aus Volumen 1 | | | | Axes swap mode: | XYZT | | | | Mirror axes: | None | | | | Data mapping: | Ramp | | | | Data range source mapping: | -1...1 | | | | Data range destination mapping: | -1...1 | | | | Data type mapping: | Unknown | | | | Voxel skip: | 0 | 0 | 0 | | Auto region of interest: | Off | | Region of interest (min): | 0 | 0 | 0 | | Region of interest (max): | -1 | -1 | -1 | | Slice interpolation mode: | Off | | | | Slice interpolation threshold: | 0 | | | | Resolution [mm]: | 1 | 1 | 1 | | Resampling mode: | Off | | | | Auto histogram mode: | Off | | Lower auto histogram boundary at (%): | 0 | | Upper auto histogram boundary at (%): | 0 |  Analysis Information |  |  | | --- | --- | | Wall thickness: | 0 | | Defect detection: | 0 | | Nominal/actual comparison: | 0 | | Number of reference objects: | 0 |  File List (678) | files (678) | dimensions | format | type | endian | header | | --- | --- | --- | --- | --- | --- | | C:/.../mou...D000.raw | not available | raw | Unknown | Unknown | 0 | | C:/.../mou...D001.raw | not available | raw | Unknown | Unknown | 0 | | C:/.../mou...D002.raw | not available | raw | Unknown | Unknown | 0 | | C:/.../mou...D003.raw | not available | raw | Unknown | Unknown | 0 | | C:/.../mou...D004.raw | not available | raw | Unknown | Unknown | 0 | | C:/.../mou...D005.raw | not available | raw | Unknown | Unknown | 0 | | C:/.../mou...D006.raw | not available | raw | Unknown | Unknown | 0 | | C:/.../mou...D007.raw | not available | raw | Unknown | Unknown | 0 | | C:/.../mou...D008.raw | not available | raw | Unknown | Unknown | 0 | | C:/.../mou...D009.raw | not available | raw | Unknown | Unknown | 0 | | C:/.../mou...D010.raw | not available | raw | Unknown | Unknown | 0 | | C:/.../mou...D011.raw | not available | raw | Unknown | Unknown | 0 | | C:/.../mou...D012.raw | not available | raw | Unknown | Unknown | 0 | | C:/.../mou...D013.raw | not available | raw | Unknown | Unknown | 0 | | C:/.../mou...D014.raw | not available | raw | Unknown | Unknown | 0 | | C:/.../mou...D015.raw | not available | raw | Unknown | Unknown | 0 | | C:/.../mou...D016.raw | not available | raw | Unknown | Unknown | 0 | | C:/.../mou...D017.raw | not available | raw | Unknown | Unknown | 0 | | C:/.../mou...D018.raw | not available | raw | Unknown | Unknown | 0 | | C:/.../mou...D019.raw | not available | raw | Unknown | Unknown | 0 | | C:/.../mou...D020.raw | not available | raw | Unknown | Unknown | 0 | | C:/.../mou...D021.raw | not available | raw | Unknown | Unknown | 0 | | C:/.../mou...D022.raw | not available | raw | Unknown | Unknown | 0 | | C:/.../mou...D023.raw | not available | raw | Unknown | Unknown | 0 | | C:/.../mou...D024.raw | not available | raw | Unknown | Unknown | 0 | | C:/.../mou...D025.raw | not available | raw | Unknown | Unknown | 0 | | C:/.../mou...D026.raw | not available | raw | Unknown | Unknown | 0 | | C:/.../mou...D027.raw | not available | raw | Unknown | Unknown | 0 | | C:/.../mou...D028.raw | not available | raw | Unknown | Unknown | 0 | | C:/.../mou...D029.raw | not available | raw | Unknown | Unknown | 0 | | C:/.../mou...D030.raw | not available | raw | Unknown | Unknown | 0 | | C:/.../mou...D031.raw | not available | raw | Unknown | Unknown | 0 | | C:/.../mou...D032.raw | not available | raw | Unknown | Unknown | 0 | | C:/.../mou...D033.raw | not available | raw | Unknown | Unknown | 0 | | C:/.../mou...D034.raw | not available | raw | Unknown | Unknown | 0 | | C:/.../mou...D035.raw | not available | raw | Unknown | Unknown | 0 | | C:/.../mou...D036.raw | not available | raw | Unknown | Unknown | 0 | | C:/.../mou...D037.raw | not available | raw | Unknown | Unknown | 0 | | C:/.../mou...D038.raw | not available | raw | Unknown | Unknown | 0 | | C:/.../mou...D039.raw | not available | raw | Unknown | Unknown | 0 | | C:/.../mou...D040.raw | not available | raw | Unknown | Unknown | 0 | | C:/.../mou...D041.raw | not available | raw | Unknown | Unknown | 0 | | C:/.../mou...D042.raw | not available | raw | Unknown | Unknown | 0 | | C:/.../mou...D043.raw | not available | raw | Unknown | Unknown | 0 | | C:/.../mou...D044.raw | not available | raw | Unknown | Unknown | 0 | | C:/.../mou...D045.raw | not available | raw | Unknown | Unknown | 0 | | C:/.../mou...D046.raw | not available | raw | Unknown | Unknown | 0 | | C:/.../mou...D047.raw | not available | raw | Unknown | Unknown | 0 | | C:/.../mou...D048.raw | not available | raw | Unknown | Unknown | 0 | | C:/.../mou...D049.raw | not available | raw | Unknown | Unknown | 0 | | C:/.../mou...D050.raw | not available | raw | Unknown | Unknown | 0 | | C:/.../mou...D051.raw | not available | raw | Unknown | Unknown | 0 | | C:/.../mou...D052.raw | not available | raw | Unknown | Unknown | 0 | | C:/.../mou...D053.raw | not available | raw | Unknown | Unknown | 0 | | C:/.../mou...D054.raw | not available | raw | Unknown | Unknown | 0 | | C:/.../mou...D055.raw | not available | raw | Unknown | Unknown | 0 | | C:/.../mou...D056.raw | not available | raw | Unknown | Unknown | 0 | | C:/.../mou...D057.raw | not available | raw | Unknown | Unknown | 0 | | C:/.../mou...D058.raw | not available | raw | Unknown | Unknown | 0 | | C:/.../mou...D059.raw | not available | raw | Unknown | Unknown | 0 | | C:/.../mou...D060.raw | not available | raw | Unknown | Unknown | 0 | | C:/.../mou...D061.raw | not available | raw | Unknown | Unknown | 0 | | C:/.../mou...D062.raw | not available | raw | Unknown | Unknown | 0 | | C:/.../mou...D063.raw | not available | raw | Unknown | Unknown | 0 | | C:/.../mou...D064.raw | not available | raw | Unknown | Unknown | 0 | | C:/.../mou...D065.raw | not available | raw | Unknown | Unknown | 0 | | C:/.../mou...D066.raw | not available | raw | Unknown | Unknown | 0 | | C:/.../mou...D067.raw | not available | raw | Unknown | Unknown | 0 | | C:/.../mou...D068.raw | not available | raw | Unknown | Unknown | 0 | | C:/.../mou...D069.raw | not available | raw | Unknown | Unknown | 0 | | C:/.../mou...D070.raw | not available | raw | Unknown | Unknown | 0 | | C:/.../mou...D071.raw | not available | raw | Unknown | Unknown | 0 | | C:/.../mou...D072.raw | not available | raw | Unknown | Unknown | 0 | | C:/.../mou...D073.raw | not available | raw | Unknown | Unknown | 0 | | C:/.../mou...D074.raw | not available | raw | Unknown | Unknown | 0 | | C:/.../mou...D075.raw | not available | raw | Unknown | Unknown | 0 | | C:/.../mou...D076.raw | not available | raw | Unknown | Unknown | 0 | | C:/.../mou...D077.raw | not available | raw | Unknown | Unknown | 0 | | C:/.../mou...D078.raw | not available | raw | Unknown | Unknown | 0 | | C:/.../mou...D079.raw | not available | raw | Unknown | Unknown | 0 | | C:/.../mou...D080.raw | not available | raw | Unknown | Unknown | 0 | | C:/.../mou...D081.raw | not available | raw | Unknown | Unknown | 0 | | C:/.../mou...D082.raw | not available | raw | Unknown | Unknown | 0 | | C:/.../mou...D083.raw | not available | raw | Unknown | Unknown | 0 | | C:/.../mou...D084.raw | not available | raw | Unknown | Unknown | 0 | | C:/.../mou...D085.raw | not available | raw | Unknown | Unknown | 0 | | C:/.../mou...D086.raw | not available | raw | Unknown | Unknown | 0 | | C:/.../mou...D087.raw | not available | raw | Unknown | Unknown | 0 | | C:/.../mou...D088.raw | not available | raw | Unknown | Unknown | 0 | | C:/.../mou...D089.raw | not available | raw | Unknown | Unknown | 0 | | C:/.../mou...D090.raw | not available | raw | Unknown | Unknown | 0 | | C:/.../mou...D091.raw | not available | raw | Unknown | Unknown | 0 | | C:/.../mou...D092.raw | not available | raw | Unknown | Unknown | 0 | | C:/.../mou...D093.raw | not available | raw | Unknown | Unknown | 0 | | C:/.../mou...D094.raw | not available | raw | Unknown | Unknown | 0 | | C:/.../mou...D095.raw | not available | raw | Unknown | Unknown | 0 | | C:/.../mou...D096.raw | not available | raw | Unknown | Unknown | 0 | | C:/.../mou...D097.raw | not available | raw | Unknown | Unknown | 0 | | C:/.../mou...D098.raw | not available | raw | Unknown | Unknown | 0 | | C:/.../mou...D099.raw | not available | raw | Unknown | Unknown | 0 | | C:/.../mou...D100.raw | not available | raw | Unknown | Unknown | 0 | | C:/.../mou...D101.raw | not available | raw | Unknown | Unknown | 0 | | C:/.../mou...D102.raw | not available | raw | Unknown | Unknown | 0 | | C:/.../mou...D103.raw | not available | raw | Unknown | Unknown | 0 | | C:/.../mou...D104.raw | not available | raw | Unknown | Unknown | 0 | | C:/.../mou...D105.raw | not available | raw | Unknown | Unknown | 0 | | C:/.../mou...D106.raw | not available | raw | Unknown | Unknown | 0 | | C:/.../mou...D107.raw | not available | raw | Unknown | Unknown | 0 | | C:/.../mou...D108.raw | not available | raw | Unknown | Unknown | 0 | | C:/.../mou...D109.raw | not available | raw | Unknown | Unknown | 0 | | C:/.../mou...D110.raw | not available | raw | Unknown | Unknown | 0 | | C:/.../mou...D111.raw | not available | raw | Unknown | Unknown | 0 | | C:/.../mou...D112.raw | not available | raw | Unknown | Unknown | 0 | | C:/.../mou...D113.raw | not available | raw | Unknown | Unknown | 0 | | C:/.../mou...D114.raw | not available | raw | Unknown | Unknown | 0 | | C:/.../mou...D115.raw | not available | raw | Unknown | Unknown | 0 | | C:/.../mou...D116.raw | not available | raw | Unknown | Unknown | 0 | | C:/.../mou...D117.raw | not available | raw | Unknown | Unknown | 0 | | C:/.../mou...D118.raw | not available | raw | Unknown | Unknown | 0 | | C:/.../mou...D119.raw | not available | raw | Unknown | Unknown | 0 | | C:/.../mou...D120.raw | not available | raw | Unknown | Unknown | 0 | | C:/.../mou...D121.raw | not available | raw | Unknown | Unknown | 0 | | C:/.../mou...D122.raw | not available | raw | Unknown | Unknown | 0 | | C:/.../mou...D123.raw | not available | raw | Unknown | Unknown | 0 | | C:/.../mou...D124.raw | not available | raw | Unknown | Unknown | 0 | | C:/.../mou...D125.raw | not available | raw | Unknown | Unknown | 0 | | C:/.../mou...D12
[truncated: 41,291 more chars]
